# Did an ancient chlamydial endosymbiosis facilitate the establishment of primary plastids?

**DOI:** 10.1186/gb-2007-8-6-r99

**Published:** 2007-06-04

**Authors:** Jinling Huang, Johann Peter Gogarten

**Affiliations:** 1 Department of Biology, Howell Science Complex, East Carolina University, Greenville, NC 27858, USA; 2 NASA Astrobiology Institute at Marine Biological Laboratory, Woods Hole, MA 02543, USA; 3 Department of Molecular and Cell Biology, University of Connecticut, 91 North Eagleville Road, Storrs, CT 06269-3125, USA

## Abstract

**Background:**

Ancient endosymbioses are responsible for the origins of mitochondria and plastids, and they contribute to the divergence of several major eukaryotic groups. Although chlamydiae, a group of obligate intracellular bacteria, are not found in plants, an unexpected number of chlamydial genes are most similar to plant homologs, which, interestingly, often contain a plastid-targeting signal. This observation has prompted several hypotheses, including gene transfer between chlamydiae and plant-related groups and an ancestral relationship between chlamydiae and cyanobacteria.

**Results:**

We conducted phylogenomic analyses of the red alga *Cyanidioschyzon merolae *to identify genes specifically related to chlamydial homologs. We show that at least 21 genes were transferred between chlamydiae and primary photosynthetic eukaryotes, with the donor most similar to the environmental *Protochlamydia*. Such an unusually high number of transferred genes suggests an ancient chlamydial endosymbiosis with the ancestral primary photosynthetic eukaryote. We hypothesize that three organisms were involved in establishing the primary photosynthetic lineage: the eukaryotic host cell, the cyanobacterial endosymbiont that provided photosynthetic capability, and a chlamydial endosymbiont or parasite that facilitated the establishment of the cyanobacterial endosymbiont.

**Conclusion:**

Our findings provide a glimpse into the complex interactions that were necessary to establish the primary endosymbiotic relationship between plastid and host cytoplasms, and thereby explain the rarity with which long-term successful endosymbiotic relationships between heterotrophs and photoautotrophs were established. Our data also provide strong and independent support for a common origin of all primary photosynthetic eukaryotes and of the plastids they harbor.

## Abstract 2

Phylogenomic analyses of the red alga *Cyanidioschyzon merolae *shows that at least 21 genes were transferred between chlamydiae and primary photosynthetic eukaryotes, suggesting an ancient chlamydial endosymbiosis with the ancestral primary photosynthetic eukaryote.

## Background

Ancient symbioses are responsible for some of the major eukaryotic innovations. It is widely accepted that mitochondria and plastids are derived respectively from an α-proteobacterial and a cyanobacterial endosymbiont in early eukaryotes [1]. It also has been suggested that the nucleus, a hallmark of eukaryotic cells, either arose directly from or was mediated by an ancient symbiosis between archaeal and bacterial partners [2-7]. Additionally, secondary and tertiary symbioses through engulfment of a plastid-containing cell played an important role in the evolution of several major eukaryotic lineages, including heterokonts, apicomplexans, dinoflagellates, euglenids, and others [8-12]. Undoubtedly, the evolution of extant eukaryotes was significantly shaped by past symbioses.

Chlamydiae are a group of obligate intracellular bacteria of uncertain evolutionary position [13-15]. Many chlamydiae, including *Chlamydophila pneumoniae *and *Chlamydia trachomatis*, are important pathogens in humans and other animals [16] whereas others such as *Protochlamydia, Neochlamydia*, and *Fritschea *are endosymbionts in environmental amoebae and insects [17,18]. Although the available evidence suggests increasing chlamydial diversity in free-living amoebae and in the environment [19], thus far no chlamydial species has been reported in photosynthetic eukaryotes or plastid-containing lineages. However, chlamydial genome analyses revealed an unexpected number of genes that are most similar to plant homologs [20,21], which, interestingly, often contain a plastid-targeting signal [13]. This observation has prompted several hypotheses, notably an ancestral evolutionary relationship between cyanobacteria (plastids) and chlamydiae [13] and gene transfer between the two groups with the donor being either chlamydiae [22,23] or plant-related groups [21,24,25]. Additionally, it has also been suggested that plants might have acquired these genes from mitochondria [26] or through intermediate vectors such as insects [17].

Reconstructing possible evolutionary scenarios that explain the chlamydial and plant sequence similarity requires an understanding of the taxonomic distribution and the origin of all involved genes. However, available phylogenetic data from chlamydial genome analyses often suffer from small taxonomic sample size [20,21]. Most other relevant studies are heavily biased toward the gene encoding ATP/ADP translocase, which has an uncertain evolutionary origin and a narrow distribution, mainly in obligate intracellular bacteria (chlamydiae and rickettsiae) and photosynthetic eukaryotes [22,25-28]. The evolutionary history of a single gene, even if correctly interpreted, might not reflect those of others. If a single evolutionary event underlies the current observation of chlamydial and plant sequence similarity, then a compatible evolutionary history of multiple genes should provide more convincing evidence.

Given the common belief that all primary photosynthetic eukaryotes, including glaucophytes, red algae, and green plants, share a common ancestry [11,29,30], we undertook a phylogenomic analysis of *Cyanidioschyzon merolae *(the only red alga whose complete genome sequence is currently available) to search for genes that are evolutionarily related to chlamydial homologs. Our data suggest a likely ancient symbiosis (*sensu *deBary; including mutualism, commensalisms, and parasitism) [31] between a chlamydial endosymbiont and the ancestor of primary photosynthetic eukaryotes. The ancient chlamydial endosymbiont contributed genes to the nuclear genome of primary photosynthetic eukaryotes and might have facilitated the early establishment of plastids.

## Results and discussion

### Chlamydiae-like genes in primary photosynthetic eukaryotes: direction of gene transfer

The nuclear genome of *Cyanidioschyzon *contains 4,771 predicted protein-coding genes [32]. Phylogenomic screen and subsequent phylogenetic analyses identified 16 probable chlamydiae-related genes in *Cyanidioschyzon*, 14 of which were also found in green plants. Five other previously reported genes [13,23] from green plants were also classified as chlamydiae-related after careful re-analyses. The genome sequences of glaucophytes are currently not publicly available, but the gene encoding ATP/ADP translocase is reportedly present in the glaucophyte *Cyanophora paradoxa *and the diatom *Odontella sinensis *[25]. In our search of the Taxonomically Broad EST Database (TBestDB) [33], ATP/ADP translocase homologs were also found in another glaucophyte (*Glaucocystis nostochinearum*), euglenids (*Astasia longa *and *Euglena gracilis*), and a haptophyte (*Pavlova lutheri*). This suggests that chlamydiae-related genes are present in all primary photosynthetic eukaryotic lineages and that the ADP/ATP translocase has been retained in at least some secondary photosynthetic groups (eukaryotic lineages that emerged by engulfing another algal cell as endosymbiont). Therefore, a total of 21 genes from primary photosynthetic eukaryotes are listed here as chlamydiae-related (Table 1). Sequences that are not exclusively related to chlamydial homologs and those that form a monophyletic group with chlamydial homologs but with insufficient bootstrap support (<80%) are not included. These very stringent criteria excluded a large portion (18/37) of previously reported chlamydiae-related plant sequences [13,23].

**Table 1:** Chlamydiae-like genes detected in red algae and green plants

Gene name or gene product	Presence	Putative function
Phosphoglycerate mutase	G	Glycolysis
CMP-KDO synthetase	G	Cell envelope formation
4-diphosphocytidyl-2-C-methyl-D-erythritol kinase (*ispE*)	R and G	Isoprenoid biosynthesis
Polynucleotide phosphorylase	R and G	RNA degradation
Aspartate transaminase	R and G	Amino acid metabolism
Tyrosyl-tRNA synthetase	R and G	Translation
Oligoendopeptidase F	R	Amino acid biosynthesis
2-C-methyl-D-erythritol 4-phosphate cytidylyltransferase (*ispD*)	R and G	Isoprenoid biosynthesis
Enoyl-ACP reductase (*fabI*)	R and G	Fatty acid biosynthesis
23S rRNA (Uracil-5-)-methyltransferase	R and G	RNA modification
Glycerol-3-phosphate acyltransferase	R and G	Phospholipid biosynthesis
ADT/ATP translocase	R and G	ATP/ADP transport
Isoamylase	R and G	Starch biosynthesis
Phosphate transporter	G	Phosphate transport
Hypothetical protein	R	Unknown
β-Ketoacyl-ACP synthase (*fabF*)	R and G	Fatty acid biosynthesis
Malate dehydrogenase	G	Energy conversion
Sodium:hydrogen antiporter	R and G	Ion transport
4-hydroxy-3-methylbut-2-en-1-yl diphosphate synthase (*gcpE*) (GcpE)	R and G	Isoprenoid biosynthesis
Sugar phosphate isomerase	G	Sugar interconversion
Cu-ATPase	R and G	Ion transport

The genome sequences of glaucophytes are not available for comparison, except for the ATP/ADP translocase, which is reportedly present in the glaucophyte *Cyanophora paradoxa *[25] and was also identified in another glaucophyte *Glaucocystis *from the Taxonomically Broad EST Database (TBestDB). G, green plants; R, red algae.

The chlamydiae-like genes identified in this study does not constitute an accurate list of all chlamydiae-related genes in primary photosynthetic eukaryotes, but rather is an estimate from our phylogenomic analyses. This number is probably an underestimate, because the evolutionary origin of many genes is difficult to ascertain using available phylogenetic algorithms, and because some other chlamydiae-like genes may exist in glaucophytes and other red algae but are not retained in the smaller genome of *Cyanidioschyzon*. With the exception of the genes encoding sugar-phosphate isomerase and a hypothetical protein, the protein sequences of all other genes listed in Table 1 contain a plastid-targeting signal as predicted by ChloroP [34] or TargetP [35], or experimentally determined to be plastid localized; this is consistent with the previous report that chlamydiae-related gene products tend to function in plastids in plants [13]. However, the *Arabidopsis *CMP-KDO synthetase homolog (GenBank: NP_175708), although containing a weak plastid-targeting signal (score 0.504 and 0.610 from ChloroP and TargetP, respectively), is believed to be associated with the endomembrane of plant cells [23].

**Figure 1:**
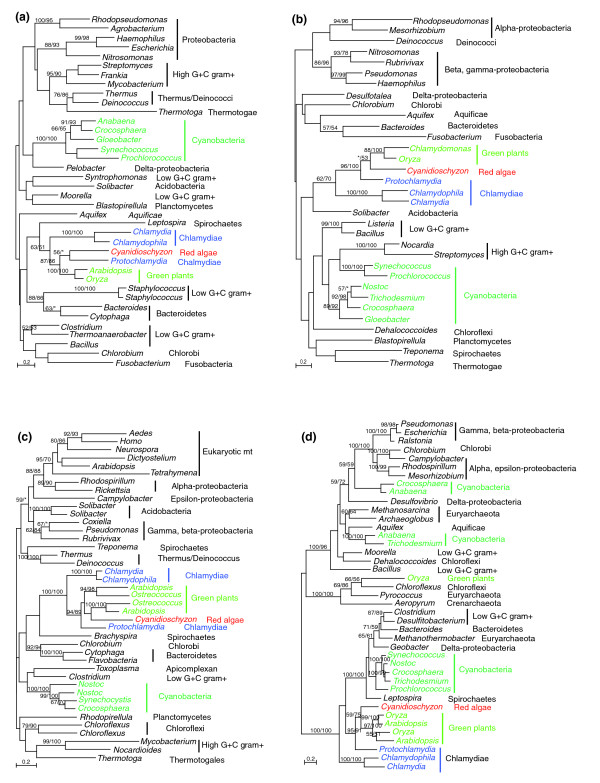
Phylogenetic analyses of chlamydiae-like genes in primary photosynthetic eukaryotes. Numbers above the branch show bootstrap values for maximum likelihood and distance analyses, respectively. Asterisks indicate values lower than 50%. **(a) **2-C-methyl-D-erythritol 4-phosphate cytidylyltransferase (*ispD*). **(b) **4-diphosphocytidyl-2-C-methyl-D-erythritol kinase (*ispE*). **(c) **β-Ketoacyl-ACP synthase (*fabF*). **(d) **Aspartate transaminase. Note that red algal and green plant sequences form a well supported monophyletic group with environmental *Protochlamydia *homologs. mt, mitochondrial precursor. Colors represent different phylogenetic affiliations.

**Figure 2:**
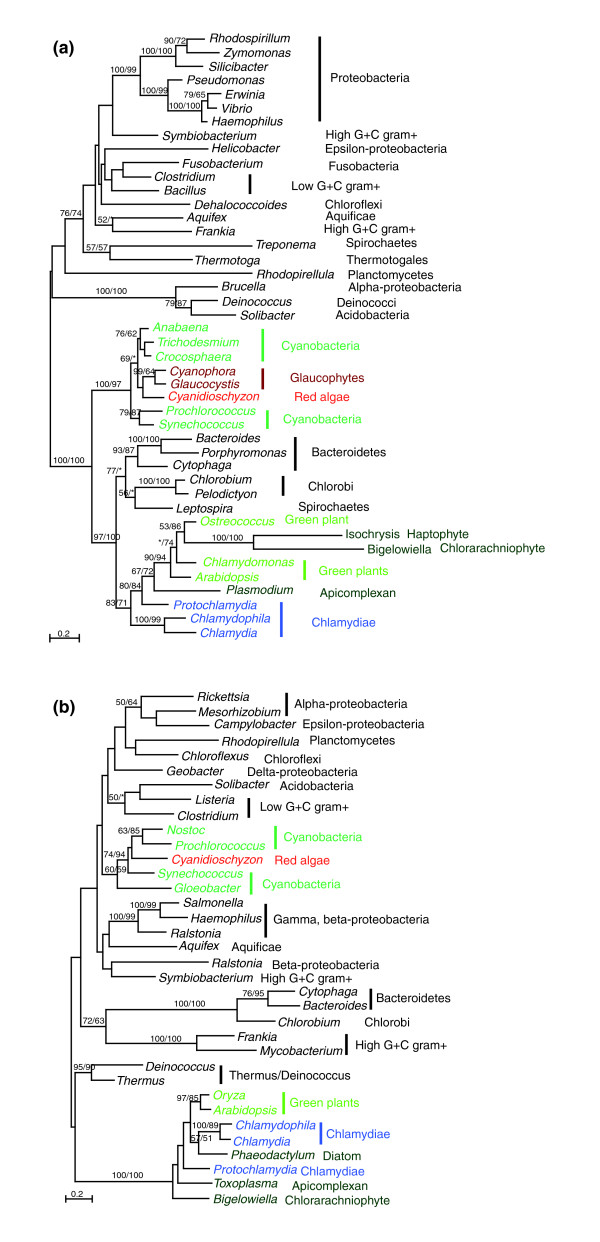
Primary photosynthetic eukaryotes contain gene copies of both plastidic and chlamydial origin. Numbers above the branch show bootstrap values for maximum likelihood and distance analyses, respectively. Asterisks indicate values lower than 50%. **(a) **4-hydroxy-3-methylbut-2-en-1-yl diphosphate synthase (*gcpE*). **(b) **Enoyl-ACP reductase (*fabI)*. Note that in panel (a) sequences from red algae and glaucophytes are of plastidic origin, whereas those from green plants, apicomplexans, haptophytes, and chlorarachniophytes are of chlamydial origin. Also note that that in panel (b) sequences from green plants, diatoms, chlorarachniophytes, and apicomplexans form a strongly supported group, whereas cyanobacterial and red alga *Cyanidioschyzon *homologs form another group. Colors represent different phylogenetic affiliations.

The chlamydial and plant sequence similarities were previously suggested to be an indication of gene transfer from plants or plant-related groups to chlamydiae [21,24,25,28]. However, the genes listed in Table 1 are predominantly distributed in bacteria, indicating a likely bacterial origin (Figures 1 and 2, and Additional data file 1). In all cases, sequences from primary photosynthetic eukaryotes and sometimes from other plastid-containing lineages form a well supported monophyletic group with chlamydial homologs. In most cases they are more closely related to homologs of *Candidatus Protochlamydia amoebophila *UWE25 (a chlamydial species that is found in free-living acanthamoebae and environmental samples and was previously classified as a member of *Parachlamydia *or *Parachlamydia*-related [36]) than to chlamydial sequences as a whole (Figures 1 and 2, and Additional data file 1). However, the sequence relationships among primary photosynthetic eukaryotes vary slightly and differ from the expected organismal relationship, mostly because of insufficient phylogenetic signal as evidenced by low internal bootstrap support, and sometimes because of possible differential gene losses or other evolutionary scenarios (for instance, see Figure 2). These chlamydial and primary photosynthetic eukaryotic sequences also do not appear to be particularly related to homologs from other eukaryotes (Figures 1 and 2, and Additional data file 1).

The bacterial nature of chlamydiae-like genes in primary photosynthetic eukaryotes suggests that they were transferred either from chlamydiae to these eukaryotes or from plastids to chlamydiae. The latter scenario (plastid-to-chlamydiae transfer) implies a plastidic (cyanobacterial) origin for the transferred genes listed (Table 1). For many of the genes, this scenario can be rejected because it does not account for the specific relationship of the chlamydiae-like genes in primary photosynthetic eukaryotes to the *Protochlamydia *homologs (Figure 1a-d, Figure 2a, and Additional data file 1), and because it is incompatible with the cyanobacterial homologs forming a well supported group that is distinct from the chlamydial homologs (Figures 1 and 2, and Additional data file 1). After all, plastids evolved from a cyanobacterial ancestor, and therefore any gene acquired by chlamydiae from plastids should also be more closely related to cyanobacterial than to other bacterial sequences. Additionally, five of the chlamydiae-like genes in photosynthetic eukaryotes lack identifiable cyanobacterial homologs. (The genes encoding ATP/ADP translocase, glycerol-3-phosphate acyltransferase, oligoendopeptidase F, sodium-hydrogen antiporter, and the malate dehydrogenase chloroplast precursors in green plants lack significant hits to cyanobacterial sequences in GenBank searches.) This suggests that the majority of the genes listed in Table 1 were probably transferred from chlamydiae to primary photosynthetic eukaryotes.

### Is there an ancestral relationship between chlamydiae and cyanobacteria?

The relationship between chlamydiae and other bacterial groups remains largely unresolved. Phylogenetic analyses of 16S rRNA suggested that chlamydiae form a sister group with either planctomycetes and verrucomicrobia [14,15,20,37] or cyanobacteria [13,38], without significant support. An ancestral relationship between chlamydiae and cyanobacteria was hypothesized by Brinkman and coworkers [13], largely based on the possession of a predicted plastid-targeting signal in chlamydiae-like plant sequences. Those authors explicitly excluded the possibility of horizontal gene transfer between chlamydiae and their hosts, and assumed that these plant plastid-targeted sequences were of cyanobacterial origin. According to Brinkman and coworkers, these plant sequences are similar to chlamydial homologs because chlamydiae and cyanobacteria (plastids) are evolutionarily related. A few additional characters uniquely shared by cyanobacteria and chlamydiae were identified in the usually structurally conserved ribosomal superoperon [13], including the absence of S10 and S14 genes, which are present in different chromosomal locations in chlamydiae and cyanobacteria. However, a more detailed phylogenetic and comparative study of S14 suggested that this gene was probably independently transferred from α-proteobacteria to cyanobacteria, chlamydiae, and actinomycetes [39]. Therefore, the absence of S14 from the cyanobacterial and chlamydial ribosomal superoperons might be due to relaxed selection to maintain redundant homologs in the genome, rather than an indication of evolutionary relatedness between the two groups.

Although the chlamydiae-cyanobacteria hypothesis offers a popular explanation for the sequence similarity between chlamydiae and plants [20,22,27], it has not been rigorously tested. The major shortcoming of this hypothesis is that all plastids certainly are derived from a past cyanobacterial rather than a chlamydial endosymbiont. Even if chlamydiae and cyanobacteria indeed shared a common ancestry, any sequences of plastidic origin should be more closely related to cyanobacterial than to chlamydial homologs, unless these sequences diverged so rapidly as to generate long-branch attraction artifacts or lateral gene transfer was involved. Based on this reasoning, we have paid particular attention to the relationships among homologous sequences from chlamydiae, cyanobacteria, and primary photosynthetic eukaryotes in our analyses.

All red algal and green plant genes listed in Table 1 clearly are more closely related to chlamydial than to cyanobacterial homologs, with the exception of the genes encoding 4-hydroxy-3-methylbut-2-en-1-yl diphosphate synthase (*gcpE*) and enoyl-ACP reductase (*fabI*), which include distinct gene copies in red algae, glaucophytes, and other plastid-containing eukaryotes (Figure 2 and Additional data file 1). Some of the identified chlamydiae-like genes (for instance, that encoding polyribonucleotide phosphorylase) also contain a number of conserved amino acid residues uniquely shared by chlamydiae, red algae, and green plants. In our molecular phylogenetic analyses of 12 of the genes that contain both cyanobacterial and chlamydial homologs, the cyanobacterial sequences form a clade that is clearly distinct from the chlamydial homologs (Figures 1 and 2, and Additional data file 1). Added to this observation is the fact that several chlamydiae-like genes are not found in cyanobacteria and that the gene encoding glycerol-3-phosphate acyltransferase has identifiable homologs (using *Arabidopsis *[GenBank: NP_849738] and *Protochlamydia *[GenBank: CAF24042] sequences as queries) only in chlamydiae, red algae, green plants, and the apicomplexan *Plasmodium*, which also contains a nonphotosynthetic plastid. The closer relationship between certain chlamydial and plant sequences was also observed in independent studies [20].

In all studies of gene transfer, there are always alternative explanations for each individual gene tree (for example, see the discussion of Figure 2 in section "Further evidence for an ancient chlamydial endosymbiosis with primary photosynthetic eukaryotes") [40]. Overall, however, the pattern from our phylogenetic analyses does not support the hypothesis that cyanobacteria (plastids) and chlamydiae share a close ancestral relationship. This consistent phylogenetic signal from multiple genes should not be dismissed lightly as artifacts of phylogenetic reconstruction, but rather suggests a clear evolutionary link between chlamydiae and primary photosynthetic eukaryotes. Furthermore, given their often specific affinity with environmental *Protochlamydia *homologs (Figures 1 and 2, and Additional data file 1), if all of these primary photosynthetic eukaryotic sequences were indeed of plastidic origin (even though they do not group with cyanobacterial homologs), then this would make chlamydiae a paraphyletic group. Such an observation contradicts the common belief that chlamydiae are monophyletic [14,15] and weighs further against the hypothesis that chlamydiae and cyanobacteria are sister taxa.

### Random horizontal gene transfer versus ancient chlamydial endosymbiosis

Conceivably, if horizontal gene transfer occurred from chlamydiae to the earlier cyanobacterial progenitor of plastids, then chlamydial genes could end up in the nuclear genomes of photosynthetic eukaryotes following subsequent intracellular transfer from plastids to the nucleus. Given the number of chlamydiae-like genes detected in our analyses and the fact that most of the original plastidic (cyanobacterial) genes were lost in modern photosynthetic eukaryotes [41], this scenario entails massive gene transfers from ancient chlamydiae to the cyanobacterial progenitor of plastids. Although gene transfer indeed occurs frequently in prokaryotes [42-44], thus far no chlamydiae-like genes have been reported in any extant cyanobacterium to suggest such massive transfer events.

Because chlamydiae are found in insects, it has also been suggested that plants might have acquired chlamydial genes through insect feeding activities [17]. However, the presence of chlamydiae-like genes in red algae and glaucophytes, which are not a favorable food source for insects, makes this scenario less likely. Furthermore, red algae, glaucophytes, and green plants represent one of the major deep lineages of eukaryotes [45,46]. Such an insect-to-plant transfer scenario would also push the emergence of insects to before the split of primary photosynthetic eukaryotes, which contradicts all available molecular and fossil evidence [47,48].

An unexpectedly high number of chlamydial genes that are most similar to plant homologs has been reported in several independent studies [13,20,21]. For example, most eukaryote-related sequences of *Chlamydia trachomatis *tend to group with plant homologs in phylogenetic analyses [21]. In a similarity-based genome survey, sequences of rickettsia, cyanobacteria, and chlamydiae represented only 14% of the analyzed genes, but they accounted for 65% of bacterial genes that were most similar to eukaryotic homologs; these cyanobacterial and chlamydial sequences disproportionately correspond to plant proteins [13]. Although our focus is to elucidate the cause of chlamydial and plant sequence similarity rather than to reiterate the previous conclusion, our analyses yielded similar findings. For all likely transferred genes in *Cyanidioschyzon *whose origins can be reliably inferred, chlamydiae-like genes (*n *= 16) account for the greatest number from any single group other than cyanobacteria (plastids) and α-proteobacteria (mitochondria), and are followed by five genes from γ-proteobacteria and β-proteobacteria. The latter are also the most represented bacterial groups in GenBank (the taxonomy browser in Entrez of the National Center for Biotechnology Information [NCBI] reported 1,692,357 protein sequences from γ and β-proteobacteria, and only 28,831 from the chlamydiae/verrucomicrobia group as of 4 November 2006). Because of a greater level of stringency, the number of chlamydiae-like genes identified in our phylogenetic analyses is much lower than previously reported (19 versus the 37 reporrted by Brinkman and coworkers [13] in green plants), but this number is still striking, given that using similar methods only a total of 31 genes acquired from all other sources (including those likely from plants and plastids) were identified in the apicomplexan *Cryptosporidium parvum *[49] and about 50 genes in the kinetoplastid *Trypanosoma brucei *[50].

The high number of genes transferred between chlamydiae and photosynthetic eukaryotes is probably due to a more stable association of these two groups in the past. In general, such an association could theoretically occur in the form of symbiosis or physical contact between donor and recipient organisms. However, given the distribution of chlamydiae-like genes mainly in primary photosynthetic eukaryotes and the fact that all extant chlamydial species are obligate endosymbionts, we propose that these genes resulted from an ancient chlamydial endosymbiosis with the ancestor of primary photosynthetic eukaryotes, rather than multiple independent horizontal transfer events or an ancestral relationship between chlamydiae and cyanobacteria. Presumably, the chlamydial symbiotic partner was similar to extant environmental *Protochlamydia*. We use the term 'symbiont' (or 'endosymbiont' in the case of chlamydiae) in the sense of deBary [31] to include mutualistic, parasitic, and commensal associations. Such an ancient endosymbiotic association, similar to those giving rise to mitochondria and plastids, would allow ample time for intracellular (or endosymbiotic) gene transfer from the chlamydial endosymbiont to the nucleus of its eukaryotic hosts (either the ancestor of all primary photosynthetic eukaryotes or individual plastid-containing lineages such as green plants or red algae) and occasionally between the chlamydial endosymbiont and the plastids. Additionally, because the protein products of these intracellularly transferred genes often target to the original organelles, the co-existence of chlamydial and cyanobacterial endosymbionts within the same eukaryotic cell also led to the targeting of chlamydial gene products into plastids and *vice versa*.

### Counting the numbers: can endosymbiosis be inferred from 21 genes?

The residence of plastids and mitochondria within eukaryotic cells led to frequent gene transfers from these organelles to the nucleus [51-53]. Indeed, it has been reported that thousands of genes were transferred from chloroplasts to the nucleus in *Arabidopsis *[53]. Therefore, an apparent question related to our hypothesis is, can we infer the chlamydial endosymbiosis event based on 21 genes?

To answer this question, it should be re-emphasized that all extant chlamydial species are obligate intracellular bacteria. Therefore, if these chlamydiae-like genes in primary photosynthetic eukaryotes resulted from gene transfer from chlamydiae, then they are probably derived from a chlamydial endosymbiont. It should also be noted that although gene transfer from organelles to the nucleus occurs frequently in eukaryotes, the actual scope of transfer might vary among lineages. For example, up to 18% of the nuclear genome was interpreted as derived from plastids in *Arabidopsis*, but a much lower percentage of intracellular gene transfer has been found in the glaucophyte *Cyanophora paradoxa *(9.1%) [54] and in the red alga *Cyanidioschyzon merolae *(Huang and Gogarten, unpublished data). Given the relatively smaller genome of *Cyanidioschyzon *(4,771 predicted protein-coding genes) and a lower level of intracellular gene transfer, it would probably not be possible to identify thousands of genes of any organellar or endosymbiont origin (mitochondrial, plastidic, or chlamydial) in our genome analyses. Most importantly, the retention of transferred genes is often related to the retention and functionality of the organelles in eukaryotic cells [41]. Because the protein products of intracellularly transferred genes often function in the original organelles, loss of certain biochemical functions or even of the organelles themselves will certainly lead to the loss of related transferred genes. For instance, even though thousands of genes were reportedly transferred from chloroplasts in *Arabidopsis *[53], the number of such genes is significantly lower in apicomplexan parasites that harbor a relict, nonphotosynthetic plastid. About 30 genes of plastidic origin (<1% of the nuclear genome) were reported in the human malaria parasite *Plasmodium falciparum *[55] and only two such genes were identified in *Cryptosporidium *that probably lost the plastid entirely [49]. None of these intracellularly transferred genes in apicomplexans are related to photosynthesis. A similar scenario was suggested for *Entamoeba*, which contains a reduced mitochondrion-derived organelle and appears to have lost most mitochondrial pathways [56]. Therefore, the number of chlamydiae-like genes identified in photosynthetic eukaryotes, albeit being a small fraction of the cyanobacterial genes reported in *Arabidopsis *and still lower than reported in some heterotrophic apicomplexans, is many times higher than in *Cryptosporidium*. This lower number of chlamydiae-like genes in primary photosynthetic eukaryotes is in accordance with the seeming absence of chlamydial endosymbionts in modern plastid-containing lineages.

### Further evidence for an ancient chlamydial endosymbiosis with primary photosynthetic eukaryotes

Because chlamydiae are found in diverse eukaryotes such as acanthamoebae and animals [17,57], it is tempting to speculate that the chlamydial endosymbiosis might have existed before the split of the primary photosynthetic eukaryotes from other eukaryotic groups. In this study, we searched the GenBank database, which includes genome sequences of many early-branching eukaryotes, and the TBestDB, which covers diverse groups of protists. Chlamydiae-like genes were found to be restricted mainly to primary photosynthetic eukaryotes and other plastid-containing lineages, supporting specifically an association between chlamydiae and the ancestor of primary photosynthetic eukaryotes. An association with an even earlier eukaryote is not supported.

The hypothesis of an ancient chlamydial endosymbiosis is consistent with the available data. For example, the gene encoding ATP/ADP translocase is a key innovation by obligate intracellular bacteria (chlamydiae and rickettsiae) that live as energy parasites. Instead of making ATP on their own, these bacterial parasites gain ATP from their host cells and transport ADP back for recycling. Aside from these obligate intracellular bacteria, recognizable homologs of the ATP/ADP translocase gene are only found in the microsporidial *Encephalitozoon *(another obligate intracellular parasite) and photosynthetic eukaryotes, where they provide plastids (the original cyanobacterial endosymbiont) with the ATP necessary for starch and fatty acid biosynthesis or as an energy supplement for carbon dioxide fixation [58-60]. The common origin of plastidic and chlamydial ATP/ADP translocases was confirmed by all available phylogenetic analyses [22,25-28], and various evolutionary scenarios have been proposed [13,22,25-28]. However, gene transfer from a chlamydial endosymbiont to its photosynthetic eukaryotic hosts offers a more logical and parsimonious explanation (also see Schmitz-Esser and coworkers [27] for related discussions).

The phylogenies of *gcpE *and *fabI *(Figure 2) also are in agreement with our hypothesis of an ancient chlamydial endosymbiont in the ancestor of primary photosynthetic eukaryotes. Like the genes encoding 2-C-methyl-D-erythritol 4-phosphate cytidylyltransferase (*ispD*) and 4-diphosphocytidyl-2-C-methyl-D-erythritol kinase (*ispE*; Figure 1a,b), *gcpE *is related to isoprenoid biosynthesis (see section "Implications for plastid and eukaryotic evolution" for discussion). The *gcpE *sequences from green plants, apicomplexans, haptophytes, and chlorarachniophytes form a strongly supported monophyletic group with chlamydial (in particular *Protochlamydia*) homologs. On the other hand, the *gcpE *sequences from red algae and glaucophytes form another strongly supported group with cyanobacterial homologs. These two groups are not particularly related (Figure 2a) and it is highly unlikely that the chlamydial *gcpE *gene was acquired from cyanobacteria or plastids. The most plausible explanation for this observation is that two distinct *gcpE *gene copies were originally contributed by chlamydial and cyanobacterial (plastidic) endosymbionts to the nuclear genome of the ancestral primary photosynthetic eukaryote and differentially retained in green plants, red algae, and glaucophytes. The chlorarachniophyte *Bigelowiella *and apicomplexan *Plasmodium*, and the haptophyte *Isochrysis *are believed to contain a green and a red algal endosymbiont, respectively [10,61,62]; their proximity in the same chlamydial group (Figure 2a) probably resulted from independent losses of the plastidic (cyanobacterial) *gcpE *gene copy in these taxa.

*FabI *is another chlamydiae-related gene, aside from the gene encoding β-ketoacyl-ACP synthase (*fabF*; Table 1 and Figure 1c), which is involved in type II fatty acid biosynthesis. Similar to the *gcpE *gene phylogeny, cyanobacterial and red algal *Cyanidioschyzon fabI *sequences form one group, whereas homologs from chlamydiae, green plants, apicomplexans, chlorarachniophytes, and diatoms form another group. These two very distinct sequence groups differ in several highly conserved insertions and deletions, but the relationship between them is less certain because of insufficient internal bootstrap support on the gene tree. Therefore, although it is more likely that the two sequence groups in photosynthetic eukaryotes are derived from cyanobacterial and chlamydial endosymbionts, respectively, it is also theoretically possible that chlamydiae acquired their *fabI *from the plastids of a plant-related group [63]. Because of the distinct sequence difference of the two *fabI *copies, this second scenario entails one of the two following possibilities. The first is the existence of two *fabI *paralogs in the cyanobacterial progenitor of plastids and subsequent independent losses in extant cyanobacteria, and in red algae and other plastid-containing eukaryotes. The second possibility is that independent transfer events occurred from plastids to the nucleus of green plants, apicomplexans, other plastid-containing groups, and the ancestor of extant chlamydiae. In either of the alternative scenarios, a chlamydial endosymbiont and its co-existence with plastids in the same host cell would provide a favorable intracellular environment for transfer between chlamydiae and other organelles.

### Implications for plastid and eukaryotic evolution

Ancient endosymbionts gave rise to organelles, including mitochondria, hydrogenosomes, and plastids [1]. Thus far, no chlamydial endosymbiont has been reported in photosynthetic eukaryotes. Whether a relict organelle derived from the proposed ancient chlamydial endosymbiont exists in extant plastid-containing lineages remains to be further investigated. On the other hand, it also would not be surprising if the chlamydial endosymbiont had degenerated entirely during the evolution of photosynthetic eukaryotes. As obligate intracellular bacterial parasites, chlamydiae depend on their hosts for certain nutrients, and consequently they have a relatively reduced genome [20,21]. Many dispensable genes were probably lost as a result of their parasitic lifestyle. Given the lack of apparent benefit to the host cell, such gene losses in an isolated intracellular system could gradually lead to deterioration of the endosymbiont genome and ultimately the endosymbiont itself [64].

Almost all chlamydiae-like genes identified in red algae and green plants (Table 1) contain a predicted plastid-targeting signal, although the example of CMP-KDO synthetase suggests that this prediction may not always be reliable. The chlamydiae-like genes are involved in a variety of biochemical activities in plastids, including fatty acid biosynthesis, ion transport, nitrogen metabolism, and RNA processing, among others. Notably, three of these genes (*ispD*, *ispE*, and *gcpE*) are key enzymes of the deoxyxylulose 5-phosphate (DXP) pathway, which leads to the formation of isopentenyl diphosphate, a major metabolite for isoprenoid biosynthesis in bacteria and plastids [65-67]. The chlamydial origin of *ispD *and *ispE *was also confirmed by independent studies [68], which hypothesized that the DXP pathway in primary photosynthetic eukaryotes was probably derived from plastids. In our phylogenetic analyses of *ispD *and *ispE*, the chlamydial and primary photosynthetic eukaryotic sequences form a monophyletic group that is distinct from the cyanobacterial homologs (Figure 1a,b), suggesting probable gene displacement after the endosymbiotic origin of plastids.

Secondary endosymbioses between photoautotrophic algae and heterotrophic host cells occurred several times during eukaryotic evolution [9-12]. In contrast, the formation of the primary cyanobacterial endosymbiosis appears to have been unique (but also see the report by Marin and coworkers [69]). Our finding of the probable participation of a third symbiotic partner offers an explanation for this rarity. As free-living photoautotrophic cells, in which ATP is generated in the main cytoplasmic compartment, cyanobacteria do not need mechanisms to transport energy-rich metabolites between membrane-enclosed compartments. However, for an enslaved cyanobacterium in a heterotrophic host to transform into a photosynthetic organelle, new transport systems are necessary. Therefore, the initial adaptation of a photoautotrophic cyanobacterium toward a photosynthetic organelle was probably a difficult process contingent on the simultaneous presence of suitable transport systems. At least in part these transporters might have evolved in a chlamydial parasite that was present within the same eukaryotic host cell. These transporters enabled chlamydiae to parasitize energy and other molecules from the host cell, but also allowed for ATP/ADP equilibration with the cyanobacterium.

We suggest that three organisms were involved in establishing the primary photosynthetic lineage: the eukaryotic host cell, the cyanobacterial endosymbiont that provided photosynthetic capability, and a chlamydial endosymbiont or parasite that facilitated the establishment of the cyanobacterial endosymbiont. The coexistence of three partners with different biological requirements and capabilities might have offered an opportunity for some transient mutualistic interactions, and the acquisition of genes such as those encoding ATP/ADP translocase and sodium:hydrogen antiporter from the chlamydial endosymbiont might have facilitated the successful endosymbiosis of cyanobacteria by allowing energy flux into the protoplastid organelle and effective regulation of ion composition. Specifically, we hypothesize that the origin and the establishment of primary plastids might have involved the following stages.

**Figure 3:**
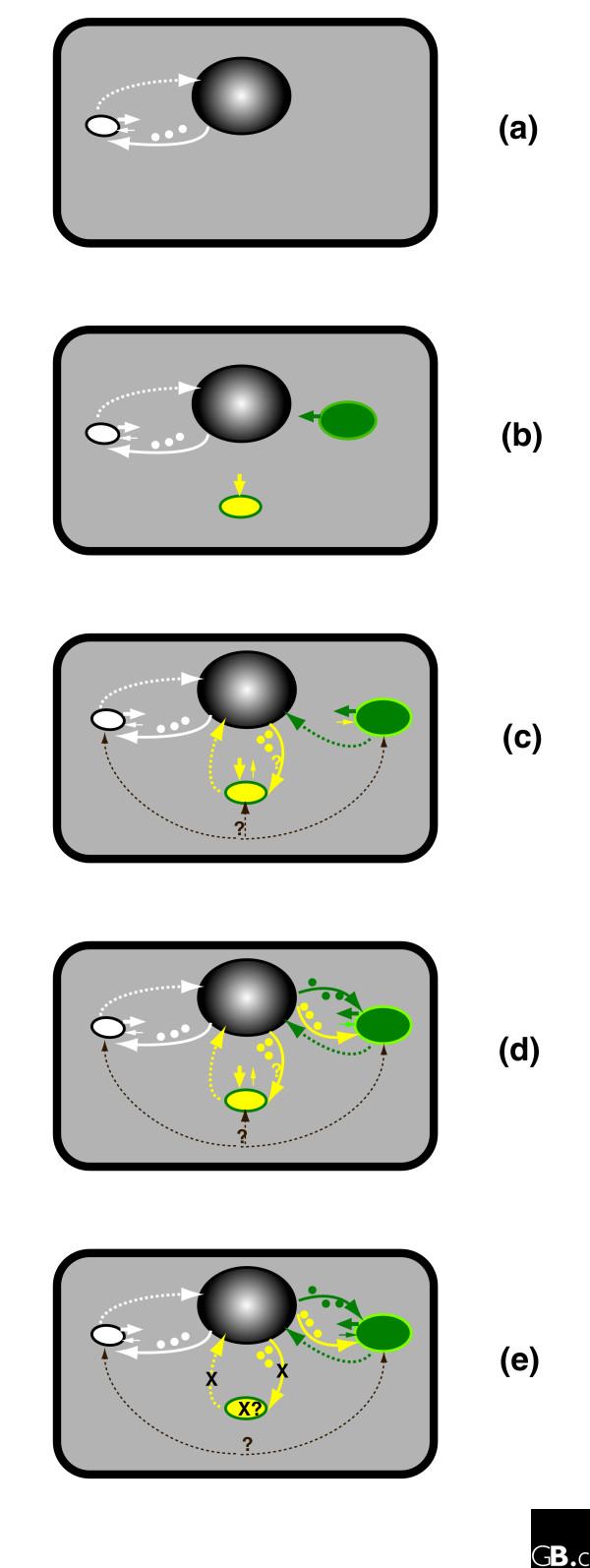
Hypothetic stages of plastid origin and establishment. The stages (as discussed in the text) are displayed as follows: **(a,b) **first stage; **(c) **second stage; **(d) **third stage; and **(e) **fourth stage. White, yellow, and green colors show α-proteobacterial (mitochondrial), chlamydial, and cyanobacterial endosymbionts, as well as genes and proteins of their respective origins. Arrows directly from the endosymbiont point to the symbiotic partner that receives the benefit, and the thickness of the arrow indicates the degree of benefit. Dashed lines indicate directions of intracellular gene transfer, whereas solid lines show protein targeting of the transferred genes. Crosses indicate chlamydial endosymbiont and gene transfer processes that might not exist in extant photosynthetic eukaryotes. Note that chlamydial endosymbiont was initially a bacterial parasite in the first stage, but it had a transient mutualistic relationship with the host cell in the second and third stages, and then might have degenerated in modern photosynthetic eukaryotes. Note also that the cyanobacterial endosymbiont was initially captured to solely benefit the host cell (panel b), and then received metabolites from the host cell (a process facilitated by the chlamydial endosymbiont) and was gradually transformed into a plastid organelle in the host cell (panels d and e).

In the first stage, a chlamydial bacterium, similar to the extant *Protochlamydia*, entered a mitochondrion-containing eukaryote as a bacterial parasite. This chlamydial endosymbiont possessed a necessary transport system to gain nutrients and other metabolites from the host cell. At about the same time, a once free-living photoautotrophic cyanobacterium was captured by the eukaryotic host by chance, initially possibly as a food source (Figure 3a,b).

In the second stage, gene transfer between the chlamydial endosymbiont and the host cell ensued because of their physical association. As a result, the eukaryotic host acquired transporters from the chlamydial endosymbiont, facilitating its communication with the cyanobacterial captive. At this stage, the relationship between chlamydial endosymbiont and the host cell might be considered transiently mutualistic. Gene transfer between the cyanobacterial captive and the host cell might also have occurred during this stage (Figure 3c).

In the third stage, the cyanobacterial captive was gradually transformed into a photosynthetic organelle (plastid) in the host cell and a stable, mutualistic relationship between the plastid and the host cell was in place. The plastid provided photosynthetic products to the host, whereas the host offered shelter and also transported protein products of the intracellularly transferred genes (both from the chlamydial endosymbiont and the cyanobacterial captive) and other necessary metabolites to the plastid organelle (Figure 3d).

In the fourth and final stage, once the plastid organelle was fully established in the host cell, the benefits of the chlamydial endosymbiont to the host became less apparent. It is possible that the chlamydial endosymbiont remained in the host cell mostly as a bacterial parasite. Such a parasitic relationship might not be sustained over a long period of time and the chlamydial endosymbiont might have gradually degenerated. It is also possible that the chlamydial endosymbiont was transformed into an organelle yet to be recognized in photosynthetic eukaryotes (Figure 3e).

Once a rich repertoire of transporters were in place in primary photosynthetic eukaryotes, transport of photosynthetic products and other metabolites across the photosynthetic organelle might be more easily adapted to the different membranes in secondary or tertiary endosymbiotic hosts [70]. This also explains the observation that secondary endosymbiosis is more frequent than primary endosymbiosis in eukaryotic evolution.

The likely ancient chlamydial endosymbiosis with primary photosynthetic eukaryotes has some other important implications for eukaryotic evolution. Ancient endosymbiosis events, such as those that gave rise to mitochondria and plastids, to a large degree defined the evolution of eukaryotes. Our data suggest that these ancient endosymbiosis events might have occurred more frequently, and some of them might have been contingent on others. Such ancient endosymbioses and subsequent intracellular gene transfers contributed to the evolution of host organisms and their descendent lineages, regardless of whether an organelle derived from past endosymbionts is retained in extant species.

The finding of this study also weighs into the relationship of primary photosynthetic eukaryotes. Although a common origin of these groups and of their plastids has been supported by many studies [29,30], several other analyses, particularly those of nuclear genes, have provided ambiguous or conflicting results [71,72]. The ancient chlamydial endosymbiosis at the root of the primary photosynthetic lineages provides strong and independent evidence for a common origin of all primary photosynthetic eukaryotes and of the plastids they harbor.

## Conclusion

The availability of a complete genome sequence of the red alga *Cyanidioschyzon *and expressed sequence tag (EST) data for diverse deep eukaryotes allows a more detailed study of the distribution and evolution of chlamydiae-like genes in primary photosynthetic eukaryotes. Our very stringent phylogenomic analyses indicate that these chlamydiae-like genes are unlikely to have derived from independent horizontal gene transfer events or evolutionary relatedness between chlamydiae and cyanobacteria. The chlamydiae-like genes in photosynthetic eukaryotes probably resulted from an ancient endosymbiosis event between chlamydiae and the ancestor of primary photosynthetic eukaryotes, with the chlamydial partner being similar to extant environmental *Protochlamydia*. This ancient chlamydial endosymbiosis with primary photosynthetic eukaryotes might also have played a role in the establishment of plastids by providing genes that possess new functions and by allowing effective communications between the cyanobacterial endosymbiont and the eukaryotic host cell.

## Materials and methods

### Data sources

Protein sequences for the red alga *Cyanidioschyzon merolae *were obtained from the *Cyanidioschyzon *Genome Project [73]. The predicted protein sequences for the green alga *Chlamydomonas reinhardtii *were acquired from *Chlamydomonas *genome sequencing project at the Joint Genome Institute [74]. EST sequences of several protists were obtained from TBestDB [33] and all other sequences were from the NCBI GenBank protein sequence database.

### Phylogenomic analyses

Phylogenomic analyses of *Cyanidioschyzon*, which is the only red algal species whose complete genome sequence is yet available, were performed using PhyloGenie [75] and the NCBI nonredundant protein sequence database to identify chlamydiae-related genes. The results were compared with those of previously published analyses and further detailed phylogenetic analyses were performed. For detailed phylogenetic analyses, sequences were selected from major groups within each domain of life. Multiple protein sequence alignments were performed using MUSCLE [76] and clustalx [77], followed by cross-comparisons and manual refinement. Only unambiguously aligned sequence portions were used. The alignments are detailed in Additional data file 1. Phylogenetic analyses were performed with a maximum likelihood method using PHYML [78] and a distance method using the program neighbor of PHYLIP version 3.65 [79], with maximum likelihood distances calculated using TREE-PUZZLE [80]. Branch lengths and topologies of the trees depicted in all figures (Figures 1 and 2, and Additional data file 1) were calculated with PHYML [78]. All maximum likelihood calculations were based on the JTT substitution matrix and a mixed model of four gamma-distributed rate classes plus invariable sites. Maximum likelihood distances for bootstrap analyses were calculated using TREE-PUZZLE [80] and PUZZLEBOOT v1.03 (ME Holder and AJ Roger; available on the web [81]). The plastid-targeting signal of identified chlamydiae-like protein sequences was predicted using the web-based ChloroP [34] and TargetP [35].

## Additional data files

The following additional data are available with the online version of this paper. Additional data file 1 contains protein sequence alignments used for phylogenetic analyses and resulting phylogenetic trees.

## Supplementary Material

Additional File 1 The document contains protein sequence alignments used for phylogenetic analyses and resulting phylogenetic trees. Each sequence name includes a GenBank GI number followed by the species name Click here for file
