# Supplementary material for: Did an ancient chlamydial endosymbiosis facilitate the establishment of primary plastids?
Source: Genome Biol. 2007 Jun 4;8(6):R99. doi: 10.1186/gb-2007-8-6-r99 (PMC2394758; doi:10.1186/gb-2007-8-6-r99)
Supplement: Additional File 1 — The document contains protein sequence alignments used for phylogenetic analyses and resulting phylogenetic trees. Each sequence name includes a GenBank GI number followed by the species name [file gb-2007-8-6-r99-S1.pdf]

Did an ancient chlamydial endosymbiosis facilitate the establishment of primary plastids?

Jinling Huang   Johann Peter Gogarten

1. Materials and Methods

Data sources: Protein sequences for the red alga *Cyanidioschyzon merolae* were from *Cyanidioschyzon merolae* genome database [73]. The *Chlamydomonas* predicted protein sequences were obtained from green alga *Chlamydomonas* genome sequencing project [74]. Several EST sequences were obtained from the Taxonomically Broad EST Database [33] and all other sequences were obtained from the NCBI databases

Phylogenetic analyses: Sequences were selected for major groups within each domain of life. Phylogenetic analyses were performed with a maximum likelihood method using PHYML [78] and a distance method using the program *neighbor* of PHYLIP version 3.65 [79] with maximum likelihood distances. All maximum likelihood calculations were based on the JTT substitution matrix and a mixed model of 4 gamma-distributed rate classes plus invariable sites. Maximum likelihood distances for bootstrap analysis were calculated using TREE-PUZZLE [80] and PUZZLEBOOT v1.03 (by Michael E. Holder and Andrew J. Roger, available from [81]).

2. Protein sequence alignments used for phylogenetic analyses and resulting phylogenetic trees. Each sequence name includes GI number from GenBank followed by species name. Numbers above the branches of the gene tree show bootstrap support values for maximum-likelihood analyses and distance analyses, respectively. Asterisks indicate support values below 50%.

1. Phosphoglycerate mutase

```
CLUSTAL X (1.83.1) multiple sequence alignment


15605455_Chlamydia_tracho       LLILLRHGQSVWNQKNLFTGWVDIPLSQQGIQEAIAAGESIKPIDCIFTS
15836396_Chlamydophila_pneumo   LLILLRHGQSVWNEKNLFSGWVDIPLSQQGIEEAFSAGRAIQPIDCIFTS
50912843_Oryza_sativa           ALILIRHGESLWNEKNLFTGCVDVPLTPKGVDEAIEAGKRICPVDVIYTS
42563306_Arabidopsis_thalia     ALILIRHGESLWNEKNLFTGCVDVPLTQKGVGEAIEAGKKISPVDLIFTS
46399436_Protochlamydia_amoebo  KLILMRHGQSQWNLANLFTGWVDIPLSFKGIEEAIEAGKQIKPIDLIFTS
27375797_Bradyrhizobium_japoni  LLVLVRHGQSEWNLKNLFTGWKDPDLTEQGVAEAREAGRKLKVFDVAYTS
13473897_Mesorhizobium_loti     TLVLVRHGQSEWNLKNLFTGWRDVDLTEQGHAEAKAAGQKLKKFDIAFTS
71022513_Ustilago_maydis        TLVLTRHGQSEWNKLNLFTGWKDPALTELGREEALTGAKRVAKFDVAFTS
15677454_Neisseria_mening       ELVFIRHGQSEWNAKNLFTGWRDVKLSEQGLAEAAAAGKKLKEFDIAFTS
16272698_Haemophilus_influe     ELVFIRHGFSEWNAKNLFTGWRDVNLTERGVEEAKTAGKKLLEFDIAFTS
16801372_Listeria_innocu        KLVLIRHGQSEWNKLNLFTGWHDVDLSEEGVVEAMTAGKRIKEFDVAFTS
71367566_Nocardioides_sp.       TLILLRHGESEWNAKNLFTGWVDVNLTEKGRAEAVRGGELMRLPDVVHTS
68231626_Frankia_sp.            TLVLLRHGESTWNRENLFTGWVDVDLSEKGLKEATRGGELLQLPDVVHTS
71274132_Homo_sapien            KLVLIRHGESTWNLENRFSCWYDADLSPAGHEEAKRGGQALREFDICLTS
Acanthmoeba_pgm                 KVVLIRHGESTWNKENRFTGWTDVDLSDKGIEEALTAAKALKTFDVAYTS
Streblomastix_pgm               ----------------------DCDLSAKGVEEAKSAGVILKDFDIAFTS
23172543_Drosophila_melano      KIVMVRHGESEWNQKNQFCGWYDANLSEKGQEEALAAGKAVKEFDVAHTS
68002649_Geobacter_metall       KLVLLRHGESVWNRENRFTGWTDVGLSEKGIEEAISAGRTLKVFDVAYTS
```

```
67934469_Solibacter_usitat                 KLVLIRHGESTWNKENRFTGWTDVDLSDEGRQQATEAAEVLKVFDVAYTS
21673238_Chlorobium_tepidu                 KLVLLRHGESQWNRENRFTGWVDVDLSEKGREEARTAGQLLKVFDLAYTS
73540010_Ralstonia_eutrop                  KLVLIRHGESTWNLENRFTGWVDVDLTDTGAAQARLAGKLLKAFDVAYTS
49479247_Bacillus_thurin                   KLVLIRHGQSLWNLENRFTGWTDVDLSENGLSEAREAGAILKTFDVAYTS
66809133_Dictyostelium_discoi              KLVLIRHGESTWNKENKFTGWTDVDLSEKGVQEAHEAGKRLLTFDIAYTS
23508399_Plasmodium_falcip                 TLVLLRHGESTWNKENKFTGWTDVPLSEKGEEEAIAAGKYLKKFDVVYTS
34763468_Fusobacterium_nuclea              KLVLIRHGESAWNLENRFTGWKDVDLSPKGVEEAKAAGKSLKVFDVAYTS
34539994_Porphyromonas_gingiv              RIVLIRHGESLWNKENRFTGWTDVDLSEKGIEEAKKAGELMKQFTKAYTS
42527203_Treponema_dentic                  RLVLVRHGESEWNKLNLFTGWTDVDLSEKGVEEAKEGGTYLKDFDICYTS
26246721_Escherichia_coli                  KLVLVRHGESQWNKENRFTGWYDVDLSEKGVSEAKAAGKLLKSFDFAYTS
49484632_Staphylococcus_aureus             KLILCRHGQSEWNAKNLFTGWEDVNLSEQGINEATRAGEKVRDIDVAFTS
48855514_Cytophaga_hutchi                  ILVIVRHGQSEWNLKNLFTGWSDVELTPTGEHEAEYAGIVLRHFDIAFTS
37521165_Gloeobacter_violac                HLILIRHGQSLWNAANKFTGWVDVPLSERGRAEATIASCKLRRVNVCFTS
76793350_Pseudoalteromonas_atl             LLTLIRHGQSIWNQQNRFTGWVDVSLSQSGVKEAQRAAQMLSRFDLAFTS
20091231_Methanosarcina_acetiv             YLIIVRHGESGWNVDGRFGGWVDVPLTGKGIKEALLCAAELEDLDVTFTS
75909856_Anabaena_variab                   RLLLVRHGETEWNRQTRFQGQIDVPLNDNGRQQAQKAGVFLQAIDFAVSS
23128494_Nostoc_puncti                     RLLLVRHGETEWNRQTRFQGQIDVPLNDNGRQQSQKAGEFLQAIDFAVSS
56751043_Synechococcus_elonga              RLLLVRHGETDWNRQKRFQGQIDIPLNDNGRAQARSAAEFLAQIDFAVSS
37520340_Gloeobacter_violac                RLLLVRHGETEWNRMERFQGQIDVPLNDQGRAQAEQAATFLKPITRAFSS
33239966_Prochlorococcus_marin             RIFLVRHGETNWNKEGRFQGQIDIPLNENGQKQALAASNFLKKFNQAFSS
76259753_Chloroflexus_aurant               RLIIVRHGESEWNRINRYQGQQDAPLSELGRKQAAALGERLRKIDVVYSS
4981935_Thermotoga_mariti                  KLYLIRHGETIWNEKGLWQGVTDVPLNERGREQARKLANSLKRVDAIYSS
15805309_Deinococcus_radiod                -LLLVRHGATAWNEGGQWQGWTDNPLGDAGRAQARALREELATFDAVYSS
73540185_Ralstonia_eutrop                  HLIVIRHGETAWNRERRLQGQLDIPLNETGEAQARALAAALAPIDAVYSS
16799370_Listeria_innocu                   TIYFVRHGKTEWNMTGQMQGWGDSPLVAEGIDGAKAVGEVLKPIDAVYTS
75908396_Anabaena_variab                   NLYLLRHGETTFSQSGNFCGKTDADLTSEGVQMAESFAKVYQKWEAVYVS
                                                                     *   *    *   :                  *

15605455_Chlamydia_tracho                  TLVRSLITALLAMTNHSSQKQSSALNERMYGELQGKNKQEVAAQFGEEQV
15836396_Chlamydophila_pneumo              TLVRSLMTALLAMTNHHSKKQSSALNERMYGELQGKNKKQTAEQFGEERV
50912843_Oryza_sativa                      SLIRAQMTAMLAMMQHRRKKTAWQLNERMYGELQGLNKQETADRFGKEQV
42563306_Arabidopsis_thalia                SLIRAQMTAMLAMTQHRRKKAAWQLNERMYGELQGLNKKETAERYGTQQV
46399436_Protochlamydia_amoebo             SLIRAQMTAMLAMSVHTSGKRAWELNERMYGELQGINKEEMAKKYGAEQV
27375797_Bradyrhizobium_japoni             VLTRAQHTLDLILGELGQKGKNLALNERDYGDLSGLNKDDARKKWGEDQV
13473897_Mesorhizobium_loti                ALSRAQKTCQHILDAVGQSDRDQALNERDYGDLSGLNKDDARKKWGEEQV
71022513_Ustilago_maydis                   ALQRAQTTLGIQLKEIGQEDKDQALNERDYGELSGLNKDDARKKWGEDQV
15677454_Neisseria_mening                  VLTRAIKTCNIVLEESDQLFKTWRLNERHYGQLQGLDKKQTAEQYGDEQV
16272698_Haemophilus_influe                VLTRAIKTCNIVLEESHQLWKNWRLNERHYGALQGLDKKATAEQYGDEQV
16801372_Listeria_innocu                   VLTRAIKTLNYVLEESDQMWKSWRLNERHYGALQGLNKQETAEKYGADQV
71367566_Nocardioides_sp.                  VQRRAINTACLALDAADRHWRSWRLNERHYGALQGKNKKETLEAYGEEQF
68231626_Frankia_sp.                       LLTRAIRTAWLALDAAGRTWRSWRLNERHYGGLQGLNKAETLAKFGDEQF
71274132_Homo_sapien                       VQKRVIRTLWTVLDAIDQMWRTWRLNERHYGGLTGLNKAETAAKHGEAQV
Acanthmoeba_pgm                            VLKRAIRTLWLVLNELDLNWKSWRLNERHYGALQGLDKAETAAKHGEDQV
Streblomastix_pgm                          VLRRAIRTLWLTLDGLELIHRHWRLNERHYGGLQGLNKAETAEKYGIEQV
23172543_Drosophila_melano                 VLTRAQVTLASILKASGHKEKTWRLNERHYGGLTGLNKAETAAKYGEAQV
68002649_Geobacter_metall                  VLKRAIKTLWLVLEEMDLMWRHWRLNERHYGALQGLNKAETAERHGMEQV
67934469_Solibacter_usitat                 VLKRAIRTLWTVLDGMDLMWRSWRLNERHYGALQGLNKAETAAKFGEDQV
21673238_Chlorobium_tepidu                 VLKRAIRTLWTVLDEMNLMWKNWRLNERHYGALQGLNKAETAQRHGDEQV
73540010_Ralstonia_eutrop                  VLKRAIRTLWHVQDEMDEMWNEWRLNERHYGALAGLNKAETAAKFGDEQV
49479247_Bacillus_thurin                   VLKRAIRTLWIVLHEMNLAWKSWKLNERHYGALQGLNKDETAQKYGEEQV
66809133_Dictyostelium_discoi              VLKRAIRTLWILLEELNLYWRQWRLNERMYGSLQGLNKSETAAKYGEDQV
23508399_Plasmodium_falcip                 VLKRAICTAWNVLKTADLLHKTWRLNERHYGSLQGLNKSETAKKYGEEQV
34763468_Fusobacterium_nuclea              YLKRAIKTLNIVLEEMDELYKSWRLNERHYGALQGLNKAETAKKYGDEQV
34539994_Porphyromonas_gingiv              YLKRAVKTLNGVLDVMDLDWKTWRLNEKHYGMLQGLNKAETAEKYGDEQV
42527203_Treponema_dentic                  YLKRAIHTLNYILSQMDREWKTWKLNERHYGGLQGLNKAETAEKYGEDQV
26246721_Escherichia_coli                  VLKRAIHTLWNVLDELDQAWKSWKLNERHYGALQGLNKAETAEKYGDEQV
49484632_Staphylococcus_aureus             LLTRALDTTHYILTESKQQWKSWRLNERHYGGLQGLNKDDARKEFGEEQV
48855514_Cytophaga_hutchi                  VLKRAIHTLSIILNQTG-ATENEALNERNYGDLQGLNKAEVGEKYGAQQL
37521165_Gloeobacter_violac                MLMRAIETAVITLTECDDICPTATLDERYYGDLQGLDKAETTAKYGKEQV
76793350_Pseudoalteromonas_atl             ELLRAQDTLYEILRHNRQCHVSQQLNERYYGDLQGLNKDKARQLFGDEQV
20091231_Methanosarcina_acetiv             KLIRAQETLFLILSKQKKIGSNEALNERYYGILQGKKKDKMKAKYGEEQI
75909856_Anabaena_variab                   SMLRPKETAEIILRHHPSINLQDGLREISHGLWEGKLEAEIEEEFPGELE
23128494_Nostoc_puncti                     TMLRPKETAEIILKQHPNVKLQDGLREISHGLWEGKLETEIEQEFPGELQ
56751043_Synechococcus_elonga              PMARPKETAELILERHPNCEVDDRLQEIGHGLWEGKLEEEIAAEFGELLQ
37520340_Gloeobacter_violac                PLLRPKATAEAILRFHPEVAFVPALQEICHGQWEGKFRAEIDLLFPGELE
```

```
33239966_Prochlorococcus_marin      SMSRPMETAKIILRNHPTIEQQDELVEIGHGLWEGKLEAEISSEWGDLLK
76259753_Chloroflexus_aurant        RLQRAAHTAQAIVAHHPGLEYDDALLEINHGEWEGKYLHEILERYADGLR
4981935_Thermotoga_mariti           PLKRSLETAEEIAR-RFEKEVEEDLRECEISLWNGLTVEEAIREYPVEFK
15805309_Deinococcus_radiod         DLTRARQTAELALP--GRALLDARLRELNLGDYEGHTLAQMQAHG--GYA
73540185_Ralstonia_eutrop           DLGRAMQTAAPLAETLGLKVSEPRLRERSYGTLQGMTYAEVAEKLPEDFA
16799370_Listeria_innocu            TSKRTQDTAAYILGDREIEIPLEELKEMSFGTWEGIRVTEIDEQHPEERA
75908396_Anabaena_variab            PMKRTIATAKPFCDAIGMEMLRDGLREGSYGEWETKSKTFAQENYSENYV
                                       *   *                * *   .

15605455_Chlamydia_tracho           KLWRRSYRIAPPQGESLFDTGQRTLPYFQERIFPLLQQGIFISAHGNSLR
15836396_Chlamydophila_pneumo       KLWRRSYKTAPPQGESLYDTKQRTLPYFEKNILPQLQHGVFVSAHGNSLR
50912843_Oryza_sativa               HEWRRSYDIPPPNGESLEMCAERAVAYFKDQIVPQLVAGVMIAAHGNSLR
42563306_Arabidopsis_thalia         HEWRRSYEIPPPKGESLEMCAERAVAYFEDNIKPELASGVMIAAHGNSLR
46399436_Protochlamydia_amoebo      HIWRRSFDVPPPNGESLQMTAARTIPYFENTIVPHLKEKIFIAAHGNSLR
27375797_Bradyrhizobium_japoni      LIWRRSYDVPPPGGESLKDTLARALPYYVQEILPSVLNGTLVAAHGNSLR
13473897_Mesorhizobium_loti         HVWRRSYDVSPPGGESLKDTGARVWPYYLHDLQPHVLRGVLVAAHGNSLR
71022513_Ustilago_maydis            HIWRRSYDIPPPGGESLKLTAERVWPYYHKEILPLVKQGVIVAAHGNSLR
15677454_Neisseria_mening           RIWRRSYDTLPPDGENLKVTLERVLPFWEDQIAPAILSGVLVAAHGNSLR
16272698_Haemophilus_influe         HIWRRSYDISPPNAENLKLTLERALPFWEDQIAPAMLSGVLVVAHGNSLR
16801372_Listeria_innocu            QKWRRSYDTLPPSGENLKVTLERVIPYWMDTIAPEIKAGVVIAAHGNSLR
71367566_Nocardioides_sp.           MLWRRSFDVPPPHTECLKDVIARFLPYWESDIVPDLRAGVLIAAHGNSLR
68231626_Frankia_sp.                LLWRRSYDTPPPLTECLRDVVVRMLPYWYDSIVPDLRSGVLVTAHGNSLR
71274132_Homo_sapien                KIWRRSYDVPPPSYESPKDTIARALPFWNEEIVPQIKEGVLIAAHGNSLQ
Acanthmoeba_pgm                     KIWRRAYAIPPPKTESLADTVERFLPYWHDTIAPAIKSGVIVAAHGNSLR
Streblomastix_pgm                   TQWRRAFAIPPPAHESLADTIVRVMPFWHDQIVPAIRSGVLIAAHGNSLR
23172543_Drosophila_melano          QIWRRSFDTPPPQFESLKLTIERTLPYWNDVIIPQMKEGILIAAHGNSLR
68002649_Geobacter_metall           MIWRRSYDIPPPLTESLKDTVARFLPYWHETIAPAVKEGVLVTAHGNSLR
67934469_Solibacter_usitat          KIWRRSYDIPPPLTECLKDTVARFLPLWHDTIAPAIRSGVIIAAHGNSLR
21673238_Chlorobium_tepidu          LIWRRSYDTPPPATECLKDTVARFLPYWHETIAPQIRDGVIITAHGNSLR
73540010_Ralstonia_eutrop           LVWRRSYDTPPPLTECLKDTVARVLPLWNESIAPDIQSGVVIAAHGNSIR
49479247_Bacillus_thurin            HIWRRSIDVRPPLTECLVDTEKRVLAYWHSEIAPKLKDGVIISSHGNTIR
66809133_Dictyostelium_discoi       LIWRRSYDIPPPKTECLKDTVERFLPLWNDTIAPTIKSGVLIAAHGNSIR
23508399_Plasmodium_falcip          KIWRRSYDIPPPFTECLKDTVERVLPFWFDHIAPDILANVMVAAHGNSLR
34763468_Fusobacterium_nuclea       HIWRRSFDVAPPLGESLKDTIARVLPYWHSDISKSLQEGVIVAAHGNSLR
34539994_Porphyromonas_gingiv       LIWRRSYDVPPPLTEALCDTVNRILPYWNETIFPTLKEHVLVAAHGNSLR
42527203_Treponema_dentic           KIWRRSFDIAPPLTESLKDTIARAVPFFEKTIKPQMLEGILITAHGNSLR
26246721_Escherichia_coli           KQWRRGFAVTPPLTESLALTIDRVIPYWNETILPRMKSGVIIAAHGNSLR
49484632_Staphylococcus_aureus      HIWRRSYDVKPPYSESLKDTLVRVIPFWTDHISQYLLDGVLVSAHGNSIR
48855514_Cytophaga_hutchi           IAWRRSYTEVPPGGESLENTCQRVIPYYQEKIEPELKDGVLIVAHGNSLR
37521165_Gloeobacter_violac         QIWRRSYSVRPPGGESLEDTRKRVYPYFTNRILGHIKQGVLVAAHGNSLR
76793350_Pseudoalteromonas_atl      HTWRRSYNVAPPNGESLAMTATRAIAYFQSHIVPALQQGVLVCAHGNSLR
20091231_Methanosarcina_acetiv      LHWCRSFDEGPPEGESLKDIYRRAVPYFEKEIFPILQDGVIVCAHQNSLR
75909856_Anabaena_variab            RWRTIPGQVQMPEGENLQQVWERSTEAWQNIVQTALDNQGLIVAHDATNK
23128494_Nostoc_puncti              RWRLVPAQVQMPEGENLQEVWERSVAAWQSIVEAALTNQVLVVAHDATNK
56751043_Synechococcus_elonga       LWKDQPEQVQMPEGENLQEVWDRSVAAWEAIVANAPEGSGLVVAHDAVNK
37520340_Gloeobacter_violac         RWQREPHSVQMPEGENLTQVWERATDAWSGIVR-GVDSGVLVVAHDAINK
33239966_Prochlorococcus_marin      RWKKSPETVQMPEGETIGDVSSRAMNCFRHIAQRLSPNDALIVAHDAVNK
76259753_Chloroflexus_aurant        EWRQHPTRSQMPGGESFSNVLKRVLDFRERICVQHAGQTVLISTHDVIVK
4981935_Thermotoga_mariti           KWSSDP-NFGMEGLESMRNVQNRVVKAIMKIVSQEKLNGVVIVSHSLSLR
15805309_Deinococcus_radiod         GWQADPWAQAVPGGESLQDVAARMRGWLTDVQAEFPGGRVIAFSHSIALR
73540185_Ralstonia_eutrop           RWQARVPDYTPPQGESLAQFHERAVEIALSLSRRHPGERIALVAHGGVLD
16799370_Listeria_innocu            KILHSPETYKANGGETYYELAERLLEGVEKIIAENPSGNILVVSHGMSLT
75908396_Anabaena_variab            KWLTEPAWNAPLGGETAVDIANRSMPVIAEIQEKHPQGNVLVVSHKATIR
                                                  *       *                    :*

15605455_Chlamydia_tracho           SLIMDLEKLSEEQVLSLELPTGQPIVYEWT
15836396_Chlamydophila_pneumo       SLIMDLEKLSEEEVLSLELPTGKPVVYQWK
50912843_Oryza_sativa               SIIMHLDKLTSQEVISLELSTGIPMLYIFK
42563306_Arabidopsis_thalia         SIIMYLDDLTSQEVTTLDLSTGVPLLYIFK
46399436_Protochlamydia_amoebo      SIIMKLDGLTTDQVVKLELATGVPVIYDFN
27375797_Bradyrhizobium_japoni      ALIMVLEKLSPEGILKRELATGVPIIYRLN
13473897_Mesorhizobium_loti         ALIMALDGKSGEEIVKLELGTGVPVIYQLN
71022513_Ustilago_maydis            AMIKDIENISDDDIVGLELATGVPILYKLD
15677454_Neisseria_mening           ALAKHIEGISDEDIMGLEIPTGQPLVYKLD
16272698_Haemophilus_influe         ALAKHIIGISDAEIMDFEIPTGQPLVLKLD
```

```
16801372_Listeria_innocu          ALVKFLEGISDDEIMELEIPTGVPLVYELN
71367566_Nocardioides_sp.         ALVKHLDGISDEDIAGLNIPTGMPLVYELD
68231626_Frankia_sp.              ALVKHLDGISDTDIANLNIPTGIPLRYELD
71274132_Homo_sapien              GIAKHVEGLSEEAIMELNLPTGIPIVYELD
Acanthmoeba_pgm                   ALVKYLDNVPESEITELNIPTAIPLVYELD
Streblomastix_pgm                 ALVKYLDNLGEDEIVLLNIPTAVPLVYDLD
23172543_Drosophila_melano        GIVKHLDNLSEDAIMALNLPTGIPFVYELD
68002649_Geobacter_metall         ALVKYLDLVSDSEIVNLNIPTGIPLVYELT
67934469_Solibacter_usitat        ALVKYLDGVSEADIVELNIPTGMPLVYELD
21673238_Chlorobium_tepidu        ALVKYLDNISDEDIVGLNIPTGIPLVYELD
73540010_Ralstonia_eutrop         ALVKYLDQISDDDIVGLNIPNGTPLVYELD
49479247_Bacillus_thurin          SLVKYLDNLSSDGVVSLNIPTSIPLVYELD
66809133_Dictyostelium_discoi     ALVKYLDNIADDKIVSMDIPTGIPLVYELD
23508399_Plasmodium_falcip        GLVKHLDNLSEADVLELNIPTGVPLVYELD
34763468_Fusobacterium_nuclea     ALIKYLLNISNEDILNLNLTTGKPLIFEMD
34539994_Porphyromonas_gingiv     GIIKVLKNISDEDIISLNLPTAVPYVFEFD
42527203_Treponema_dentic         ALVKYFENLSDEEIISVNIPTGVPLVYEFD
26246721_Escherichia_coli         ALVKYLDNMSEEEILELNIPTGVPLVYEFD
49484632_Staphylococcus_aureus    ALIKYLEDVSDEDIINYEIKTGAPLVYELT
48855514_Cytophaga_hutchi         ALMMHLEKISPEDIAHVDLATGAPRLYEFS
37521165_Gloeobacter_violac       SIIMILETLSEEEVPKVELATGVPIVYELD
76793350_Pseudoalteromonas_atl    AIIMHIEKMTAAQIAAYELKTASPHIYQCN
20091231_Methanosarcina_acetiv    ALIKHIEGISNEDIRKIRLANARPVIYTFS
75909856_Anabaena_variab          TLLCHILGLPTDNFWNFRQGNGAVSVIDYP
23128494_Nostoc_puncti            TLLCHILGLSLENFWNFRQGNGAVSVIDYP
56751043_Synechococcus_elonga     VILCHVLGLSPADIWSIKQGNGAVTVVDYP
37520340_Gloeobacter_violac       AIVAAAVGAGPEDFWRFKQGNGSVTVIDYP
33239966_Prochlorococcus_marin    TILCNLLGLTNADIWKVKQGNGGITIIDLT
76259753_Chloroflexus_aurant      ILVADALGMNMDRINRIWVTNASISVIEYG
4981935_Thermotoga_mariti         AFICWILGLPLYLHRNFKLDNASLSVVEIE
15805309_Deinococcus_radiod       TLARDLLGLPLEPQVNYPIP--YAERIKNG
73540185_Ralstonia_eutrop         CLYREATGMTLEAPRQHELLNASINRLRSD
16799370_Listeria_innocu          LLLYLLQGIEDHRKEAPRILNTSISIVEYQ
75908396_Anabaena_variab          IMLCSLLGDLGRYRYRVNILVASVSMVKFD
                                   :
```

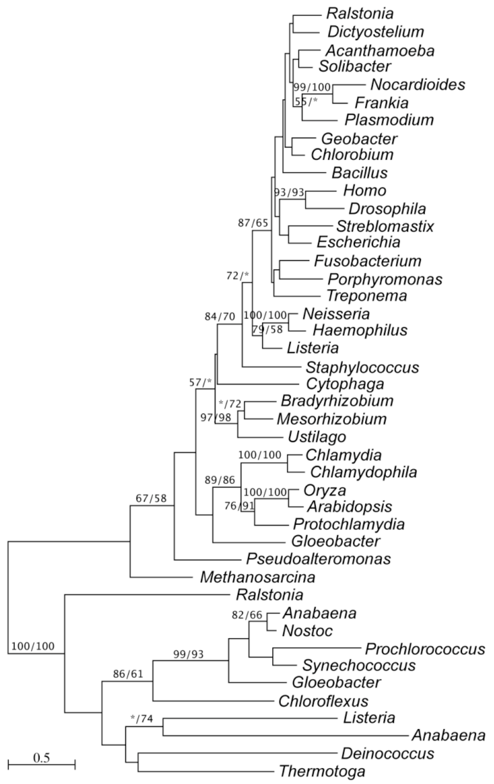

Figure 1. Phylogeny of Phosphoglycerate mutase. Note that green plant sequences form a monophyletic group with chlamydial (Particularly *Protochlamydia*) homologs. Most of the cyanobacterial sequences form a well-supported clade. The cyanobacterial *Gloeobacter* contains multiple copies of this gene (either due to duplication or gene transfer), one of which groups with plant and chlamydial sequences with insignificant support. Acanthamoeba_pgm and Streblomastix_pgm are EST sequences obtained from Taxonomically Broad EST Database.

---

2. 2-C-methyl-D-erythritol 4-phosphate cytidylyltransferase (*isp*D)

CLUSTAL X (1.83.1) multiple sequence alignment

```
33639743_Prochlorococcus_marin      HVLIPAAGSGSRMKNKLLIELEGESLIFWTIKWVGIIGQPYGGDTRQKSV
78169602_Synechococcus_sp.          HLLIAAAGSGRRMGNKLLLPLLGRPLIAWTVDWIGIVGQDIGGATRQESV
75702355_Anabaena_variab            YLLIPAAGVGKRMGNKLLLEVRSQPIIAWTLLWIGIISQPTGGSTRQESV
67924037_Crocosphaera_watson        YLLIPAAGMGKRMGNKLLLNLLGKPLLSWTLLWIGIIGQPYGGETRQQSV
35213360_Gloeobacter_violac         HLLIPAAGRGSRMGNKLLLPLSGRPLLAWTLAWIGIIGQPMGGETRQQSV
77543804_Pelobacter_carbin          YVLIPAAGMGRRMGNKQYLPLGDRPVLSHTIAHIFVICPEAGGAERQDSV
67916396_Clostridium_thermo         SAVVVAAGKGTRMNNKQYIDIDGIPLLARTLKEVVLVVNNNGGAQRQDSV
76796588_Thermoanaerobacter_et      SAVIVAAGKGIRMGNKVYLTIAGKPVLYYSLKEIVVVVSKEGGSERQYSV
2983746_Aquifex_aeolic              TAIILAAGRGSRIGRKQFATLCGKPLFMHSLEEVILVLPEDGGPERQDSV
47569820_Bacillus_cereus            TLIIPAAGQGKRMGNKLFLLINEVPIIVHTLRNIIMAINEEGGAERQDSV
67940186_Chlorobium_phaeob          YAIIAASGVGKRMKSKQFLQIGGFPVIYHTLSSVFIATRQEGGKERQDSI
```

```
19712794_Fusobacterium_nuclea       TFILAAAGQGKRMNPKQFLDYRGEPLFYSSLKDIIIITNKEGGSERQYSI
3328896_Chlamydia_tracho            SLVLLGGGKGERFNPKQYTHLCGEPLILHALHEVVVVCEEQPGTLRQDSV
33236444_Chlamydophila_pneumo       SLILLSGGQGTRFGPKQYLPLNGTPLVLHSLKEVIVVCDPSPGERRQDSV
18395376_Arabidopsis_thalia         SVILLAGGQGKRMKPKQYIPLLGQPIALYSFFEIVVVCDPFPGKERQDSV
56785242_Oryza_sativa               SVVLLSGGQGKRMGPKQYLPLLGLPIALHSLKEVVVVCDPDPGKERQDSV
46399602_Protochlamydia_amoebo      TVVLLAGGMGTRMKPKQYLTIQEKPIALHSFEQMIVVCEPQPGLRRQDSL
CM1057_Cyanidioschyzon_merolae      SVILLAGGLGSRMRPKQFLQLCGKTVSERSLLQIIIVVEKRPGLERQDSV
71542892_Syntrophomonas_wolfei      RVVIAAAGSGSRMGNKQYILLKSRPLLAYSLDEIVIVAQARGGLERQDSI
68269972_Moorella_thermo            SMIIAAAGRGRRLGNKVFLPLAGKPMLAYSLAEIIIVTRPDGGAERQDSI
67933737_Solibacter_usitat          AVILPAAGLGTRMGRKQFMLLEGSPILMHTVREIVIAVRAEGGNSRQESV
42628890_Haemophilus_influe         IVVLPAAGVGSRMQPKQYLTLLGKTLLEHTLDKIILAVSKDGGTTRAESV
75241862_Escherichia_coli           CAVVPAAGFGRRMQPKQYLSIGNQTILEHSVHRVVIAISPGGGEERADSV
30138746_Nitrosomonas_europa        IALIPAAGSGSRMGPKQYRPLASKPMIYHALRMVCVVLAPDGGATRAESV
46197745_Thermus_thermop            SVLIPAAGNGLRLGPKAFLQVGGRTLLEWTLAEVLVALPPGGGATRQASV
66796900_Deinococcus_geothe         AALIPAAGSGTRLGPKAFVEVAGQSLLARSVAEVLVALPEGGGTTRQESV
29607624_Streptomyces_avermi        AAVIPAAGRGVRLGPKALRALNGTPMLIHAVRLVVVVAPPDGGESRQESV
68174235_Frankia_sp.                AAIVPAAGRGERLGPKALRALGGRPMLVRTVEQIVVAAPPTGGAERVDSV
76782527_Mycobacterium_tuberc       VAIVPAAGSGERLAPKAFYQLDGQTLIERAVDTVVVAVPADGGSNRTDTV
77743258_Rhodopseudomonas_palu      AAIIVAGGRGLRAGPKQYRTLAGQPVIYRSMQAVQPVTSPDGGATRQQSV
17739863_Agrobacterium_tumefa       GIVIVAAGRGERAGPKQYRPIGGRAVIEHTLAPIVVVSHADGGATRQQSV
87310771_Blastopirellula_marin      GVILPAAGRSTRFGKKVYASLAGEAVWLHSAKQLIVVISPEGGAERSDSI
82749957_Staphylococcus_aureus      YAGILAGGIGSRMGPKQFLDIDNKPILIHTIEEIIIATPAQGGTDRNETI
82749960_Staphylococcus_aureus      YAGILAGGIGSRMGPKQFLDLDNKPILIHTLEKIIIATPQQGGSDRNDTI
60494614_Bacteroides_fragil         TALIVAGGKGLRMGPKQFLPIGGKPVLMRTLEQIILVLPREGGETRFHSV
48855261_Cytophaga_hutchi           FAVIVAGGSGSRMGPKQFLPIGNAPILMHTIKNIILVLPEEGGKNRFQSV
4981956_Thermotoga_mariti           VAILLAAGKGERMSPKQFLEIEGRMLFEYPLSGVVIVTRREGGDTRSQSV
45601697_Leptospira_interrog        YVLILAGGTGSRMGPKQFLELNGEPILIHSLKRIVLVSHFEGGENRHSSM
                                       : ..* . *   *         :   .   :        *  *  ::

33639743_Prochlorococcus_marin      FNGLNSKVLIHDGARCLVGPDLINKCAMEQNDAVILATKVTDTIKTPNRQ
78169602_Synechococcus_sp.          LRGLAGHVLIHDGARCLVQPDLFDRCAVAAGAALIAATPVTDTIKTPDRA
75702355_Anabaena_variab            YNGLQAQVLIHDGARCLATPNLLNSCAQAHCSGLIAAVPVKDTIKTPDRR
67924037_Crocosphaera_watson        YNGLQANVLIHDGARCLVTPNLFDRCAEENCQGLIAAIPVKDTIKTPKRS
35213360_Gloeobacter_violac         YRGLLAQVLIHDGARCLASPALFERCAAAEFSAIVAAVPVKDTIKTIPRE
77543804_Pelobacter_carbin          RNGLQAIVLIHDGARPLLPPALIEPTVAAQCGAAIVGVPVKDTIKTPDRS
67916396_Clostridium_thermo         YNGLCQIVVIHDGARPFITEEIIEECIEAEYGASTAAVPVKDTIKTLERS
76796588_Thermoanaerobacter_et      YNGITNIIAIHDGARPLVEKETVIKALKELHKAVALGVPVKDTIKTPDRK
2983746_Aquifex_aeolic              FNALLQIVVIHDSARPLATKKMFLEVAQLDYHGKVVASPARDTLKTLNRS
47569820_Bacillus_cereus            YNAIQHYVLVHDGARPFVTNKVIQDVLTAKYGASICAVPVKDTVKTVERS
67940186_Chlorobium_phaeob          YNCIELAILVHDGARPFIQPDEIDEIAALQYGACVPATKPKDTIKTLDRS
19712794_Fusobacterium_nuclea       YNAIKKIVIIQDAARPFLKDKYIEESLKIDCDGAIIGVKCKDTIKTPNRD
3328896_Chlamydia_tracho            FSGLQQWVCIHDGVRPFVYADEVIEVCSAKTGAAALASPATYTIKTLDRD
33236444_Chlamydophila_pneumo       FSGLQQWVIIHDGARPFIYPDEIHDLLETKIGATALASPIPYTIKTLDRD
18395376_Arabidopsis_thalia         YSGLQELVCIHDSARPLVNTEDVEKVLKDAVGAAVLGVPAKATIKTLDRK
56785242_Oryza_sativa               YNGLQELVCVHDSARPLVSSEDVKKVLEDVHGAAVLGVPVKATIKTLDRK
46399602_Protochlamydia_amoebo      WNGIKLLVCIHDSARPFIEIDRIRQTVAEKWGAAVLGVRVKATIKTPNRA
CM1057_Cyanidioschyzon_merolae      RSGLALLVCVHDAARPLVSRSCIRSCLRDIYGAAAIGVPVKATIKTIDRS
71542892_Syntrophomonas_wolfei      WAGLEEFVAVHDGARPFVTSELIEEILKERWGAAIPGVAVRDTLKTLDRS
68269972_Moorella_thermo            AEGLKAWVAVHDGARPFLTPALLEKTILANTGAAIAALPVKETIKTLDRK
67933737_Solibacter_usitat          ENALSSLVAVHDAVRPFIDLDIMHAVFDEETGAAIVGVPAVDTVKTLPRE
42628890_Haemophilus_influe         LNGLNAWVLVHDAARPCLQHADIDKLLAIKQGA-ILAIPVTDTIKTEDRS
75241862_Escherichia_coli           LAGLKAWVLVHDAARPCLHQDDLARLLALSRTGGILAAPVRDTMKTVDRN
30138746_Nitrosomonas_europa        TNGLKAWILVHDAARPGLSRALVERLLDQDEIGGLLAVPLADTLKTEPRE
46197745_Thermus_thermop            ARLLEALVLVHDVARPFVSRGLVARVLEARSGAAVPVLPVPDTLMVVPRE
66796900_Deinococcus_geothe         WRLLHAVVLVHDAARPFLPGAVVTALLEAETGAATAALPVADTLVLVPRE
29607624_Streptomyces_avermi        KLGLDAIVLVHDAARPLVPVDTVDAVIEAGAPAVVPALPLADTVKTPERA
68174235_Frankia_sp.                RRALRAVVLVHDAARPLTPPELVDAVAAAGHPAVIPVVPLADTVKTPPRD
76782527_Mycobacterium_tuberc       NLALTVFVLVHDAARALTPPALVARVVEAGYAAVVPVLPLSDTIKTPERA
77743258_Rhodopseudomonas_palu      YSGLEAIVLIHDAARAFVDAALISRAIVARTGAAVPTIPVTDTIKTPDRA
17739863_Agrobacterium_tumefa       LAGLEAHVMIHDAVRPFVAADMLERIAALGAGGVLPALPVTDTLKTVSRQ
87310771_Blastopirellula_marin      ANALALMIAVHDAARPCISDRLIDNVFAAEHGAAMLATPVAGTLKTVSRA
82749957_Staphylococcus_aureus      MNIIDHVIVTHDAVRPFLTQRIIKENIEVKYGAVDTVIEAIDTIVIPVRN
82749960_Staphylococcus_aureus      MNIVKHVIVTHDAVRPFLTHRIIKENIQAEYGAVDTVIDAIDTIVIPVRN
60494614_Bacteroides_fragil         KNGLALVVGIHDGVRPFVSQEVIARCFLEVRKAVIPVIDVVETVRTVSRN
48855261_Cytophaga_hutchi           RNGLNSLVAIHDGVRPFISKRIIEESFKVEKGNAVAAVELKDSIRTVNRV
```

```
4981956_Thermotoga_mariti           RSALEFYVLVHDSARPFLRKKHVSEVLRRETGAATLALKNSDALVYIPRK
45601697_Leptospira_interrog        LCGLSVIILVHDAARPFVLADELDSLCEKSDGIATLASRTSETVLFLDRE
                                       :   :  :* .*       .                   ::    *

33639743_Prochlorococcus_marin      NLWAAQTPQGFLVKRLRKAHTDDASLFEMLNWQVKIIEGNSSNIKITSPL
78169602_Synechococcus_sp.          ELWAAQTPQGFEVEQLRQGHTDDASLFERLGWSVQVLDAGPSNIKVTTPF
75702355_Anabaena_variab            NLWAAQTPQGFNVELLKQCHTDDAALFEKCGIEVQIVEGEETNLKVTTPQ
67924037_Crocosphaera_watson        NLWAAQTPQGFDVNLLKESHTDDAALFEKCQLPVKIVQGEETNLKVTTPV
35213360_Gloeobacter_violac         QLWAAQTPQGGNVGRLKAAHTDDAALCELRGEPVQVVPGEETNLKITTPA
77543804_Pelobacter_carbin          LLWQAQTPQAFRFGLIRDAHTDDASLIEWLGGRVAMIEGSYRNIKITTPE
67916396_Clostridium_thermo         TLWAVQTPQAFLKDIIMKAHTDDTVLVERMGMPVRVVMGSYNNIKITTRE
76796588_Thermoanaerobacter_et      YLWAIQTPQVFERNLIIKAHTDDTVLVERLGYKVKVVEGDYRNIKITTPE
2983746_Aquifex_aeolic              LIWHAQTPQAFRRDILLECHTDDASLLERYGYSVGVVEGSYWNVKITYPE
47569820_Bacillus_cereus            QLKAVQTPQGFSVSLLLEAHTDDASLVERIGKQVGVVEGSYYNIKVTTPE
67940186_Chlorobium_phaeob          RLLQVQTPQGFASKTLIQAHTDDAALVEEFFQHIRIHETGYHNIKITTPE
19712794_Fusobacterium_nuclea       NLIMVHTPQTFKFEILKKAHTDDASLVEMISGKIKIIYGDYDNIKITVQE
3328896_Chlamydia_tracho            ALAVIHTPQCLDTEVLREGLSDDTEAAELLGIEPTLVFSNRVQIKVTYPE
33236444_Chlamydophila_pneumo       NLAIIHTPQCIKTEILREGLVDDIEAAEIIGKPSQLVFNKHPQIKISYPE
18395376_Arabidopsis_thalia         TLWEMQTPQVIKPELLKKGFTDDVSIVEYLKHPVYVSQGSYTNIKVTTPD
56785242_Oryza_sativa               TLWEMQTPQVMRPSLLRDGFTDDVSIVEYLKHSVYITEGSYTNIKVTTPD
46399602_Protochlamydia_amoebo      FLWEMQTPQIVRLKLLYDGFSDDVSLVELIDKPVKVVEGSYLNIKITTPE
CM1057_Cyanidioschyzon_merolae      RLWEIQTPQVVRARLLRQGFTDDVSVVELLDYPVKITMGEYANIKITTPE
71542892_Syntrophomonas_wolfei      SVLAIQTPQIFRYCELHKAYTDDASLFEKYIGRVKVVPGDNRNLKITTPE
68269972_Moorella_thermo            GLWSAQTPQVFRREWLVDAYTDDAALVERLGYPVKLVPGEETNIKITTPG
67933737_Solibacter_usitat          KLVMAQTPQVFRHDLLLRAFTDESSMVERLDVEVSVVPGSDRNIKITKPT
42628890_Haemophilus_influe         QLWQAMTPQFFPVDILRDALTDEASAIELAGFRPHLVAGRSDNLKVTRPE
75241862_Escherichia_coli           GLWHALTPQFFPRELLHDCLTDEASALEYCGFHPQLVEGRADNIKVTRPE
30138746_Nitrosomonas_europa        RLWQAQTPQMFRTKLLLEALTDDASAVEALGFSPKLVTGDAYNFKVTYPQ
46197745_Thermus_thermop            AFRLVQTPQGFFTALLREAHSDDAQLVQALGYPVALVEGEATAFKITHPQ
66796900_Deinococcus_geothe         GLWAVQTPQAFRRALLLRAHTDDAGLIARLGLPVRLVPGDARLFKVTTPG
29607624_Streptomyces_avermi        RLRAVQTPQGFDRDTLVRAHTDDASMVEQLGARVVVVPGHEEAFKVTRPL
68174235_Frankia_sp.                GLRAVQTPQGFRRDVLSAAYTDDAGLVEALGVPVTTIPGAQEAFKVTRPA
76782527_Mycobacterium_tuberc       GLRAVQTPQGFTTDLLLRSYTDDASLVEHIGGQVQVVDGDPLAFKITTKL
77743258_Rhodopseudomonas_palu      TLRIAQTPQAFRYDVILEAHTDDAAIAEWAGLTVATFEGDAANMKLTTPE
17739863_Agrobacterium_tumefa       GLYAAQTPQSFSYSDILDAHTDDASIAEWTGLTVTLTEGSVDNVKLTLKR
87310771_Blastopirellula_marin      GIWEAQTPQVFRRDWFAAAYTDDAQLLEKMGRRVLIVPGSQLNRKITTQE
82749957_Staphylococcus_aureus      EMYQGQTPQSFNIKLLQDSYSDACKIIVESGHAVKLVRGELYNIKVTTPY
82749960_Staphylococcus_aureus      EMYQGQTPQSFNINLLKESYSDACKIIVETNKPVRLVKGELYNIKVTTPY
60494614_Bacteroides_fragil         DYKLVQTPQVFDADLLKRAYTDDASVVEAMGVPVYLVEGNRENIKITTPF
48855261_Cytophaga_hutchi           DYRIVQTPQTFKISQVRDAFTDDASVAEAAGYSIQLIEGSYENIKITTPE
4981956_Thermotoga_mariti           GVYRILTPQAFSYEILKKAHADDTEPVQKLGVKIALVEGDPLCFKVTFKE
45601697_Leptospira_interrog        HVWFMKTPQGIRGDVLKELLTDLCSWALTFGKTSSIVESNPLNLKITRKE
                                          ***      .     *                      *::

33639743_Prochlorococcus_marin      DLKIAKLFL
78169602_Synechococcus_sp.          DLTVAEAVL
75702355_Anabaena_variab            DLAIAEFIL
67924037_Crocosphaera_watson        DLAIAEFIL
35213360_Gloeobacter_violac         DLLVAEAIL
77543804_Pelobacter_carbin          DLVLAQAFL
67916396_Clostridium_thermo         DLIFAEVLA
76796588_Thermoanaerobacter_et      DLIVAEAFL
2983746_Aquifex_aeolic              DLEMVKKIM
47569820_Bacillus_cereus            DLLIAESFL
67940186_Chlorobium_phaeob          DIHLAEAIL
19712794_Fusobacterium_nuclea       DLKFLK---
3328896_Chlamydia_tracho            DLLFAETLL
33236444_Chlamydophila_pneumo       DLTIAQALL
18395376_Arabidopsis_thalia         DLLLAERIL
56785242_Oryza_sativa               DLLLAERLM
46399602_Protochlamydia_amoebo      DLLIAQSIL
CM1057_Cyanidioschyzon_merolae      DMILAEMII
71542892_Syntrophomonas_wolfei      DLAIARTIL
68269972_Moorella_thermo            DLVLAGALL
67933737_Solibacter_usitat          DMELAHLFL
```

```
42628890_Haemophilus_influe         DLALAEFYL
75241862_Escherichia_coli           DLALAEFYL
30138746_Nitrosomonas_europa        DLKLAELIL
46197745_Thermus_thermop            DLVLAEALA
66796900_Deinococcus_geothe         DLALAQAVA
29607624_Streptomyces_avermi        DLVLAEAVL
68174235_Frankia_sp.                DLVLAEALL
76782527_Mycobacterium_tuberc       DLLLAQAIV
77743258_Rhodopseudomonas_palu      DFVREESRL
17739863_Agrobacterium_tumefa       DIAMADEKL
87310771_Blastopirellula_marin      DLVIAEATL
82749957_Staphylococcus_aureus      DLKVANAII
82749960_Staphylococcus_aureus      DLKVANAII
60494614_Bacteroides_fragil         DLKVASALL
48855261_Cytophaga_hutchi           DLAMAEAIL
4981956_Thermotoga_mariti           DLELARIIA
45601697_Leptospira_interrog        DLDLAEVFS
                                    *:
```

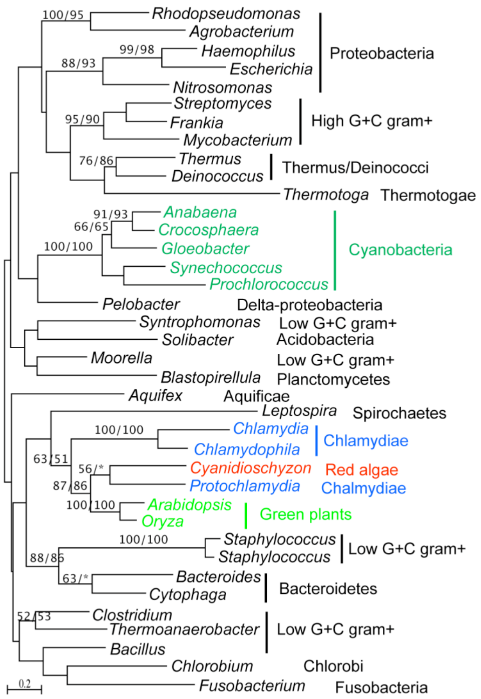

Figure 2. 2-C-methyl-D-erythritol 4-phosphate cytidylyltransferase (*ispD*). Note that sequences of primary photosynthetic eukaryotes form a monophyletic group with those of chlamydiae (in particular *Protochlamydia*) whereas cyanobacterial homologs form another distinct group. This gene is part of the DXP pathway that is mainly limited to bacterial and plastids of photosynthetic eukaryotes.

---

3. Polynucleotide phosphorylase

CLUSTAL X (1.83.1) multiple sequence alignment

```
46446277_Protochlamydia_amoebo      FETGKIARQAGGAVVVRCGETVVFTTACAASTTDFLPLRVDYQEKFSSAG
15605577_Chlamydia_tracho           FETGKIARQASGAVLVKMNETWVFSSACAAEAVDFLPFRVDYQEKFSSAG
29840519_Chlamydophila_caviae       FETGKIARQANGAVLARMQETWVFSSVCAAEPVDFLPLRVDYQEKFSSIG
50934417_Oryza_sativa               VETGHIGRQASASVMVTDGETIVYSSVCLADPSDFFPMSVHYQERLSAAG
6006870_Arabidopsis_thalia          VETGLMGRQASSAVTVTDGETIVYTSVCLAEPSDFLPLYVHYQERFSAVG
CM1081_Cyanidioschyzon_merolae      LETGFFARQAGGAVAVRSGDTVVYCTACSDPNIDFVPLRVDYVEKFSAVG
16803371_Listeria_monocy            VEVGQLAKQASGAALIRYGDTVVLTAAVGSPG-DFFPLTVNYEEKMYSVG
42523058_Bdellovibrio_bacter        IETGRLAKQADGAALVSSGNNMVLVTATSSE-LDFFPLTVEYIEKFYATG
51892669_Symbiobacterium_therm      LETGRLAKQASGSVLVNYGDSVVLCTAVGSEGVDFFPLTVDYEERMYSVG
16078732_Bacillus_subtil            VETGQLAKQANGAVMIRYGDTAVLSTATASP-LDFFPLTVNYEERLYAVG
56543019_Zymomonas_mobili           LETGQVARQADGAVIATLGETVVLCAVTAAEGQDFFPLTVHYQEKYSAAG
49176320_Escherichia_coli           LETGMMARQATAAVMVSMDDTAVFVTVVGQPGQDFFPLTVNYQERTYAAG
55981108_Thermus_thermo             LETGKYAKQASGSVLVRYADTVVLATAQASEA-DFLPLTVEFEERHYAVG
30248196_Nitrosomonas_europa        IETGEIAKQAHGAVIVSMDDTVVLVTAVGDPGQDFFPLTVDYQEKFYSAG
19712941_Fusobacterium_nuclea       VSTGKISRQSSGAIVIQYGDTVLLSTANRSKGADFFPLTVDYIEKFYSSG
48784235_Burkholderia_fungor        LETGEIARQASGAVIVDVEDTVVLATVVGAPGQDFFPLTVDYLEKTYAAG
45658536_Leptospira_interr          LETGSWAKQAHGAVVYKSGNLVLLATVCAAEGQDFFPLTCEYTEKLYSVG
15644097_Thermotoga_mariti          VQYGKVAKQSSGSALVRFGDTVVLATANISEGIDFVPLTVEFQERFYAAG
56751675_Synechococcus_elonga       LELGLFAPQAGGSVLISSGDTAVLVAATRSEGIDFLPLTVDYEERMYAAG
6459851_Deinococcus_radiod          IETGKLAKLVSGSVTVRYGDTLLLVTAQASSKLDFLPLTVEFEERHYAVG
46106240_Rubrobacter_xylano         LETGKLARQAGGAVTARFGDTTVLSTATRSPGATFLPLSVDIEERMSSAG
15073173_Sinorhizobium_melilo       LETGKIARQADGAVLATYGETVVLATVVSAPGQDFFPLTVNYQEKTYAAG
48766529_Rhodospirillum_rubrum      LETGRIARQADGAVMASLGDTTVLCTAVGASKFDFFPLTVNYQEKTFAAG
45509695_Anabaena_variab            LKVGLLAPQAGGSVLIESGDTAVLVTATRSEGIDFLPLTVDYEERLYAAG
17133532_Nostoc_sp.                 LKVGLLAPQAGGSVLIESGDTAVLVTATRSEGIDFLPLTVDYEERLYAAG
3323203_Treponema_pallid            LESGYLARQANGSVYLQCEGTAILATVCSSEGLDYVPLTVDFNEKYYAVG
48894082_Trichodesmium_erythr       LKVGLFAPQAGGAVMIESGDTAVLVTATTAEGIDFLPLLVDYEERLYAGG
53715606_Bacteroides_fragil         LETGKLAKQADGSVMLRMGNTMLLATVCAAPGTDFMPLQVEYKEKFAAFG
15605776_Aquifex_aeolic             IETGKYAKLTDGSVVVRQGGTAVLVTAVMSTDVDFTPLAVDYRERASAYG
21674467_Chlorobium_tepidu          IETGKMAKQADGSAVVTMNDTMVLATVVSSPNQDFFPLQVEYREKYSAAG
32141272_Streptomyces_coelic        FETGRLARQAAGSAVAYLDDDTMVLSATSADQLDFFPLTVDVEERMYAAG
15827380_Mycobacterium_leprae       FETGRLALQAAGAVVAYLDADNMLLSATTAEQFDFFPLTVDVEERMYAAG
24943088_Homo_sapien                ISSGKLARFADGSAVVQSGDTAVMVTAVSKPS-QFMPLVVDYRQKAAAAG
24651641_Drosophila_melano          FSSGRLARFANGTAVCQMGDTAVMVTAVAKPGQGFMPLVVDYRLKNAASG
50910551_Oryza_sativa               FETGKMARFANGSVVISMDDTHVLSTVAAAPVRDFLPLTVDYQEKQYAQG
                                    .. *  .    .:         :  :.       : *:  .   :  : *

46446277_Protochlamydia_amoebo      KTLGGFIKREGRPTEKEVLVSRLIDRPIRPMFEEGYYNEVQLLSFVWSYD
15605577_Chlamydia_tracho           RTSGGFLKREGRPSEREILVSRLMDRSLRPSFPNRLMQDIQVLSYVWSYD
29840519_Chlamydophila_caviae       KTLGGFIKREGRPTEREILTSRLIDRSMRPSLPNRLMQDVQVLSYVWSYD
50934417_Oryza_sativa               RTSGGFFKREGRAKDHEVLVCRLIDRPLRPTMPKGFYYETQILSWVFSYD
6006870_Arabidopsis_thalia          RTSGGFFKREGRTKDHEVLICRLIDRPLRPTMPKGFYNETQILSWVLSYD
CM1081_Cyanidioschyzon_merolae      RTSAGYLKRE-RPGDREIQVSRLIDRPLRPCIAEGFFKETQVLANVFSFD
16803371_Listeria_monocy            KVPGGFLKREGRPSDRATLTARLIDRPIRPLFAEGFRNEVQITSTVFSVD
42523058_Bdellovibrio_bacter        KIPGGYFKREAKPTNDAVLIARLIDRPIRPVFPEGYRHETQIVATVLSAD
51892669_Symbiobacterium_therm      RIPGNWFRREGRPTSKAILWARLTDRPIRPLFPEGFRNDVQVVCTVLSVD
16078732_Bacillus_subtil            KIPGGFIKREGRPSEKAVLASRLIDRPIRPLFADGFRNEVQVISIVMSVD
56543019_Zymomonas_mobili           RIPGGFFKRERGATERETLISRLIDRPIRPLFPEGFYNETLVIAQVMSYD
49176320_Escherichia_coli           RIPGSFFRREGRPSEGETLIARLIDRPIRPLFPEGFVNEVQVIATVVSVN
55981108_Thermus_thermo             KIPGSFMRREGRPGEKAILSARMTDRPIRPLFPKGFRHEVQIIVTVLSAD
30248196_Nitrosomonas_europa        RIPGSFFKREGRPSEKETLTSRLIDRPIRPLFPDGFYNEVQVVAMVLSSD
19712941_Fusobacterium_nuclea       KFPGGFNKREGRPSTNATLVARLIDRPIRPMFPDGFNYDVHIVNTVLSYD
48784235_Burkholderia_fungor        KIPGGFFRREGRPSEGETLISRLIDRPLRPLFPEGFYNEVQVVIHVLSLN
45658536_Leptospira_interr          RFPGGYFKREAKPPEHEILISRIIDRPIRPLFPEGYFCEVQLQVQVLSAD
15644097_Thermotoga_mariti          KIPGGFIKREGKPSESAILSARLIDRPIRPLFPKKLRNEVQVIVTVLSVD
56751675_Synechococcus_elonga       RIPGGFLRREGRPPERATLTSRLIDRPLRPLFPSWLRDDLQVVATTLSMD
6459851_Deinococcus_radiod          KIPGSFQRREGRPGEKAILSARITDRQIRPLFPKGYRHETQVIITVLSAD
46106240_Rubrobacter_xylano         KIPGGFLKREGKPSEKAILTARLTDRPIRPLFPKDYYYEVQVIGTVLVAD
15073173_Sinorhizobium_melilo       KIPGGYFKREGRPSENETLVSRLIDRPIRPLFPDGYKNDTQVIITVMQHD
48766529_Rhodospirillum_rubrum      KIPGGFFKREGRPSEKETLVSRLIDRPIRPLFVHGYKNETQLVCTVLCHD
45509695_Anabaena_variab            RIPGGIMRREGRPPEKTILTSRLIDRPLRPLFPSWLRDDLQIVALTMSMD
17133532_Nostoc_sp.                 RIPGGIMRREGRPPEKTILTSRLIDRPLRPLFPSWLRDDLQVVALTMSMD
3323203_Treponema_pallid            KMPGGFIKREGRPKDREILISRLIDRPMRPLFEKEFGRDIHVVPTCISSD
48894082_Trichodesmium_erythr       KIPGGFLRREGRPPEKVTLTSRLIDRPLRPLFPSWLRDDIQIVATTLSMD
53715606_Bacteroides_fragil         RFPGGFTKREGRASDYEILTCRLVDRALRPLFPDNYHAEVYVNIILFSAD
```

```
15605776_Aquifex_aeolic             RIPGGFTKREGKPTDREILVSRVIDRPIRPLFPEGFFHDVIITALTLSAD
21674467_Chlorobium_tepidu          KFPGGFFKREGRPSEKEILSARLIDRALRPLFPDGYYQETQIIISVISSD
32141272_Streptomyces_coelic        KIPGSFFRREGRPSEDAILTCRLIDRPLRPSFKKGLRNEIQVVATIMALN
15827380_Mycobacterium_leprae       RIPGSFFRREGRPSTDAILTCRLIDRPLRPSFVDGLRNEIQVVVTILSVD
24943088_Homo_sapien                RIPTNYLRREVGTSDKEILTSRIIDRSIRPLFPAGYFYDTQVLCNLLAVD
24651641_Drosophila_melano          RIPMNFMRRELGPSEKEILSARLIDRSLRPLFHKDYRTETQLVCNMLAMD
50910551_Oryza_sativa               VIPTTYMRREGAPKERELLCGRIIDRPIRPLFPPGFYHEVQIMVNVISSD
                                           :**  .        *: ** :** :      :  :       :

46446277_Protochlamydia_amoebo      GINSPEPLAICGASAALVISDIPLIKPVGAVRIGFIDAQFIVNPTIEQQK
15605577_Chlamydia_tracho           GKTLPDPLAICGASAALAISEVPQNCIVAGVRVGLVGGKWVINPTRDELS
29840519_Chlamydophila_caviae       GVTLPDPIAICGVSAALAISDIPQISVVAGVRVGFVNNSWVVNPTKAEMD
50934417_Oryza_sativa               GIHSPDSLAITAAGVAMALSEVPNKQTIAGVRVGMINDQFVVNPTTEQMD
6006870_Arabidopsis_thalia          GLHAPDALAVTSAGIAVALSEVPNAKAIAGVRVGLIGGEFIVNPTVKEME
CM1081_Cyanidioschyzon_merolae      GTHQADTLAICGAGAALALSPVPMCARVAGVRIGMRDGSFVVNPTEQQMR
16803371_Listeria_monocy            QDCSPEMAAMLGSSVALVISDIPFEGPIAGVDVGRIDGKYVINPTIEQAE
42523058_Bdellovibrio_bacter        GAFPLEILASLGASAALHCSDIPFNGPTAAVQIARVDGQFVANPTPQQME
51892669_Symbiobacterium_therm      PDHPVEIMGMIGASAALTISDIPFEGPIGGVIVGLVDGQFVINPTAEQQE
16078732_Bacillus_subtil            QNCSSEMAAMFGSSLALSVSDIPFEGPIAGVTVGRIDDQFIINPTVDQLE
56543019_Zymomonas_mobili           GENEPDILAMIAASAALALSGVPFLGPIGAARVGYQDGEFILNPTLEQLE
49176320_Escherichia_coli           PQVNPDIVAMIGASAALSLSGIPFNGPIGAARVGYINDQYVLNPTQDELK
55981108_Thermus_thermo             QKNPPDILGPIAASAALMLSDIPWEGPIAAVRVGLIGGSFVLNPTLQELE
30248196_Nitrosomonas_europa        TEIDADIPAMIGTSAALILSGIPFDGPVGAARVGYINNEYILNPTTTQLK
19712941_Fusobacterium_nuclea       EINIPDYLGVIGSSLALMISDIPFLGPVASVIVGYKNGEFILNPSPKELE
48784235_Burkholderia_fungor        PEIPADIPALIGASAALAVSGLPFNGPVGAARVAYINNEYVLNPTRPQMK
45658536_Leptospira_interr          GDVSVAGHALNAASAALAVSDIPFNGPIAGARIGRVNGELILNPTTKEIL
15644097_Thermotoga_mariti          PNVPPDVVGIFAASLALNVSKIPFEGIVAGIRVGYRDGQFIALPSEEDIE
56751675_Synechococcus_elonga       ETVPPDVLAATGASIATLVAKIPFYGPMAAVRVGLVGDDFIINPTYREIE
6459851_Deinococcus_radiod          GQNAPDVLGPIGAAAALSISDIPWAGPTACVRVGQIDGQYVVNPTTEQLT
46106240_Rubrobacter_xylano         QENPYDVVSMVGASAALALSDIPFGGPIGAVRVGRHDGGFILNPTYQQLE
15073173_Sinorhizobium_melilo       LENNPDVVAMVAASAALTLSGVPFMGPVGGARVGYINGEYVLNPHLDEMD
48766529_Rhodospirillum_rubrum      LENNPDIVAIVGASAALTLSGLPFLGPIGAARVGLIDGAFVLNPTRDQMA
45509695_Anabaena_variab            EQVPPDVLAVTGASIATLIAKIPFNGPMAAVRVGLVGDDFIINPTYAEIE
17133532_Nostoc_sp.                 EQVPPDVLAVTGASIATLIAKIPFNGPMAAVRVGLVGDDFIINPTYAEIE
3323203_Treponema_pallid            MVHPHDVLAIVASSAAVTLSDIPFHGPVAAVRVAYLNGSYVINPTFSQID
48894082_Trichodesmium_erythr       EEVPPDVLAVTGSSVAVLLAKLPFYGPMAAVRVGLVGDDFIINPTYREVK
53715606_Bacteroides_fragil         GVDMPDALAGLAASAALAVSDIPFNGPISEVRVARIDGKFVINPTFDQLE
15605776_Aquifex_aeolic             DKYDPDVLAITGASAALHISRIPFEGPIAGVRVCRVNGEFVANPTYEQRK
21674467_Chlorobium_tepidu          TINDADVLGGIAASAAIMVSDIPFANPMSEVRVGRINGLFIVNPDINELA
32141272_Streptomyces_coelic        PDHLYDVVAINAASASTQLAGLPFSGPIGGVRVALIRGQWVAFPTHTELE
15827380_Mycobacterium_leprae       PNDLYDVLAINAASASTQLGGLPFSGPIGGVRVALIDGTWVAFPTVEQLE
24943088_Homo_sapien                GVNEPDVLAINGASVALSLSDIPWNGPVGAVRIGIIDGEYVVNPTRKEMS
24651641_Drosophila_melano          AVHSPDVLAINAASMALSLSDIPWNGPIGAVRVGLCDGEVLINPTRRELQ
50910551_Oryza_sativa               GKQDPDVMAANASSAALMLSDIPWNGPIGVIRVGRIDGNFVLNPTVDELG
                                            .  . . :   . :*     .   :       :  *   :

46446277_Protochlamydia_amoebo      QSKLDLLIAGTEEAVLMIEGFCDFLTEDQVLEAIEIGHRSIKTICQTLYP
15605577_Chlamydia_tracho           ASKLDLVMAGTASAVLMIEGHCDFLTEEQVLEAIAFGQTYIAKICDAIFS
29840519_Chlamydophila_caviae       VSRMELVLAGTENAILMIEGHCDFLTEEQVIEAIEFGHKHIATICRAIFT
50934417_Oryza_sativa               DSELDLVMAGTDSAILMIEGYCDFLTEEKLLQAVETGQGAIREICKAIFS
6006870_Arabidopsis_thalia          ESQLDLFLAGTDTAILTIEGYSNFLPEEMLLQAVKVGQDAVQATCIAIFS
CM1081_Cyanidioschyzon_merolae      GECMDMVVAGTSSAVLMIEAGCLFASEQQIAEAIEVAQRAIAQICEGIAF
16803371_Listeria_monocy            KSDISLTVAGTYDAINMVEAGAKEVSEEAMLEAIMFGHEEIKRLCEFQEI
42523058_Bdellovibrio_bacter        KSDMDMIVAGTRNGLLMVEGETKFISEADALAALKFGHQSLIPLLNAQQR
51892669_Symbiobacterium_therm      RSEMHLVVAGTEHAVLMVEAGAKEVPEQTMLDAILFGHEVIKNIIPTISE
16078732_Bacillus_subtil            KSDINLVVAGTKDAINMVEAGADEVPEEIMLEAIMFGHEEIKRLIAFQDT
56543019_Zymomonas_mobili           KSDLDLVVGATRDAVMMVESEANELPEEVMLNAVSFAHESLQPVIKAITE
49176320_Escherichia_coli           ESKLDLVVAGTEAAVLMVESEAQLLSEDQMLGAVVFGHEQQQVVIQNILD
55981108_Thermus_thermo             ESQLDLVVAGSKEAILMVEAEAGEVDEETLVQALEFAHKEMQPILELQGK
30248196_Nitrosomonas_europa        ESQLNLVVAGTQKAVLMVESEADELSEDVMLGAVTYGHDQMQQVIDMIPS
19712941_Fusobacterium_nuclea       ESELDLIVAGTKEAVNMVEAGAKELDEETMLKAIMFAHENIKKICEFQDI
48784235_Burkholderia_fungor        ESALDLIVAGTERAVLMVESEAQQLSEEVMLGGVVFGHEQMQIAIDAIAD
45658536_Leptospira_interr          NSDLDLVVAGTKTHIVMIEGEAKELSNEEMIAALRFAQKHIAEFVTLQDK
15644097_Thermotoga_mariti          KGLMDITVAGTKDAVTMVEGEAKEVTEEDMVKALRFAHSVIKELVDFQGI
56751675_Synechococcus_elonga       KGDLDLVVAGTPAGVIMVEAGANQLPEADVIEAIDFGYEAVQELIKAQAE
```

```
6459851_Deinococcus_radiod          RSRMDLVVAGTREAVMMVECGAQTVSEDDLVGAIEFAHAEMQGVIALILT
46106240_Rubrobacter_xylano         ESDLDIVVAGTKEAITMVEAGAREVTEDVVVEALEVAQGVIREQAEAIEE
15073173_Sinorhizobium_melilo       ESTLDLVVAGTQEAVLMVESEAKELPEDVMLGAVVFGQKGFQPVIDAVHT
48766529_Rhodospirillum_rubrum      DSVLDLVVAGTREGVLMVESEAHELSEQQMLDAVMFGHEGYQPVIDAIAA
45509695_Anabaena_variab            AGDLDLVVAGSPHGVIMVEAGANQLPERDIIEAIDFGYEAVRDLIKAQAN
17133532_Nostoc_sp.                 AGDLDLVVAGSPHGVIMVEAGANQLPERDIIEAIDFGYEAVRDLIKAQAN
3323203_Treponema_pallid            AASMEVVVAGTRQGITMVEGGAREVSEDLMLGALEQAQEHIKALCDMQVQ
48894082_Trichodesmium_erythr       NGDLDLVVAGSPQGVIMVEAGANQLPEQDIIEAIDFGYEAVCDLIQAQSE
53715606_Bacteroides_fragil         QADMDIMVAATYENIMMVEGEMSEVSEAELLEAMKVAHEAIKVHCKAQEK
15605776_Aquifex_aeolic             EADLDIVMAGTKDAIVMVEGGGKEIPEEVLADALFFGLDAIKEVIEAQEL
21674467_Chlorobium_tepidu          QSDMDICIGGTEDTICMLEGEMKEISEAEMLDAIKFGHDAIKKICALQLN
32141272_Streptomyces_coelic        DAVFDMVVAGTDKTIKLVEGGAEAPTEEVVAAGLDAAKPFIKVLCRAQGR
15827380_Mycobacterium_leprae       RAVFDMVIAGTENVIELVECGAQAPTESIVAQGLEVAKPFIAALCTAQGR
24943088_Homo_sapien                SSTLNLVVAGPKSQIVMLEASAENILQQDFCHAIKVGVKYTQQIIQGIAD
24651641_Drosophila_melano          TSQLDLVVSAKQNLVVMLEGKGNVVLQQDLLKAIKQGTREAQFIIHEITE
50910551_Oryza_sativa               LSDLNLVYACSRDKTLMIDVQAREITERDLQAGMKLAHAEAVKCINPQDS
                                       : :  .        ::       :     .:  .

46446277_Protochlamydia_amoebo      AKHIAYVIKDVSSKMMRQMILNENVRSDGRTSTDIRFIDIEQSLLPRAHG
15605577_Chlamydia_tracho           LLNVKAAFKTAKSNQMRALIQDLGIRVDGRTTTEIRPISIETPFLPRTHG
29840519_Chlamydophila_caviae       ALNIKTAFKQAKSDYMRALIREQSVRSDGRAITTIRPISIDTSFLPRTHG
50934417_Oryza_sativa               EVDVKLVFKEVSSKFLRRRIVEGGKRSDGRSPCELRPINSQCGLLPRAHG
6006870_Arabidopsis_thalia          EVDVKLVFKEVSSKLLRRRIVEGGKRSDGRTLDEIRPINSRCGLLPRAHG
CM1081_Cyanidioschyzon_merolae      ETLCRMAWKRVLSSEMRRLIVREKVRADGRKPHQVRPIDIVQTPLPCAHG
16803371_Listeria_monocy            LSEVAHILEMIEKDEMRRLISQDKIRPDGRKVNEIRPLSSEVGMLPRVHG
42523058_Bdellovibrio_bacter        KKELNSIVEDLKYQEARSMILDRAVRIDGRDVKTVRPIANEVGILPRAHG
51892669_Symbiobacterium_therm      K-HIAYLLKKVLKEEVRRAIIEEGIRPDGRSLTEIRPIECAVDLLPRPHG
16078732_Bacillus_subtil            IKQVKQILSKLVKNEVRRLITEEKVRPDGRGVDQIRPLSSEVGLLPRTHG
56543019_Zymomonas_mobili           KLRIQKLAKKLEAKIVRTAILKEGRRIDGRDLKTVRPIRSQVGFLPRTHG
49176320_Escherichia_coli           ENELGEILHAIEKNVVRSRVLAGEPRIDGREKDMIRGLDVRTGVLPRTHG
55981108_Thermus_thermo             KPLYESAFDEVVRRELRRLVLEEGKRADGRGPKDLRPIWIEVDVLPRAHG
30248196_Nitrosomonas_europa        EQAVREIGFALEARIVRNSILDGESRIDGRGTRTVRPITIRHGVLPRTHG
19712941_Fusobacterium_nuclea       ILEFKTYYHDLMKKLVREAILYHKHRVDGRTTTEIRPLDAQINVLPIPHG
48784235_Burkholderia_fungor        KATVGNVLFDIEAKIVRTQILNGEPRIDGRDTRTVRPIEIRTGVLPRTHG
45658536_Leptospira_interr          IKDIKAYLHELEYEIVREQVLTKGTRFDGRKLDEIRSISVEINPLPGPHG
15644097_Thermotoga_mariti          KPFVSELYEDAVKKTMRRLIVEKGIRADGRKPTEIRPISCEVGLFPRTHG
56751675_Synechococcus_elonga       PKALGNTFKALTKTLMRQQILRDGVRVDGRKLDQVRPISSQVGLLPRVHG
6459851_Deinococcus_radiod          Q-ALKDAFGKVEKRELRRLILEENLRADGRDSKTVRPIWIEARPLPTAHG
46106240_Rubrobacter_xylano         VPLVLDALAQLQKEAFRKLYLEDRKRTDLRAFDEIRPTTAEAHVLPRVHG
15073173_Sinorhizobium_melilo       AEEIAAVFKHLQAKIVRWNILDTKSRIDGRDLVTVRPIVAEVGILPRTHG
48766529_Rhodospirillum_rubrum      LALAGAAFKDLEKDIVRGSILDTGLRIDGRDTKTVRPIEIYPGILPRAHG
45509695_Anabaena_variab            SKALGNTFKDITKYFMRRQIVEDNVRVDGRKLDQVRPVSSQVGVLPRVHG
17133532_Nostoc_sp.                 SKALGNTFKDITKYFMRRQIVEDNVRVDGRKLDQVRPVSSQVGVLPRVHG
3323203_Treponema_pallid            RRLFDALFHEMEYEILRLNILDRGLRIDGRAIDAIRPIACEVGVLPRPHG
48894082_Trichodesmium_erythr       SMLLGNAFKSVTKKLMRQQIVAEGIRVDGRKLDEVRPVSCRVGVLPRVHG
53715606_Bacteroides_fragil         GALIDRYYHDVEKEAMRRCILDEGKRLDGRKTTEIRPIWCEVGYLPGPHG
15605776_Aquifex_aeolic             HFAVKYFYKKLESRLMREKVLKEGVRIDGRKPNEIRPIWIEVHPFERPHG
21674467_Chlorobium_tepidu          EHMIEECIHAVEKKVMRHMILDDGKRLDGRTLEQVRPISIELGLIPRAHG
32141272_Streptomyces_coelic        EKEISAAYRALTKSLVRERVIKEKKRIDGRGVTDIRTLAAEVEAIPRVHG
15827380_Mycobacterium_leprae       EKELGAAFRSLTKKLVRQRILTNHFRIDGRGITDIRALSAEVAVVPRAHG
24943088_Homo_sapien                PYEIIESFNVVAKEVFRSIVLNEYKRCDGRDLTSLRNVSCEVDMFKTLHG
24651641_Drosophila_melano          PSLITEQFNQTSRTIFRELIFERGLRCDGRDYDQLRNISCQVDMYKPLHG
50910551_Oryza_sativa               LKFLHKAVDTVRKQVIRKRIIEKGLRVDGRQLDEVRPLYCESSTYPILHG
                                                    *        * * *    :*            **

46446277_Protochlamydia_amoebo      SSLFTRGETQALAVCTLGGASMAQRFEDLEFYLQYSFPPYSVGEVGRVGA
15605577_Chlamydia_tracho           SCLFTRGETQSMAVCTLGGENMAQRFEDLNFYLQYFFPPFSVGEVGRIGS
29840519_Chlamydophila_caviae       SCLFTRGETQTVAVCTLGSEAMAQRYEDLNFYLQYFFPPFSVGEVGRIGS
50934417_Oryza_sativa               SALFTRGETQALAVVTLGDYQMAQRIDNLVFYLQYTFPPSSVGEVGRIGA
6006870_Arabidopsis_thalia          STLFTRGETQALAVVTLGDKQMAQRIDNLEFYLQYTFPPSSVGEVGRIGA
CM1081_Cyanidioschyzon_merolae      STLFTRGETQTLAVVTLGGQDSAQRSESLLFYLQYMFPPFAVGEVGRIGA
16803371_Listeria_monocy            SGLFTRGQTQALSVCTLAPLREHQIIDGLGFMHHYNFPQFSVGETGPRRA
42523058_Bdellovibrio_bacter        SGLFTRGETQVLGTVTLGTADDEQMVDSLLFMLHYNFPPYSVGEVGRMSG
51892669_Symbiobacterium_therm      SGLFQRGQTQVLSICTLGTLGDMQKLDDLTFMHHYNFPPYSVGETRPLRG
16078732_Bacillus_subtil            SGLFTRGQTQALSVCTLGALGDVQILDGLGFMHHYNFPQFSVGETGPMRG
```

```
56543019_Zymomonas_mobili           SALFTRGETQALVSTTLGTADAEQMIDGLTFMLHYNFPPYSVGEVGRFGA
49176320_Escherichia_coli           SALFTRGETQALVTATLGTARDAQVLDELMFLFHYNFPPYSVGETGMVGS
55981108_Thermus_thermo             SAVFTRGETQVLGTVTLGTGRDEQILDDLGFLVHYNFPPFSTGEVRRLRG
30248196_Nitrosomonas_europa        SALFTRGETQALAITTLGTARDEQKIDALQFMLHYNMPPFATGETGRVGT
19712941_Fusobacterium_nuclea       SALFTRGETQSLAITTLGTKEDEQLIDDLEFYLHYNFPPYSVGEVGRMGS
48784235_Burkholderia_fungor        SALFTRGETQALVVATLGTKGDEQNIDALEFMLHYNMPPFATGETGRVGS
45658536_Leptospira_interr          SAVFTRGQTQSLGVVTLGTGSDNQRYETLEFMLHYNFPAFSVGEVRRSSG
15644097_Thermotoga_mariti          SALFTRGETQSLGIVTLGAPMDVQIIDTLLFMLHYNFPPFCTGEVKPLRG
56751675_Synechococcus_elonga       SGLFQRGLTQVLSIATLGTPGDAQELDDLHYLHHYNMPPYSVGETRPMRS
6459851_Deinococcus_radiod          SAVFTRGETQVLGVTTLGTERDEILIDDLTFLLHYNFPPYSTGEVKRMGG
46106240_Rubrobacter_xylano         TGLFTRGETQVLSSLALADLGLVQRLDTLEYMHHYYFPPYSTGETGRLGP
15073173_Sinorhizobium_melilo       SALFTRGETQAIVVATLGTGEDEQYVDSLTFMLHYNFPPYSVGETGRMGS
48766529_Rhodospirillum_rubrum      SALFTRGETQALVTTTLGTGQDEQIIDALEFMLHYNFPPYSVGEAGRMGS
45509695_Anabaena_variab            SGLFNRGLTQVLSACTLGTPGDAQNLDDLQYLHHYNFPPFSVGETKPLRA
17133532_Nostoc_sp.                 SGLFNRGLTQVLSACTLGTPGDAQNLDDLQYLHHYNFPPFSVGETKPLRA
3323203_Treponema_pallid            SAVFTRGETQSLAVVTLGAMSDGQVYDDIEFILHYNFPPFSVGEIGRMG-
48894082_Trichodesmium_erythr       SSLFNRGLTQVMSAVTLGTPGDAQELDDLHYLHHYNFPPFSVGETKPLRQ
53715606_Bacteroides_fragil         SAIFTRGETQSLTSVTLGTKLDEKIIDDVLFLLHYNFPPFSTGEAKAQRG
15605776_Aquifex_aeolic             NAIFTRGQTQAYVTVTLGTPDEALIIETIAFMLHYSMPPFSVGEAKPWGP
21674467_Chlorobium_tepidu          SALFTRGETQALVTLTLGTKKDAQSVDTLTFMLHYNFPPFSVGEIGRVGG
32141272_Streptomyces_coelic        SALFERGETQILGVTTLNMLRMEQQLDTLSYMHNYNFPPYSVGETGRVGS
15827380_Mycobacterium_leprae       SALFERGETQILGVTTLDMVKMAQQIDSLGYMHHYNFPPFSTGETGRVGS
24943088_Homo_sapien                SALFQRGQTQVLCTVTFDSLESGIKSDQVIFMLHYEFPPYATNEIGKVTG
24651641_Drosophila_melano          SALFQRGQTQVFCTVSLDSQESAMKLDSLAFMLHYEFPPYATGEVGRIGP
50910551_Oryza_sativa               SALFSRGDTQVLCTVTLGAPGDAQRLDSIVFMLHYSFPPFSINEVAKRGG
                                    . :* ** **     ::         : : :  :* :*  . .*

46446277_Protochlamydia_amoebo      PGRREIGHGKLAERALMAVIFPYTIRLESNITESNGSSSMATVCGGCLAL
15605577_Chlamydia_tracho           PGRREIGHGKLAEKALSHVLFPYIIRLESNITESNGSSSMASVCGGCLAL
29840519_Chlamydophila_caviae       PGRREIGHGKLAEKALSHTLFPYTIRIESNITESNGSSSMASVCGGCLAL
50934417_Oryza_sativa               PNRREIGHGMLAERALEPILFPYTIRVESTITESNGSSSMASVCGGCLAL
6006870_Arabidopsis_thalia          PSRREIGHGTLAERALETILFPYTIRVESTVIESNGSSSMASVCGGCLAL
CM1081_Cyanidioschyzon_merolae      PGRREIGHGMLAQRALEPVVFPYVIRVESNIMESNGSSSMASVCGGSLAL
16803371_Listeria_monocy            PGRREIGHGALGERALQYVIFPYTIRLVSEVLESNGSSSQASICGSTLAM
42523058_Bdellovibrio_bacter        TSRREIGHGNLAERAIKAVLFPYTIRIVSEVLESNGSSSMGTVCSGIMAL
51892669_Symbiobacterium_therm      PGRREIGHGALAERALEPVIFPYTIRVVSEVLESNGSSSMASVCGSTLAL
16078732_Bacillus_subtil            PGRREIGHGALGERALEPVIFPYTVRLVSEVLESNGSTSQASICASTLAM
56543019_Zymomonas_mobili           PGRREIGHGKLAWRALHPVLFPYTIRVLSDITESNGSSSMATVCGGCLAL
49176320_Escherichia_coli           PKRREIGHGRLAKRGVLAVMFPYTVRVVSEITESNGSSSMASVCGASLAL
55981108_Thermus_thermo             VSRREVGHGNLAKRALKAVMFPYTIRVVGDVLESNGSSSMATVCAGCLAL
30248196_Nitrosomonas_europa        PKRREIGHGRLAKRALLAVIFGYSMRVVSEVTESNGSSSMASVCGGCLSL
19712941_Fusobacterium_nuclea       PGRRELGHGSLAERALSYVIFPYTIRVVSEITESNGSSSQASICGGSLSL
48784235_Burkholderia_fungor        PKRREIGHGRLAKRALAACLFGYSIRVVSEITESNGSSSMASVCGGCLAL
45658536_Leptospira_interr          PGRREIGHGNLAERALKLVLFPYVIRVVSEILESNGSSSMASVCSGSLAL
15644097_Thermotoga_mariti          PSRREIGHGHLAERALKNMLFPYTIRVVSEILESNGSSSMATVCSGSLAL
56751675_Synechococcus_elonga       PGRREIGHGALAERALLPVLFPYVIRVVSEVLSSNGSTSMGSVCGSTLAL
6459851_Deinococcus_radiod          QSRREIGHGNLAKRAIRAVLFPYVIRVVGDVLESNGSSSMGTVCAGTLSL
46106240_Rubrobacter_xylano         PRRREIGHGALAERALLPVIFPYAIRIISEVLESNGSSSMASVCGSTLAL
15073173_Sinorhizobium_melilo       PGRREIGHGKLAWRAIHPMLFPYTLRVVSEITESNGSSSMATVCGTSLAL
48766529_Rhodospirillum_rubrum      PGRREIGHGKLAWRAIHPMMFPYTVRVVSEITESNGSSSMATVCGTSLAL
45509695_Anabaena_variab            PGRREIGHGALAERAILPVLFPYVIRVVSEVLSSNGSTSMGSVCGSTLAL
17133532_Nostoc_sp.                 PGRREIGHGALAERAILPVLFPYVIRVVSEVLSSNGSTSMGSVCGSTLAL
3323203_Treponema_pallid            VGRREIGHGCLAHRSLSAVIFPYTVRVVSEILESNGSSSMATVCSGTLSL
48894082_Trichodesmium_erythr       PGRREIGHGALAERALIPVIFPYVIRVVSEVLSSNGSTSMGSVCASTLGL
53715606_Bacteroides_fragil         VGRREIGHGNLAHRALKRMIYPYVVRVVSDILESNGSSSMATVCAGTLAL
15605776_Aquifex_aeolic             PRRREIGHGALAERAIEPLLYPFIIRVVSDILESNGSTSMATVCGASLAL
21674467_Chlorobium_tepidu          AGRREIGHGNLAERAIKMVMFPYTVRLVSDILESNGSSSMASVCGGTLAA
32141272_Streptomyces_coelic        PKRREIGHGALAERAIVPVLFPYAIRQVSEALGSNGSTSMGSVCASTMSL
15827380_Mycobacterium_leprae       PKRREIGHGALAERALVPVLFPYAIRQVSEALGSNGSTSMGSVCASTLAL
24943088_Homo_sapien                LNRRELGHGALAEKALYPVIFPFTIRVTSEVLESNGSSSMASACGGSLAL
24651641_Drosophila_melano          VGRREMGHGALAERSLLPTLYPFTVRLTSEVLESNGSSSMASVCGGSLAL
50910551_Oryza_sativa               LNRREVGHGTLAEKALLAVLFPYTVRVNSEVMASDGSTSMASVCGGSMAL
                                      ***:*** *. :.:   :: : :*  .    *:**:* .: *.  :.

46446277_Protochlamydia_amoebo      MDAGVAIKRPVAGIAMGLILENFIILSDILGIEDALGDMDFKVTGDQNGI
```

```
15605577_Chlamydia_tracho           MDAGVPIKAPVAGIAMGLILDRAIILSDISGIEDHLGDMDFKVAGTAKGI
29840519_Chlamydophila_caviae       MDAGVPIKTPIAGIAMGLILDNVTILSDISGLEDYLGDMDFKVAGNTEGI
50934417_Oryza_sativa               QDAGVPIKFPVAGIAMGLVLDTPLILSDITGAEDASGDMDFKVAGNENGI
6006870_Arabidopsis_thalia          QDAGVPVKCSVAGIAMGMVWDTPLILSDITGAEDASGDMDFKVAGNEDGV
CM1081_Cyanidioschyzon_merolae      MDAGVPVSAAVAGVAMGLILDQYLILTDILGMEDALGDMDFKVAGTRTGI
16803371_Listeria_monocy            LDAGVPIKAPVAGIAMGLVKLGYTILSDIQGMEDHFGDMDFKVAGTKDGI
42523058_Bdellovibrio_bacter        LDAGVPLKGNVAGVAMGLIKEGVAVLTDILGDEDHLGDMDFKVAGSPAGI
51892669_Symbiobacterium_therm      MAAGVPIKAPVAGVAMGLVEDPYAVLTDIQGIEDALGDMDFKVAGTKKGV
16078732_Bacillus_subtil            MDAGVPIKAPVAGIAMGLVKSGYTVLTDIQGMEDALGDMDFKVAGTEKGV
56543019_Zymomonas_mobili           MDAGVPLTRPVSGIAMGLILEKFAILSDIMGDEDHLGDMDFKVAGTEKGI
49176320_Escherichia_coli           MDAGVPIKAAVAGIAMGLVKEGYVVLSDILGDEDHLGDMDFKVAGSRDGI
55981108_Thermus_thermo             MDAGVPIRAPVAGVAMGLVWEEAVILTDILGLEDALGDMDFKVAGTRKGV
30248196_Nitrosomonas_europa        MDAGVPLKAHVAGIAMGLIKEGFAVLTDILGDEDHLGDMDFKVAGTEHGI
19712941_Fusobacterium_nuclea       MSAGVPIKEHVAGIAMGLIKEGFTVLTDIMGLEDHLGDMDFKVAGTKSGI
48784235_Burkholderia_fungor        MDAGVPMKAHVAGIAMGLILEGFAVLTDILGDEDHLGDMDFKVAGTEQGV
45658536_Leptospira_interr          MAAGVPIQGSVSGIAMGLFSDSYAVLSDIAGLEDHFGDMDCKIAGTRKGI
15644097_Thermotoga_mariti          MDAGVPIKKHVAGIAMGLILEEEIILTDIIGMEDHYGDMDFKVAGTRDGI
56751675_Synechococcus_elonga       MDAGVPIKKPVSGAAMGLIREGYRVLTDIQGIEDFLGDMDFKVAGTDQGI
6459851_Deinococcus_radiod          MDAGVPLKAPVAGVAMGLVMEGYRVLTDILGLEDALGDMDFKVCGTAEGV
46106240_Rubrobacter_xylano         MDGGVPIKAPVAGVAMGLVKEGYVILTDIQGLEDHMGDMDFKVAGTRDGI
15073173_Sinorhizobium_melilo       MDAGVPLAKPVAGIAMGLIKEDFAVLSDILGDEDHLGDMDFKVAGTDAGI
48766529_Rhodospirillum_rubrum      MDAGVPLKNAVAGIAMGLIKEDFAVLSDILGDEDHLGDMDFKVAGTANGI
45509695_Anabaena_variab            MDAGVPILKPVSGAAMGLIKEGVRVLTDIQGIEDFLGDMDFKVAGTDAGI
17133532_Nostoc_sp.                 MDAGVPILKPVSGAAMGLIKEGVRVLTDIQGIEDFLGDMDFKVAGTDAGI
3323203_Treponema_pallid            LHAGVPIKKPVAGIAMGLITDGYAILSDILGEEDHLGDMDFKVAGTCDGI
48894082_Trichodesmium_erythr       MDAGVPIVKPVSGAAMGLIKEDVRILTDIQGIEDFLGDMDFKVAGTDSGI
53715606_Bacteroides_fragil         MDAGVKIKKPVSGIAMGLIKNAYAVLSDILGDEDHLGDMDFKVTGTKDGI
15605776_Aquifex_aeolic             FDAGVPMKKHVAGIAMGLILEKYVILSDILGDEDHLGDMDFKVAGTKDGI
21674467_Chlorobium_tepidu          MDGGIPLKKPVSGIAMGLIKEGYAVLSDILGNEDHLGDMDFKVAGTRDGI
32141272_Streptomyces_coelic        LNAGVPLKAPVAGIAMGLISQEYVALTDILGAEDAFGDMDFKVAGTKEFV
15827380_Mycobacterium_leprae       LNAGVPLKAPVAGIAMGLVSDDFVTLTDILGAEDAFGDMDFKCAGTKDFV
24943088_Homo_sapien                MDSGVPISSAVAGVAIGLVTKTYRLLTDILGIEDYNGDMDFKIAGTNKGI
24651641_Drosophila_melano          MDAGVPVSAPAAGVAIGLVTKFYRILTDILGIEDYMGDMDMKVAGTRKGF
50910551_Oryza_sativa               MDAGIPVREHVAGVSVGLVSEVYRILTDILGLEDHLGDMDFKIAGTRRGI
                                      .*: :    :* ::*:.      *:** * **  **** *  *    .

46446277_Protochlamydia_amoebo      TAFQMDIKVEGITIEIMRVALKQAKEGRVHILNKMLAVCPTYKGEMSRYA
15605577_Chlamydia_tracho           TAFQMDIKIEGITHKIMEQALAQAKQGRSHILNLMTQVLASPKGTVSKYA
29840519_Chlamydophila_caviae       TAFQMDIKVEGITPTIMQAALAQAKAGRQHILDIMKEALAAPKTDLSQYA
50934417_Oryza_sativa               SAFQMDIKVVGITLPIMEHALLQARDGRKHILNEMSKCSPPPAKVLSPYA
6006870_Arabidopsis_thalia          TAFQMDIKVGGITLEIMEKALIQAKAGRRHILAEMAKCSPPPTLSLSKYA
CM1081_Cyanidioschyzon_merolae      TALQMDIKVEGITLGIIREALEQARDARIHMLDVMDAACSAPRSTVPSSV
16803371_Listeria_monocy            TALQMDIKIDGLSRQILDEALTQAKEGRLHILEHLTSTISAPREELSAYA
42523058_Bdellovibrio_bacter        TALQMDIKIDSVSFEVMEQALAQAKEGRAHILNEMEKVMKVPRGQISEFA
51892669_Symbiobacterium_therm      TALQMDMKISGTSREILEKALMQAREGRMFIMEKMLACISEPRKELSPWA
16078732_Bacillus_subtil            TALQMDIKIEGLSREILEEALQQAKKGRMEILNSMLATLSESRKELSRYA
56543019_Zymomonas_mobili           TSLQMDIKVAGITEEIMQKALEQAKGGRAHILGEMSKALGEVRSEISNLA
49176320_Escherichia_coli           SALQMDIKIEGITKEIMQVALNQAKGARLHILGVMEQAINAPRGDISEFA
55981108_Thermus_thermo             TALQMDNKVGGLPREVLKEALLQAREARLKILDLMEAVLPAPRPELKPFA
30248196_Nitrosomonas_europa        TALQMDIKIQGITKEIMQVALLQAKEGRLHILEIMKQSLPIARESISVHA
19712941_Fusobacterium_nuclea       TALQMDIKITGITEEIMRIALNQAHQARIQILELMNNTISKP-AELKSNV
48784235_Burkholderia_fungor        TALQMDIKIQGITREIMQVALAQAKEGRLHILGKMTSAVSGANTVLSDYA
45658536_Leptospira_interr          TAFQMDLKVTGVSFDVLESVFEQAQRGRFHILDIMEKHISKASSTLAGTA
15644097_Thermotoga_mariti          TAFQMDCKVSGVSDELLMKALMQAREARMYILDRMYETISAPRPHLSKYA
56751675_Synechococcus_elonga       TALQMDMKIHGLPLEIIADAINQAKPARLHILNKMLEAIATPRADLSTYA
6459851_Deinococcus_radiod          TALQMDIKVGGITPQIMREALAQAKEGRLHILGKMAEVLAAPRAELSPTA
46106240_Rubrobacter_xylano         TALQMDMKITGVSAELLKEALEQARRGRLQILDIMREEIAEPRPEISDHA
15073173_Sinorhizobium_melilo       TSLQMDIKIEGITEEIMGVALNQAKGGRLHILGEMAKAISESRGQLGEFA
48766529_Rhodospirillum_rubrum      TSLQMDIKITSITKEIMNVALNQAKDGRIHILGEMTKALGNARSDVSDFA
45509695_Anabaena_variab            TALQMDMKISGLSLEVIAQAIHQAKDARLHILDKMLQTIDTPRTETSPYA
17133532_Nostoc_sp.                 TALQMDMKISGLSLEVIAQAIHQAKDARLHILDKMLQTIDQPRTETSPYA
3323203_Treponema_pallid            TGFQMDVKVEAVSASLMKEALQQARVGRLHILSVMNQTISAPSVHISRYA
48894082_Trichodesmium_erythr       TALQMDMKITGLPIEVIADAIHQAKPARIHILDKMLSVLDLPRPDMSPYA
53715606_Bacteroides_fragil         TATQMDIKVDGLSYEILERALNQAKEGRMHILGKIEETISEPRTELKDHA
15605776_Aquifex_aeolic             TSVQMDIKVKGITKEIMLDALKQAREGRLYILEKMYEAIPEPRKEPHPYT
```

```
21674467_Chlorobium_tepidu          TACQMDIKIDGLDYHILETALEQARKGRLHILDVMAEAIPESRADIGKYA
32141272_Streptomyces_coelic        TALQLDTKLDGIPASVLAAALKQARDARLHILDVMMEAIDTP-DEMSPNA
15827380_Mycobacterium_leprae       TALQLDTKLDGIPSQVLAGALAQAKDARLTILEVMAEAIDRP-DEMSPYA
24943088_Homo_sapien                TALQADIKLPGIPIKIVMEAIQQASVAKKEILQIMNKTISKPRASRKENG
24651641_Drosophila_melano          TAIQADLKIPGIPLKVVMESLQKATDAKSNILDIMSEAIREPRKYPKESW
50910551_Oryza_sativa               TAIQLDIKPAGIPLDIICESLEPARKARNQILDRMDQEISSARAFNDGSS
                                    :. * * *  .    ::   :  *  .:  ::  :

46446277_Protochlamydia_amoebo      PRIETIQIKPSKIAVVIKQIRAIIEQTGVQIDIDGLVNIAAIDLVSIEKA
15605577_Chlamydia_tracho           PRIETMQINTSKIATVIKQIRQIIERSGAQVDINGVINIAASTQESINKA
29840519_Chlamydophila_caviae       PRIETMQIKPNKIATVIKQIRQIIEEAGVQIDINGLVSISASSPQAIEKA
50934417_Oryza_sativa               PLIHVMKVKPNKVNLIIKTIKSIIEETGVDIDTGGTVKITARDLSSLEKS
6006870_Arabidopsis_thalia          PLILIMKVHPSKVYSLIKKVKSIIEESGVEIDMQGTVKIMAIDVASLERA
CM1081_Cyanidioschyzon_merolae      PKIKIINIDPKRIGDVIRVVRSIIEACGGEIDIEGVVSILGPETELLERA
16803371_Listeria_monocy            PKIITLNIKPEKIKDVIKQINAIIDETGVKIDIEGTVYIASQDQAMNRKA
42523058_Bdellovibrio_bacter        PRIETIKIKPDKIREVIKVIRGITEATGVKIEIQGTINIASADPEATKKA
51892669_Symbiobacterium_therm      PRIITMKISPEKIRDVISTINKIIEETKVEIDIQGTIYIAAVNLEAGERA
16078732_Bacillus_subtil            PKILTMTINPDKIRDVIKQINKIIEETGVKIDIEGTIFISSTDESGNQKA
56543019_Zymomonas_mobili           PRIETMSVPKDKIRDVIKVIREIVATTGAKVDIEGTVRLSSSDPANIEAA
49176320_Escherichia_coli           PRIHTIKINPDKIKDVISVIRALTEETGTTIEIEGTVKIAATDGEKAKHA
55981108_Thermus_thermo             PRILSLKVPVEKIGLVIKNVRALEE-LGVEVDIEGTVRIYSSDLQAALEA
30248196_Nitrosomonas_europa        PRIIKFKINPEKIRDVIAVIRALTEETGTTIDISGSVTIACVSSEGGEQA
19712941_Fusobacterium_nuclea       PRIQQITIPKDKIAALIKNIKGIIEQTGATVDITGLVSVFAKDADTLEKT
48784235_Burkholderia_fungor        PRMITIKINPEKIRDVISVIRALTEETGTTIDISGVVTIASTSSEGMAEA
45658536_Leptospira_interr          PRIIVRNIPKDRIGELIKNVRGISELTGAELYIEGKVTISGSNQESAEKA
15644097_Thermotoga_mariti          PIIKVTKVDPEKVADVIRVIKKIIKDFDVKVEIDGLVKVVGSSEENVDKA
56751675_Synechococcus_elonga       PRLFRIQINPEQIGLVIKTIRSITEQTGAKIDIEGAVTISAVDADSALRA
6459851_Deinococcus_radiod          PHILSLKINPELIGKVIKQVRELEA-MGAQVTIEGTVRIFSASGESAEAV
46106240_Rubrobacter_xylano         PRVEVIQIPTDKIGLLIKTINALQDEYGVNISVEGTVYVAGVEGMSVKAA
15073173_Sinorhizobium_melilo       PRIEVMNIPVDKIREVIKVIREIVEKTGAKINIDGTVKIASASAKEIEAA
48766529_Rhodospirillum_rubrum      PRITTIKVPPQKVREVIKVIREITEVTGTKIDIEGTIKIASADAEATQRA
45509695_Anabaena_variab            PRLLTIKIDPDMIGLVIKTIKGITEETGAKIDIEGTVTISAVDENKAKRA
17133532_Nostoc_sp.                 PRLLTIKIDPDMIGLVIKTIKGITEETGAKIDIEGTVTISAVDENKAKRA
3323203_Treponema_pallid            PHIESFKIAVEKIGALIKTVKSLSDQYRVTINTDGTVTVSGRDAQSVFDA
48894082_Trichodesmium_erythr       PRLLSFKIDPEMIGLVIKTIKGITEETGVKIDIDGTVTIAAADGEKAKQA
53715606_Bacteroides_fragil         PRIETMTIPKEFIGAVIKIIQGMQEETGATITIEGRIEISGTNKKSIDDA
15605776_Aquifex_aeolic             PKVEVVDVPEEKAPLIISTVKKIYDETGVKVWVGGKVYLFVFPGGDVEKA
21674467_Chlorobium_tepidu          PRLTTIQIPVDAIGMVIETIRSITEETGAEINIDGTVTIACSSPEATKAA
32141272_Streptomyces_coelic        PRIITVKIPVDKIGEVIKMINQIQEDTGADITIEGTIYIGAAQGSQAEAA
15827380_Mycobacterium_leprae       PRVITIKVPVDKIGEVIKVINAITEETGAQISIEGTVFVGATDGLSAQAA
24943088_Homo_sapien                PVVETVQVPLSKRAKFVYNLKKLQAETGVTISQVETFSVFAPTPSVMHEA
24651641_Drosophila_melano          PVSETLTVEPQQRAQLILHMKRIYLETGTSLTAVTHFNVFAPSQAAMDEA
50910551_Oryza_sativa               PRLATLSFSSDSLRKLLFHRKKIEQETGARVSVSGTVTIVAKTQPIMDKA
                                    *      .  .    .:   . :       :     . :

46446277_Protochlamydia_amoebo      KAIIHGLTIEIGRIYSGKAISIAPFGVFVEILPGKEGLCHIVKQGDMLMV
15605577_Chlamydia_tracho           KELIEGLTVEVGKVYNGRVTSIATFGVFVEVLPGKEGLCHIVKEGDKLAV
29840519_Chlamydophila_caviae       KSMIEGLVVEVGKIYEGRVTSIVPFGAFVEILPGKEGLCHIVKQGDILAV
50934417_Oryza_sativa               KAIIANLTPKVGEIYNCEIKTIAPYGAFVEIAPGREGLCHIFKVGDRIDV
6006870_Arabidopsis_thalia          KAIISGLTPSVGDIYNCEIKSMAPYGAFVEIAPGREGLCHIYKVGDRIDV
CM1081_Cyanidioschyzon_merolae      ARMIRNITVEVSQVFQARVTKVLPFGAYVELDPGKEGWLHILREDQVIAV
16803371_Listeria_monocy            IAIIEDIVVEVGEVYTGKVRRIEKFGAFVELFKGTDGLVHILKLGDEVTV
42523058_Bdellovibrio_bacter        IAMINDIIAEVGKTYKGRIVKIAEFGAFVEILPNTQGLLHILKEGEIIDV
51892669_Symbiobacterium_therm      RQMIEALVPEPGMIYTGRVTRLMQFGAFVEILPGKEGLVHIVNIGDEVTV
16078732_Bacillus_subtil            KKIIEDLVVEVGQLYLGKVKRIEKFGAFVEIFSGKDGLVHIVKIGDEILV
56543019_Zymomonas_mobili           REWINGIVPEVGKIYNGKVVNIVDFGAFVNFMGGRDGLVHVLSEGQEVKV
49176320_Escherichia_coli           IRRIEEITIEVGRVYTGKVTRIVDFGAFVAIGGGKEGLVHILQMGQEVPV
55981108_Thermus_thermo             KKRIEDLTAKVGEIYEGTVTRITPFGAFISLFPGTEGLLHILKVGDVIKV
30248196_Nitrosomonas_europa        RKRIEDITVEVGRIYEGTVLKLLDFGAIVSVLPGKDGLLHILKEGQTVRV
19712941_Fusobacterium_nuclea       LKLVDSFVVEYNEVYEGRVVSIMKFGAFMEILPGKEGLLHILSVGDVFKV
48784235_Burkholderia_fungor        KKRIENITVEVGQVYEGTVLKLLDFGAIVNILPGKDGLLHILKDGQQVKV
45658536_Leptospira_interr          AKMVDGFFVEVGKIYEGKVKRIADFGAFVEILPGKEGLCHIVKEGDIIRV
15644097_Thermotoga_mariti          IELIREIAIEVGEVLEGKVTRIEPYGLFIEVRPGKIGLLHQVKVGDTIKV
56751675_Synechococcus_elonga       KSIIEGMTITAGDVYIGKVTRIIPIGAFVEFLPGKEGMIHILTVGDEVVV
6459851_Deinococcus_radiod          KARIEAVTAKVGEEFEGTVVKIAPFGAFVNLFPGQDGMLHILTVGDKLKV
```

```
46106240_Rubrobacter_xylano         VSAIKGMTVEAGDIYVGKVVKTTNFGAFVELTPGRDGLLHILNEGDVVKV
15073173_Sinorhizobium_melilo       RKWIHSIVPEVGQVYEGTVVKTADFGAFVNFFGARDGLVHIVKEGDKVWV
48766529_Rhodospirillum_rubrum      VDWIKGIVPEIGVVYTGKVVKIMDFGAFVNFLGTRDGLVHIVNVGDQVKV
45509695_Anabaena_variab            RNIVQGMTLNEGDVYAGRVTRIIPIGAFVEFLPGKEGMIHIVAVGDEVIV
17133532_Nostoc_sp.                 RNIVQGMTLNEGDVYAGRVTRIIPIGAFVEFLPGKEGMIHIVAVGDEVIV
3323203_Treponema_pallid            KVAVVGLTPRVGRVYQGVVKRIVEFGAFVEIFPGKEGLCHVLQEGQRICV
48894082_Trichodesmium_erythr       CNIIQGMTLNPGDVYVGRVTRIIPIGAFVEVFAGKEGMVHILAIGDEVIV
53715606_Bacteroides_fragil         IRLIKGIVPEVGEVYKGKVRSIMPYGAFVEFLPGKDGLLHIIKEGDEIEV
15605776_Aquifex_aeolic             KQMIQDIVVEVGAVYKGTITRVEPYGVFVELWPGKIGLLHVYKVGEEIIV
21674467_Chlorobium_tepidu          VETIKTLVPEVGTIYMGKVRDIRDLGAFVEFLPKTDGLVHILKVGDRIKV
32141272_Streptomyces_coelic        RATINGIAPEVGERYLGTVVKTTTFGAFVSLLPGKDGLLHILGVGAKVQV
15827380_Mycobacterium_leprae       INKINAIAPTVGERFLGTVVKITEFGAFVSLLPGRDGLVHIVNVGDKLRV
24943088_Homo_sapien                RDFITEICLEFGAVYTATITEIRDTGVMVKLYPMTAVLLHNLEVGQEIQV
24651641_Drosophila_melano          KELIEGYMLEFGGIYTAKITELRDTGVMVILYPMPPALLHNLEVGQEIQV
50910551_Oryza_sativa               IEKVEFLVIEVGRTYKGVVSSIKEYGAFVEFNGGQQGLLHIVSVGQVLSL
                                       :       .             *  : .        *    .  . :

46446277_Protochlamydia_amoebo      KVLDINERGQIKLSR
15605577_Chlamydia_tracho           KLLSINEKGQLKLSH
29840519_Chlamydophila_caviae       KLLSINEKGQYKLSH
50934417_Oryza_sativa               KLIEINDKGQLRLSS
6006870_Arabidopsis_thalia          KLIEVNEKGQLRLSV
CM1081_Cyanidioschyzon_merolae      KVLEVNARGQVRVSR
16803371_Listeria_monocy            KVIEVDQQGRVNLSR
42523058_Bdellovibrio_bacter        KVLEVDRSGRVKLSR
51892669_Symbiobacterium_therm      KVTEIDRLGRINLSI
16078732_Bacillus_subtil            KVTEIDKQGRVNLSR
56543019_Zymomonas_mobili           KVLEIDNRGKVRLSM
49176320_Escherichia_coli           KVLEVDRQGRIRLSI
55981108_Thermus_thermo             KVHRIDERGKIDLIR
30248196_Nitrosomonas_europa        KVLEADEKGRLRLSM
19712941_Fusobacterium_nuclea       RVISM-EGGKISLSK
48784235_Burkholderia_fungor        KVIQTDEKGRVRLSA
45658536_Leptospira_interr          KVLNVDKTGKIDLSR
15644097_Thermotoga_mariti          QVINIDDLGRLQFKR
56751675_Synechococcus_elonga       KVREIDQKGRVNLTR
6459851_Deinococcus_radiod          KIANIDDRGKIDLIR
46106240_Rubrobacter_xylano         RVLEIDKQNRISLEM
15073173_Sinorhizobium_melilo       KLMGFDERGKVRLSM
48766529_Rhodospirillum_rubrum      KCVGFDDRGKIKLSM
45509695_Anabaena_variab            KVREIDNKGRINLTR
17133532_Nostoc_sp.                 KVREIDNKGRINLTR
3323203_Treponema_pallid            KLIDIDRMGRLNLSY
48894082_Trichodesmium_erythr       KVREIDSKGRVNLTR
53715606_Bacteroides_fragil         KLIDIPKTGKFKLSR
15605776_Aquifex_aeolic             KVLDLDELGRPRFTT
21674467_Chlorobium_tepidu          KLIDVPRTGKFALSI
32141272_Streptomyces_coelic        EIAEIDSRGKLSLIP
15827380_Mycobacterium_leprae       EIADIEKRGKISLVL
24943088_Homo_sapien                KYFGRPADGRMRLSR
24651641_Drosophila_melano          KYFGRPVSGFMRLSR
50910551_Oryza_sativa               TCIGQDLRGNIKLSL
                                            .   .
```

```
46446277_Parachlamydia    FYLQYSFPPYSVGEVGRVGAPGRREIGHGKLAERALMAVIPTKEQFPYTIRLESNITESNG   442
15605577_Chlamydia        FYLQYFFPPFSVGEVGRIGSPGRREIGHGKLAEKALSHVLPETSRFPYIIRLESNITESNG   436
29840519_Chlamydophila    FYLQYFFPPFSVGEVGRIGSPGRREIGHGKLAEKALSHTLPDPAKFPYTIRIESNITESNG   435
50934417_Oryza            FYLQYTFPPSSVGEVGRIGAPNRREIGHGMLAERALEPILPPEEDFPYTIRVESTITESNG   570
6006870_Arabidopsis       FYLQYTFPPSSVGEVGRIGAPSRREIGHGTLAERALETILPSDDDFPYTIRVESTVIESNG   595
cm1081_Cyanidioschyzon    FYLQYMFPPFAVGEVGRIGAPGRREIGHGMLAQRALEPVVPPREVFPYVIRVESNIMESNG   555
16803371_Listeria         FMHHYNFPQFSVGETGPRRAPGRREIGHGALGERALQYVIPSEEEFPYTIRLVSEVLESNG   438
48833185_Magnetococcus    FYLNYTFPPYCVGETGRMGAPGRREIGHGKLATRALTAIVPSAEVFPYTLRITSEITESNG   439
42523058_Bdellovibrio     FMLHYNFPPYSVGEVGRMSGTSRREIGHGNLAERAIKAVLPDFEKFPYTIRIVSEVLESNG   439
1892669_Symbiobacterium   FMHHYNFPPYSVGETRPLRGPGRREIGHGALAERALEPVIPPEEEFPYTIRVVSEVLESNG   450
16078732_Bacillus         FMHHYNFPQFSVGETGPMRGPGRREIGHGALGERALEPVIPSEKDFPYTVRLVSEVLESNG   437
56543019_Zymomonas        FMLHYNFPPYSVGEVGRFGAPGRREIGHGKLAWRALHPVLPSKADFPYTIRVLSDITESNG   434
49176320_Escherichia      FLFHYNFPPYSVGETGMVGSPKRREIGHGRLAKRGVLAVMPDMDKFPYTVRVVSEITESNG   459
55981108_Thermus          FLVHYNFPPFSTGEVRRLRGVSRREVGHGNLAKRALKAVMPKEEDFPYTIRVVGDVLESNG   448
30248196_Nitrosomonas     FMLHYNMPPFATGETGRVGTPKRREIGHGRLAKRALLAVIPPVEEFGYSMRVVSEVTESNG   436
19712941_Fusobacterium    FYLHYNFPPYSVGEVGRMGSPGRRELGHGSLAERALSYVIPTEEEFPYTIRVVSEITESNG   449
56415214_Salmonella       FLFHYNFPPYSVGETGMVGSPKRREIGHGRLAKRGVLAVMPDMDKFPYTVRVVSEITESNG   436
48784235_Burkholderia     FMLHYNMPPFATGETGRVGSPKRREIGHGRLAKRALAACLPSADEFGYSIRVVSEITESNG   441
45658536_Leptospira       FMLHYNFPAFSVGEVRRSSGPGRREIGHGNLAERALKLVLPKPDEFPYVIRVVSEILESNG   435
15644097_Thermotoga       FMLHYNFPPFCTGEVKPLRGPSRREIGHGHLAERALKNMLPPEEEFPYTIRVVSEILESNG   438
56751675_Synechococcus    YLHHYNMPPYSVGETRPMRSPGRREIGHGALAERALLPVLPSKEEFPYVIRVVSEVLSSNG   445
6459851_Deinococcus       FLLHYNFPPYSTGEVKRMGGQSRREIGHGNLAKRAIRAVLPSFEEFPYVIRVVGDVLESNG   471
46106240_Rubrobacter      YMHHYYFPPYSTGETGRLGPPRRREIGHGALAERALLPVIPSEEEFPYAIRIISEVLESNG   416
8766529_Rhodospirillum    FMLHYNFPPYSVGEAGRMGSPGRREIGHGKLAWRAIHPMMPAKDAFPYTVRVVSEITESNG   436
23349057_Brucella         FMLHYNFPPYSVGETGRMGSPGRREIGHGKLAWRAIHPMLPAAEQFPYTIRAVSEITESNG   438
45509695_Anabaena         YLHHYNFPPFSVGETKPLRAPGRREIGHGALAERAILPVLPPKEQFPYVIRVVSEVLSSNG   446
17133532_Nostoc           YLHHYNFPPFSVGETKPLRAPGRREIGHGALAERAILPVLPPKEQFPYVIRVVSEVLSSNG   446
3323203_Treponema         FILHYNFPPFSVGEIGRMGV-GRREIGHGCLAHRSLSAVIPDPEQFPYTVRVVSEILESNG   434
48894082_Trichodesmium    YLHHYNFPPFSVGETKPLRQPGRREIGHGALAERALIPVIPKSDIFPYVIRVVSEVLSSNG   446
53715606_Bacteroides      FLLHYNFPPFSTGEAKAQRGVGRREIGHGNLAHRALKRMIP--EDYPYVVRVVSDILESNG   438
15605776_Aquifex          FMLHYSMPPFSVGEAKPWGPPRRREIGHGALAERAIEPLLPPEEEYPFIIRVVSDILESNG   435
21674467_Chlorobium       FMLHYNFPPFSVGEIGRVGGAGRREIGHGNLAERAIKMVMPSEQEFPYTVRLVSDILESNG   453
32141272_Streptomyces     YMHNYNFPPYSVGETGRVGSPKRREIGHGALAERAIVPVLPTREEFPYAIRQVSEALGSNG   460
6968686_Campylobacter     FMVNYNFPGFSVGEASPIKAPGRRELGHGNLAKRALYPSV--DENYPYVIRLVSEILESNG   457
15827380_Mycobacterium    YMHHYNFPPFSTGETGRVGSPKRREIGHGALAERALVPVLPSVEEFPYAIRQVSEALGSNG   474
24943088_Homo             FMLHYEFPPYATNEIGKVTGLNRRELGHGALAEKALYPVIP--RDFPFTIRVTSEVLESNG   481
24651641_Drosophila       FMLHYEFPPYATGEVGRIGPVGRREMGHGALAERSLLPTLP--NDYPFTVRLTSEVLESNG   479
50910551_Oryza            FMLHYSFPPFSINEVAKRGGLNRREVGHGTLAEKALLAVLPPEGEFPYTVRVNSEVMASDG   500
```

Figure 3a. A segment of multiple protein sequence alignment of polynucleotide phosphorylase showing conserved residues shared by chlamydiae, red algae and green plants.

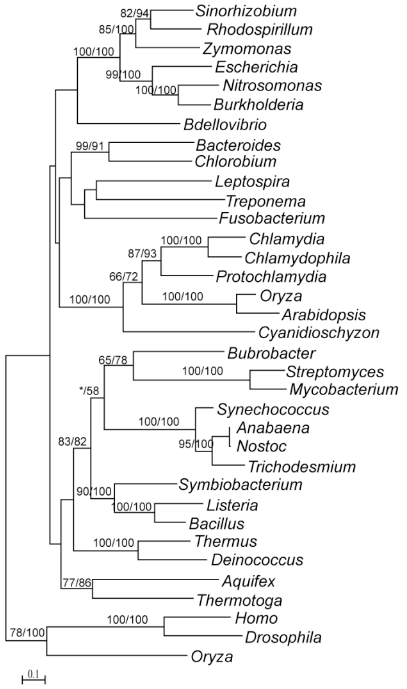

Figure 3b. Phylogeny of polynucleotide phosphorylase showing that sequences of chlamydiae, red algae and green plants form a monophyletic group that is distinct from those of cyanobacteria.

4. Aspartate transaminase

CLUSTAL X (1.83.1) multiple sequence alignment

```
19915595_Methanosarcina_acetiv      YSDRINALPPYLFAAIDEAKDEMIVIDLGVGDPDLPTHPHIVEAMREAVC
11498021_Archaeoglobus_fulgid       LADRLEKIPPYLFAEIDAMKRKKLVIDFGVGDPDLPTPEHIVEALKNAAE
15605813_Aquifex_aeolic             FSDRLKVLPPYLFAELDRKKQEKIVIDLGVGDPDMPTPKPIVEAAKKALE
45505954_Anabaena_variab            FAKRLEKIPPYLFAEINRKREALIIINIGVGDPDKPTPAHILQAMREAID
48894959_Trichodesmium_erythr       FAKKLEKISPYPLTEINRKKNELIIINFGAGDPDQPTPNHIVQAMHEAIK
67920389_Crocosphaera_watsonii      RADRLNALPPYVFARLDELKAKARLIDLGMGNPDGMAPQPVIDGAIAALQ
68270744_Moorella_thermo            EARRIRELPPYLFARIEKKIAEARIISLGIGDPDMPTPSHVIDKLVAEAH
57234493_Dehalococcoides_ethen      LSKRIENLPPYLFVQISKKIAEKRVISFAIGDPDLPTPKHILAELCKAAE
67917639_Chlorobium_limico          QFDKIKRLPKYVFAAVNELKMAERVIDFSMGNPDGPTPQHIVDKLVESIN
57168677_Campylobacter_coli         RFNTIERLPNYVFAEVNAIKMAARIIDFSMGNPDGKTPQHIIDKLCESAN
70731920_Pseudomonas_fluore         RFARIDRLPPYVFNITAELKMAARIIDLSMGNPDGPTPPHIVEKLVTVAQ
26248758_Escherichia_coli           RFTRIDRLPPYVFNITAELKMAARIIDFSMGNPDGATPPHIVEKLCTVAQ
68558669_Ralstonia_metall           RFARIDRLPPYVFNITAELKMAARIIDMSMGNPDGATPPHIVAKLVDAAQ
48765475_Rhodospirillum_rubrum      DFYRIKRLPPYVFAEVNAMKARARIIDLGMGNPDQPTPQHIVDKLCEAAT
68193651_Mesorhizobium_sp.          EFHKVRRLPPYVFEQVNRLKASARIIDLGMGNPDLPTPKAIVDKLCEVVR
45509383_Anabaena_variab            PADRIQQLPPYVFARLDELKAKARLIDLGMGNPDGATPQPVVDAAIQALQ
52079908_Bacillus_lichen            HSDVLKQLPEQFFASLVKKVNEKIVINLGQGNPDQPTPEHIVDTMAQAVR
89894681_Desulfitobacterium_ha      KARRLSSLGASVFTEMDDLRKELELINLSIGSPDRSPSAEIRKVLAEGVL
18893347_Pyrococcus_furios          LSDKLDLVNPSEIRKLFDLAAGMKVISLGIGEPDFDTPAHIKEYAKEGLD
53796147_Chloroflexus_aurant        IARRIRAVPPSGIRRFFDIAATMPVISLGVGEPDFITPDQIRAAGQASID
56784224_Oryza_sativa               ISPTVSALRPSKTMAITDQATALRVIGLAAGEPDFDTPHVIAEAGMNAIK
14601942_Aeropyrum_pernix           QERVVEIEGETAFAYLA-VARKLIVISFGIGQPDFPTPHHIREAAKKALD
CM2642_Cyanidioschyzon_merolae      NENFSKLQGGYLFPQIAQRRREYLLISLGIGDTTQPIPPHICAGLTQGAK
37703720_Oryza_sativa               NANMAKLQAGYLFPEIARRRAAHLIISLGIGDTTEPIPDVITNAMAERAH
21436149_Arabidopsis_thalia         NSNMSKLQAGYLFPEIARRRSAHLVISLGIGDTTEPIPEVITSAMAKKAH
37703722_Oryza_sativa               NPNMEKLQKGYLFPEISIKREEHLVISLGIGDTTEPIPSIVTSAMAEYAL
25315768_Arabidopsis_thalia         NVNLEKLKNNYLFPEINRRELEHILISLGTGDTTEPIPEQITSHMSNFAH
62184887_Chlamydophila_abortu       NTNFSNLEANYLFSGIRQKIQAFRIIDLSIGDTSYPLHTSVIHTFTQSVE
8163283_Chlamydia_muridarum         NPNFTSLTQNYLFSDLRNRIAQFHVINLSIGDTTQPLDTSVAEAFSQAIA
46399960_Protochlamydia_amoebo      NVHLTKLQSGYLFPEINRRKNEFLLINLGIGDTTQPIPLYISEAMQNFAK
48895399_Trichodesmium_erythr       NDNYLKLKAGYLFPEIARRVNTFAIIKLGIGDVTEPLPKACIQAMTKAVA
72382904_Prochlorococcus_marin      NADYLKLKAGYLFPEISRRITEFSLIRLGIGDVTEPLPLACREAMKAAIE
23124300_Nostoc_puncti              NNNYLKLKAGYLFPEISRRVNAFAIIRLGIGDVTEPLPEACRTAMIKAVE
67924273_Crocosphaera_watson        NDNYLKLKAGYLFPEIARRVNTFVIIKLGIGDVTEPLPEACRTAMIKAVE
56750696_Synechococcus_elonga       NDNYLKLKAGYLFPEIARRVNAFAIIRLGIGDVTEPLPVACRQAMIQAVE
53713935_Bacteroides_fragil         NEHFLKLPGSYLFSDIAKKVNTFKIIRLGIGDVTRPLPKACIEAMHKAVE
67916709_Clostridium_thermo         NENYLKLPGSYLFSEIARRVDNFRIIRLGIGDVTKPLAPAVIDALHKAVD
68004982_Geobacter_metall           NDNYLKLKAGYLFPEIGRRVREFSVIRLGIGDVTRPLAPAIIKAFHDAVD
45658671_Leptospira_interr          NENYLKLKAGYLFPEISKRVKIYSIIRLGIGDVTLPIVPSVVDAMVEASK
84489723_Methanosphaera_stadtm      NEYYSLIQNNYLFVEIARRVDEYQLIKMGIGDVTRPLAKSVVEAFKRAVD
2621088_Methanothermobacter_th      NENYLLLKSSYIFSEINRRVEEFQIIRMGIGDVTRPLPEAVVEAFHRAVD
89896148_Desulfitobacterium_ha      NENYLKLPGSYLFSEIARRVNEFKIIRLGIGDVTRPLAPVVVEAMKQAVE
                                                                :* :. *.

19915595_Methanosarcina_acetiv      DPKTYPSYAGMPEFREAAAEWCKKLDPATEVLSLIGSKEAVAHIPLAFNP
11498021_Archaeoglobus_fulgid       KVERYPSYEGMLSFRESVARFYRRLDPESEVISLIGSKEGIAHLPLAFND
15605813_Aquifex_aeolic             NPENYPSYVGKYEFRKAVADWYKRLDPNTEVITLIGSKEGIAHFPLAFNP
45505954_Anabaena_variab            EASNYPPYEGTQEFREAAVKWMERLNPNTEVVSSIGSKEAIHNTFLAFEA
48894959_Trichodesmium_erythr       DSTNYPPIKGLKEYRQAAADWMKRLNSETEVISSIGSKESIHNLFLAFNP
67920389_Crocosphaera_watsonii      KPQTYPPFEGTASFREAITAWYRRLDPSSEALPLIGSKEGLGHLALAYNP
68270744_Moorella_thermo            NPENYPTSEGLLAFRQAVADWYQRLDPRREVVTLIGSKEGIAHISLCYDP
57234493_Dehalococcoides_ethen      DPANYPETEGLPVLRKAMAEWYEKLNPDTEVLPLIGSKEGIGHAAWCFDP
67917639_Chlorobium_limico          KPRTYSVSKGIYKLRGAVGSWYRRLDLDREVVVTMGSKEGYVHLVQAINP
57168677_Campylobacter_coli         KDKTYSTSMGIYKLRLAICNWYKRLDPESEVVATMGSKEGFVNLARAINP
70731920_Pseudomonas_fluore         REDTYSTSKGIPRLRRAISRWYKDIDPESEAIVTIGSKEGLAHLMLATDQ
26248758_Escherichia_coli           RPDTYSTSRGIPRLRRAISRWYQDIDPESEAIVTIGSKEGLAHLMLATDH
68558669_Ralstonia_metall           RPDTYSASKGIPRLRRAISHWYREIDPDKEAIVTIGSKEGLAHLMLATDR
48765475_Rhodospirillum_rubrum      NPRAYSMSRGIPGLRKALAGYYARLDPETEAIVTLGSKEGLANLAQAISP
68193651_Mesorhizobium_sp.          DPRTYSSSRGIPGLRRAQAAYYARLNPDTQVVATLGSKEGFANMAQAIAP
```

```
45509383_Anabaena_variab           NPANYPPFEGTASFRRAITNWYNRLDPDSEALPLLGSKEGLSHLAIAYNP
52079908_Bacillus_lichen           NPENYSSFRGSRSLKEAAAAFYQRLDPEREVAVLFGGKAGLVELPQCLNP
89894681_Desulfitobacterium_ha     DGGSYTLTRGTESFRSGCARWYKELDPEKEVLPLMGSQDGLAHIFLALDP
18893347_Pyrococcus_furios         KGLTYGPNVGLPELREAIAEKLKKADPNSEIMVLVGANQAFLMSLATFKD
53796147_Chloroflexus_aurant       RTTAYTSNSGLLELRVALAEHLARYDPETELLITVGVSEAMLGAALALDP
56784224_Oryza_sativa              DGYTYTPNAGTLELRKAICNKLQEYSPD-QVLVSNGAKQCITQAVLAVSP
14601942_Aeropyrum_pernix          EGFTYTETAGIPELREAIAWYLNSVSPE-EVIATTGAKTAIFLGMALYRP
CM2642_Cyanidioschyzon_merolae     KLATYGDGEGLYALRKSIASRLYGIRPE-EVFVSDGAKCDIARLQMVFGS
37703720_Oryza_sativa              ALSTYGAEQGEKKLRAAIAATYYAIEET-DIFVSDGAKCDISRLQVLFGS
21436149_Arabidopsis_thalia        ELSTYGAEQGAKPLRAAIAKTFYGIGDD-DVFVSDGAKCDISRLQVMFGS
37703722_Oryza_sativa              ALSTYGPEQGHKNLRKEIADKVYPIKES-EVFISDGAQCDIARLQTLFGP
25315768_Arabidopsis_thalia        GLSTYGLEQGNKTLRKAIAETFYRVKSN-EVFVSDGAQSDISRLQLLLGS
62184887_Chlamydophila_abortu      KLGNYGPELGLPALREKLSEVFYHVSPE-EIFISEGAKMDIFRIFSLFGP
8163283_Chlamydia_muridarum        RFSSYGPDFGLPSLRQKLSEKLYHVNAE-EIFISDGAKVDLFRLLSFFGP
46399960_Protochlamydia_amoebo     QLASYGTEQGSILLREAIAEQYYQIDPQ-EVFVSDGSKCDVGRLQILFGS
48895399_Trichodesmium_erythr      EMGDYGPEQGYAWLREKIAAQDFQIDAS-EIFISDGSKCDSGNILDIIGN
72382904_Prochlorococcus_marin     EMGTYGPEQGYKWLREIISENDYIISAE-EIFVSDGSKCDSSNILDILGK
23124300_Nostoc_puncti             DMGDYGPEQGYAWLREKIAAQDFQIDAS-EIFISDGSKCDTGNILEIFGH
67924273_Crocosphaera_watson       DMGDYGPEQGYGWLRETIAAQDFQIDGS-EIFVSDGAKCDSGNILDIFGK
56750696_Synechococcus_elonga      DMGQYGPEQGYAWLREKIAAHDFQVDAS-EIFISDGSKCDCGNILDIFGN
53713935_Bacteroides_fragil        EMTSYGPEQGYDFLIEAIIKNDYALSPT-EVFVNDGAKSDTGNIGDILRH
67916709_Clostridium_thermo        EMAKYGPEQGYSFLVSKIIEYDYMLDED-EVFVSDGAKSDTGNFQEIFGL
68004982_Geobacter_metall          DLATYGPEQGYDWLINAIIEKSYKLKTE-EMFISDGSKCDCANILDIFAL
45658671_Leptospira_interr         EMGTYGPEQGYSFLLKSIADHDYGIDES-EIFVSDGSKCDCGNIQEIFST
84489723_Methanosphaera_stadtm     ELGDYGPEQGYDFLIEDVIENDYKLKNS-EVFISDGAKCDTGNFQELFSK
2621088_Methanothermobacter_th     EMAEYGPEQGYPFLREAIAENDYAITAD-EIFISDGAKCDTGNIQEIFGL
89896148_Desulfitobacterium_ha     EMGRYGPEQGYDFLIEKIIANDYALGMD-EVFVSDGAKSDTANFQEIFGV
                                       *    *                   :     * .

19915595_Methanosarcina_acetiv     GDVVLYTDPGYPVYKIGTLFAGGEPYSLPLKAENSFLPDLDSKRAKLFFF
11498021_Archaeoglobus_fulgid      GDYVLVPEPGYPVYYSSTLLADGVPYEMPLKEENKFLPDFQLRKAKIMFL
15605813_Aquifex_aeolic            GDIVLCPDPAYPVYRIGAIFAGGTPYTVPLKEENNFLPDLDSKKAKIIWI
45505954_Anabaena_variab           GDYTLIPDPGYPVYRTSTIFAGGEPFTMPLKAENKFLPDLDLRKAKMLWV
48894959_Trichodesmium_erythr      GDYAIIPDPGYPVYRTSTIFTLGQPYSMQLRAENGFLPILSNEKAKLLWI
67920389_Crocosphaera_watsonii     GDVVLVPSPSYPAHFRGPLIAGADLYPLILSAEKDWLIDLNSKAAKILYF
68270744_Moorella_thermo           GDINLVPDPGYPVYNIGTLLAGGESYFMPLTAANGFLPDLGARRAKLMFI
57234493_Dehalococcoides_ethen     GDVALVPDPAYPVYAISSQLAGAEVFYMPLNKENNFLPDFNASKAKILWI
67917639_Chlorobium_limico         GDLAMVPDPCYPIHSQAFILAGGNVHRLKLEDEDAFFNNIETPKPKYLVV
57168677_Campylobacter_coli        GDVAIVPAPAYPIHTQAFIIAGGNVAKMPLKDENKFFEDLDKPRPKYVVV
70731920_Pseudomonas_fluore        GDTVLVPNPSYPIHIYGAVIAGAQVRSVPLVPGVDFFAELERPKPKMMIL
26248758_Escherichia_coli          GDTVLVPNPSYPIHIYGAVIAGAQVRSVPLVEGVDFFNELERPKPKMMIL
68558669_Ralstonia_metall          GDTVLVPDPSYPIHIYGAVIAGADIRSVPLTPGIDFFAELERPKPKMIVL
48765475_Rhodospirillum_rubrum     GDIVLAPNPSYPIHAFGFIIAGGAVRHIPAKPGPEFLEAMERPKPVAVLI
68193651_Mesorhizobium_sp.         GDVVLCPNPTYPIHAFGFIMSGGVIRSIPAEPDHNFIPVLERPKPLALIL
45509383_Anabaena_variab           GDVVLVPSPAYPAHFRGPVIAGGTVHSLILKPENDWLIDLTARKAKILYF
52079908_Bacillus_lichen           GDTVLVPDPGYPDYWSGVELARANMETMPLTADNQFLPDYSREKAKLMYL
89894681_Desulfitobacterium_ha     GDVALIPDPGYPIYTAGLVLAGGEKVALPLREENGFLPDLSAQQAKIMFL
18893347_Pyrococcus_furios         GEEVLIPSPMFVSYAPAVILAGGKPVEVPTYEDNEFRINVDDEKTRALII
53796147_Chloroflexus_aurant       DDEVLVPEPCFVAYPACVTFADARVVYVPTNVADGFQVSGAAPRTKAILI
56784224_Oryza_sativa              GDEVLIPAPYWVSYPEMATLAGATPVILPTSISENFLLRPELEKSRLLIL
14601942_Aeropyrum_pernix          GDEVIIPDPSYYAYAQVAKLFGARPVYVPMKPGLGFRFDIEGEKTRMIVV
CM2642_Cyanidioschyzon_merolae     EVTVAVQDPSYPVYVDTAVMTGAGIVYMRCDAANDFFPDLSKPRTDLIFF
37703720_Oryza_sativa              NVKIAVQDPSYPAYVDSSVIMGGNIEYMKCSPENGFFPDLSSPRTDIIFF
21436149_Arabidopsis_thalia        NVTIAVQDPSYPAYVDSSVIMGGNIEYMRCTPENGFFPDLSTGRTDIIFF
37703722_Oryza_sativa              NVTIAVQDPTFPGYVDNGVIMGAGIEYMRCAPENAFFPDLSRRRTDVIFF
25315768_Arabidopsis_thalia        NVTIAVQDPTFPAYIDSSVIIGQNVVYMPCGPNNSFFPDLAMPRTDVIFF
62184887_Chlamydophila_abortu      GKTIAVQDPSYPVYIDTALLTGRKIIKLPCTKETHFFPEIPQEAIDIFCI
8163283_Chlamydia_muridarum        NQIVAVQDPSYPAYIDIARLTGKEIVSLPCLQENDFLPVFPETHIDILCL
46399960_Protochlamydia_amoebo     DATIAVQNPTYPAYVDTGVINGQRITYMSCLPENNFFPDLANPKTDLIYF
48895399_Trichodesmium_erythr      NNTIAVTDPVYPVYVDTNVMAGEGLVYLPIRAENNFTAEIPTQKVDLIYL
72382904_Prochlorococcus_marin     ENKIAVTDPVYPVYVDTNVMTGKGLSYIPINSENGFEASIPKDKFDLIYL
23124300_Nostoc_puncti             DNLIAVTDPVYPVYVDTNVMVGEGLVYLPITADNNFTAEIPSKKVDLIYL
67924273_Crocosphaera_watson       NNKIAVTDPVYPVYVDTNVMAGDGLVYLPISADNNFLADIPTEKVDLIYL
56750696_Synechococcus_elonga      NNRIAVTDPVYPVYVDTNVMAGDGLVYLPISAENNFTAEIPSEKVDLIYL
53713935_Bacteroides_fragil        DNSVGVTDPIYPVYIDSNVMCGSNVTYMPCTAENHFIPAIPEKRIDIVYL
```

```
67916709_Clostridium_thermo         DNKVAVTDPVYPVYVDSNVMAGENITYLPCTAENNFIPELPKEKVDIIYL
68004982_Geobacter_metall           DNVVAIGDPVYPVYNDTNVMIGKGIVYMPCTEANGFIPSLPTEKVDIIYL
45658671_Leptospira_interr          DSKIAVADPVYPVYVDTNVMAGSNLIYMPATKENGFQPEIPKEKADIVYL
84489723_Methanosphaera_stadtm      DNIIAVTDPVYPVYVDSNVMAGENIVYLPATSENDFVPSLPSEKVDIIYL
2621088_Methanothermobacter_th      DNVVAVTDPVYPVYVESNVMAGSGLVYLPCTEENSFIPSLPEERVDLIYL
89896148_Desulfitobacterium_ha      DNIMAVTDPVYPVYVDSNVMAGGRIIYLPCTEEGDMKPELPTAPVDMIYL
                                            * :  :     :       :                   . .

19915595_Methanosarcina_acetiv      NYPNNPTSATADMKFFEKVVEFCKKNDIIAVHDNAYSQMVYAPSFLAAEG
11498021_Archaeoglobus_fulgid       NYPNNPTAAVAPKEFIKEAIDFCIDNKIILAHDAAYSEITFAPSFLEFED
15605813_Aquifex_aeolic             NYPNNPTSAPPTLEFYKKLVDWAKEYNVIIASDNAYSEIYTPPSILQVPG
45505954_Anabaena_variab            NYPNNPTGALATLEFFEELVAFCQQHSILLCHDHAYSEMAYPPSVLQIPG
48894959_Trichodesmium_erythr       NYPNNPTGATASLEFFEELVGFCKHYDILLCHDNAYSEIAYPPSILQVPN
67920389_Crocosphaera_watsonii      NYPNNPTTATAPRAFFEEIIKWATHYEIMLVHDLCYAELAFPTSLLEIPG
68270744_Moorella_thermo            NYPNNPTGAVADLKFFQEVVEFARSYDLIVCHDAAYSEITYAPSFLQAPG
57234493_Dehalococcoides_ethen      NYPNNPTGAVAGLDFFKEAAEFAAKHNLAVCHDGPYSEIAFPVSFLEADG
67917639_Chlorobium_limico          NFPNNPTTATVDISFYERLVELARKERFYIISDIAYAEITFTPSVLQVPG
57168677_Campylobacter_coli         NFPHNPTTVTCEKSFYERLVATAKKERFYIISDIAYADLTYTPSILEVEG
70731920_Pseudomonas_fluore         GFPSNPTAQCVELDFFERVIALAKQYDVLVIHDLAYADIVYAPSIMQVPG
26248758_Escherichia_coli           GFPSNPTAQCVELEFFEKVVALAKRYDVLVVHDLAYADIVYAPSIMQVPG
68558669_Ralstonia_metall           GFPSNPTAACVELDFFERVIALARKHDIFVVHDLAYADIVFAPSIMQVPG
48765475_Rhodospirillum_rubrum      NFPSNPTAYVADLDFYGQVVDFCRHHGIWILSDIAYSEIYFPPSVLQIPG
68193651_Mesorhizobium_sp.          NYPSNPTACLASLDFYKDVVAFARKHDIIILSDLAYAEIYFPPSVLQVPG
45509383_Anabaena_variab            NYPSNPTGATAPREFFEEIVAFARKYEILLVHDLCYAELAFPTSLLEIPG
52079908_Bacillus_lichen            NYPNNPTGAQATSAFFEETVRFAKSNGICVVHDFAYGAIGYPVSFLETAG
89894681_Desulfitobacterium_ha      NYPNNPTAAVAPLSFFEEVVDFARKNRIVVCHDAAYSELAFPVSFLQVPG
18893347_Pyrococcus_furios          NSPNNPTGAVLTKKDLEEIADFANEHDLMIISDEVYEHFIYHYSIAALDG
53796147_Chloroflexus_aurant        GYPNNPTGAVLSRERLLEVAAVAERHDLIVISDEIYDRLVYHTCFAALPG
56784224_Oryza_sativa               CSPSNPTGSVYPKELLEEIADIVKKYRLLVLSDEIYEHIIYHTSFASLPG
14601942_Aeropyrum_pernix           NNPHNPTGSVFPPDQVEAIHDIARRRGLIILADEIYDNFLYFKSTLSLPD
CM2642_Cyanidioschyzon_merolae      CSPNNPTGAAATREQLAELVAFARHNGSIIVYDAAYAPFIRPRSILEIDG
37703720_Oryza_sativa               CSPNNPTGAAASRDQLTKLVKFAKDNGSIIVYDSAYAMYISPKSIFEIPG
21436149_Arabidopsis_thalia         CSPNNPTGAAATREQLTQLVEFAKKNGSIIVYDSAYAMYMSPRSIFEIPG
37703722_Oryza_sativa               CSPNNPTGHAASREQLRQLVELARRNGSIIVFDSAYSSYISPRSIYEIPG
25315768_Arabidopsis_thalia         CSPNNPTGYVASRKQLHQLVDFAKTNGSIIIFDSAYAAFIEPRSIYEIPG
62184887_Chlamydophila_abortu       CSPNNPTGTVLNREQLKELVDYANAQGSIILFDAAYSAFISPTSIFDIPG
8163283_Chlamydia_muridarum         CSPNNPTGTALNKNQLRAIVRYAIEHNILILFDAAYSAFISPKSIFEIPD
46399960_Protochlamydia_amoebo      CSPNNPTGSAATNEQLRELVQFAKKRQSIIIFDAAYASFVRPRSIYEIEG
48895399_Trichodesmium_erythr       CFPNNPTGATATKEYLQTWVNYARANDAIILFDAAYEAFITPHSIYEIEG
72382904_Prochlorococcus_marin      CFPNNPTGAVATKEQLVSWVKYAKENNSLILFDAAYEAFIKPHSIFEIEG
23124300_Nostoc_puncti              CFPNNPTGATATKEYLKAWVDYAKANNSIIFFDAAYEAYITPHSIYEIEG
67924273_Crocosphaera_watson        CFPNNPTGATATKEYLKAWVDYAKANNAIIFFDAAYEAFITPHSIYEIEG
56750696_Synechococcus_elonga       CFPNNPTGAVASREYLQAWVDYARANGAIILFDAAYEAFITPHSIFEIPG
53713935_Bacteroides_fragil         CYPNNPTGTTLTKAELKKWVDYALANDTLILFDAAYEAYIRPHSIYEIKG
67916709_Clostridium_thermo         CFPNNPTGMTLSREELKKWVDYARENRAIILFDSAYEAYIRPHSIYEVEG
68004982_Geobacter_metall           CFPNNPTGTVATKAELKKWVDYANANDAVIFFDAAYEAFITPHSIYEIEG
45658671_Leptospira_interr          CYPNNPTGTVTTKESLKAWVEYAKKNNSIILYDSAYEAFISPRSIYEVEG
84489723_Methanosphaera_stadtm      CYPNNPTGTTLTKEQLKEWVDYAHENDALILFDAAYESFIKPHSIFEIEG
2621088_Methanothermobacter_th      CYPNNPTGTTLTEKQLAEWVDYARDSGSLILFDAAYEAYIQPHSIYEVEG
89896148_Desulfitobacterium_ha      CFPNNPTGMTLTKEELKVWVDYARENKAIILFDSAYEAFIRPRSIYEVEG
                                      * ***                         *  *       .     .

19915595_Methanosarcina_acetiv      AMDIGIELYSHSKTYNMTGWRLGFAVGSKALIKGLGKVKSNVDSGVFDAI
11498021_Archaeoglobus_fulgid       AFEVCVEFNSLSKTYNMTGWRIGFACGNRDILAGLLKVKTNVDSGVFEAI
15605813_Aquifex_aeolic             AKDVAIEFHSLSKTYNMTGWRIGMAVGNKELVAGLGKVKTNVDSGQFGAV
45505954_Anabaena_variab            AKDIAIEFHSLSKSYNMTGWRIGFAVGNAYAIQGLSQVKTNVDSGVFKAI
48894959_Trichodesmium_erythr       AKDIAIEFHSLSKTYNMSGWRIGFVAGNSLGIKGLSQVKSNIDSGVFQAI
67920389_Crocosphaera_watsonii      GREIGVEFHTLSKTYNMAGWRVGFVVGNGEIIQGLRTLKTNLDYGIFSAV
68270744_Moorella_thermo            AKEVGIEFNSVSKPYNMTGWRLGWACGRADVIEALARIKSNIDSGAFQAV
57234493_Dehalococcoides_ethen      AKEVGIEFHSLSKSYNMTGWRIGMAVGNAKMIDALRRFKSNLDSGIPQAI
67917639_Chlorobium_limico          AKDVAVESYTLSKTYNMAGWRVGFMVGNAKLIGALEKIKSWLDYGTFTPI
57168677_Campylobacter_coli         AKDVAVETYTLSKSYNMAGWRVGFTVGNKRLVAALKKIKSWFDYGMYTPI
70731920_Pseudomonas_fluore         AKDIAVEFFTLSKSYNMAGWRIGFMVGNPELVSALARIKSYHDYGTFTPL
26248758_Escherichia_coli           ARDMAVEFFTLSKSYNMAGWRIGFMVGNKTLVSALARIKSYHDYGTFTPL
68558669_Ralstonia_metall           AKDIAVEFFTLSKSYNMAGWRIGFMVGNPDLVAALTRIKSYHDYGTFTPL
```

```
48765475_Rhodospirillum_rubrum       AKEVAIEFTSLSKTYNMPGWRIGFAAGNRHLIGALTRIKSYLDYGAFTPV
68193651_Mesorhizobium_sp.           AMDVAVEFTSMSKTFSMPGWRMGFAVGNERLIAALSRVKSYLDYGAFTPI
45509383_Anabaena_variab             AKDIGVEFHTLSKTYNMAGWRVGFVVGNRHVIQGLRTLKTNLDYGIFAAL
52079908_Bacillus_lichen             AKDAGIEIYTLSKTYNMAGWRVGFAVGNASVIEALNLYQDHMYVSLFKAV
89894681_Desulfitobacterium_ha       AKEVGIEFHSVSKTYNLAGVRLGFAVGNREIIGALAELKSNIDYGVFEPA
18893347_Pyrococcus_furios           MFGRTITVNGFSKTFAMTGWRLGFVVAPSWVIEKMVKFQMYNATCPVTFI
53796147_Chloroflexus_aurant         MRERTVLLGGFSKAYAMTGWRLGWLAAPAEITAAVRKFHQYAIMSAPTMS
56784224_Oryza_sativa                MWDRTLTVNGFSKAFAMTGWRLGYLAAPKHFVAACGKIQSQFTSGASSIS
14601942_Aeropyrum_pernix            WRENLVYVNGFSKTFSMTGWRLGYVVLRREVIPKALDLAVTIYSCPPSIA
CM2642_Cyanidioschyzon_merolae       ALECAIEVNSFSKYAGFTGVRLGWTVVPSALAKDFGRVMNTAFNGASNIA
37703720_Oryza_sativa                AKEVAIETASFSKYAGFTGVRLGWTVVPKELAKDFNRIVCTCFNGASNIS
21436149_Arabidopsis_thalia          AEEVAMETASFSKYAGFTGVRLGWTVIPKKLAKDFNRIICTCFNGASNIS
37703722_Oryza_sativa                AREVAIEVSSFSKFAGFTGVRLGWAVVPDELARDFDRVVCTCFNGASGIA
25315768_Arabidopsis_thalia          AREVAIEVSSFSKFAGFTGVRLGWSIIPDELINDFHRIVTTSFNGASNIA
62184887_Chlamydophila_abortu        ARSCAIEVNSFSKSLGFAGVRLGWNVVPKDLIRDWERLLFTTFNGVCLPV
8163283_Chlamydia_muridarum          ARFCAIEINSFSKSLGFAGIRLGWTVIPKELIRDWKRFLSTTFNGASIPA
46399960_Protochlamydia_amoebo       AKEVAIEVGSFSKMIGFTGVRLGWSVVPKQLQQDWERIVCTFFNGASNIA
48895399_Trichodesmium_erythr        AKECAIEFRSFSKNAGFTGTRCALTVVPKTLWKLWNRRQSTKFNGVSYIV
72382904_Prochlorococcus_marin       ARDCAIEFRSFSKNAGFTGTRCAFTVIPKSLWSLWNRRQSTKFNGVSYVV
23124300_Nostoc_puncti               AREVAIEFRSFSKNAGFTGTRCALTVVPKTLWKLWNRRQSTKFNGVSYIV
67924273_Crocosphaera_watson         AKDCAIEFRSFSKNAGFTGTRCAFTVVPKQLWKLWNRRQSTKFNGVSYIV
56750696_Synechococcus_elonga        ARDCAIEFRSFSKNAGFTGTRCAFTVVPKGLWGLWNRRQSTKFNGVSYIV
53713935_Bacteroides_fragil          AKKCAIEFRSFSKTAGFTGVRCGYTVVPKELNRLWNRRQCTKFNGTSYIT
67916709_Clostridium_thermo          ADEVAIEFRSFSKTAGFTGTRCAYTVVPKKVNSLWNRRQTTKFNGVPYII
68004982_Geobacter_metall            AKKCAIEFRSFSKTAGFTGVRCGLVVVPEEVNKLWLRRTTTKFNGASYPV
45658671_Leptospira_interr           AKEVAIEFRSFSKTAGFTGLRCAYIVIPKELNSLWNRRHTTKFNGVSYVT
84489723_Methanosphaera_stadtm       AKDVAVEFRSYSKVAGFTGTRCAYCVVPEEVNPLWNRRQSTKFNGVSYPV
2621088_Methanothermobacter_th       AREVAIEFRSFSKNAGFTGTRCAFTVVPEELRELWNRRQTTKFNGVSYPV
89896148_Desulfitobacterium_ha       AREVAVEFRSFSKTAGFTGTRCAYTVVPKDINKLWLRRQTTKFNGVSYPV
                                          :     **   :.* * .

19915595_Methanosarcina_acetiv       QIAGIAALQACVDDTNKIYEERRNVLIEGLTAMGLEVKPPKATFYVWAPV
11498021_Archaeoglobus_fulgid        QEAAIAAMDRVIEENCKVYQRRRDLLVEGLRDVGIDAEKPKATFYVWAKV
15605813_Aquifex_aeolic              QDAGIVALEEEVEKIRDVYRERKKIMTEALEKIGLEIYRSDYTFYLWIKV
45505954_Anabaena_variab             QKAAIAAYEVELQAVMSVYQNRRDIIVKGLQSLGWPIEPPKATLYVWVPV
48894959_Trichodesmium_erythr        QKAATEAFEKHLQSLVAVYQKRRDILIKGLQSLGYPIKANLASLYAWVPV
67920389_Crocosphaera_watsonii       QAAAETALDSYIKTVQERYQTRRDFLIKGLGELGWNIKPSQATMYLWVPC
68270744_Moorella_thermo             QYAGIAALQEGLAEVRRVYQERRDIIVEGFNSLGWHLEKPKATFYVWAPV
57234493_Dehalococcoides_ethen       QLMAIAALQDVISQNCAVYQRRRDRLVEALRNIGMEVTAPKASLYIWAPV
67917639_Chlorobium_limico           QVASTIALQSCVAEICDVYRRRRDVMIKSFDNAGWPILPPRASMFVWARI
57168677_Campylobacter_coli          QVAATIALQSCVDEIRATYDKRMHILLEAFENAGWKLHKPRASMFVWAKL
70731920_Pseudomonas_fluore          QVAAIAALQQCVRDIAEQYRQRRNVLVKGLHELGWMVENPKASMYVWAKI
26248758_Escherichia_coli            QVAAIAALQQCVRDIAEQYKRRRDVLVKGLHEAGWMVEMPKASMYVWAKI
68558669_Ralstonia_metall            QVAAIAALQQCVKEIAAQYQSRRDVLARGLIESGWPVDIPKASMYIWARI
48765475_Rhodospirillum_rubrum       QVAATAALQDCVEDIRTLYRERRDVLIDGLAQAGWDIPSPPASMFAWAPL
68193651_Mesorhizobium_sp.           QVAATAALGTEIEEVRSVYHRRRDVMVDAFGRAGWQVPPPPATMFAWAPI
45509383_Anabaena_variab             QTAAETALDIYLHEVQQRYRTRRDFLIQGLGELGWDVPKTKATMYLWVKC
52079908_Bacillus_lichen             QDAAAAALQACVQEQNERYEKRRNAWIRAVRDIGWHADAPQGSFFAWMPV
89894681_Desulfitobacterium_ha       LQAGAYALQENVEQNRRAYQERRDIWVKGCAQAGWSMPSPRGSMFIWAPV
18893347_Pyrococcus_furios           QYAAAKALWKAVEEMRKEYERRRNLVWKRLNEMGLPTVEPKGAFYIFPRI
53796147_Chloroflexus_aurant         QYAGLEALEEAVQAMVREYDRRRKVIVSGLNSIGLPTFEPQGAFYAFPQV
56784224_Oryza_sativa                QKAGLAALGEAVSTMVKAFQERRDYLVKSFKELGVKISEPQGAFYLFIDF
14601942_Aeropyrum_pernix            QKAGVAALWGPVREMVEEFRSRARILYDILSQAGIEPYLPEGAFYMFPRV
CM2642_Cyanidioschyzon_merolae       QQGGMACLLAEIEQLISYYLENTRILREGMESLGFSVYGGRNAPYIWVRF
37703720_Oryza_sativa                QAGGLGCLLKAMSDVVGFYKENTKIIVDTFTSLGFNVYGAKNAPYVWVHF
21436149_Arabidopsis_thalia          QAGALACLLEAMHKVIGFYKENTNIIIDTFTSLGYDVYGGKNAPYVWVHF
37703722_Oryza_sativa                QAGGVACLRGAVARVVGVYRENARVLVETFRSLGKEVHGGGDAPYVWVRF
25315768_Arabidopsis_thalia          QAGGLACLLKEIRSVNNYYKENRKILMDTLVSLGLKVYGGVNAPYLWVHF
62184887_Chlamydophila_abortu        QESAIAGVLFPNLEAISHYRYNSSLLREALQKADFSVYGGEQAPYLWVEV
8163283_Chlamydia_muridarum          QEAATAGLL-SKLEAVHYYKENSHLLKTSLLKAGFQVFGGEHAPYLWVKP
46399960_Protochlamydia_amoebo       QAGGLAALLQAIDELSSYYMKNSNILKKAFEECGYKVYGGENVPYLWVHF
48895399_Trichodesmium_erythr        QRGAEAVYKAQVKGLIQFYLENAKIICSQLQAAGLTVYGGVNAPYVWVQT
72382904_Prochlorococcus_marin       QRGAEAVYKTQIKKLVSFYMDNAEIIKSNLTAAGFEVFGAVNAPYAWIKT
23124300_Nostoc_puncti               QRGAEAVYQAQIKGLVSFYLENAKIIREKLTAAGLSVYGGVNAPYVWVKT
67924273_Crocosphaera_watson         QRGAEAVYKAQIKALVNFYLENAQIICNKLTGAGFDVYGGVNAPYIWLKT
```

```
56750696_Synechococcus_elonga        QRGAEAVYQAQIKELVAFYLENARIIREELTAAGLDVHGGVNAPYVWVKT
53713935_Bacteroides_fragil          QRAAEAIYKEQIQETINYYMTNARIMKEGLESTGLKVYGGVNAPYLWVKT
67916709_Clostridium_thermo          QRAAAAVYQKQTKETIDYYMENAKIIKQGLEDIGLTVFGGVNAPYIWLKT
68004982_Geobacter_metall            QKAAAAVYWKQNKEIIDYYMENARIIREGLAAAGLTVYGGVNAPYIWLKT
45658671_Leptospira_interr           QKGAEACYKKEIQTSIAYYMANASKIRDGLKKAGYEVFGGVNAPYIWLKT
84489723_Methanosphaera_stadtm       QRAAQAIYKKEIQENLDYYVKNAQVIRESLEKMGLKVYGGVDSPYIWFKT
2621088_Methanothermobacter_th       QRAAEAVYQREIRESIDYYMENARIIRESLERAGLRYYGGVNAPYIWIRT
89896148_Desulfitobacterium_ha       QAGAAAVYKKQIQATIDYYMENARIIREGLQEAGFKVFGGVNAPYIWMKT
                                        .               :  .          .         : :

19915595_Methanosarcina_acetiv       SIEFAKLLLEEAGIVATPGVGFGDAGEGYVRFALTKPVERIKEAVER
11498021_Archaeoglobus_fulgid        SIEFVKQLIDKAGIVATPGIGFGKSGEGFVRFALTRGEGVIEEAIDR
15605813_Aquifex_aeolic              SAEFVGRLIDEAGIVCTPGNGFGEYGEGYFRISLTVPTERLLEAAER
45505954_Anabaena_variab             STEFTTLLLDKCGIVVPPGVGYGVSGEGYFRIALTICEERLHEAIQR
48894959_Trichodesmium_erythr        SQEFATLLLEKCGIIVVPGNAYGTSGEGFIRIALTVEDKRISQALQR
67920389_Crocosphaera_watsonii       STDFALDVLEKTGIVFTPGNAFGEGGEGYVRISLIADCDLLGEALNR
68270744_Moorella_thermo             SASFAEMVLEKAGVIITPGNGYGNYGEGYFRIALTISKERMQEAIER
57234493_Dehalococcoides_ethen       SASFATELLDKTGVVVTPGTGYGTSGEGYIRLSLTVPDEQLEKGIAK
67917639_Chlorobium_limico           SLEFSKKLLTEGKVAVSPGIGFGAYGDEYVRVAMIENEERIRQAARN
57168677_Campylobacter_coli          SLEFSKQLLQHAEVAVSPGIGFGEAGDEYVRIALIENENRIRQAARN
70731920_Pseudomonas_fluore          SLEFAKKLLAEAKVCVSPGVGFGEYGDDHVRFALIENQDRIRQAVRG
26248758_Escherichia_coli            SLEFAKKLLNEAKVCVSPGIGFGDYGDTHVRFALIENRDRIRQAIRG
68558669_Ralstonia_metall            SLEFAKQLLAKAKVSVSPGIGFGDYGDEYVRFALIENESRIRQAVRG
48765475_Rhodospirillum_rubrum       SLEFSKLLMREAKVAVSPGVGFGEYGDSHVRIALVENKHRIRQAVRN
68193651_Mesorhizobium_sp.           SLEFSKLLVEKADVAVAPGIGFGEHGDDYVRIALVENEHRIRQAARN
45509383_Anabaena_variab             STDFALNLLQQTGVVVTPGNAFGVAGEGYVRISLIADCDRLGEALGR
52079908_Bacillus_lichen             SEAFSDLLLEKANVVTAPGIGFGKHGEGYVRVGLLTSEERLREAASR
89894681_Desulfitobacterium_ha       SRSFAFALAREAGVIVVPGVAFGEYGEGYVRIGMVQDQEVLQEAVRR
18893347_Pyrococcus_furios           SKEFSELMLMEAKVAVVPGSAFGRAGEGYIRISYATAYEKLEEAMNR
53796147_Chloroflexus_aurant         SEAFCEQLLHSEQVAVVPGDAFGPSGAGFVRACYATALNKIEEALER
56784224_Oryza_sativa                SESLCMFLLEKAQVALVPGDAFG--DDKCIRMSYAAALSTLQTAMEK
14601942_Aeropyrum_pernix            VEQLAEKLLYSYGVLVLPGTSFPESGREHVRLSFATATSDVKEGAEI
CM2642_Cyanidioschyzon_merolae       SWDVFTEFLEKCQVVTVPGAGFGPAGVEYVRLSAFAPREACQEAVRR
37703720_Oryza_sativa                SWDVFAEILEKAHVVTTPGSGFGPGGEGFVRVSAFGHRENIIEAARR
21436149_Arabidopsis_thalia          SWDVFAEILEKTHVVTTPGSGFGPGGEGFVRVSAFGHRENILEACRR
37703722_Oryza_sativa                SWDVFAEILEKTHVITVPGSGFGPGGEGFIRVSAFNSRDKVLEACQR
25315768_Arabidopsis_thalia          SWDVFNEILENTHIITVPGSGFGPGGEEYLRISGFGRRDHIVEASKR
62184887_Chlamydophila_abortu        DEDVFDFFLHHYHIAITPGKGFGSCGKGYVRFSSLGKTEDIVAACQR
8163283_Chlamydia_muridarum          YRDLFDFFLQEYHIAITPGIGFGLCGSGFVRFSSLGKREDVLVACER
46399960_Protochlamydia_amoebo       SWEAFEILLKQSQLVSVPGSGFGSAGEGFLRFSAFGKQSDITVALPR
48895399_Trichodesmium_erythr        SWDFFDKLLQNCNVVGTPGSGFGAAGEGYFRISSFNSRENVNEAMKR
72382904_Prochlorococcus_marin       SWDFFDFLLEKANVVGTPGSGFGAAGEGYFRLSAFNSRENVEKAMQR
23124300_Nostoc_puncti               SWEFFDKLLQTVNVVGTPGSGFGAAGEGYFRISAFNSRENVEEAMKR
67924273_Crocosphaera_watson         SWDFFDKLLQTTNVVGTPGSGFGAAGEGYFRISAFNSRENVEEAMKR
56750696_Synechococcus_elonga        SWDFFDKLLQVCNVVGTPGSGFGAAGEGYFRISAFNSRENVVTAMQR
53713935_Bacteroides_fragil          SWRFFDQMLYEANVVGTPGVGFGPSGEGYIRLTAFGERDDCIEAMRR
67916709_Clostridium_thermo          SWEFFDIMLKEINVVGTPGSGFGPSGEGYFRLTAFGSRENTLEAVER
68004982_Geobacter_metall            SWDFFDKLLTECNVVGTPGSGFGPSGEGFFRLSAFGHRENVIEAVER
45658671_Leptospira_interr           SWDFFDKLLNKAQVVGTPGSGFGPAGEGYFRLSAFGKKEDVEEAIAR
84489723_Methanosphaera_stadtm       FFDILLKEAHVVSTPGAGFGPSGEGYLRLTAFNTYENTVEAMDRISK
2621088_Methanothermobacter_th       SWQFFDTLLNDAEVVGTPGSGFGPSGEGYFRLTAFNSFRNTVKAMER
89896148_Desulfitobacterium_ha       SWEFFDKLMTEAHVVGTPGAGFGANGEGFFRLTAFGTRENTEKAIER
```

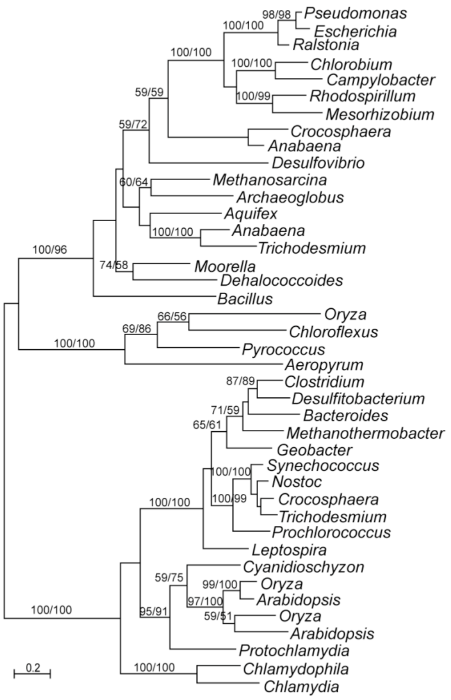

Figure 4. Phylogeny of Aspartate transaminase. It appears that gene duplication, differential losses and gene transfer were involved in the evolution of this gene. In the lower part of the tree, red algae and green plant sequences form a monophyletic group with *Protochlamydia* homolog with strong support whereas cyanobacterial sequences form another distinct clade. Two other chlamydial sequences appear to be more distant from those of *Protochlamydia*, red algae and green plants.

---

5. Bacterial tyrosyl-tRNA synthetase
CLUSTAL X (1.83.1) multiple sequence alignment

```
17935718_Agrobacterium_tumefa       RGFIHQIVTAYIGYDPTASSLHVGHLTQIMMLHWMQKTGHQPISLMGGGT
13470335_Mesorhizobium_loti         RGFIHQIVTAYVGYDATATSLHIGNLISATMLYWLQETGHRPIALMGGGT
67916376_Clostridium_thermo         RGYIAQLITFYIGFDPTADSLHVGHFLQMMVMAHMQKAGHRPIALIGGGT
34764162_Fusobacterium_nuclea       RGYLKQLVTFYIGFDPTADSLHVGHFIAMMFMAHMQQHGHRPIALAGGGT
15639820_Treponema_pallid           RGFIRQCLTFYVGVDPTGSSLHVGHMLPMFALKHLCDAGHRGCVLIGGGT
16768608_Drosophila_melano          RGFFHGIQSIYAGFDPTADSLHVGNLLVIMGLIHCQRAGHRPIALVGGAT
7705710_Homo_sapien                 RGLFKDFQTIYCGFDPTADSLHVGHLLALLGLFHLQRAGHNVIALVGGAT
89306341_Tetrahymena_thermo         RELLFQCFSAYAGFDPTADSLHMGNLISILTMIRLSLLGIKPILLVGGAT
66815010_Dictyostelium_discoi       RGFIHQMVSLYAGFDPTADSLHIGNLLTLMVMLHFKRHGHNPIALMGGAT
34914470_Oryza_sativa               RGLVEATLKAYCGFDPTAESLHLGNLLGLVVLSWFRRCGHNAVALVGGAT
6957729_Arabidopsis_thalia          RGLLESILRVYCGFDPTAESLHLGNLLGIIVLSWFQRCGHQAVGLIGGAT
153655_Chlamydomonas_reihd          RGLIQEVLSVYCGFDPTADSLHLGNLLGIVVLAWFQRCGHEPVALLGGAT
46400444_Protochlamydia_amoebo      RGFIDAVIKVYCGFDPTADSLHLGNLVAIMGLAWFQRFGHTPVAIVGGAT
CM3397_Cyanidioschyzon_merolae      RGLIDAVVAVYVGFDPTADSLHIGNLLGLLVLRWFQKLGHRPVAIIGGAT
76788775_Chlamydia_tracho           RGILDNSVSAYLGFDPTAPSLHIGHWIGICFLRRLAAYGITPVALVGGAT
15618276_Chlamydophila_pneumo       RNILENFIAAYLGFDPTAPALHIGHWIGICFLKRLAALGITPIALVGGAT
66854407_Anaeromyxobacter_deha      RSLVHDQITGYVGFDPTADSLHVGHLLAVMSLAWLQRCGGTPIIVVGGGT
53711359_Bacteroides_fragil         RGMVHDMVTAYVGIDPTADSLHIGHLCGVMILRHFQRCGHKPLALIGGAT
16080019_Bacillus_subtil            RGLIQQMIRLYSGFDPTADSLHIGHLLPILTLRRFQLAGHHPIALVGGAT
29376289_Enterococcus_faecal        RGLVYQVVKLYIGFDPTADSLHIGHLLPILMLRRFQQNGHVPIALVGGGT
```

```
75212974_Escherichia_coli          RGLVAQVIALYCGFDPTADSLHLGHLVPLLCLKRFQQAGHKPVALVGGAT
22125858_Yersinia_pestis           RGLVAQVISLYCGFDPTADSLHLGHLVPLLCLKRFQLAGHRPVALVGGAT
59800530_Neisseria_gonorr          RGLIAQTIALYCGFDPTADSLHIGHLLPVLALRRFQQAGHTPIALVGGAT
34498527_Chromobacterium_viola     RGLIAQTVTLYCGFDPTADSLHIGSLVPILMLKRFQQAGHRPVALVGGAT
32446275_Rhodopirellula_baltic     RGLLNQTQTIYIGFDPTATSLHVGGMMQLMMLRRFQRAGHNPIALVGGAT
6325160_Saccharomyces_cerevi       RGLVSQVIKLYCGVDPTAQSLHLGNLVPLMVLLHFYVKGHDIVTVIGGAT
75907148_Anabaena_variab           RGIAEVFLRVKYGIDPTGADIHLGHSIPMRKLRGFQDAGHTAVLIIGDFT
71675409_Trichodesmium_erythr      RGISEIFLRVKLGIDPTGVDVHLGHSIPVRKLRAFQDAGHTAVLIIGDFT
33861885_Prochlorococcus_marin     RGIEESFLRVKLGIDPTGTDIHLGHSILFKKLRAFQDNGHVAVLIIGDFT
46579366_Desulfovibrio_vulgar      RGCAELILRIKAGFDPTAPDLHLGHTVLIHKLRHFQELGHTVIFLIGDFT
15595865_Pseudomonas_aerugi        RGADEILLRIKAGFDPTAPDLHLGHTVLINKLRQFQDLGHQVIFLIGDFT
16080897_Bacillus_subtil           TGAHEVILKIKLGLDPSAPDVHLGHTVVLNKLRQFQENGHIVQLLIGDFT
4980991_Thermotoga_mariti          RNVVDLILRVKLGVDPSRPDLHLGHAVVLRKLREFQDLGHTVVLIIGDFT
15606818_Aquifex_aeolic            EGTVEIILRVKAGFDPTAPDLHLGHVVLLQKLRQFQQLGHEVFFIIGDFT
67919131_Chlorobium_limico         GNVVEIILKIKLGADPSRPDLHLGHSVVLKKLREFQDLGHEAILIIGDFT
45657564_Leptospira_interr         RGTVDLILKIKAGFDPTAPDLHLGHFVQLKKLKHFQDLGHEVSFLLGDFT
6460463_Deinococcus_radiod         RGVVDLVLRVKLGADPTRPDLHLGHAVILRKMRQFQDLGHKVIMLIGDFT
76260159_Chloroflexus_aurant       RGVAEVILRLKQGFDPTKPDMHIGHAVGLRKLRAFQELGHQVVLIVGDWT
                                               * *.:   :*:*       :      *     : *. *

17935718_Agrobacterium_tumefa      GMVGDPSFKEEARKLMTIDMIEDNITSLKHVFANNPALMINNADWLRGLN
13470335_Mesorhizobium_loti        SMIGDPSFRDDQRSLLTPEAIATNIEGIKRIFGRNDAIMVNNADWLMKLN
67916376_Clostridium_thermo        AMVGDPTGRTDMRKMMTREEIKHNADCFKKQLSKGKAIMVDNADWLLDLN
34764162_Fusobacterium_nuclea      GMIGDPSGRSDMRTMMTVEMIDHNVECIKKQMQKDKAILANNADWLRNLN
15639820_Treponema_pallid          ARIGDPSGKTSMRKMLDYATLDAYAGAIVAQLDHRHVFYVNNRDWLAHLN
16768608_Drosophila_melano         GLIGDPSGRKTERNQLGETVIETNLKAIEQQLRRAPLIIVNNADWYADLQ
7705710_Homo_sapien                AALGDPSGRTKEREALETERVRANARALRLGLEAGSFTVLDNSAWYQKQH
89306341_Tetrahymena_thermo        GLIGDPSGKTQERNMLTKEEVLSNVQGIENTLRKEDLQIINNYDFYKDMN
66815010_Dictyostelium_discoi      ALIGDPSGKSSDRVMLTEEFIEGNLKHIRENITAEDTLVVNNIEWNKTMD
34914470_Oryza_sativa              GRVGDPSGKSAERPELDLAAVETNSNAIKSLIGQGSFQILDNYDWWKDIT
6957729_Arabidopsis_thalia         GRVGDPSGKSLERPELDADTLEKNIAGITKIIIKGSYVIFNNYDWWKDMT
153655_Chlamydomonas_reihd         GRVGDPSGRSTERPVLSEEQIEHNVTSIGNLLRGPAVRVLNNLDWFGSMG
46400444_Protochlamydia_amoebo     GMIGDPSGKNAERQLLDEKKIQLNLKGISKNLEAAPAIILNNLDWFKNFT
CM3397_Cyanidioschyzon_merolae     ARIGDPSGKSVERPLLAASVIEENAARIAENLRQTEPLVLNNYDWLGNMR
76788775_Chlamydia_tracho          GMIGDPSGKSVERSLLDQAQVLDNSKKIAAALASPGIRIVNNADWLGSLS
15618276_Chlamydophila_pneumo      GMVGDPSGKQSERSLLQTSEVFDNSQKITACLQRPGVTLVNNADWLQEIS
66854407_Anaeromyxobacter_deha     GMVGDPSGKRSERPVLSVEEIDRNVAAIRAQLERNAARVRNNADWLRSIG
53711359_Bacteroides_fragil        GMIGDPSGKSAERNLLDEETLRHNQACIKKQLAKNRAELVNNYDWMKEFT
16080019_Bacillus_subtil           GLIGDPSGKKAERTLNTADIVSEWSQKIKNQLSRNPAVIANNFDWIGKMN
29376289_Enterococcus_faecal       GMIGDPSFKDAERSLNTLDTVKAWSESIKGQLSRNPAILANNYDWLGELS
75212974_Escherichia_coli          GLIGDPSFKAAERKLNTEETVQEWVDKIRKQVAPNSAIAANNYDWFGNMN
22125858_Yersinia_pestis           GMIGDPSFKASERKLNTEDTVNEWVEKIRHQVSPNSAIAANNYDWFGGMN
59800530_Neisseria_gonorr          GMIGDPSFKAAERSLNSAETVAGWVGSIRSQLTPNAAIMANNADWFGSMN
34498527_Chromobacterium_viola     GMIGDPSFKAAERKLNTPDVIEGWVEKIRKQVEPNAAVMANNYDWFGKMN
32446275_Rhodopirellula_baltic     GMIGDPSGKSEERNLLSADQLQKNVDGVAAQMRHNAAKLLNNFDWMKDYS
6325160_Saccharomyces_cerevi       GKVGDPSGRKTERDVMENDIRQSNVASISQQLQRGKYTPRNNFNWWKDIK
75907148_Anabaena_variab           ARIGDPTGKSEMRRQLTEEDVKQNAQTYLDQVRPGRLEVRYNSEWLSRLD
71675409_Trichodesmium_erythr      ARVGDPTGKSEARRQLTPEQVQENAKTYLDQVRPGRLEIRYNSEWLSKLD
33861885_Prochlorococcus_marin     AQIGDPTGKNKTRVQLSEEEVKENSKTYLNQLGMDRIEIRYNSEWLKNLN
46579366_Desulfovibrio_vulgar      GLIGDPSGRSDTRPPLTREQVLANAETYKQQVFKEKTVVDFNSRWMGEFG
15595865_Pseudomonas_aerugi        GMIGDPSGKSVTRPPLTREQVLENAETYKSQVFKAKTEVAFNSTWMDQLT
16080897_Bacillus_subtil           GKIGDPTGKSAARKQLTDEEVQHNAKTYFEQFGKEKVELHYNSKWLKTLN
4980991_Thermotoga_mariti          ARIGDPSGRNETRPMLTKEEVLENAKTYQEQAFKKRTELRFNGEWLDRMT
15606818_Aquifex_aeolic            AMIGDPTGRSQTRPPLSREQVLENAKTYEHQVFKEKTTVVFNSTWLEELG
67919131_Chlorobium_limico         AMIGDPSGKSKTRPQLSAEEARENGKSYFEQASKAKTTICYNADWLGKMS
45657564_Leptospira_interr         AMIGDPTGKSETRKRLSREEVLENSKTYQNQVFKVKTKIVYNSNWCSKMN
6460463_Deinococcus_radiod         ATIGDPSGKSKTRPPLSLEEARANAESYLAQCRLEALEIRYNSEWLEQLG
76260159_Chloroflexus_aurant       AQIGDPSGRDETRTRLSAAEVRANAETYMEQFFRQRTEVRWQSEWFGQFT
                                   . :***: :   *                            :  :

17935718_Agrobacterium_tumefa      YLEFLRDVGRFSVNRMLSFDSVKTRLSLSFLEFNYMILQAYDFVELCRLQ
13470335_Mesorhizobium_loti        YVEFLRDVGRFSVNRMLTFDSVKLRLSLSFLEFNYMILQGYDFVELCRLQ
67916376_Clostridium_thermo        YIEFLRDIGVFSVNRMLTAECFKSRLGLSFIEFNYMLMQSYDFLKLCIMQ
34764162_Fusobacterium_nuclea      YIEFLRDVGEFSVNRMLAAECYKSRMGLSFLEFNYMIMQAYDFYVLCTMQ
15639820_Treponema_pallid          YIDFLREVGAFSVNKMLTYEAYKKRLGLSFLEFNYQLLQSYDFLTLVELQ
```

```
16768608_Drosophila_melano          LIDFVANMGRFRMGSMLSRSSVQSRLGMSFTEFTYQIFQAYDWLHLCCFQ
7705710_Homo_sapien                 LVDFLAAVGVFRMGTLLSRQSVQLRLGMSLAEFFYQVLQAYDFYYLCRVQ
89306341_Tetrahymena_thermo         ILDFIRDIGKFRMGNLLNKETIANRLGLSYTEFTYTLLQGYDFEKLCIIQ
66815010_Dictyostelium_discoi       VISFLREVGSFRVGSMIKKDFIQNRLGISFTEFCYSLFQANDFAHLCSIQ
34914470_Oryza_sativa               LLDFLKEVGRARVGTMIAKESVKKRLGMSYTEFTYQLLQGYDFLYMVNVQ
6957729_Arabidopsis_thalia          MLDFLNKVGRARVGTMMAKESVKKRLGMSYTEFTYQLLQAYDFLHLINVQ
153655_Chlamydomonas_reihd          FLTFLREVGKARVGTMMAKDSVRTRMGISFTEFTYQLLQGYDFVHLVRVQ
46400444_Protochlamydia_amoebo      FISFLRDVGKFRLSPMLAKDSVKTRLGMSFTEFCYQILQGYDFLHLVTVE
CM3397_Cyanidioschyzon_merolae      LLDFLRDVGKARVGTMLSKESVRLRMGLSFTEFTYQLLQGYDFAYLVLLQ
76788775_Chlamydia_tracho           MVDFLRDVGKFRLGSMLAKDVVKQRVGISYTEFSYLLLQSYDFAHLVVLQ
15618276_Chlamydophila_pneumo       LIDFLGDIGKFRLGQMLVKDTIKQRVGISYTEFSYLILQSYDFYHLTILQ
66854407_Anaeromyxobacter_deha      LMEFLRDVGKFTVNYMLAKDSVKGRMGISFTEFSYQLIQAYDFWHLCELQ
53711359_Bacteroides_fragil         FLDFAREVGKITVNYMMAKESVKKRLGLSFTEFTYQLLQGYDFLHLCKLQ
16080019_Bacillus_subtil            VIDFLRDVGKFGINYMLAKDTVSSRIGISYTEFSYMILQSYDFLNLCKLQ
29376289_Enterococcus_faecal        LIDFLRDVGKFTINYMMSKESVKRRIGISYTEFAYQLLQAYDFLKLCLLQ
75212974_Escherichia_coli           VLTFLRDIGKFSVNQMINKEAVKQRLGISFTEFSYNLLQGYDFACLVVLQ
22125858_Yersinia_pestis            VLTFLRDIGKFSVNQMINKEAVKQRLGISFTEFSYNLLQAYDFACLVALQ
59800530_Neisseria_gonorr           CLDFLRDIGKFSVNAMLNKESVKQRIGISFTEFAYSLLQGYDFAELAVLE
34498527_Chromobacterium_viola      ALEFLRDIGKFSVNAMIKKESVQQRIGISYTEFSYSLLQGYDFAELCKLQ
32446275_Rhodopirellula_baltic      YLEFLRDVGKFPVGAMMGKESVRSRLGLSYTEFSYMLLQAYDFVNLCRIQ
6325160_Saccharomyces_cerevi        MLDFLADFGRIRVQSMLARDSISSRLGLGFNEFTYQVLQAYDFYHLVTIQ
75907148_Anabaena_variab            LGKITELLATMTVGQMLAKEGFADRYPIFLHEFLYPLMQGYDSVAIADVE
71675409_Trichodesmium_erythr       LADILELLATMTVGQMLAKEGFALRYPIYIHEFLYPLMQGYDSVAVADVE
33861885_Prochlorococcus_marin      LNSIIELMGSATVSQMLAKEEFNKRYPIALHEFLYPLLQGYDSVAVSDIE
46579366_Desulfovibrio_vulgar       AADFIRLASRYTVARMMERDDFEKRYPIAVHEFLYPLVQGYDSVALADVE
15595865_Pseudomonas_aerugi         PADFIRLASQYTVARMLERDDFSKRYPIAIHEFLYPLVQGYDSVALADVE
16080897_Bacillus_subtil            LEDVIELAGKITVARLMERDDFEERIPISLHEFFYPLMQGYDSVVLSDIE
4980991_Thermotoga_mariti           FADVIILASKYTVARMLERDDFAKRFPIAISEFLYPLAQAYDSVAISDVE
15606818_Aquifex_aeolic             TKGLIELCAKYTVARMLEREDFSKRFPIYIHEFIYPLLQAYDSVAIADVE
67919131_Chlorobium_limico          FSDVIRLSSHYTVARMLERDDFERRYPISIHEFLYPLAQGMDSVHLNDVE
45657564_Leptospira_interr          FEDVLVLSSKYNVARMLERDDFSKRYPISMIEFLYPLVQGYDSVAMCDVE
6460463_Deinococcus_radiod          YKDIIGLAAKYTVARILERDDFTKRLPISMHELLYPLTQGYDSVALADVE
76260159_Chloroflexus_aurant        LEHALDLAGRFTLAQMLAHETFRKRYPLTILELMYPMLQAYDSVAIADVE
                                            .   :  ::  .    *  :   *: * : *. *   :  .:

17935718_Agrobacterium_tumefa       MGGSDQWGNIINGIDLGHRMGLYALTSPLLTTSSGAKMGKSASGAVWLNK
13470335_Mesorhizobium_loti         MGGSDQWGNIINGVDLGHRMGLYALTTPLLTTSSGAKMGKSAKGAVWLNG
67916376_Clostridium_thermo         LGGDDQWSNILGGIDLIRRKEVYGMTFTLLTTSEGKKMGKTEKGALWLDA
34764162_Fusobacterium_nuclea       LGGDDQWSNMIAGVELLRRKDAYAMTCTLLTNSEGKKMGKTAKGALWLDP
15639820_Treponema_pallid           IGGDDQWGNIVAGADLVRRVRVHGLTFPLITRADGQKMGKTEQGALFLDP
16768608_Drosophila_melano          MGGSDQTGNLMTGHELISRVEVFGLTLPLVTTEEGDKFGKSAGNAVWLDG
7705710_Homo_sapien                 LGGSDQLGNIMSGYEFINKLTVFGITVTLITSTTGAKLGKSAGNAVWLNR
89306341_Tetrahymena_thermo         VGGSDQWGNITSGIELIRRKHVYGITTNLVLTAQGKKFGKSEGNALWLDP
66815010_Dictyostelium_discoi       IGGSDQWGNITAGCDLIRKKLAQGITIPLLTNSAGKKLGKSEGNSIWLSP
34914470_Oryza_sativa               IGGSDQWGNITAGTELIRKILAHGLTFPLLLKSDGTKFGKTEDGAIWLSP
6957729_Arabidopsis_thalia          IGGSDQWGNITAGTDLIRKILAYGLTFPLLLKNDGTKFGKSEDGAIWLSP
153655_Chlamydomonas_reihd          IGGSDQWGNITAGTDLIRRLLCYGLTFPLLVDSEGRKFGKSVGGAIWLAA
46400444_Protochlamydia_amoebo      LGGSDQWGNITAGTDLIRKVHAYGITFPLLTKSDGQKFGKSEKGAVWLSP
CM3397_Cyanidioschyzon_merolae      AGGSDQWGNIVAGLELTRKLYVHGLTFPLLTSKDGKKLGKTEKGAVWLSP
76788775_Chlamydia_tracho           CGGSDQWGNITSGIDYIRRRGAYGLTYPLLTDSKGKKIGKTESGTIWLDP
15618276_Chlamydophila_pneumo       CGGSDQWGNITSGIDFIRRKGAYGLTYPLLTNAQGKKIGKTESGTVWLDS
66854407_Anaeromyxobacter_deha      MGGSDQWGNITAGAELVSRKDVHGLTFPLLTTASGTKFGKTEGGAVWLDP
53711359_Bacteroides_fragil         MGGSDQWGNITTGTELIRRTNAYALTCPLITKADGGKFGKTESGNIWLDP
16080019_Bacillus_subtil            IGGSDQWGNITAGLELIRKSEAFGLTIPLVTKADGTKFGKTEGGAIWLDK
29376289_Enterococcus_faecal        LGGSDQWGNITSGIELLRREQGFGLTMPLITKADGTKFGKTEGNAVWLDA
75212974_Escherichia_coli           IGGSDQWGNITSGIDLTRRLHVFGLTVPLITKADGTKFGKTEGGAVWLDP
22125858_Yersinia_pestis            IGGSDQWGNITSGIDLTRRLHVYGLTVPLITKADGTKFGKTEGGAVWLDP
59800530_Neisseria_gonorr           IGGSDQWGNITAGIDLTRRLNVFGLTLPLVTKSDGTKFGKTEGGAVWLNA
34498527_Chromobacterium_viola      IGGSDQWGNITAGTDLTRRLNVYGLTMPLVTKADGTKFGKTESGTIWLDA
32446275_Rhodopirellula_baltic      AGGSDQWGNITAGIDLGRRMLLFGITAPLLTTSDGRKMGKTESGAVWLDP
6325160_Saccharomyces_cerevi        VGGNDQWGNITAGIDLINRIQPFGITVPLLTTATGEKFGKSAGNAVFIDP
75907148_Anabaena_variab            LGGTDQKFNIAVGRDLQRHFGQFGVLLPILIGTDGQKMSKSLGNYVSLSE
71675409_Trichodesmium_erythr       LGGTDQKFNIAVGRDLQRHFGQFGLLMPILLGTDGQKMSKSLGNYVGLSE
33861885_Prochlorococcus_marin      LGGTDQKFNIAIGRDLQRHFKQYGILLPILTGLDGKKMSKSESNTVGLSE
46579366_Desulfovibrio_vulgar       LGGTDQKFNLLVGRHLQSQYGQCILTMPLLEGLDGKKMSKSLGNYVGIDE
```

```
15595865_Pseudomonas_aerugi         LGGTDQKFNLLMGRELQRAYGQVILTMPLLEGLDGKKMSKSLGNYIGIQE
16080897_Bacillus_subtil            LGGTDQHFNVLMGRHFQERYNQVVILMPLLEGLDGEKMSKSKHNYIGINE
4980991_Thermotoga_mariti           LGGTDQLFNLLVGRKIQEEYGQIVMTMPIIEGTDGLKMSKSYGNYIAFND
15606818_Aquifex_aeolic             IGGTDQKFNLLIGRDIQREYGQVCITLPLLVGTDGRKMSKSYGNYVGITE
67919131_Chlorobium_limico          LGGTDQKFNLLVGRDLQREYGQVCITMELLVGTDGEKMSKSLGNAICFND
45657564_Leptospira_interr          LGGTDQKFNLLVGRDLQREYGQCVLTLPLLVGLDGKKMSKSLGNYVGITE
6460463_Deinococcus_radiod          LGGTDQLFNNLVGRALQRDYGQVVLTLPLLVGLDGEKMSKSLDNYIGLTD
76260159_Chloroflexus_aurant        FGGTDQKFNILAGRELMAQLGQQVFLVPLIPGTDGRKMSKTFNNTVDIRM
                                     ** **  *   *           .   ::    * *:.*:  . : :

17935718_Agrobacterium_tumefa       DVYDFWQYWRNTEDADVVRFAKLFTTLPMDEIAKKILATEVTAILHGRAA
13470335_Mesorhizobium_loti         DPYDFWQYWRNTEDADVERFLKIFTRLPLPEIAKKILATETTAIVHGREA
67916376_Clostridium_thermo         NPYEFYQYWRNIHDADVIKCLKLLTFVPMEEIAKKRLAFEVTKLIHGEEE
34764162_Fusobacterium_nuclea       KPYEFYQYWRNIDDQDVEKCLALLTFLPMDEVAKKVLAYEVTKIIHGEEE
15639820_Treponema_pallid           APYDFFQYWRNTPDEDVRRFLLLFTFLSVRDVAKELLAYEVTRLMHGTAV
16768608_Drosophila_melano          NPFALYQFFLRMPDSEVEKLLKLFTFIPLPQVAQTLLAEDVTLLVHGESG
7705710_Homo_sapien                 DPFELYQFFVRQPEDSVERYLKLFTFLPLPEIPQKRLAAEVTKLVHGREG
89306341_Tetrahymena_thermo         ASYDIFQYLINVPDQLSINYLKMFSFLPLHQIAQNTLAEQIIGMVQGDSQ
66815010_Dictyostelium_discoi       HPYKFYQYWIQVSDEDVERLLKLFTLIPLEEIGQKTIAEHVTRLIHGQKG
34914470_Oryza_sativa               KPYKFYQYFFSVPDIDVIRFMKILTFMSLDEIIQRRLAEEVTRFVHGEEG
6957729_Arabidopsis_thalia          SPYKFYQYFFSVPDVDVIRFLKTLTFLSLDEIAQIKLAEEVTRFVHGEEG
153655_Chlamydomonas_reihd          EPYKFYQYLFQVTDADVVKLLKMLTFLPLPEVAQKRLAEEVTRFVHGEEG
46400444_Protochlamydia_amoebo      DSYEFYQHLIRVEDADVINLMRMLTFLDMGEIAQKRLAEEITRLVHGEEG
CM3397_Cyanidioschyzon_merolae      QPYEFYQYLVKVADDDVCNYLRKLTFLPLEQIAQKLLAMEVTRIVHGDKG
76788775_Chlamydia_tracho           APYELFQYFLRLPDQEISKVMRTLTLLDNEEIVKKYIAEVIVKDVHGSEG
15618276_Chlamydophila_pneumo       DPFELYQYLLRLPDDTIPKIARTLTLLSNEEIVKEFVAQDILSAIHGDLG
66854407_Anaeromyxobacter_deha      APYKFFQFWLNTDDRDVERLLKFFTFLSLDEIAQRRLAEDVTARVHGPDV
53711359_Bacteroides_fragil         RPYKFYQFWLNVSDADAERYIKIFTSLDKAEILQKRLAKEVTVMVHSEED
16080019_Bacillus_subtil            EPYEFYQFWINTDDRDVVKYLKYFTFLSKEEIAQKRLAEEVTSLVHGREA
29376289_Enterococcus_faecal        EPYEFYQFWINTDDRDVVKFLKYFTFLTLDEIAQKALAKEVTTLVHGEAA
75212974_Escherichia_coli           KPYKFYQFWINTADADVYRFLKFFTFMSIEEIVQYVLAELVTRLVHGEEG
22125858_Yersinia_pestis            KPYKFYQFWINTADADVYRFLKFFTFMSLEEIAQYVLAENVTGMVHGPEG
59800530_Neisseria_gonorr           KPYQFYQFWLKVADADVYKFLKYFTFLSIEEIAQRILAEEMTRLIHGEEA
34498527_Chromobacterium_viola      KPYAFYQFWLGTADADVYKFLRYFSFLSVDEIGQRILAEQVTELVHGKAA
32446275_Rhodopirellula_baltic      EPYAFYQYWINVADEDVMRCLAYLTEIERAEYAQKRLAQWLTELVHGEAG
6325160_Saccharomyces_cerevi        SAYDVYQFFYNTLDADVPKFLKIFTFLNSSEIGQTLLAKEVTDMLYGVGS
75907148_Anabaena_variab            HPGQKYQKLQGVPDQMLEQYFELLTDLPID--RQMLLAWEIVKQYHGEQA
71675409_Trichodesmium_erythr       DPLSMYSKLEKIPDDQVENYFELLTNLNINDIRQKLLALDIVSQYHSQEA
33861885_Prochlorococcus_marin      DSLSMYSKLEKVPDNIIPSYFELLTELDLTLLLQRRMALEVTSLYHGKEE
46579366_Desulfovibrio_vulgar       SPADMFGKLMSVSDELMWRYFELISSRSLDEIVKESLAYELTTRYHGEDK
15595865_Pseudomonas_aerugi         APGVMYSKLVSIPDTLMWRYFELLSFRSLDEIIKIKLAEEIVARFHGEEA
16080897_Bacillus_subtil            HPNDMYGKTMSLPDSLMKKYIHLATDLELEEKAKMLLARTIVRMYHGEKA
4980991_Thermotoga_mariti           PPEEMYGKLMSIPDELIIKYMRLLTDIPEERIVKMVLAYEITRFFHGEEN
15606818_Aquifex_aeolic             DPKTMFAKIMSIPDEIMWDWFLLLTDYNKEEIAKKLLAFTIVKRFHSEEE
67919131_Chlorobium_limico          PPNDMYGKVLSIPDTLIETYHRLLVTGNKELLAKRTLAKDIVAQYYSINA
45657564_Leptospira_interr          TPIDMFGKLMSISDDLMWNYFELLTDLPLPEIIKTELAKLIMDQFSSPSE
6460463_Deinococcus_radiod          EPHAMFAGLMKVPDPLLDNYFTLLTDLPRERIAHRELAREVVRAFHPDAD
76260159_Chloroflexus_aurant        PPSEMYGRIMSMSDEVLPLYFEVLTDVPLAEILKMRLAREIVAQFHDPAA
                                         :       :                   :  :*

17935718_Agrobacterium_tumefa       AEEVLSLIVRAGLASSNGEARRHVQGGAVKINEQGVSKLSVGKKKHVLV
13470335_Mesorhizobium_loti         ANQILSLLVTAGLASSNGEARRHIQGGAVRINDQSVSKLSLGKKKHVLV
67916376_Clostridium_thermo         ALNIIDLLLKTKLIPSKGEGRRLIEQGGISVNDVRVEIIKKGKKTYHRV
34764162_Fusobacterium_nuclea       ATKLLDVLVDRKIIKTKSEGRRLIEQNGMSLNDEKIKLLKLGKKKFYNI
15639820_Treponema_pallid           AQVVTDLFVQVGLCTTKSDARRLIAQGGAFVGLQRVAIVRAGKKRVVRI
16768608_Drosophila_melano          LKQILELAMKAKCFPTETDAVRIINAGGFYVNQKRVQLLRVGKRNFYIV
7705710_Homo_sapien                 LDSVLDTCRKANAIPDGPRGYRMITEGGVSINHQQVTLLKIGKRNFYII
89306341_Tetrahymena_thermo         SAEYIDMIGDLKLLKNKAEIKRNIENGGAYINNKRISVARFGKKNYYLI
66815010_Dictyostelium_discoi       VEEILNLFAQIS-DASKSKVKQLLQSKSIYLNNIPVAYLKSGKKIYYLI
34914470_Oryza_sativa               LEELVDLAVSTGLLASKSAVRRLIKQGGLYLNNIRIDLLSAGKKNKMVV
6957729_Arabidopsis_thalia          LKEIVDLSVSAGLFESKSAARRMLKQGGFYMNNERVDVLSAGKKNKVVV
153655_Chlamydomonas_reihd          LAQLADVMVAIKLQPSKSAARKLIKGGGVYLNNAKVVLVAAGKKNKMLV
46400444_Protochlamydia_amoebo      LKILIDLLVETKLQTSKSEARRLLRNGGVYINNKKIELLAAGKKNKMII
CM3397_Cyanidioschyzon_merolae      VETLVDVMVRAGLQKSKGEARRLIANGGAYVNDEKVSLLAAGKKNKMLV
76788775_Chlamydia_tracho           LAQWLDIVVELGFCSSRGQARRLIQQRGLYINQEPLALLSQGKRKKQVI
```

```
15618276_Chlamydophila_pneumo       LEEWLDLFLVLGLCKSKGEIRRLIEQKGVYINNVPIALLAQGKKRKLVL
66854407_Anaeromyxobacter_deha      TRSVADLLVKVGLAASKGEVRRGVAGRGFSLNGAVLELLQKGKRNYALV
53711359_Bacteroides_fragil         YNAAVDLFVNAAVFASKGEMRKLVQGGGVSLNKEKLALVQRGKKNYYLI
16080019_Bacillus_subtil            LEQLVDVLVQSKLSPSKRQAREDIQNGAVYINGERQTVLRRGKKKYFLV
29376289_Enterococcus_faecal        YHQLVDLLVTSKIEPSKRQAREDVQNGAIYVNGERRQVIRRGKKKYFLL
75212974_Escherichia_coli           LQALMQALVDSELQPSRGQARKTIASNAITINGEKQSLLRRGKKNYCLI
22125858_Yersinia_pestis            LAALQQALVNAELVPSRGQARTMIGSNAVAINGEKQALLRRGKKHYCLI
59800530_Neisseria_gonorr           LAAAVEALVKTGLAASNKEARGFVNAKAVLLNGKPAEILRRGKRNHALL
34498527_Chromobacterium_viola      LEALIDALAAGGLAKSKSEARTFIQSGAVSVNGIKVDLLKRGKKLYALV
32446275_Rhodopirellula_baltic      VQSIVEALQTAKLCNSGGDARRALSEGGVYVNNTRVEVLRRGKRKYALL
6325160_Saccharomyces_cerevi        GSDEIDLIKICKLVNSVSEARRKLSQGSVYLHHSKSKILRIGKQKCFII
75907148_Anabaena_variab            AQELAYLLGATGLCKSTGEGKRKIQEGGVRLDGDRISVLQVGKNKFVRL
71675409_Trichodesmium_erythr       ALELFYVVSASGLCKSSSEARKKIAGGAVRLDDERISVLRVGKKNFVRL
33861885_Prochlorococcus_marin      ALRFFYLLSSLNLFKSSSESKRSIKGGGVKIDSYKLVILQIGKKIIKRF
46579366_Desulfovibrio_vulgar       AAEPPAFLEAAGLVKSRGEAKRLIKEGALSVDGVRCDVIKLGKKRFLRL
15595865_Pseudomonas_aerugi         AASVASVLNKAGLVKNAAAARDLLGAGSVKVDGQVV-VFQAGKKAFARI
16080897_Bacillus_subtil            AEAMIDLLVKLKLLSSKSEARRMIQNGGVRIDGEKVTIIQVGKRKFLKL
4980991_Thermotoga_mariti           AKKIVDLLVEIGAASSKSEAKRLVSQGGVYIDGERIEVLRVGKRKFYRI
15606818_Aquifex_aeolic             ARKAVDFLVKIGAVKSKNEARRVIQGGGLKINGEKVTKVKVGKKKFYRV
67919131_Chlorobium_limico          AREVTDLLVSLGAAPSKSEARRMIQQNAVTVNEEKITILKAGKRKFYKI
45657564_Leptospira_interr          NEELVWVLSKLSFIPSVSEGRRLIKAGGLYLSEDKITLVRQGKKGKFLK
6460463_Deinococcus_radiod          LDAMAKLVVLAGLEPSNGAARKLIQNRGLKLGGETYSVIQKGKDKFARL
76260159_Chloroflexus_aurant        AAAIVEVLVASGLAPSKSEARRLIDGGGVRVDGERVEVVQVGRRKFVRV
                                                            :   .  :          *:
```

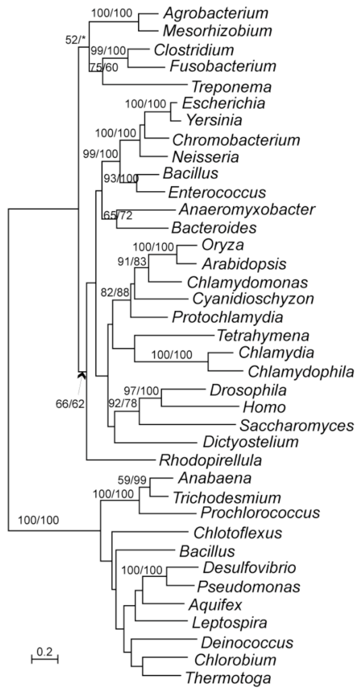

Figure 5. Phylogeny of bacterial tyrosyl-tRNA synthetase. Again, sequences of red algae, green plants and *Protochlamydia* form a monophyletic group. Sequences of *Chlamydia* and *Chlamydophila* group with *Tetrahymena* homolog, but with insignificant support. The sequences of *Dictyostelium*, *Saccharomyces*, and *Homo* are annotated to be mitochondrial precursors and therefore of likely α-proteobacterial origin.

---

6. Oligoendopeptidase F

CLUSTAL X (1.83.1) multiple sequence alignment

```
33235980_Chlamydophila_pneumo       WDTTLMYLSKKFSVERKLDQLYIYAHLIHDQDIYTLFSQEISWIQPALIA
15835007_Chlamydia_murida           WDTKSLYLTEVFSIERTLEKLYVYAHLTHDEDILTSFSEEISWIQPALIA
46400453_Protochlamydia_amoebo      WNVEELYLDLMMSMDRELSKLYTYAHLRHDEDIAHKFSQEISWFQPELLA
CM3763_Cyanidioschyzon_merolae      WDLSRLYLEEYLRIARVIERVYTYAHLLHDEDLHTRYAEQTSFLEPELVE
87310907_Blastopirellula_marin      WDLASLYLKFDLDVDRLLERVGTYAFLKTTENQGSKLGQAASYISPEIQS
32446842_Rhodopirellula_baltic      WDLSSLYLDFDGEFDRMAERCGTYAFLRTTENQAVKASQASSFVRPELLS
2688150_Borrelia_burgdo             WDLSFLFLNKYYEIMEDLEKVSYYAMLQLETDVATKVSNTTSYFMPKILK
41819184_Treponema_denticola        WNIELLFLKASTGVDRIAEKVGNYAFLQKSSNEVTELSAATSWLMPAIME
65320776_Bacillus_anthra            WNLTDIYLTKSEEISSIISLMFAYARLQSDLDTHVKVSAAKSFFSPFLLS
28211583_Clostridium_tetani         WNINKLYFKTEESILRLAEKLSVYAHCRSDEDTFPEILGLSSFFIPEILS
76260026_Chloroflexus_aurant        WDPYSIFLALSEDILRNVGQITVFATMFYTVDTQARASAALAFGEPELLA
66798507_Deinococcus_geothe         WDIEALFLRAADEVELRLTRLLSYASMAASVDGAARFGSVTAFAKPELLA
76259709_Chloroflexus_aurant        WDLSQIFLTLRDEVGRRLYAIYVYATHLKDSDSAARIQAALAFIEPELLT
55379750_Haloarcula_marism          WDLDSLYLETYEDLMRTVSNVAAYARMRRDEDTSSEASSAASFLDPELQD
15790868_Halobacterium_sp.          WALASIYLELRDAVYRDAKTVSAYASMRSDEDTMTDVSSATSFFDPELQA
68194738_Enterococcus_faeciu        WDLTKIFIEFVLRVYRQTEVIYVYAHLKNDQDTFSKVSEAVSWFEPEILQ
16414804_Listeria_innocu            WDLTTIYLQFRDKAYDLISNLYVYAHLKMDQDTVTKLMSALSYYDPEILA
65318570_Bacillus_anthra            WRLEDIFLQYEDEISMRLGKLYTYAHMRYDQDTYSQVSSSTAYIVPEILS
56379199_Geobacillus_kausto         WRLEDIFLQCQDDVSMRLGKLYTYAHMRYDQDTYSEASSAMAFIVPEILA
68056395_Exiguobacterium_sibir      WNLESIYLQLRDEISRRLHKLYTYAHMRYDENTASQIGATLAFMTPELLE
27467608_Staphylococcus_epider      WDLTTIFLSLEDELGTKLEKVYVYAHLKQDQDTVIKYSSAWSFLVPEILQ
66856279_Anaeromyxobacter_deha      WKLADLFLSEMGALQNALDRVYVYASARADEDTAVDVQAALSWVRPAVLE
56380195_Geobacillus_kausto         WNVADIYFALNEQTARKLEKLSLYAHLKRDINIAAQYAAKTAFIEPELLA
83319855_Mycoplasma_capric          WDFDYLYLDLEEKVDFITNKLSTYTRMG-DTNQLQDIEIETSFISPETKK
19714448_Fusobacterium_nuclea       WNLNDIYEILMEKIAKLLDRLYLYPYMLKDLDSYSKFATETAWISPEMLE
65317438_Bacillus_anthra            WDLSDLYLLLEEELLMKLTKLYSYANLKESTDRWTKVHTALSFIHNEILS
27467983_Staphylococcus_epider      WDLKDLFLDEYERILIEIDRLYNYPELRLSVDTSGKLAGLLSFVDSEILE
34483139_Wolinella_succin           WDLSALFIGEYENLSDAIGRIMSYAFLVFAEDTCNEISEHLLFFEIEFGD
57240464_Campylobacter_lari         WNLSTLFLKNYEELILKLSHILTYVYLNFAQDTSKKIEENLLFFELEFCE
77740978_Rhodopseudomonas_palu      WNLADLYVKRYEAIDDLAGRLGSYAGLAHAGDSLTAASVHLLFFALELNR
15075820_Sinorhizobium_melilo       WRLTDLYVKEFEALDDLMGRIASFAGLTYFSDTLTDISAHLLFFSLELNR
78193764_Geobacter_metall           WDTDPLYLRRYEALTELLVKPQLYAHLLFAADAGNLMGRELLFFDLEIME
                                    *    ::                :       :          :

33235980_Chlamydophila_pneumo       LHTGTANEEKILARRTAYLAQFQRYYDYRNTFANLLNGKVQAHLFEAKAR
15835007_Chlamydia_murida           LHTGTSREEKILARKSAYQKQCQRYHGYRLSLANLLNGKIQAHLFNAKAH
46400453_Protochlamydia_amoebo      LHTLSFENEKLLAREKAFNQYHHQYLAYENTLCELLSGQVQNHLFQARSR
CM3763_Cyanidioschyzon_merolae      VHTLSASEEALLARRNAFTTLHGKYRSFENTLAELLSGAVETHVFHAKAR
87310907_Blastopirellula_marin      IFTLSKKEERILARKTAFHQYYAQFKGHENTLAATLSGSIQRDVYYAKVR
32446842_Rhodopirellula_baltic      IHTLTDREERLLARKKAFDQYYQVFSEHENTLAATLAGSVHRDVYYARAR
2688150_Borrelia_burgdo             THILSEQEEKILARKEAFLRFYQKYKNNENTLANLIISDFKKNHFIAKTR
41819184_Treponema_denticola        IHTLSDKEEKILSREKAYKQLYGVYGAHKNTIASLYTGQVQQNVALAKIR
65320776_Bacillus_anthra            VHVLNKDQEEILSRKEAYKAYYKPYVQLKNSIASTLSAAIKNNVTVSKLR
28211583_Clostridium_tetani         IHILSKEEEMILARKDAFEALFDTYEKYKNTFSTALTSSIKNFIFNSKMR
76260026_Chloroflexus_aurant        IHIRSAEVEELLGRKNAWEGYADAHIAVENTMAQCLAAGVKQNVFIARAR
66798507_Deinococcus_geothe         LHVRSPEVEELLGRRQAWENYADAHLAVRHAQAAMYAANVRQNVFLARAR
76259709_Chloroflexus_aurant        IHIRSAEVEQVMARRDAFKGYYSAFLPFRNTIATTLGAAIRSHVIEARLR
55379750_Haloarcula_marism          LHTRSAEVENLLARQRVYEAFYDEWETVRNAVGTAYKNAVKTDVKMANAR
15790868_Halobacterium_sp.          CHTRSAEVEGLLSRQTVHEQFYDTISGVRNTIGSTMKTQMKRDVKLAEAR
68194738_Enterococcus_faeciu        LHVLSAEQESLLAREAAFKGLYSVYEQFRNTFASTLGTHIKGHNFKAKVR
16414804_Listeria_innocu            MYILSEKEEALLARKDAFLGVYSVYEGLKNTLASTLNGQVKKSNFYASTR
65318570_Bacillus_anthra            IHVLSEAEEALLAREDAFKAVYETYGKYKNTFASTLSGAVKRNNFNARVR
56379199_Geobacillus_kausto         IHVLSAEEEAILARRDAFYAVYHTYEKFQNTFANMLAGTVKKDNFFARIR
68056395_Exiguobacterium_sibir      VHVLSEAEEAILAREAAFKAMYGTYSQYTNTLASTLAGSVKKDNFYAEVR
27467608_Staphylococcus_epider      LHILDANTEKLLTRASAFRNVYKAYGAHNNTLGATLAGEVKKNVFNARTH
66856279_Anaeromyxobacter_deha      LHTLPAGEERIVAREKVFHAYFGALKQYERTIGTALYAQVKAHLFERDVR
56380195_Geobacillus_kausto         LHTLTKREERLLARKAAFEAKAKSYEAIEQTAAATLYASVQADELYANVR
83319855_Mycoplasma_capric          IHILSEHDEELLSRLKASEIFSKNFSSKKHSFALIYEGILQSDYEDTKLR
19714448_Fusobacterium_nuclea       IHVLSEDKEQLLSRKLAFEALYKSYENSKNTFAAIYRAIVQRNVASCNAR
65317438_Bacillus_anthra            LYTLSPETEEALARRKAYSSFVSTLKRYKNTVATTYATEVKKQVTLSRLR
27467983_Staphylococcus_epider      LYQLSADVEKVLARRKSFRAFSDALRQYQHTTAATYNMQVQQEKIEADLR
34483139_Wolinella_succin           LHTLSLPEERIMLREKAARSLTKGLAPHQGVLAYILNVVRKENAILCDIR
57240464_Campylobacter_lari         LHNLSKKEERVILRKKASKCFSKTLKKQNKLLVYIFNMIKSELASICELR
```

```
77740978_Rhodopseudomonas_palu      IYQLEDRVEQLFHRKAAGQALAKTFKANERTFALITNTLAKDKEISDRWR
15075820_Sinorhizobium_melilo       IYQLDDRLEQLFLRKKAALALAETFKANIRTFTLVTNTLAKDKEISDRWR
78193764_Geobacter_metall           IHTLTEREEQLLKRERAFAIFLRRHEEEGIALGAIFNTVALDHGQELELR
                                     .      *  . *                                   :

33235980_Chlamydophila_pneumo       NYPSCLEASLFQHNIPTTVYINLINETKLINRYFNLKKEALNLKEFHFYD
15835007_Chlamydia_murida           NYKSCLEAALFQNNISTSVVTTLIETVKLITKYFQLKQKALGLPDFHFYD
46400453_Protochlamydia_amoebo      HYTSCLESALFPKNIPTSVYHALIEAVTVLHRYMGLRKRILKLDQLHLYD
CM3763_Cyanidioschyzon_merolae      RYRSALEAALTPNAIPTKVYENLLDTIRHHHRYVALRRRLLGVDRIHMYD
87310907_Blastopirellula_marin      GYDSAIHQALYADDVPQSVYDNLIESVHALHRYYELRRRKMKLRDIHHYD
32446842_Rhodopirellula_baltic      NYESSLEASLFPDNMPVSVYDNLIQAVRAVHEYLDVRRRKMNLPDMHHYD
2688150_Borrelia_burgdo             RFQNTFSMQLFSNNIDKKVYTNLIETVNVLNDYYEFRKKVLNQEYLYHYD
41819184_Treponema_denticola        GYASAREKSLYVDKVPTAVYDNLVDTIHPLHKFYSMLKKHLGLKELRHYD
65320776_Bacillus_anthra            KYPSALEKSLFGDMVPKEVYENLIETTKSLHTYNKLRKEKLNVDELRQYD
28211583_Clostridium_tetani         KFNSSLESSLKPNNIPLEVYDTTIKTINSLHRYVKIKKKLLGLEEMHMYD
76260026_Chloroflexus_aurant        RYASALEAALKPNFIPLEVFHNLIATFEIWHRYWRVRRAALGVDELHVYD
66798507_Deinococcus_geothe         RYPDAITASLAPARIPVEVVTTLLATYRTWHRYWNVRREWLHLPELREYD
76259709_Chloroflexus_aurant        NYSSALEAALAPNEIPVEVYHNLIATVERFHRYLSVRRRLLGLDELHFYD
55379750_Haloarcula_marism          DYDTAREAALDGPNVPVEVYDTLVDTVHTLHRHADLKRQSIGADELRMWD
15790868_Halobacterium_sp.          NYDTARQAALDSGNIPVEVYDTLVDTVDTLQRHADLKRRALGVDDLEMWD
68194738_Enterococcus_faeciu        NYSSAREASLSNNHIPESVYDTLVDVVNLLHRYMELRKRLLEVEKLHMYD
16414804_Listeria_innocu            GYKSAREAALSGNHIPETVYDSLLKSVNLLHRYVKLRKELLGLEELHMYD
65318570_Bacillus_anthra            KYDSARQAALSNNNIPEAVYDQLIESVNLLHRYIDIRKRALGLDELHMYD
56379199_Geobacillus_kausto         RYRSAREAALDANNIPESVYDNLIAAIHLLHRYVRLRKRVLGLDELHMYD
68056395_Exiguobacterium_sibir      KFKTARESALHGNTIPEQVYDGLIEAVHLLHRYVALRKRILGVDELHMYD
27467608_Staphylococcus_epider      HYHSARERALSNNHIPEAVYDNLIKTVHLLHRYTKLRQELLGLDDLKMYD
66856279_Anaeromyxobacter_deha      RFDSSLAAALFRDNVPTAVYERLVADVNTLHRYLKLRQRMLGLPALRYED
56380195_Geobacillus_kausto         RYPSGLAAALAADDVPKEVFDHLIAATRALHRYVELRRRALGLDRVHSYD
83319855_Mycoplasma_capric          SYDSSLQASLSSDSISIDIYLKLLEIGKPLEDFLLLTKKHFKLDKFYPSD
19714448_Fusobacterium_nuclea       NYESSLDRALENKNIPREVYFSLVESTQPLRRYVELRKKALKLKEYHYYD
65317438_Bacillus_anthra            KFESVTHMLLEPQNVPLEMYNNQLDIIYHMRRFADLKKKVLGLDQMLFCD
27467983_Staphylococcus_epider      GYDSVIDYLLQDQEVTKDMFDRQIDVIMVMQKYAKIIQRVHNLDKMRFED
34483139_Wolinella_succin           GYATPEESRHIDNQISQKSVDSMLEVVNIVEEFYDLKREILGYEELYDYD
57240464_Campylobacter_lari         SYENPETPRHIRNQISKKSVDALISASEIVSKFYNTKKKILGYKKLKDYD
77740978_Rhodopseudomonas_palu      GFEDIADARHLSNRVEREVVDALVNSVRLSHRYYALKARWFKKKSLAHWD
15075820_Sinorhizobium_melilo       GFEDIADSRHLANRVEREVVDALAASVKLSHRYYAMKAKWLGMEQMDFWD
78193764_Geobacter_metall           SYRHPMEPTNLGNEIPEEVVNRLMDVSELARDYFRLKARLLGLPKLKNTD
                                     :             :                 .                *

33235980_Chlamydophila_pneumo       VYAPISYEEGVDLVCKSLLPLGTHYVEILRNGLRWVDRYENKHKRSGAYS
15835007_Chlamydia_murida           VYAPISYEEAVSLICDSLTPLGDNYVEALRKGLGWVDKYENQNKRSGAYS
46400453_Protochlamydia_amoebo      IYVPLSYKEAEDIVIESVAPLGSEYQSLLAKGLRWVDRYENQNKRSGAYS
CM3763_Cyanidioschyzon_merolae      MYAPLPYEDAVKSVLASLAPLGDEYLRVASAGLRWVDRYENKGKRSGAYS
87310907_Blastopirellula_marin      TYVPITWDEGVESIMDALIPLGTEYCDVLKDGLRWSDRYPNAGKQSGAFS
32446842_Rhodopirellula_baltic      TYVPITWDQAVNVVLESLQPLGDEYVSTLEEGLRWADRYPNRGKQSGAFS
2688150_Borrelia_burgdo             VYVPLSFEDACEKILKSLEVLGNEYTKILRNGLRWVDKYENTGKRSGAFS
41819184_Treponema_denticola        VYMPLPYNEAVDIITEALKPLGEEYVKTIRNGLGWVDKYENEGKRSGAFS
65320776_Bacillus_anthra            LSVDLPYDNAFDIMIESLAPLGEEYIETLKSFKRYIDVRETPGKCSGAYN
28211583_Clostridium_tetani         LYVPLPFEEGINMVEKGLKPLGEEYLNVFKKGIGWIDIYENKGKRGGAYS
76260026_Chloroflexus_aurant        TKAPLPYEQAVDWICEGMAPLGQEYVQIMRRGLRWVDVYPNRGKRAGAFS
66798507_Deinococcus_geothe         VKAPLTYAQAVEWLCEGLAPLGSEYIQDMRAGLRWVDYAANDGKRQGAYS
76259709_Chloroflexus_aurant        LYVQPPYHEACDLMRSAFRPLGEEYSAALEQMFRWIDVYENVGKRSGAYS
55379750_Haloarcula_marism          LYTPLEYEQACEYVTEAVAPLGDDYQSRLADGLRWVDVYETEHKQSGAYS
15790868_Halobacterium_sp.          LYMPIEYADAREHVIEAVAPLGEDYQQRVADGLNWVDVYENRGKRSGAYS
68194738_Enterococcus_faeciu        LYTPVTYEEAKEKALEALKPMGEEYMAIVEKAFRWIDVVENKGKRSGAYS
16414804_Listeria_innocu            LYTPLTYEEAKEIVLEALKPLGDEYQSILKEAFRWIDVMENKGKRSGAYS
65318570_Bacillus_anthra            LYTPLKYEEAQDMLLKSLHVLGDEYVDILKEAYRWVDVYENKGKRSGAYS
56379199_Geobacillus_kausto         LYTPLTYEEAKQYMLEGLAPLGEEYVAIVKEGLRWVDVRENKGKRSGAYS
68056395_Exiguobacterium_sibir      MYTPLSYEEAKQLMVDGLAPLGPEYKHILEEGLRWVDVRETRGKRSGAYS
27467608_Staphylococcus_epider      LYTPLPYEEAKSWMLKALEPMGEEYLNVVKEGLRWVDVYENKGKRSGGYS
66856279_Anaeromyxobacter_deha      LYVPLDVDQAVALTLDAVKPLGDAYVSKLRAGLGWTDFLPVTGKRAGAYS
56380195_Geobacillus_kausto         LYVPLPVETAKTLIVEGLKPLGADYIKQVHRAFRWLDVYPRPKKYTGGYN
83319855_Mycoplasma_capric          RQLKLTVEQAKEHIRKALSILGEEYLKNLEIAWNKIDYYEDTNKRDGAYS
19714448_Fusobacterium_nuclea       NSINIKYDDAKEMVLKSVEPLGEDYQQKMKRAIGWLDVFETKNKRSGAYS
65317438_Bacillus_anthra            LHAPLTYEEAGKLIQDSLQVLGDEYSAIIKKGFRWVDLADNVGKSTGAFC
```

```
27467983_Staphylococcus_epider      LKISISIEESKKYIYGALKVLGDDYVKMLESAYRWIDFAQNKGKDTGAYC
34483139_Wolinella_succin           RYAPLDFESAQKIVLQALGEFSPRFGEIAQRAFGWIDSHPRPRKRGGAFS
57240464_Campylobacter_lari         RYAPIDFDQGKDIVLKAFEKFSKDFYKIAKEAFNWIDVYPKEFKQSGAFS
77740978_Rhodopseudomonas_palu      RNAPLGWPEAQDMVLSAYGAFSPEMASIAQRFFRWIDAPVRPGKAPGAFS
15075820_Sinorhizobium_melilo       RNAPLPWNEARDTVLSAYHAFAPEMAAIARRFFGWIDAPVRPGKAPGAFA
78193764_Geobacter_metall           VYAPATFEEARDLVLEAYGAFQPRFRDICAAFFRRIDVLPRPGKGGGAFC
                                             .      .   :               *      *  *.:

33235980_Chlamydophila_pneumo       SGCYDPYILLNYTNTLYDVSVIAHEAGHSMHSYFSREAYHDAQYPLFLAE
15835007_Chlamydia_murida           SGCYDPYILLNYTGTLYDVSVVAHEGGHSMHSFLSHKHYHEAQYPIFLAE
46400453_Protochlamydia_amoebo      SGCFDPYILMNYKNLLRDVFTLAHEAGHSMHSLYSRRTYQYSDYPIFLAE
CM3763_Cyanidioschyzon_merolae      SGCYDPYILHNYDANINDAFTLAHELGHSMHSHFSRQHYHYADYSIFVAE
87310907_Blastopirellula_marin      CGTFDPYILMNYKDVLDDVFTLAHEAGHSMHSYYSAGNYQYYNYTIFVAE
32446842_Rhodopirellula_baltic      CGSFDPFILMNFKEVLNDVFTLTHEAGHSMHSWYSSKNYQYYDYTIFVAE
2688150_Borrelia_burgdo             AGSYNPYILLNYKESIRDMFTLAHEAGHSMHSYFSIKNFPHYNYSIFEAE
41819184_Treponema_denticola        AGGYDPYILMNYKDVIRDVFTLVHEGGHSMHSWYSVRNYPCYHYTIFEAE
65320776_Bacillus_anthra            FGVYGPFILLNHHDDLNSLFTLTHECGHGMHTHYSHGYRISAHYTIFVAE
28211583_Clostridium_tetani         SGSYDPYVLLNYNYKLNDVSTLAHEMGHSIHSYYSKENYIYSNYTIFCAE
76260026_Chloroflexus_aurant        TGAPGPFIMMSYNDDIFGLSTLAHELGHSMHSYYTRRTVIYTNYGLFLAE
66798507_Deinococcus_geothe         NGSARPYIFMTWQGTLGSYSTLAHEIGHSMHSLLSQRAYAVPRYTLFHAE
76259709_Chloroflexus_aurant        GGSYGPYILLNYQDRLRDVFTLAHELGHSLHSYFTRTHFVYGNYTIFVAE
55379750_Haloarcula_marism          GGTYDPFILMNYQDDVESMYTLAHELGHSMHSEYTSEEYVYSGYEIFVAE
15790868_Halobacterium_sp.          GGTYDPFILMNYQDDISSMFTLAHELGHSLHSQYTSEHYVYSNYEIFVAE
68194738_Enterococcus_faeciu        SGSYDPYILLNWHDTLDQLFTLVHEMGHSVHSYFTRSNYVYGDYSIFLAE
16414804_Listeria_innocu            SGSYSPYILLNWQDNINNVYTLAHELGHSVHSYYTRKNFVYGDYSIFLAE
65318570_Bacillus_anthra            SGAYGPYILMNWHDNVNNLFTLAHEFGHSVHSYYTRKTHVYGDYSIFVAE
56379199_Geobacillus_kausto         SGAYGPYILLNWQDNVNNLFTLVHEFGHSVHSYYTRKTYPYAHYSIFVAE
68056395_Exiguobacterium_sibir      SGAYDPFILMNWQDNVNNLFTLAHEFGHSVHSYYTRQNYAYGDYSIFVAE
27467608_Staphylococcus_epider      SGGHLPFILLNWSDTVSDLYTLVHEFGHSAHSYFSRQNSNLSDYTIFVAE
66856279_Anaeromyxobacter_deha      TGVYGPYQLLNFNGQWTDVSTLAHEAGHSMHTVLAFEKYATSNYATFVAE
56380195_Geobacillus_kausto         TGAYDPFILLNYNGSLDSVLTMAHELGHAMHSVYTNRTYHYSGHSIFTAE
83319855_Mycoplasma_capric          TGGHGPIILMNWDDKLNSVNTLAHECGHSVHTLFSEQNYPLSQYPIILAE
19714448_Fusobacterium_nuclea       INIYDPYMLLNYQETMDDVFTLAHELGHTLHSMLSSEAYSTADYTIFVAE
65317438_Bacillus_anthra            SSPYGPYILITWQNTMRGCFTLAHEFGHAGHFYLANKNIMNVRPSMYFVE
27467983_Staphylococcus_epider      ASPYISYVFISWTGKMAETFVLAHELGHAGHFALAQNHLLESEASMYFVE
34483139_Wolinella_succin           HGTVPPYVLLNHTDGRRDIFTMAHELGHAIHQELSKGVCLNADTPLTTAE
57240464_Campylobacter_lari         HSSTPPFILLNYTNQRRDLFTLAHELGHTIHQKLSYKVFLNQDTPLTTAE
77740978_Rhodopseudomonas_palu      HPTTPPYVLMNYQGKPRDVMTLAHELGHGVHQVLAAKNALMAPTPLTLAE
15075820_Sinorhizobium_melilo       HPTVPPYVLVNYMGKPRDVMTLAHELGHGVHQVLAGAQALMASTPLTLAE
78193764_Geobacter_metall           MGMTPPYVLLNFTGNLRDVATLAHELGHGLHFVLAQKQMLNYHAPLPLAE
                                         .  : .         .:.** **  *   :             .*

33235980_Chlamydophila_pneumo       IASTFNEMLLMEALSKIVIITKTLDTIFATLFRQTFFAAFEYEIHLTEEF
15835007_Chlamydia_murida           IASTLNETLLMEFLLKIAILSRSLDTIFATLFRQTLFADFELEAHLTEEF
46400453_Protochlamydia_amoebo      VASTFNEDLLSRLLIKIFLVNQKVEDIRATLFRQTMFAEFELLIHLTPQL
CM3763_Cyanidioschyzon_merolae      VASTFNEELYSRYLFKAYLVNRSLEDLRGTLFRQAMFADFELRIHLTPAT
87310907_Blastopirellula_marin      VASTFNEQLLSQYMQRAYLVNRDIDAIRGTIIRQTMFAEFEKITHLTVRS
32446842_Rhodopirellula_baltic      VASTFNEQLLTHHLLRAYLINNELDSIRATVVRQTMFAEFEKKTHLTVAS
2688150_Borrelia_burgdo             IASIINEQILAEYLLIKYIKLTQIDDMISTFFRQTMFAEFEYIIHVVKET
41819184_Treponema_denticola        VASTFNEELLFRHMLRAYLLSVRASDILATLYRQTMFAEYEKITHLTVEN
65320776_Bacillus_anthra            VASTVNEVLLIHHLLRNHLVNHFIEKFKGTFFTQIMFAEFEKITHLNAQV
28211583_Clostridium_tetani         VASTTNECLLSNYMIKLYFINQQLEQIRTTVFRQVMFAEFERITHLTSYD
76260026_Chloroflexus_aurant        VASNFNQALVRDYLFQIAIIEEAMSNFHRYFFIMPTLARFELAIHLTATI
66798507_Deinococcus_geothe         VASNFNQAMVRSHLLEVALIEEALANFHRYFFIMPTLAAFELESYLSAPD
76259709_Chloroflexus_aurant        VASTLNEALLTHYMLRRRLLAQQIEEIRGTIFRQTMFAAFELWMHLTADA
55379750_Haloarcula_marism          VASTVNETLLTHHLLRRHILNEYLERFRSTLYRQTMFAEFEHRTHLTPDR
15790868_Halobacterium_sp.          VASTVNEALLVNHLLRRHILNEYLERFRSTLYRQTLFADVEQRLHLTPDR
68194738_Enterococcus_faeciu        IASTTNENILTEYLLRAYVLNHYLDGFKGTVFRQTQFAEFEHFMHLTSEY
16414804_Listeria_innocu            VASTTNENLLTDYLLRAYLLNHYLDGFKGTVFRQTQFAEFEHAIHLTADY
65318570_Bacillus_anthra            VASTCNEALLNDYLLRLYLLNHYLEGFRGTVFRQTMFAEFEHIIHVTPDM
56379199_Geobacillus_kausto         VASTCNEALLNDYLLRLYLLNHYLEGFRGTVFRQTMFAEFEHLIHLTAET
68056395_Exiguobacterium_sibir      VASTTNEALLNDYLLKLYLLNNQLETFRGTLFRQTMFAEFEHQIHLTPEF
27467608_Staphylococcus_epider      VASTCNEALLSDYMDRLLLLNQELERFRATLFRQTMFAEFEHKIHLTPNR
66856279_Anaeromyxobacter_deha      VASTLNENLLFRRAIRLALLGNRLESLRTTLFRQTMFAEFELAIHLTGER
56380195_Geobacillus_kausto         VASTANEWLMLDYLYKLRLLIEQIEQIRGTLYTQVMYSEFERIIHLTADE
```

```
83319855_Mycoplasma_capric           VASTINEHLLFDYMYKIYLLQERIFNLVSTFYRQIQFADFEYRASLTSEV
19714448_Fusobacterium_nuclea        VASTFNERLILDYMLKIALLEQALGNIVGTYYIQTLFATYEYEAHITPDI
65317438_Bacillus_anthra             APSTMNELLLAQHLLRWVIL-QLLGTYYHNFVTHLLEGEYQRRVYLTATT
27467983_Staphylococcus_epider       APSTMNEMLMANYLFRWVIGSILSRTYYHNMVTHLLEAAYQREVYLTAPL
34483139_Wolinella_succin            TASVFAEMLLFDKMKKRTLYAGKLEDIFATLFRQSVFTNFERLIHIKAEE
57240464_Campylobacter_lari          TASVFAEMLIFDYIKLLSLYAAKIEDIFATLYRQINFTTFERRFHLSAQE
77740978_Rhodopseudomonas_palu       TASVFGEMLTFKRLLRQALLAGKVEDMINTVVRQIAFYSFERAIHLTAER
15075820_Sinorhizobium_melilo        TASVFGEMLTFRALLRKAMLAQKVEDMINTVVRQIAFYDFERKVHLTAED
78193764_Geobacter_metall            TASVFGEMLLTRFLLKIALLCAKIEDIIATTFRQNVLTRFEERMHLTSSR
                                      .*   : :         .                     :    :

33235980_Chlamydophila_pneumo        LSATYGNLQKEFYGSALEWARIPHFYYNFYVVQYATGIIAALSFAEKILF
15835007_Chlamydia_murida            FSQSYKKLQHIFYGSSIEWARIPHFYYNFYVVQYATGIIASLCFSEKILF
46400453_Protochlamydia_amoebo       LKQEYRKLNHKYFGIDIEWARIPHFYYNFYVFQYATGISAALAIADKVVF
CM3763_Cyanidioschyzon_merolae       LRQTYRTLNEEWFGIEWELFRIPHFYYNFYVVQYATGISAALALAERVLF
87310907_Blastopirellula_marin       FQEAYLELLKKYFGLSLECFRIPHFYRAFYVVKYATGLSAAIALSKRVLF
32446842_Rhodopirellula_baltic       FRAAYRELLDAYFGLELECFRIPHFYRAFYVVKYATGLSAAVALSRRVLF
2688150_Borrelia_burgdo              LTETYMNLLKKYFGSPLECLRIPHFYSPFYVVQYATGIAAALSIYKGIKF
41819184_Treponema_denticola         LRSEYRKLLELYFGSDLEGMRIPHFYRAFYVVKYSTGISASMALAERVCF
65320776_Bacillus_anthra             FSDVYENLFREYNGVKYGWARIPHFYRPFYVVKYATGFASAIQIADKLLF
28211583_Clostridium_tetani          LCSIWHDLNLKYFGIDIEWSRIPHFYSPFYVVQYATGYAAANSFATKILF
76260026_Chloroflexus_aurant         FNDLMADLFAEGYGVGNTWAQFSTHYANFYVVQYATGIAGAHALAAPIIF
66798507_Deinococcus_geothe          LIQLTADLLQEGYGSGITWAQFSTHYANFYAYQYATGISAAHQLLAQFEF
76259709_Chloroflexus_aurant         LSQYYRDLVIRYHGLAYEWMRIPHFYYNFYVVQYATGISAALALSRQIIF
55379750_Haloarcula_marism           LDDLYRDLKGDYYEIAREWMRIPHFYRAFYVVQYATGISAAVALVDGILF
15790868_Halobacterium_sp.           IDEVYGDRKRAYYAIEREWMRIPHFYYNFYVFQYATGISAAVALVEDILF
68194738_Enterococcus_faeciu         LSDSYGKLNAKYYGIKFEWSRIPHFYYNYYVFQYSTGFSAASALAKKILY
16414804_Listeria_innocu             LTETYFDINKKYYGIGYEWSRIPHFYMNYYVFQYATGFSAASALSAKILF
65318570_Bacillus_anthra             LTEIYYDLNKKYFGIGLEWSRIPHFYYNYYVYQYATGFSAATALSKQILF
56379199_Geobacillus_kausto          LSALYYDLNKTYFGIGLEWARIPHFYYNYYVYQYATGFSAATALSKQILF
68056395_Exiguobacterium_sibir       LTKTYYALNKTYFGIGLEWARIPHFYYNYYVYQYATGISAAAALTSQILF
27467608_Staphylococcus_epider       MNEEYAKLNKLYFGISKEWSRIPHFYMNYYVYQYATGYSAAQSLSHQILF
66856279_Anaeromyxobacter_deha       MSALYLGLVRKYYGYGAEWTYIQHFHYDFYVYQYATSLVASTAIARAIRM
56380195_Geobacillus_kausto          LNNLWLGLLKTYYGSALGWLRIPHFYDAFYVVKYATSLAASYAIVSDIQF
83319855_Mycoplasma_capric           LNNLFKEVEDDYGYSGYGWPRISHFHSPFYVVKYAIDVTASYKLYDDIKF
19714448_Fusobacterium_nuclea        LSEIMFNLFKKYFGQKIIWARIPHFNSPFYVVQYATSFASSAKLYEDLKL
65317438_Bacillus_anthra             LTEIKTNVLSTFWGAGLTWMRQPHYYMGLYSYTYSAGLTASTAVAQMIKV
27467983_Staphylococcus_epider       LNEIMLNTYKAFFGVELTWMRQPHYYMGLYSYTYSAGLTIGTVVSQCIKT
34483139_Wolinella_succin            FNHLWMEENQKMFGYASWWSYIPHFHTPFYCYAYSYGQLLVLALFGLYKF
57240464_Campylobacter_lari          LSEIWLEESRKMFQYGLWYSYIPHFHSPFYCYAYAYAQLLVLALYGLYKF
77740978_Rhodopseudomonas_palu       IGQIWLSVQSESLGYETFWMYIPHFHSPFYVYAYAFGDCLVNSLYAVYEM
15075820_Sinorhizobium_melilo        LGRIWLSVQSESLGYETYWAYIPHFHSPFYVYAYAFGDCLVNSLYAVYQM
78193764_Geobacter_metall            LCDLWWEENGKLYGYRWGWSYISHFHARFYCYSYTFAELLVLSLYRKYLI
                                     :                       .    * : *:        .

33235980_Chlamydophila_pneumo        LKSGRSDFPLNILKKSGLDMTTSAPLDKAFAFITKKIDLL
15835007_Chlamydia_murida            LQSGGSDFPIEILKKSGLDMTSSAPMLKAFSYIERKLDEL
46400453_Protochlamydia_amoebo       LKGGCSQYPIEMLKMAGIDMESPLPVKSTIQKFDQLLNEL
CM3763_Cyanidioschyzon_merolae       LRAGSSVDPIDALRIAGVDMTSPEPVQAALRRFGALVDEL
87310907_Blastopirellula_marin       LKGGCSKYPLDLLRDAGVDMEQPTAVETALTHFEGLVDEL
32446842_Rhodopirellula_baltic       LRGGCSQDPLDLLRGAGVDMTQPEAVKTTLDHFASLSKQL
2688150_Borrelia_burgdo              LKTGGSKYPLDSLNITGVDLTKKATIENTINIFKCRLEEI
41819184_Treponema_denticola         LKSGGSRYPIESLKLAGVDMASPAPVQAACDNFAKIVDEL
65320776_Bacillus_anthra             LKGGSSDYPLNLLKKAGVDLTTPEPIESALNRFSELVEEF
28211583_Clostridium_tetani          LKSGDNDYPINILKKACIDMTTSKPMEDIVKRFNDLLDML
76260026_Chloroflexus_aurant         LKAGGSRFPLDILRRAGVDLASPEPVERTFAVMASYVDRL
66798507_Deinococcus_geothe          LKSGGSLDPIDALKQAGVDMLSPAPVEATFRVLSGYVDRL
76259709_Chloroflexus_aurant         LRSGSSRSSIDLLRDAGVDMTSPEPIQAAMDTFAGLVDQL
55379750_Haloarcula_marism           LRSGSRQYPLELLRDAGVDMASPDPVESALSTYSDYLDEF
15790868_Halobacterium_sp.           LTRGSSAYPIELLQDAGVDMSTSAPIERAIGVYDHYLDEA
68194738_Enterococcus_faeciu         LKAGNSDYPVEVMKKAGVDMTQAAYIEDAMSMFEQRLNEL
16414804_Listeria_innocu             LKAGSSDYPIDVLKKAGVDMATPNPVDDALKVFEQRLDEL
65318570_Bacillus_anthra             LKAGSSDYPIEVLKKAGVDMASPEPVKEALQVFEEKLNEL
56379199_Geobacillus_kausto          LKAGSSDYPIEVLKKAGVDMTSAEPIRQACQVFAEKLDEM
68056395_Exiguobacterium_sibir       LKAGSSDYPIEVLKAAGVDMTTKAPVEAALRQFERVLDEF
```

```
27467608_Staphylococcus_epider      LKKGSSNYPIEILKNAGVDMTTPQPIEEACEVFEQKLDAF
66856279_Anaeromyxobacter_deha      LAAGGSRYPVDLLREAGVDMTTPAPFAAAMEEMNATMDEM
56380195_Geobacillus_kausto         LRSGASDDPIRLLKRAGVDMTSPEPIERVLRHFTALVDEL
83319855_Mycoplasma_capric          LKAGGHKEPLKIMLDAGIDFNKEQTYLPLINGISDYIKEL
19714448_Fusobacterium_nuclea       LKSGGNNHPMEQLKLAGVDLTKKESFDAVAKEFDRLLDIL
65317438_Bacillus_anthra            LRAGGTMKPLELMKHAGVDMSKPDAIRKAVSYVGSLIDEL
27467983_Staphylococcus_epider      LQAGGSQSPIELAQIAGVDITTDAPLKETINYISNLVDEL
34483139_Wolinella_succin           LSLGGSKRPAELVGIFGFDIEDSEFWNIGIAEVRSLLEEF
57240464_Campylobacter_lari         LSSGGSKSPKELVAMFGFDIESDEFWNIGLEQVRILVDDF
77740978_Rhodopseudomonas_palu      LSAGGTKHYSELLKPFGLDARDPQFWDGGLSVIAGMIDEL
15075820_Sinorhizobium_melilo       LKAGGTKHHSELLAPFGLDATDPSFWAQGLSMIEGLIDEL
78193764_Geobacter_metall           LESGGSLSPADTVKPAGIDLADPHFWQKGYDFLAELIEEL
                                    *   *             .*                  .
```

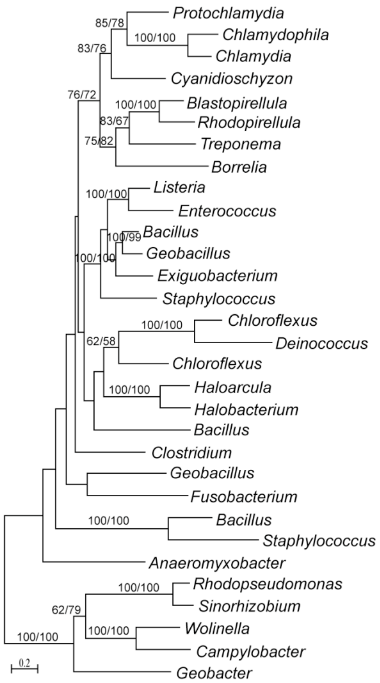

Figure 6. Phylogeny of Oligoendopeptidase F, showing that red algal and chlamydial sequences form a monophyletic group. No significant hits were found in GenBank non-redundant protein sequence database using red algal and chlamydial (*Chlamydophila* GI 15618060, *Protochlamydia* GI 46400453) sequences as queries.

---

7. 4-diphosphocytidyl-2-C-methyl-D-erythritol kinase (*isp*E)

CLUSTAL X (1.83.1) multiple sequence alignment

```
77741291_Rhodopseudomonas_palu      EQARAKVNLTLRVIGRRVDGYHELESVVVFADCADGLTLNLVIKAARLLK
14026931_Mesorhizobium_loti         WHAHAKINLALHVTGRRADGYHLIDSLAVFTRFGDRVEINLVLKARDALP
30180818_Nitrosomonas_europa        FPAPAKLNLFLHVIGRREDGYHLLQTVFRFIDHSDRLHFDLCVRAAKLLL
47573077_Rubrivivax_gelati          VPAPAKLNLFLHVTGRRDDGYHLLQSVFALIDWADTLHFDLCLRAARLLL
28868320_Pseudomonas_syring         LPAPAKLNLMLHILGRRPDGYHELQTLFQFLDYGDELGFNLIVKAARALL
46133381_Haemophilus_influe         FPSPAKLNLFLYINGKLPNGYHELQTLFQFLDFGDWLDINLIYRAAKLLL
50877624_Desulfotalea_psychr        MRAPAKVNLSLQVLSRRADGYHDLATHMQKVSLYDELELNLAVRAALAYR
52216746_Bacteroides_fragil         TFPNAKINLGLNITEKRPDGYHNLETVFYPIPLEDALEINLVVKAYLLLP
67939927_Chlorobium_phaeob          VKAFAKINLGLLITGKRQDGYHTLETVFSPINWYDELTFNLCLKAAKALK
4981944_Thermotoga_mariti           LVSYAKLNLYLDVLGKRSDGYHEIVGLFQTISLHDTLTVNTIKRAWEMFF
```

```
67934269_Solibacter_usitat          LRALAKINLDLRVLGKRPDGFHELRTIFQTISLADTLEINLVVRAARMVG
3322649_Treponema_pallidum          LRAHAKVNMHLWVGARRADGLHSIESVMQRITLADSLSLNTLTRAYARFD
47096183_Listeria_monocyt           ITAPAKINLSLDALYKREDGYHEVEMVMTTIDLADRLYLNLIYQAALLLM
10172673_Bacillus_halodu            VKAPAKINLSLDVLHKRPDGYHEVDMIMTTVDLADRIDLNLAYQAAALLE
17132325_Nostoc_sp.                 LIAPAKINLYLEIIGDRPDGYHELVMILQSIDLADEIEINLVYRAAELMG
71676002_Trichodesmium_erythr       LIAPGKINLYLEIIGDRPDGYHELAMVLQSISLADKIHINIAYRAASLMG
67924114_Crocosphaera_watson        LIAPAKINLYLEIVGDRPDGYHELVMILQSIDLGDRLEINIAYKAAQLMG
35210662_Gloeobacter_violac         LRAAAKINLYLEILGVRPDNFHELVMVLQSVDLADTVTLNLAVRAVEVLE
73659963_Dehalococcoides_sp.        LLAPAKVNLSLEVLYRRKDGYHELRSIIQSLSLCDRLSFSLVSKAVELFT
33632756_Synechococcus_sp.          VTAPAKVNLHLEVLGIRSDGFHELAMVMQSIDLADRLSFNLVIRAAQLLL
84518232_Prochlorococcus_marin      VYASAKINLHLEVLGLRKDGFHELAMVMQSIDLVDEIEINLIIKAAKLIL
54018383_Nocardia_farcin            VRAPSKVNLHLGVGDLRPDGYHDLTTVFQALSLGDDLRINLVWKAAVRLP
29607239_Streptomyces_avermi        VRVPAKVNVQLAVGGARPDGFHDLANVFLAVGLYDEVTANLAARAAIALP
6460432_Deinococcus_radiod          HFAPAKINLGLSVLGVRENGYHDLHSLMVPLTVGDELEINLVYRAARAYG
87310106_Blastopirellula_marin      VHAPAKLNLFLELLGKRSDGFHELETIMTAINVFDTLRFNLVYRAVDLVR
2983445_Aquifex_aeolic              VLSPAKINLGLWVLGRLPSGYHEILTLYQEIPFYDEIYINLVYKGLREFI
29834922_Chlamydophila_caviae       FFSPAKLNLFLKLHGKTSRGFHEMTTQYQVIDFGDTLCLNLVWKSLQIFD
15834807_Chlamydia_murida           FFSPAKLNIFLQLLGKREDGFHEIVTRYQAVSFGDQLSLNSIWKSTALFD
166959_Chlamydomonas_reinhard       LFSPSKINLFLRVVRRREDGYHDLASLFHVIDLGDYMEFNLVIKALNLYM
34908970_Oryza_sativa               LFSPCKINVFLRITGKRPDGFHDLASLFHVISLGDTIKFNLIIKALNLYN
46400864_Protochlamydia_amoebo      LFSPAKINLFLKVIGKRADGYHELSSLFQTISAGDILTFNLVLKAMRLFL
CM4233_Cyanidioschyzon_merolae      LYSPSKINVFLRVIRRRPDGFHELATVMQAISLVDRIGFNLIIRALDTFK
19713437_Fusobacterium_nuclea       IFSNAKINIGLNVFQKESDGYHNIDSIMAPIDLSDEMDVNILYKTYKIFE
45599560_Leptospira_interrog        MISPAKINLGLEIPFKRLDGFHEIRSVFLKISWGDDIEINILYKTFIKAG
                                         *:*: *        . * :          * :  .   :

77741291_Rhodopseudomonas_palu      TGGFSLDKQLPIAAGIGGGSADAAAALRLLARSNGIALMEAAKRTGADVP
14026931_Mesorhizobium_loti         PVSIKLEKNLPIASGVGGGSSDAAAVLRGLAQTWALDIARIGLSLGADVP
30180818_Nitrosomonas_europa        GVKIHLEKNIPLGGGLGGGSSDAATTLIALNRLWGINWMALGLELGADVP
47573077_Rubrivivax_gelati          GADISIDKDVPWGAGLGGGSSDAASTLLALNRLWGLHWLALGLQLGADVP
28868320_Pseudomonas_syring         GMDIWLEKRLPMGGGIGGGSSDAATTLLGLNHLWQLGWAQLGLTLGADVP
46133381_Haemophilus_influe         GANIHLDKILPMGGGVGGGSSNAATALVSLNYLWQANLAKLGLTLGADVP
50877624_Desulfotalea_psychr        GVRISLEKNIPVGAGLGGGSSDAGTVLRGLNQLLDNEFIEMARPLGADVP
52216746_Bacteroides_fragil         PVDIYLYKHIPSGAGLGGGSADAAFMLKLLNEKFNLHLEEYAAILGADCA
67939927_Chlorobium_phaeob          GVAITLTKRIPFGAGLGGGSSDAATTLRVLNALWELDVHGIATGLGADVP
4981944_Thermotoga_mariti           GLKVTLKKEIPVGSGLGGGSSNAAAVLRYLGEVFKIPLLNIAAQVGSDVP
67934269_Solibacter_usitat          RIEMRLTKRIPMGAGLGGGSSDAAAVLLALPVLAGRVLSHIGEQLGSDVP
3322649_Treponema_pallidum          GVRVRVVKRIPAGSGLGGGSADAAALLCGLDTLFGTTLREVAYSVGSDVP
47096183_Listeria_monocyt           GVRITIDKHIPVSAGLAGGSSDAAAALKGLNVIWELGLAEISSEIGSDIA
10172673_Bacillus_halodu            GVSIYITKRIPVAAGLAGGSSDAAATLRGLNDLWQLGLAVLGAEIGSDVS
17132325_Nostoc_sp.                 GVDITVHKHIPVAAGLAGGSTNAAAVLVGIDLLWNLGLEELGSTLGSDVP
71676002_Trichodesmium_erythr       GVEITINKYIPMAAGLAGGSTNAAAVLVGLDLMWELGLQELGARLGSDVP
67924114_Crocosphaera_watson        GVNITIDKQIPVAAGLAGGSTDGAAVLVGLNLLWELGLQKLGEKLGSDVP
35210662_Gloeobacter_violac         GVEIVIEKRIPVASGLAGGSTDAAAVLAGLNVLWDLGLQSLGAQIGSDIP
73659963_Dehalococcoides_sp.        GVNLKIAKRIPLVSGLGGDSSCAAAVLKGLNKLWGCGYMEIGAELGSDVP
33632756_Synechococcus_sp.          GAAIHLEKRIPIGAGLAGGSSDGAAALVGLNALWGLGHQRFAAELGSDMP
84518232_Prochlorococcus_marin      GALIYLRKKIPIGAGLAGGSSDGAATLVGLNSLWGLNFEDMAAELGSDVP
54018383_Nocardia_farcin            LVEIVIDKGIPVAGGMAGGSADAAAALVGLNELWDMGLSALAAELGSDVP
29607239_Streptomyces_avermi        DVHLHIAKDIPVAGGMAGGSADAAGALLACDTLWGTNALDICAELGSDVP
6460432_Deinococcus_radiod          GADLVLHKRLPLASGLGGGSSDAASTLLALAELYPAPDPALALTLGADVP
87310106_Blastopirellula_marin      GAKIELVKRIPSASGLGGASSDAAAALLAADIVWKLGWTELAAQLGSDIP
2983445_Aquifex_aeolic              NYSIFIQKNIPPGAGLGGGSSNLAVVLKKVNELLGSPLRELVGSISADAP
29834922_Chlamydophila_caviae       PVAWRLHKRIPIGAGVGGGSSNAATALYALNEHFQTQLQELGKKIGMDVP
15834807_Chlamydia_murida           PVSWRVVKHIPIGSGLAGGSSNAATALFALNQMFQTGLRSLAEKVGMDTP
166959_Chlamydomonas_reinhard       YYKVKLHKRVPHGAGLGGGSGNAATTLFAANKLMGGIALEWSGEIGSDIS
34908970_Oryza_sativa               FFWIHLDKKVPTGAGLGGGSSNAATALWAANQFSGCIAQEWSGEIGSDIP
46400864_Protochlamydia_amoebo      HLRIHLDKRLPSQAGLGGGSSNAATTLWACNQLAGEIVMQWGSEIGADIP
CM4233_Cyanidioschyzon_merolae      HYRVHLDKQIPTEAGLGGGSGNAATALFAANRLCGEPAIRWASEIGSDTA
19713437_Fusobacterium_nuclea       KIDIILKKNIPSEAGLGGGSSNAGFFLKLLNKHYGNVYEKLAMRVGSDVP
45599560_Leptospira_interrog        VKIHLTKRISP-AGGLGGGSTNAASLLNFL-FSWCPFFFVLAAEIGSDVP
                                           :  *  .*:.* *   .  *                  . * .

77741291_Rhodopseudomonas_palu      VCVAASCIMGGIGEKLTPLVNDLEAPAKQVQPVVGEVIALLMSGSGATCF
14026931_Mesorhizobium_loti         MCLAKPLVARGIGDELSMVRNDLEPAALALQPAIGRALSWLMSGSGATCF
30180818_Nitrosomonas_europa        VFIYRNAFAEGVGEELHAVHNDLEPVAMRMQPVIAGWLGWLMSGSGSCMF
```

```
47573077_Rubrivivax_gelati          FFVGHNAWVEGIGERLTPIHNDLQTPAMAYSDEVVQALALLMSGSGSAVF
28868320_Pseudomonas_syring         VFVRHAAFAEGVGEILTPVRNDCKAAVERRYPEVRNALNLLLTGTGSCVF
46133381_Haemophilus_influe         IFVHHAAFAEGVGEKITYCKNDCEKVVINHYSNVEKALNWLLTGTGACVF
50877624_Desulfotalea_psychr        FFASSAAFATGIGDILQPLHNDLEEIVSDLCPAIDEMKSLLMSGSGSTVF
52216746_Bacteroides_fragil         FFIKKPTFAEGIGNIFSPVFNDFEKSVFPQYPVIEEIKKELMSGSGSSVF
67939927_Chlorobium_phaeob          YFLEGLAYATGIGEILEDLENDFEPAVFDHYPAVRKVKEQLLSGSGSAVF
4981944_Thermotoga_mariti           FFLYGTALVRGRGEIVEKLYNVFEKVFLEKHPEVMDGLRNFMTGSGSVFF
67934269_Solibacter_usitat          FFLLGAATGIGRGSELFPLRNDFEAVVFEQHPKLAAIKRRLMSGSGSALY
3322649_Treponema_pallidum          FFLAQAACVLGGGEQLVPLFNSLDAPVQRRYPVVALARWDLMSGSGSXVF
47096183_Listeria_monocyt           FCVYGTALATGRGEKISALGNVLESVTLEKNPQVKRIKDRMMSGSGPTVF
10172673_Bacillus_halodu            FCVYGTARATGRGEIIERIHNVLESVTLDLYPEVRNIKEQMMSGSGPTVF
17132325_Nostoc_sp.                 FCVAGTVIATGRGEQLSPLHNDLEKVVLPSYPQVLHLRELLMSGSGPSVF
71676002_Trichodesmium_erythr       FCIGGTALGTRRGEILSPLHNDLEKVALSEYPQLLKLREAFMSGSGPTMF
67924114_Crocosphaera_watson        FCVSGTAIATGRGEKLDAIHNDLEKVVLPEYTQVTQLRDILMSGSGPTVF
35210662_Gloeobacter_violac         FCVTGTALAMGRGEVLSPLHNDLERAVLPAYPQVSALREHLMSGSGPAVF
73659963_Dehalococcoides_sp.        FFIMGTAMMEGRGETVTPLFNAFEKVAFELFPELVKYRWQFLAGAGPTLF
33632756_Synechococcus_sp.          FCVAGSQLCFGRGELLEPLQNDLQEVVEPETPAVQAALRLLMSGSGPSCF
84518232_Prochlorococcus_marin      FCISGAQLCFGRGECLEPLINDLQEVVAPATPAVEKALQFLMSGSGPSCF
54018383_Nocardia_farcin            FALHGTALGTGRGERLLPVGNDLQAAAVSLKPELRRTLRAGVSGSGPTCA
29607239_Streptomyces_avermi        FSLVGAALGTGRGERLRELSNDLQPAALSLFPSLSDTLEAGVSGSGPTTA
6460432_Deinococcus_radiod          FFLLGAALAEGVGERLTPVFNSLQAGVLARVPSVLTTLEALMSGSGATCF
87310106_Blastopirellula_marin      FFLHGLALCRGRGEIITPLHNRLTEPSRQLSTEIDRLTRCMMSGSGSACF
2983445_Aquifex_aeolic              FFLLKSAIGRGKGEVLEPVENVLGDIARELYPEINEVYRFVVSGSGSTVY
29834922_Chlamydophila_caviae       LFFSGSAIGVGCGEEILPYDNDLEKSVFRFRKDLEEKKQMLMSGAGATLF
15834807_Chlamydia_murida           FFFSGSALGLGRGEKVVTLRNDLENPVFRLRLDLKEKKRWLLTGSGATLF
166959_Chlamydomonas_reinhard       VFFSGAAYCTGRGEVVEDVVNDLEQPAFDNLPELAALKQRLMTGSGSTIV
34908970_Oryza_sativa               FFFSGAAYCTGRGEIVEDIVNDLEPPAFEVLPSLKRLKKRIMSGSGSTIV
46400864_Protochlamydia_amoebo      FFFSGTAHCTGRGECVNSLFNDLEASAFEIKPELKILKNTLMSGSGSSFF
CM4233_Cyanidioschyzon_merolae      FFFSGSAFCTGRGEQLHPIVNDLEGPAFALMPPLRHLKTFLMSGSGTSFF
19713437_Fusobacterium_nuclea       FFIKKTARVGGKGNRVDLVENSLEQGILETDTNIKMLKMTLMSGSGSTYY
45599560_Leptospira_interrog        FFLGGHAFVTGKGEILEEIWNDFEPVAFQLHPELGVLKDKFLTGSGSSMY
                                                *. .    *            :       ::*:*.

77741291_Rhodopseudomonas_palu      AIF
14026931_Mesorhizobium_loti         GLF
30180818_Nitrosomonas_europa        AEF
47573077_Rubrivivax_gelati          AEA
28868320_Pseudomonas_syring         GAF
46133381_Haemophilus_influe         AEF
50877624_Desulfotalea_psychr        GVF
52216746_Bacteroides_fragil         GLF
67939927_Chlorobium_phaeob          GLF
4981944_Thermotoga_mariti           ALY
67934269_Solibacter_usitat          GLF
3322649_Treponema_pallidum          GLY
47096183_Listeria_monocyt           ALI
10172673_Bacillus_halodu            GLV
17132325_Nostoc_sp.                 ALC
71676002_Trichodesmium_erythr       ALT
67924114_Crocosphaera_watson        TLC
35210662_Gloeobacter_violac         GLA
73659963_Dehalococcoides_sp.        TLL
33632756_Synechococcus_sp.          ALF
84518232_Prochlorococcus_marin      AIF
54018383_Nocardia_farcin            FLC
29607239_Streptomyces_avermi        FLT
6460432_Deinococcus_radiod          GLA
87310106_Blastopirellula_marin      ALC
2983445_Aquifex_aeolic              FFG
29834922_Chlamydophila_caviae       VSY
15834807_Chlamydia_murida           VRY
166959_Chlamydomonas_reinhard       CFG
34908970_Oryza_sativa               GIG
46400864_Protochlamydia_amoebo      CIG
CM4233_Cyanidioschyzon_merolae      ALG
```

19713437_Fusobacterium_nuclea   TFV
45599560_Leptospira_interrog   GLV

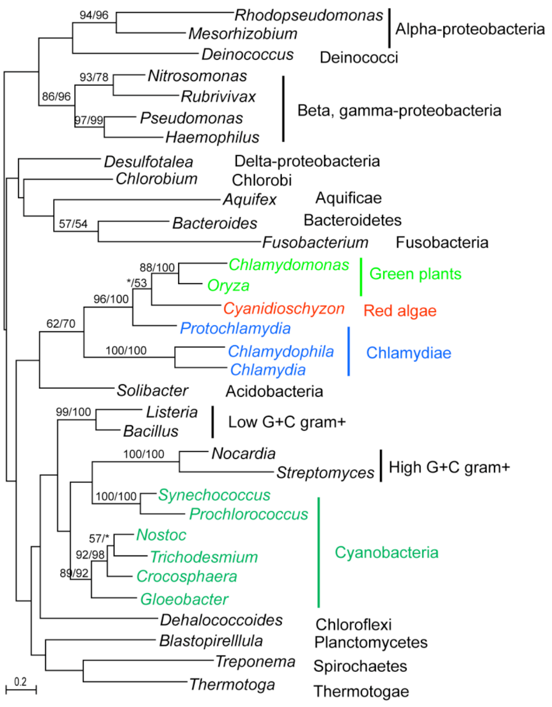

Figure 7. Phylogeny of 4-diphosphocytidyl-2-C-methyl-D-erythritol kinase (*ispE*). Note that sequences of primary photosynthetic eukaryotes form a monophyletic group with those of chlamydiae (in particular *Protochlamydia*) whereas cyanobacterial homologs form another distinct group. This gene is part of the DXP pathway that is mainly limited to bacterial and plastids of photosynthetic eukaryotes.

---

8. Enoyl-ACP reductase (*fabI*)

CLUSTAL X (1.83.1) multiple sequence alignment

48853605_Cytophaga_hutchin   LLKGKRGIIFGALDENSIAWKVAQRAHEEGAVFTLELENLFDKSMEILGK
60491878_Bacteroides_fragilis   LLKGKRGIIFGALNEQSIAWKVAERAVEEGAVITLDLENVFKRSMEVLGQ
67919153_Chlorobium_limico   LLKGKKGIVFGPLDESSIGWQIALHAYQEGAEIAIDVDACFREMKEKIGS
86740599_Frankia_sp.   LLEGKRVLVTGVLTEASIAFSVARIAQEQGAAVVLHLAGLAGQVLEHVDG
66737267_Mycobacterium_tuberc   LLDGKRILVSGIITDSSIAFHIARVAQEQGAQLVLHLASLAGRVTEAIGK
6459755_Deinococcus_radiod   DLSGKTALVMGVANARSLGWAIAEQLLQAGCRVGFDMDALFARVKDEFSE
46199980_Thermus_thermop   DLSGKKALVMGVTNQRSLGFAIAAKLKEAGAEVALELDALFAGVKEAFGG
23128764_Nostoc_punct   NLTGKNALVTGIANNRSIAWGIAQQLHKAGANLGIQIQFTFETIREQWGK
78712168_Prochlorococcus_marin   NLTGKKILVTGIANNRSIAWGIAQQLSKAGAELGIQIEEIFENIKNNWGG
CM4316_Cyanidioschyzon_merolae   DLRGKRALITGIANQRSIAYGIAQQLRRAGAELGVDLEGLFETVKQKWGG
86606338_Synechococcus_sp   DLTGKKALVTGIANERSIAWGIAQQLHKAGATLGVQIDELFQAIAQKWGQ
35214762_Gloeobacter_viol   TLAGKKALITGIANNRSIAWAIAQAFHAAGAALAVQIAALFETIAQKWGQ
32447170_Rhodopirellula_baltic   QFEGKKGLIIGVANDHSIAWAIAQEILKQGGKCGFDIRTIFEHTEKTFGK
67932874_Solibacter_usitat   LLEGKFALILGIANKWSLAYAIAQAFSREGATLVLDLAALTESLQGFQRP
16413428_Listeria_innocu   SLEGKTYVVMGVANKRSIAWAIARSLNEAGAKLVFAITATFETIKDKAGK

```
67916335_Clostridium_thermo         NLLGNKNIVMGVRNKWSIAWGIVKAVQEEGANVIIEINSLFAAIKEKYGV
16765044_Salmonella_typhim          FLSGKRILVTGVASKLSIAYGIAQAMHREGAELAFSIDAMFAELGNVWPK
68058530_Haemophilus_influen        FLTGKRILVTGLASNRSIAYGIAKSMKEQGAELAFSIQNCFAELSKRWDK
68558071_Ralstonia_metall           FLAGKRILITGLLSNRSIAYGIAAACKREGAELAFQIDATFAALGQRWEK
2983915_Aquifex_aeolicus            LLEGKRALITGVANERSIAYGIAKSFHREGAQLAFDIKNLKKFLEENWGS
13476200_Mesorhizobium_loti         LMAGKRGLILGIANNRSIAYGIAKACVDHGAEIALSLDEVFANVAKHWGK
13474637_Mesorhizobium_loti         LMKGKRGLVMGVANDHSIAWGIAKKLSEHGAELAFSVAATFETLGKEWGG
67458967_Rickettsia_felis           LLQGKKGLITGIANNMSISWAIAQLAKKHGAELWFSISNLFDDIKEKWGS
86155412_Campylobacter_fetus        -MNGKKGLIVGVANKMSIAYGIAKQCKEQGASLAFHLDSLASKIKADFEG
76259288_Chloroflexus_aurant        LMEGKKGLILGVANDRSIAWGIAQALHREGATIGFDITALIEQARETFGQ
39982876_Geobacter_sulfur           LLEGKKAVVFGIANEKSIAWAIAQAFRREGADLAIDIAAVFARIERSWGR
68555265_Ralstonia_metall           PLAGARVLVVGVANEDSIAWGCARAFREMGAEIALQMDALFARIAAEWGQ
51857752_Symbiobacterium_therm      LMTGRKCLVMGVANKRSIAWGIARALHREGAELAFQIEAAFAELGRRWGK
50944223_Oryza_sativa               DLRGKRAFIAGVADDNGYGWAIAKALAAAGAEILVTVKEVAESVKNDFGS
18396215_Arabidopsis_thalia         DLRGKRAFIAGIADDNGYGWAIAKSLAAAGAEILVTVQEAAECVKKDFGS
89898427_Chlamydophila_felis        DLTGKVAFIAGIGDDQGYGWGIAKVLAEAGATIIVTISEVVEQVTKDFGH
76167348_Chlamydia_tracho           DLTGKIAFIAGIGDDNGYGWGIAKMLAEAGATILVTVSEVVEQVKKHFGH
32307472_Bigelowiella_natans        DLKGKKAFVAGVADDQGFAWAISKCLVEAGADVSLTISEVANLVEKDIGK
32423792_Phaeodactylum_tricor       -------FIAGIGDSTGYGWAIAKQLAEAGATIIVDIRSCAEAVAKDYGK
46400427_Protochlamydia_amoebo      NLKGKKAFIAGVGDDQGFGWAIAKALAEAGAEIIITISEVCEAIGKDFGK
34555736_Toxoplasma_gondii          DLRGQTAFVAGVADSHGYGWAIAKHLASAGARVALTIKEVAVKVKQDLGN
                                           .: *     . .:         *    . :

48853605_Cytophaga_hutchin          IDFILHSIGMSPRKGKEYGDMNYDWFLKTLDVSALSFHKIMHVAEKKYGS
60491878_Bacteroides_fragilis       IDFVLHSIGMSPRKKRTYDDLDYNMLNTTLDVSAVSFHKMIQAAKKQYGS
67919153_Chlorobium_limico          VDFIVHSIGMSQRKQLPYEDLNYEWFMRTLDVSGLSLHRLVAYALKNNGS
86740599_Frankia_sp.                LDGVLHAIGFAPVLGGPFVEAAWSEVGTALQISTWSLASLTRAALPLKAS
66737267_Mycobacterium_tuberc       LDGVVHSIGFMPGMGIPFFDAPYADVSKGIHISAYSYASMAKALLPIGGS
6459755_Deinococcus_radiod          LHLLVHSIGFAPAMDGRFIDTTADDWNTALNVSAYTLVAAARRAEPLGGS
46199980_Thermus_thermop            LDYLVHAIAFAPAMEGRYIDTRRQDWLLALEVSAYSLVAAAQRAEPLGGG
23128764_Nostoc_punct               LDILIHCLAFASDLSGDFSQTSRSGFSTALEISTYSLVQLSGAAKPLGGS
78712168_Prochlorococcus_marin      IDGLVHCLAFAGELIGDYSATSSEGFDRALNISAYSLAPLCKAAKPLGAG
CM4316_Cyanidioschyzon_merolae      LDLLVHCLAFANDLEGNFSETSRNGFSVALSVSTYSLIEMCGLAKPLGGS
86606338_Synechococcus_sp           VDILVHCLAFVKELSGNFSDISREGFALAMDVSTYSLIALCRAAKPLGSS
35214762_Gloeobacter_viol           LDILVHCLAFGNDLQGPFTGVTRDGYGLAMEVSAFSLIALASQAAPLGGS
32447170_Rhodopirellula_baltic      IDFLLHSIAFADDLGRETMHTSRSGFKLAMESSVFSLLAVTAAAEPIGGS
67932874_Solibacter_usitat          LHAVVHSLAFANDLSRPFMETSRSGFLLAQDVSAYSLVAVARAVAPVGGS
16413428_Listeria_innocu            LSGLAHCIAFANYLTGDYLEVDRKSFLQAHEISAYSFTAVARALKHLDAS
67916335_Clostridium_thermo         LHGLVHSIAHAKDLQNDFIYTSREGFAHAMNISAYSLVAVSRGAKELGGS
16765044_Salmonella_typhim          FDGFVHSIGFAPQLDGDYVNVTREGFKVAHDISSYSFVAMAKACRTMGSA
68058530_Haemophilus_influen        FDGFIHAIAFAPQLDGDYVNATREGYRIAHDISAYSFVAMAQAARPYNAA
68558071_Ralstonia_metall           FDGLVHSIGFAPAIAGNFLDLSREGFRIAHDISAYSFPALAKAAMPLKAS
2983915_Aquifex_aeolicus            LDIIVHSIAYAPEFKGGVIDTSREGFKIAMDISVYSLIALTRELLPLNGA
13476200_Mesorhizobium_loti         LDFLVHAIAFSDELTGRYVETTRDNFLRTMDISVFSFTTIAKRAEALGGS
13474637_Mesorhizobium_loti         LDFVVHAIGFSDELKGLYADTSRDNFVRTMVISCYSFTEVARNAAALGGS
67458967_Rickettsia_felis           FDFLLHGMAFADELKGRYVDTSLENFHNSLHISCYSLLELSRSAEALGGS
86155412_Campylobacter_fetus        IDFIVHAVAYAPALEGEFIDTTKEAFDVAMGTSVYSLISLTKAVLPVGGS
76259288_Chloroflexus_aurant        IDFLVHAIAFANELSGTILNTTREGFRIALEISAYSLIALVKAAEPIDAS
39982876_Geobacter_sulfur           LDTLVHSVAFANELKGSFLNTTREGFSLAMDISAYSLIALAKGAHPLQGS
68555265_Ralstonia_metall           LDSLVHSVAFAPDLQGGLLNSSAAGFARAMDVSCHSFIRMARRAVPLGGT
51857752_Symbiobacterium_therm      LDVVVHSIAYARDLQRDFVDTTREGWRIAQEVSAWSLIAVARAAKPLGGS
50944223_Oryza_sativa               IDILVHSLANGPEVTKPLLETSRRGYLAALSASSYSFVSLLQHFLPIGGA
18396215_Arabidopsis_thalia         IDILVHSLANGPEVSKPLLETSRKGYLAAISASSYSFVSLLRHFLPIGGA
89898427_Chlamydophila_felis        IDILIHSLANSPEISKSLLETSRKGYLSALSTSSYSLVSLLAHFGPIGGS
76167348_Chlamydia_tracho           IDILVHSLANSPEIAKPLLDTSRKGYLAALSTSSYSFISLLSHFGPIGAS
32307472_Bigelowiella_natans        IDILVHSLANGPEVRNPLLETSRKGYLAAQSASAYSLVSMVQKFGPIGGS
32423792_Phaeodactylum_tricor       IDILVHSLANGPEVTKPLLETSRAGYLAASSASAYSLVSMTKHFGEIGGS
46400427_Protochlamydia_amoebo      IDIFVHSLANAPEVQKPLLETSRKGYLAALSSSSYSFVSLLSHLGPYQGS
34555736_Toxoplasma_gondii          IDILVHSLANGPEVTKPLLETSRKGYLAASSNSAYSFVSLLQHFGPIGGS
                                    .  . * :.                       *  :            .

48853605_Cytophaga_hutchin          ILALSYIAAQRVFPDYTDMADAKSTLESIARGYGARFAKLKVRVNTISQS
60491878_Bacteroides_fragilis       IVALSYVAAQRTFYGYNDMADAKALLESIARSFGYIYGREHVRVNTISQS
67919153_Chlorobium_limico          VVALSYIASQRNYWTYSDMGDAKSLLESIARSFGPRLAQRGIRINTISQS
86740599_Frankia_sp.                IVGLDFDASV-AWPSYDWMGVAKAGLASATRYLARDLGPRGVRVNLVAAG
```

```
66737267_Mycobacterium_tuberc       IVGMDFDPS-RAMPAYNWMTVAKSALESVNRFVAREAGKYGVRSNLVAAG
6459755_Deinococcus_radiod          IISLTYHASQQVVPRYNVMGVAKAALEAATRYLAADLGEREIRVNTISAG
46199980_Thermus_thermop            IVTLTYYASEKVVPKYNVMAIAKAALEASVRYLAYELGPKGVRVNAISAG
23128764_Nostoc_punct               IVTLTYLGGVRAIPNYNVMGVAKAGLEMSVRYLAAELGPQNIRVNAISAG
78712168_Prochlorococcus_marin      VVSLTYLGSERAIPNYNVMGVAKAALEASVRYLSAELGPEKVRVNAISAG
CM4316_Cyanidioschyzon_merolae      VVTLSYLGAERVVPNYNIMGVAKAGLECSVRYLAAELGPACVRVNGISAG
86606338_Synechococcus_sp           ILTLTYLGGVRVVPNYNMAAVTKAGLETCVRYLAAELGPAGIRVNAISAG
35214762_Gloeobacter_viol           ILTLTYIGSVRAMSGYGVMGPAKAALEANTRYLAAELGAKNLRVNAVSAG
32447170_Rhodopirellula_baltic      VLTMTYYGGEKCVPGYNVMGVCKAALDASMRYLAFDMGARGVRVNALSAG
67932874_Solibacter_usitat          LSTLTYVGSTRVITNYNVMGVAKASLEAGVRYLASEMGAKGIRVNAISAG
16413428_Listeria_innocu            LLTLTYLGGERVVENYNIMGVAKASLDASVRYLAMDLGAIGVRVNAISAG
67916335_Clostridium_thermo         IITLTYMGSEKVFKGYNVMGVAKAALEASVRYLAADLGEANIRVNAISAG
16765044_Salmonella_typhim          LLTLSYLGAERAIPNYNVMGLAKASLEANVRYMANAMGPEGVRVNAISAG
68058530_Haemophilus_influen        LLTLSYLGAERAIPNYNVMCLAKASLEAATRVMAADLGKEGIRVNAISAG
68558071_Ralstonia_metall           LLTLTYLGAERVVPNYNTMGLAKASLEASVRYLASAVGPRGMRANGISAG
2983915_Aquifex_aeolicus            IVTLSYYGAEKVVPHYNVMGIAKAALESTVRYLAYDIAKHGHRINAISAG
13476200_Mesorhizobium_loti         LLTLTYYGAEKVMPHYNVMGVAKAALEASVRYLAVDLGGKKIRVNAISAG
13474637_Mesorhizobium_loti         MITLTYAGSVRVMPNYNVMGVAKAGLEASVRYLANDYGPRGIRVNGISAG
67458967_Rickettsia_felis           IVTLTYYGAEKVIPNYNVMGISKAALEASVKYLATDLGENNIRVNAISAG
86155412_Campylobacter_fetus        VLTLTYLGGAKFVPHYNVMGVAKAALESSVRYLARDLGKKNIRVNAISAG
76259288_Chloroflexus_aurant        VLTLTYHGARQVIGSYNVMGVAKAALEASVRYLAAGLGPRGIRVNAISAG
39982876_Geobacter_sulfur           IITLSYYGGQKVFPSYNVMGVAKAALEMSVRYLAEAVGPDGIRVNAISAG
68555265_Ralstonia_metall           MFAMSYDGANRVVPNYDLMGPVKAALEASCRYLAYELGPRNIRVHAISPG
51857752_Symbiobacterium_therm      IMTLTYLASERVVDRYNIMAPAKAALECNVRYLAHELGPSNIRVNAISAG
50944223_Oryza_sativa               SISLTYIASERAIPGYGGMSSAKAALESDTKVLAFEAGRKGIRVNTISAG
18396215_Arabidopsis_thalia         SISLTYIASERIIPGYGGMSSAKAALESDTRVLAYEAGRKSIRVNTISAG
89898427_Chlamydophila_felis        SISLTYLAASRAVPGYGGMSAAKAALESDTKMLAWEAGRKWIRVNTISAG
76167348_Chlamydia_tracho           TISLTYLASMRAVPGYGGMNAAKAALESDTKVLAWEAGRRWVRVNTISAG
32307472_Bigelowiella_natans        VLSLTYDASNKVVPGYGGMSSAKASLESDTRTLAYEAGRKHIRVNTVFAG
32423792_Phaeodactylum_tricor       ILSLTFIASERVVPGYGGMSSAKAQLESDTKVLAWEAGRKWIRVN-----
46400427_Protochlamydia_amoebo      ALTLTYLASEKIIPGYGGMSSAKAALESDTRTLAWEAGRKWIRVNTISAG
34555736_Toxoplasma_gondii          AVTLSYLAAERVVPGYGGMSSAKAALESDTRTLAWEAGQKYVRVNAISAG
                                       : :  .      *      *: *    :  .   .    * :

48853605_Cytophaga_hutchin          PTKTTAGKGISGFGAFYDFADLMSPLGSADECAGYSIVLFSDLTRKVTMQ
60491878_Bacteroides_fragilis       PTFTTAGSGVKGMDKLYDFANRMSPLGSADECADYCIVMFSDLTRKVTMQ
67919153_Chlorobium_limico          PTYTKAGSGIPGFDKMYEYSDLMSPLGSAEECAEYTMTLLSDLSRKVTMQ
86740599_Frankia_sp.                PLRTMAAKSIPGFSQFEEVWDGRAPLGDTTPAAQACVALLSDWFPATTGE
66737267_Mycobacterium_tuberc       PIRTLAMSAIVGIQLLEEGWDQRAPIGDATPVAKTVCALLSDWLPATTGD
6459755_Deinococcus_radiod          PMRTIAARSIPHFGKLYNKGAMNAAFGTSEEVGKLALFLLSDLSSGITGQ
46199980_Thermus_thermop            PVRTVAARSIPGFTKMYDRVAQTAPLRTQEEVGNLGLFLLSPLASGITGE
23128764_Nostoc_punct               PIRTLASSAVGGILDMIHHVEEVAPLRTQLEVGNTAAFLCSDLSSGITGQ
78712168_Prochlorococcus_marin      PIRTLASSAIGGILDMIHNVEEKAPLRTQTEVGNTAAFLLSDLSSGISGQ
CM4316_Cyanidioschyzon_merolae      PIRTLASSAIGGITTMIRNVEQNAPLRTQEEVGKTAAFLLSDAASGITGQ
86606338_Synechococcus_sp           PIRTLASSAIADFHAMLHAVEEKAPLRTQLEVGNVAAFLASDLASGITGQ
35214762_Gloeobacter_viol           PIRTLAASGVGNIHEAIHKTEEMSAIKRQEEVANTVLFLASDLASAITGQ
32447170_Rhodopirellula_baltic      PIRTLAGRAAG-VEEMLTMYEHMAPMGTHEEVGKSGAFLLSDASSGISGE
67932874_Solibacter_usitat          PIKTVSARAVKDFSTVLEAIQTRAPLKDASEVADTAVFLASDLGRGVTAN
16413428_Listeria_innocu            PIRTVSARGVSGFSDSISLVEERAPLKQAEEVGDTAYYLFSNLSRGVTGE
67916335_Clostridium_thermo         PVKTLSAKGVKNFGDILDAVPKKAPLKTQEDLGKTALYLLSDLSSGVTGE
16765044_Salmonella_typhim          PIRTLAASGIKDFRKMLAHCEAVTPIRTIEDVGNSAAFLCSDLSAGISGE
68058530_Haemophilus_influen        PIRTLAASGIKNFKKMLSTFEKTAALRTIEDVGNSAAFLCSDLASGITGE
68558071_Ralstonia_metall           PIKTLAASGIKDFSRLLKHFEDVAPIRTIEEVGNVAAFLLSDLASGVTGE
2983915_Aquifex_aeolicus            PVKTLAAYSITGFHLLMEHTTKVNPFGTIEDVGDTAVFLCSDWARAITGE
13476200_Mesorhizobium_loti         PIKTLAASGIGDFRYILKWNEYNSPLKTQEEVGDSGVYFLSDLSRGVTGE
13474637_Mesorhizobium_loti         PVRTLAGAGISDARHMFSYQQRNSPLRTIDEVGGSALYLLSDLASGVTGE
67458967_Rickettsia_felis           PIKTLASSAIGDFSTMLKSHAATAPLKTQEDVGGAAVYLFSNLSKGVTGE
86155412_Campylobacter_fetus        PIKTLAASGIGDFKMILKWNECNAPLKSIDDVGKSGMYLLSDLASGVTGE
76259288_Chloroflexus_aurant        PIRTLAASGIANFRSLHKHFAEYAPLRTIEDVGNAALYLCSPLAAGVTGE
39982876_Geobacter_sulfur           PLKTLAAAGVGGFNQIAGHVAEKAPLRSQEEVAGAALYLASDLSSGVTGE
68555265_Ralstonia_metall           PLKTRAASGLKDFDALLAEAAGRAPLGDIMDVGFATAYLATPYARRLSGN
51857752_Symbiobacterium_therm      PLKTLAASAVKGLSTIRDIVEERAPLRTLEEVGDVGLFLASPLSRCITGE
50944223_Oryza_sativa               PLGSRAAKAIGFIEKMIEYSYVNAPLQLADEVGNTAAFLVSPLASAITGS
18396215_Arabidopsis_thalia         PLGSRAAKAIGFIDTMIEYSYNNGPIQTADEVGNAAAFLASPLASAITGA
89898427_Chlamydophila_felis        PLASRAGKAIGFIEKMVDYYLDWSPIPTTEQVGAAAAFLVSSLASAITGE
```

```
76167348_Chlamydia_tracho          PLASRAGKAIGFIERMVDYYQDWAPLPEAEQVGAAAAFLVSPLASAITGE
32307472_Bigelowiella_natans       PFGSRPARAIGFIERVINYSYKNAPIESAMEVGQTAGFLLSPLAFAITGQ
32423792_Phaeodactylum_tricor      ---------------------------TSQQV------------------
46400427_Protochlamydia_amoebo     PLRSRAARAIGFIDRMINYSQVNAPLQKACEVANVAAFLGSDLACAITGS
34555736_Toxoplasma_gondii         PLKSRAASAIGKIDYAIDYSYNNAPLRHSDDVGGAALFLLSPLARAVSGV


48853605_Cytophaga_hutchin         NLMHDGGFSSTGM
60491878_Bacteroides_fragilis      NLFHDGGFSSVGM
67919153_Chlorobium_limico         NLFHDGGYSSMGA
86740599_Frankia_sp.               IVHVDGGVHAVGI
66737267_Mycobacterium_tuberc      ILYADGGAHTQLL
6459755_Deinococcus_radiod         TIYVDAGLSIMTV
46199980_Thermus_thermop           VVYVDAGYHIMGM
23128764_Nostoc_punct              VLYVDAGYEIMGM
78712168_Prochlorococcus_marin     TIYVDAGYCINGM
CM4316_Cyanidioschyzon_merolae     ILYVDSGYVIMGS
86606338_Synechococcus_sp          VIYVDSGYSILGF
35214762_Gloeobacter_viol          TIYVDCGFSIMGG
32447170_Rhodopirellula_baltic     ILHVDGGYHAMGS
67932874_Solibacter_usitat         CLFVDAGMQVLGF
16413428_Listeria_innocu           VIHVDSGYHIIGF
67916335_Clostridium_thermo        VIHVDSGYNIMGI
16765044_Salmonella_typhim         VVHVDGGFSIAAM
68058530_Haemophilus_influen       IVHVDAGFSITAM
68558071_Ralstonia_metall          ITYVDGGYNIVGT
2983915_Aquifex_aeolicus           VVHVDNGYHIMGV
13476200_Mesorhizobium_loti        VHHVDSGYHVVGM
13474637_Mesorhizobium_loti        IHYVDSGYHIVSM
67458967_Rickettsia_felis          IHYVDCGYNIMGS
86155412_Campylobacter_fetus       IHYVDAGYNIMGM
76259288_Chloroflexus_aurant       IHYVDAGFNVVVP
39982876_Geobacter_sulfur          VHFVDSGYNIIGL
68555265_Ralstonia_metall          TVYVDGGVHVMA-
51857752_Symbiobacterium_therm     TIYADNGFHIMGV
50944223_Oryza_sativa              TVYVDNGLNTMGL
18396215_Arabidopsis_thalia        TIYVDNGLNAMGV
89898427_Chlamydophila_felis       TLYVDHGANIMGI
76167348_Chlamydia_tracho          TLYVDHGANVMGI
32307472_Bigelowiella_natans       VVYVDNGLSPMGL
32423792_Phaeodactylum_tricor      -------------
46400427_Protochlamydia_amoebo     TIYVDYGLHAMGV
34555736_Toxoplasma_gondii         TLYVDNGLHAMGQ
```

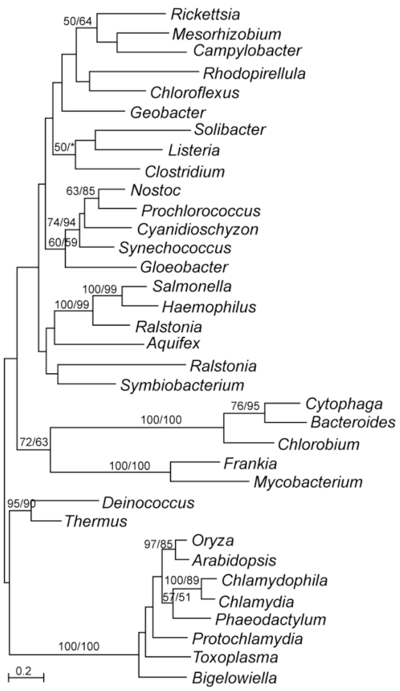

Figure 8. Phylogeny of Enoyl-ACP reductase (*fabI*). Note that sequences from green plants, diatoms, chlorarachniophytes, apicomplexans and chlamydiae form a strongly supported group whereas cyanobacterial and red algal sequences form another group. See discussions in the text.

---

9. 23S rRNA (Uracil-5-)-methyltransferase

CLUSTAL X (1.83.1) multiple sequence alignment

```
82740724_Shewanella_sp.            RRRARLATLFCQVLAKPLSDLIVPFAKLLLGHLELIAADNGLSFTPGNFV
75827716_Vibrio_choler             RRRARLSLMWCPVLEPSLNALLPDLNALLLGHVELVKGDNTLSFLPSHFI
39983735_Geobacter_sulfur          GYRCRAQFKICVICDPAINLTLRDLRLVLIPQIDAATGEDGLSFRRGGFS
2982968_Aquifex_aeolic             NYRVRVQFKICLLAHERINELIPHIREVLVHVNYSPTRDE-YRVSNDSFF
82743019_Shewanella_sp.            AFRNKAKMVVCLLYPSDMQKLLHRLTQFVLKFILLTRSQVRLFIRPKSFF
75825214_Vibrio_choler             AFRNKAKMVVCPLYPQPMQELLAYLENWILKFILLTRSENSMVIRPKSFF
71366121_Nocardioides_sp.          GVRNKAKMVVCPLHTPGIQAALPVLADFVLKYLLVTESPD-LHLRPQSFF
39983433_Geobacter_sulfur          HYRNSAKLVVCPLHHPLINRVVTAVKEGILRYLVVRISERTFTISPRSFF
18893255_Pyrococcus_furios         GHRNRIDLAVCPVFGKTSKKAIERLREYILRYMVLREGKFTYLIHPNSFF
18892856_Pyrococcus_furios         EFRNISNFIVCPVFSNRTPLYIRAMKEFLVHYLQVREGKFTYLLRPGIFF
40068573_Nanoarchaeum_equita       YYRNRMDYPICKMLSKEAEIIINEFNKFILRYLVIREGKFTYYISPNSFF
14195332_Chlamydia_muridarum       RGRNKMEFSFCLMIDERAIDILNYTRSWWLCTLTVRIGNVSFHVRPRSFF
33236756_Chlamydophila_pneumo      RGRNKMEFSFCLLIHEQTMDILKLTREWWLCTLTVRTGSPQFSLRPRSFF
46400819_Protochlamydia_amoebo     HYRNKMELTFCLLANGWFAQAARAIQKWWLRTLTLREGLRTFRISPSAFF
42565079_Arabidopsis_thalia        HYRNKMEFSFCLLQSEPGNLVLAAVQDCWLKHLMLRTGRNVFQISANSFF
34906470_Oryza_sativa              RYRNKMEFSFCLLQSEPADKVLAIVQETWLKHLMIRTGRNIFQISANSFF
21645898_Chlorobium_tepidu         HYRNKVEFSCCYLAKECMNRVLNVLRDFALRNLMLRYSERHFRISANSFF
```

```
29337947_Bacteroides_thetai          FYRNKLEFTFCWLQDDISNRIRNAVRDYALRNLIIRTSSTGFKVGPKSFY
50756251_Gallus_gallus               EYRNKCEFLITIHIPAVAKKVVKAFQDYIWKQLTVRTSRN-FRISPHAFF
54641671_Drosophila_pseudo           GYRNKNEFTVLPHLPDQAKWAAQSFQKLVFRQLMVRCSSATFRISPLAFF
45599387_Leptospira_interog          MYRHKVQLPFCRIQDEDLTTVAAAIRHWALRHIVLRKANATFQIGLSTFF
47094248_Listeria_monocy             RYRNKSQFQTCIVQQPVTIKVTNFVRDLLVRTIVVRTGVKTFDLSARAFF
65317856_Bacillus_anthra             HYRNKSQLQVCMIQHKATNEATKVVRRILVRTIVTRTAVQTFELSARAFF
47092904_Listeria_monocy             RYRNKSQVPVCLIHNEQGDFAVQKTREILIRHIMTRFAHTTFAISARSFY
65317755_Bacillus_anthra             VYRNKAQVPICLIQAEENDTLIQEVKRICLRHVMARYGQVTFAISARSFY
23125360_Nostoc_puncti               GYRNKSTYPLCPVQDARLNPLLAEVKQDIIRHLGLRIGRHTFQVRPDTFF
53763114_Synechococcus_elonga        GYRNKVSYPLCPVQDDRLNPLLAGIKADILRHLGLRIGQRTFAIGPETFF
35213705_Gloeobacter_violac          GYRNKSTFPICPVQDSRLDPLLAAARELILRHLGLRIGERTFRIEPTTFF
33634926_Prochlorococcus_marin       GYRNRALIPLCPVLDRGIDQYLASIKDDLLRHLGIRIGQKTLLLSTTTFF
46401273_Protochlamydia_amoebo       GWRCRAKLAVCLVHHPRINQAVTMIQNWMLRYLQFVVERASICFQPSNFA
42565237_Arabidopsis_thalia          GWRCRAKLAVCKSHHPNINAAIELLREGILRYVQMAVTTHSISLDPSSFG
CM4438_Cyanidioschyzon_merolae       HWRTLAKLAVCQVHHAALNKAFKCLECVLLRYVQLAVERRTMVLPPFAFQ
19712946_Fusobacterium_nuclea        NYRNKIIEPFNILNSKLGNKIIKELKEILLRNIMIRTNSNNFHISPTSFF
4981638_Thermotoga_mariti            HYRTKMEFHFCEVAPEGTGEILNTVKEAVLKHLVIRYAFSTFQIPPTAFF
45600206_Leptospira_interrog         YYRNRMDFAVCLIQSKESNEELYRFRNLILKYFTLRKAKNTFRVPFDSFF
69299484_Silicibacter_sp.            HSRRRATLTVCHLLAPELIAALPMAEDLAPLSVTITTSQAGVCPPPGAFL
41819164_Treponema_denticola         SFFEKSIFVSSLSLKENKSSGSVKIKDCPIRNLLKSNLKEYIKFNPLGFF
66797570_Deinococcus_geothe          HYRNTAQYLVPLVMAPIQAVMDRLDPERLVVAALIGAGEPRVGITATGFA
                                                                                    * 

82740724_Shewanella_sp.              QVNSQINKAMVILDLFCGMGNFSLPLAKMGADVIGVEGVAEMVSQALLLD
75827716_Vibrio_choler               QVNRAVNQHMVVLDLFCGLGNFTLPLAKQAQAVVGVEGVDEMVQHAVLLD
39983735_Geobacter_sulfur            QVNYRQNRVLVVLDLFCGNGNFSLPLASGGAQLVGVEDYGPSIDDAVILD
2982968_Aquifex_aeolic               QVNYTLWEDFLGLDLHCGVGFFTIPLSEQGNFIEGADANPSAIKDAVVVD
82743019_Shewanella_sp.              QTNPQVAAQLYLWDLFCGVGGFGLHCATKDIALTGIEIEAEAISCAIIVN
75825214_Vibrio_choler               QTNPQVAEQLYMWDLFCGVGGFALHCAAPNTAVTGIEIEPEAIASAVLVN
71366121_Nocardioides_sp.            QTNTAMAAELYVWDLYCGVGGFALAIATAHAEVTGVEVSGEAIASAVVVN
39983433_Geobacter_sulfur            QVNSGGARIIYVVDLYCGIGGIALFLAGTAREVHGIEVVEAAVNDAVVLN
18893255_Pyrococcus_furios           QTNSYQAVNLVVVDMYSGVGTFGVYLAKKGMKVVGFDSNAFAIEMAVIVD
18892856_Pyrococcus_furios           QTNSYALPLLLVLDLYSGIGTFSLYLTKKGFNVVGVEINKTAVEVALILD
40068573_Nanoarchaeum_equita         QTNSYQAVNLVVLDLYSGVGLFSLQIANEVKKVIGIEIVEEAVEMAIIVD
14195332_Chlamydia_muridarum         QPQSRQAEKIILLDLYCGAGTIGISLSPYVKKIIGVELVPDAVASAVVID
33236756_Chlamydophila_pneumo        QPQITQAAKIILLDLYCGAGTIGIMLSPYVKNVIGVEIIPDAVASAIIID
46400819_Protochlamydia_amoebo       QPNTEQAEKLYVYDLYCGTGTLGICSAKYVKEVIGIELSRESVLDAVMVD
42565079_Arabidopsis_thalia          QTNTHQAEVLYVLDLFCGTGTIGLTLARRAKHVYGYEVVPQAITDAVISD
34906470_Oryza_sativa                QTNTKQADVLYILDLFCGTGTIGLTLARRAKHVYGYEVVPEAIADAIISD
21645898_Chlorobium_tepidu           QTNTRQAETLYVYDLYCGTGTITLYLARHCKQAIGIEVVESAVKDAIVTD
29337947_Bacteroides_thetai          QTNSEQAYNLYVYDLYTGTGTIANFVSRQARQVIGIEYVPEAIEDAIITD
50756251_Gallus_gallus               QVNTQAAEVLYVLDICCGTGTIGISLAKKVKKVIGIELCQEAVQDATIVD
54641671_Drosophila_pseudo           QINTEGANVLYMLDICCGTGTIALAFAKHCKRVLGVEIVPDAIKDAAVVD
45599387_Leptospira_interog          QVNPFQIENLYVVDAYCGIGTISLYIASKSKKVIGLEENSSSIRSAVVLD
47094248_Listeria_monocy             QLNPFQTERLYLVDAYCGVGTIGQAFAGKVKEVRGMDIIPESIEDAVIVD
65317856_Bacillus_anthra             QLNPEQTVVLYIVDAYCGVGTIGLWLANDAAEVRGMDVIPEAIADAIVVD
47092904_Listeria_monocy             QVNPIQTEVLYVIDAYCGIGSISLCLAKKAKHVYGVEIVDQAIQDALVVD
65317755_Bacillus_anthra             QVNPEQTKVLYVIDAYCGIGSISLFLAQKAKKVYGVEIVPEAIEDAMVVD
23125360_Nostoc_puncti               QVYTETAEALLLVDAYCGIGTLTLPLAKKVRIATGLEVQPAAVEQAVILD
53763114_Synechococcus_elonga        QVNTEQAERLLLFDLYCGVGTLTLPLASEVGSVLGLEILPESVVQAVLLD
35213705_Gloeobacter_violac          QICTSQAEILAVVDAYCGIGTLSLPLARAARAVVGIESHVRSVEQAVVVD
33634926_Prochlorococcus_marin       QVNTLQAERIVIIDAYCGIGTISLPIAATGYHVIGLELHAEAITQALVLD
46401273_Protochlamydia_amoebo       QANLDLFEKLLVVEFFAGVGTIGLSVAAKCSWIKCEEINPHSKECFVIVD
42565237_Arabidopsis_thalia          QANTRAFDSLLVADLYAGAGVIGLSLARKCSSVKCIEVNKEARLSFVVVD
CM4438_Cyanidioschyzon_merolae       QANLTAYRAMLIAEFCAGVGSIGLSVVPAHIYLWASDIHPGGQVAFVIAD
19712946_Fusobacterium_nuclea        QINVKQAKRLYIVDAYSGTGTIGMIIAKKAKKVYAIEIVKSASEDGIIFD
4981638_Thermotoga_mariti            QSNYSITSKLIVLDLYAGIGTFSVRTSFSAARVISVESSRVAVKAGIILD
45600206_Leptospira_interrog         QPNPEGFQPILLVDLFCGSGFFSRIFAHKFLKITGIDSIESSLEIALIAD
69299484_Silicibacter_sp.            QATPEGESALVIVDLFAGAGTFSLPLAAEVHAVEGEREMMQALELGVVID
41819164_Treponema_denticola         QSNLEMTEKLIVLDFYSGVGTFSLFAYDQAKEIHLVEHNKHALAYAVFVD
66797570_Deinococcus_geothe          QVNPEAAGLAYAVDLYGGSGAIGRHLAPHFARVTVLDTAAEALVRGIVVD
                                     *            :   * * :                         . :

82740724_Shewanella_sp.              PARAGAFESLQWPRKVLYVSCNPASLARDSAVLLYRLQRLGLIDMFPQTH
75827716_Vibrio_choler               PARAGAEGIVDQAKRVVYVSCNPATLARDSQSLLFRLEKLGMLDMFPHTS
```

```
39983735_Geobacter_sulfur           PPRTGARDAVGEPERIVYVSCDPPTLGRDLGVLRYRVVGSHGVDMFPQTY
2982968_Aquifex_aeolic              PPRSGLLREERDPDKIVYISCNPTTFARDLKILTYELKRLKLIDNFPQTY
82743019_Shewanella_sp.             PPRRGIGEALCQPRAILYSSCNPKTLAKDLEHIQYHLTKVQLFDLFPHTD
75825214_Vibrio_choler              PPRRGLGSELTAPQHILYSSCNPQTMVKDIAELAYQMSRVQWFDMFPHTD
71366121_Nocardioides_sp.           PPRRGLGPELSGVRHVLYSSCNAHTLARDLDAMPLRPVRAQLLDMFPNTA
39983433_Geobacter_sulfur           PPRKGCDAGVLNPARIVYVSCAPATLARDLDILAYATLRVQPVDMFPQTP
18893255_Pyrococcus_furios          PPRAGLHPKLVKPETIVYVSCNPKTFKVNIEQLNYIIEELIALDMFPHTP
18892856_Pyrococcus_furios          PPRKGLG-EFAGPENVVYVSCNPKRFILDFKNYLYKVEDAILIDMFPHTP
40068573_Nanoarchaeum_equita        PPRAGLTNKAIEPDTIVYVSCNPYTQKRDINKLKYKIIDMQPLDMFPNTP
14195332_Chlamydia_muridarum        PPRCGMQNKALKPKKIIYVSCNPLTQISECSILVYQLQRMQPIDQFPHTH
33236756_Chlamydophila_pneumo       PPRCGMQSKVLKSPKIVYISCNPKTQFQECADLIYRIKKMQPIDQFPYST
46400819_Protochlamydia_amoebo      PPRIGLDAKAIKAPKLVYISCNPKTQVMNLGPLIYQLQIVQPVDQFPQTV
42565079_Arabidopsis_thalia         PNRPGMHMKLIKSPRIIYVSCNPATCARDLDYLCYKLMSVQPVDMFPHTP
34906470_Oryza_sativa               PNRPGMHMKLIKAPRIVYVSCNPATCARDLDYLCYELKTVIPVDMFPHTP
21645898_Chlorobium_tepidu          PPRAGMHPKALDPERIVYVSCNPDNLARDGKEIAYRMTSAQPVDMFPQTN
29337947_Bacteroides_thetai         PPRAGMHQDVVDPKRIVYVSCNPATQARDLQLLDYKVKAVQPVDMFPHTH
50756251_Gallus_gallus              PPRAGLHSRVILLKKLIYVSCNPRAAMNNFVDLCFRPVKAMAVDLFPQTK
54641671_Drosophila_pseudo          PPRAGLHNRSIAINRLVYVSCNPHSAKRNFIELAFYPKSAVAVDMFPHTT
45599387_Leptospira_interog         PPREGLDAETKSVNQILYVSCNPETLLRDAIELTFKYEKITPVDLFPHTS
47094248_Listeria_monocy            PPRSGCDQGLIKAKQLVYVSCNPSTLARDLALLAYRIRYMQPVDMFPQTA
65317856_Bacillus_anthra            PPRTGCDDKLLEPKQVVYVSCNPSSLARDVQALMYEVEYVQPVDMFPHTA
47092904_Listeria_monocy            PPRKGCDEKLLEPKKVVYVSCNPGTLARDMKILTYVAKKVQPVDMFPMTT
65317755_Bacillus_anthra            PPRKGCDEALLNPKRVVYVSCNPATLARDLKVLEYKTQEVQLVDMFPHTT
23125360_Nostoc_puncti              PPRKGCDRAVIDPSRIVYVSCKVATLARDLKLLCYTITRVQPADFFPQTA
53763114_Synechococcus_elonga       PPRKGCEPAVIEPQRIAYVSCNPATLARDLQALCYRLLLVQPLDFFPQTA
35213705_Gloeobacter_violac         PPRKGCDPEVLEPMRVVYVSCNPATLARDLKRLVYCLVGVQPVDLFAQTH
33634926_Prochlorococcus_marin      PPRKGLDQMVIKPERVAYLSCDPATLARDLKQLIYQIDELQPIDFFPQTM
46401273_Protochlamydia_amoebo      PPRKGLSRKFIEVDQLIYVSCGWDSFKKNKETLIWNLKKLEGYSLFPGSE
42565237_Arabidopsis_thalia         PPRRGLDASLRQPQRLIYISCGWESFKEDCKSLLWELEKAHGFNFFPGTD
CM4438_Cyanidioschyzon_merolae      PPRAGLGPVFISLERFIYVSCSMSSFMRDVRQLVWTLRHAQPYLFFPGAD
19712946_Fusobacterium_nuclea       PPRKGLETSIIDLKEVVYISCNPSTFARDVKLFSYVLKKLQAVDMFPQTS
4981638_Thermotoga_mariti           PPRSGAGPEVMKPERIVYVSCDPSTLVRDLKVLVYSIVRVKPFDMFPQTY
45600206_Leptospira_interrog        PPRAGLGEFVLDVSYFFYVSCNPTSQKSDLWKLKFQIQKILITDPYPQTP
69299484_Silicibacter_sp.           PPRAGAEAQIAQLPLISYVSCNPVTFARDAEVLVYKLNWVQVVDQFRWSS
41819164_Treponema_denticola        PPRIGIDKEALSTRQIFYISCDPVTFARDTASLLYKLEKHFLFDFYPQTH
66797570_Deinococcus_geothe         PPRAGLDEGARDADRLVYVSCDPATWARDVGDLTWRLGEVTPHDFYPQTS
                                    * * *          . * **       :                :  :

82740724_Shewanella_sp.             HIEAMALFEL
75827716_Vibrio_choler              HLESMALFVK
39983735_Geobacter_sulfur           HIESVTLLIR
2982968_Aquifex_aeolic              HIESIALLEV
82743019_Shewanella_sp.             HFEVLAMLVK
75825214_Vibrio_choler              HAEVLTLLVR
71366121_Nocardioides_sp.           HYEVLALLER
39983433_Geobacter_sulfur           HVENIALLVK
18893255_Pyrococcus_furios          HIELVGKLRR
18892856_Pyrococcus_furios          HVEAVIKLRK
40068573_Nanoarchaeum_equita        HIENVILMKK
14195332_Chlamydia_muridarum        HLENVVLLER
33236756_Chlamydophila_pneumo       HLENIILLER
46400819_Protochlamydia_amoebo      HVENITLLTL
42565079_Arabidopsis_thalia         HIECVCLLEL
34906470_Oryza_sativa               HIECICVLEL
21645898_Chlorobium_tepidu          HIETVACFER
29337947_Bacteroides_thetai         HVENVVLLEL
50756251_Gallus_gallus              HCELLILFER
54641671_Drosophila_pseudo          HTELVILFER
45599387_Leptospira_interog         HLESVSVFTK
47094248_Listeria_monocy            HVETVVLLQL
65317856_Bacillus_anthra            HVENVVKLTL
47092904_Listeria_monocy            HIEAVTVLHL
65317755_Bacillus_anthra            HVECVAWLKL
23125360_Nostoc_puncti              HVEAAAFLVL
53763114_Synechococcus_elonga       HVETVALLEL
```

```
35213705_Gloeobacter_violac         HVECVATLER
33634926_Prochlorococcus_marin      HIECLALLTR
46401273_Protochlamydia_amoebo      HIELLTLFER
42565237_Arabidopsis_thalia         SIEVLAVFKR
CM4438_Cyanidioschyzon_merolae      HLEMVAVFDR
19712946_Fusobacterium_nuclea       HIECVGLIER
4981638_Thermotoga_mariti           HVETAVTLVK
45600206_Leptospira_interrog        HLESVAFLKR
69299484_Silicibacter_sp.           HTELAASFSL
41819164_Treponema_denticola        HIETLGIFRK
66797570_Deinococcus_geothe         HVEVVSVLER
                                      *    :
```

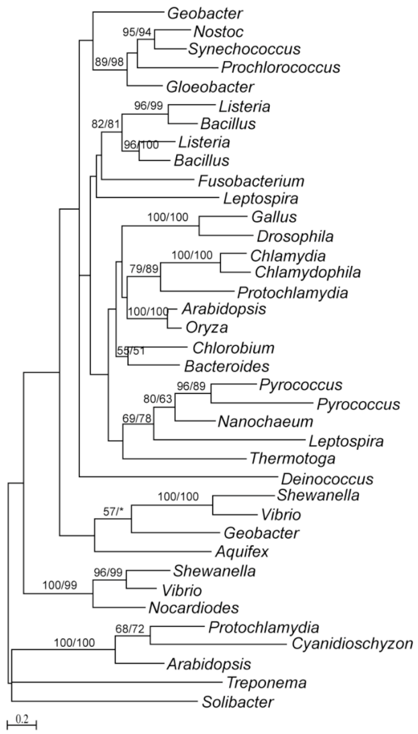

Figure 9. Phylogeny of 23S rRNA (Uracil-5-)-methyltransferase. Note that this gene contains two copies in green plants. One groups with red algal and *Protochlamydia* sequences with strong support (lower branch). BLAST search of GenBank protein nr sequence database using the red algal sequence had significant hits (Evalue < e-8) only to *Protochlamydia* and *Arabidopsis* sequences.

---

10. CMP-KDO synthetase

CLUSTAL X (1.83.1) multiple sequence alignment

```
34397752_Porphyromonas_gingiv      IPARFASSRFPGKPLADMLGKSMIQRVHERIVPRAVVATDDERIRQAVED
48853699_Cytophaga_hutchi          IPARYASTRFPGKPLIQINGKTMIQCVYDQVITEVLVATDDDRIEAEVLR
33635764_Prochlorococcus_marin     VPARLESSRLPNKVLADIGGMPMLQRVLERCPNAVVLCTDSDRLKEMAED
2983277_Aquifex_aeolic             IPARLGSTRLKEKPLKNLLGKPLIRWVVEGLGERVILATDSERVKEVVED
77545625_Pelobacter_carbin         IPARYASSRFPGKPLARILGKTMIQRVYERTIDRVVVATDDSRIADVVSG
39983882_Geobacter_sulfur          IPARFASTRFPGKALADIAGKPMVQHVYERTVSEVVVATDDDRIAQAVRG
```

```
67930978_Solibacter_usitat         IPARFSSTRFPGKVLAQIANKTMLQHVYERALTSTIIATDDERVYTAAKS
56686778_Synechococcus_elonga      IPARYASERLPGKVLLPIAGRPMLQWVYEATFDQVAIATEDPRVVEAAAA
82738953_Pseudomonas_putida        IPARFGSTRLPGKPLADIAGKPMVQHVYERAAQCVVIATDDERVANAVRD
48766205_Rhodospirillum_rubrum     IPARMASTRLPGKPLADIHGVPMIVQVWRRAIGPVLVAAAEDEIAQAVRA
77690215_Rhodopseudomonas_palu     IPARMAATRLPGKPLLDIGGVPMIVHVLRRAIGRVAVATDTPEIAEAVIA
84712599_Polaromonas_naphth        IPARLASSRLPNKPLADINGVPMVVRVAQRAALRTVVAADGTEIIEKCAA
72117710_Ralstonia_eutrop          IPARLASTRLPNKPLADIGGHPMIVRVAERAAQRTVVATDAPAVAEACAA
82736692_Pseudomonas_putida        IPARLRSTRLPGKPLLLIAGKPMVQHVWEQAASRVVIATDDTSILEACQA
68056808_Haemophilus_influe        IPARFASSRLPGKPLADIAGKPMIQHVFEKAANRVIIATDNENVADVAKN
32448211_Rhodopirellula_baltic     IPARLASSRLSQKLLLQAGGKSVLQHTYEAAAEEVIVAVDDPRLAAEVDS
32261715_Helicobacter              IPARLESTRFPKKVLCDIGGLPMVVRTALNAVDSVVVACDDEQIASVCKT
60493187_Bacteroides_fragil        IPSRFASTRLPEKPLVKIAGKEMVLRVAEIAGCNYIVATDHEKIVNFCKE
83859819_Oceanicaulis_alexan       IPARYGSTRFPGKPLHEIAGVPMVERVRRLAIDRVIVATDDARILAAVEG
15835072_Chlamydia_murida          LPARWGSSRFPGKPLAKILGKTLIQRSYENALDCVVVATDDQRIFDHVVE
29834703_Chlamydophila_caviae      LPARWGSARFTGKPLASILGKSLIRRTYENILDKVIVATDDQRIMDHVLD
46400100_Protochlamydia_amoebo     IPARYGSTRFPGKPLASILGKTLLQRTYENSLSDLIVATDDERIFEHVRS
7688437_Zea_mays                   IPARFASTRFEGKPLVPILGKPMIQRTWERVLDHVVVATDDERIAECCRG
51854266_Oryza_sativa              IPARFASSRFEGKPLAPILGKPMIQRTWERVLDHVVVATDDERIAECCRG
30695393_Arabidopsis_thalia        IPARYASSRFEGKPLVQILGKPMIQRTWERSLDHIVVATDDERIAECCRG
24197538_Leptospira_interr         IPARYASSRFPGKPLAKIGDKTMIEWTYRNALSELVVATDDVRIHEVVQK
19714354_Fusobacterium_nuclea      IPARYSSTRLEGKPLKMIEGHTMIEWVYKRALDSLIVATDDERIYNEVIN
24195250_Leptospira_interr         LPARMASSRFPDKPLVKISGLEMIEHVRRRVVDEVVVATCDEIIKQRVES
84517633_Prochlorococcus_marin     IPARLDSKRLPSKPLIKILGKTIIERTYSQAKNKIIIATDSLEIKKHCDD
67935837_Chlorobium_phaeob         IPARLDSSRLEKKMLADLQGEPLIVRTWRQAADRVVVATDNDEIASVLQA
                                   :*:*  : *:  * *    .  ::            :.     :

34397752_Porphyromonas_gingiv      FEVVMTSPECSSGTERCREAFIVLNLQGDEPFIQKEQIDLLISPKIVLDH
48853699_Cytophaga_hutchi          FKVVRTLPEHPSGTDRCFEAYFIINVQGDEPFIQPEQIRTLATPKVLVNA
33635764_Prochlorococcus_marin     WSVLMTSASCNSGSERIASVAAVINVQGDQPFLDPAVVTTMAVVKTLLAI
2983277_Aquifex_aeolic             LEVFLTPSDLPSGSDRVLYVVLIINYQGDEPFVYEEDIKLIFDVKVVLDR
77545625_Pelobacter_carbin         FEVQMTRADHATGTDRLAEVTLIVNVQGDEPLIDPHMIEAAVVVKVVTDR
39983882_Geobacter_sulfur          FRVEMTSRDHETGTDRLAEVAIIVNVQGDEPLIEPAMIDEAIVVKVVTDL
67930978_Solibacter_usitat         FRVRMTRADHLSGTDRVAEVAIIVNIQGDEPLIDPAAIDAAVVVKVVTDH
56686778_Synechococcus_elonga      FEAILTSADLASGTDRVAEASVIANVQGDQPFVTPGLLQALVTVKVVCDQ
82738953_Pseudomonas_putida        FEYVMTSPDHPSGTDRLAEVMIYINLQGDEPLVRPGDIETLATVKVVLAE
48766205_Rhodospirillum_rubrum     ANAVLTDPDLPSGSDRVWQAVVVVNVQGDLPTLDPGLIIRAVVVKAVVQT
77690215_Rhodopseudomonas_palu     HEVVMTRADHPSGSDRIHEALTVINLQGDFPTIRPEQIGAVLVVKVVGDN
84712599_Polaromonas_naphth        FQTVLTRVDHPSGSDRLAEACIVVNVQGDEPLINPALIDAVAVVKVVLDA
72117710_Ralstonia_eutrop          HDAVLTRADHPSGTDRLAEVAIVVNVQGDEPLIDPQLIDDVAVVKVVCDA
82736692_Pseudomonas_putida        FEVLMTRADHESGTDRLAEVAIVVNVQGDEPLIPPVIIDQVAAVKVVSDK
68056808_Haemophilus_influe        FEVCMTSVNHNSGTERLAEVVIIVNIQGDEPLIPPVIVRQVAAVKVLTDK
32448211_Rhodopirellula_baltic     FQARLTSVDCQSGTDRIAEVAILINVQGDEPEIDPKTIDAVACVKAVLGD
32261715_Helicobacter              HPCVMTAKSHSSGTDRCAEASIILNIQADEPFLETSVIRTLQLVKVVLNA
60493187_Bacteroides_fragil        NAVMMTSENCKSGTERCWDVTFIVNLQGDNPLCPPWFIEQLIGTTVEVDK
83859819_Oceanicaulis_alexan       FEAVMTPESCRNGTERAFEAVVIVNLQGDAPLTPPWVVDAAAGTTVVFDK
15835072_Chlamydia_murida          FLCVMTCESCANGTERVEEAVIVVNIQGDEPCLSPSIIDGLVKVKCVFDK
29834703_Chlamydophila_caviae      FDCVLTSPECANGTERTAETIIIVNIQGDEPCLQHTVVDALVKVKCVFDK
46400100_Protochlamydia_amoebo     FKVVMTSEQCPTGTDRLAEVLAIVNIQGDEPCLNPLTINLAVIVKCVMDQ
7688437_Zea_mays                   FDVIMTSASCKNGSERCCEALIVVNIQGDEPLIEPEIIDGVVRVKCVVDN
51854266_Oryza_sativa              FDVIMTSESCRNGSERCCEALIVVNIQGDEPLIEPEIIDGVVRVKCVLDN
30695393_Arabidopsis_thalia        FDVIMTSESCRNGTERCNEALVVVNIQGDEPLIEPEIIDGVVRVKCVVDN
24197538_Leptospira_interr         FRSVMTSSDHPSGTDRIIEVAIIVNIQGDEPGIEPELIDGVARVKVIIDR
19714354_Fusobacterium_nuclea      FQAIMTSKNHTNGTSRIAEVCTIINIQGDEPLIEYEMINSLINVKVVCDK
24195250_Leptospira_interr         FKAVMTSDVHRGCIDRVAEAAIVIVVQGDEPLILPAMLDDLVAPKVVVDK
84517633_Prochlorococcus_marin     IKVCLTSKNCLTGTDRVAEVAQYINLQGDEPIFPVSELNEFIIPKMVFTK
67935837_Chlorobium_phaeob         YEVVMTSPHARCGTERIAEAAIYVNLQGDEPLISPENIDLALTVKVVMDN
                                        *        .*   .      *.* *      :      .

34397752_Porphyromonas_gingiv      GGYALYFSRSVIPYLRKHIGIYAFRPTVLRKITSLPQSTAEQAESLEQLR
48853699_Cytophaga_hutchi          NGEAIYFSRQTIPYLRKHIGMYAYRPDILAQITQLKPSALELAESLEQLR
33635764_Prochlorococcus_marin     DGRALYFSRSAIPHVRGHVGMYGFRGDVLACWSGMPPSPLEALERLEQLR
2983277_Aquifex_aeolic             EGYALYFSRSPIPYFRKHVGIYGFRKETLMEFGAMPPSKLEQIEGLEQLR
77545625_Pelobacter_carbin         RGFALYFSRAPIPHPRRHIGLYVYRKDFLLTFAGLPESPLERLEKLEQLR
39983882_Geobacter_sulfur          EGYALYFSRSPLPFFRKHVGLYVYRRDFLMEFAKMPPTALELAEKLEQLR
67930978_Solibacter_usitat         AGDAIYFSRCPIPFDRKHVGLYVYQRDFLLSYSTLPVGPLERSERLEQLR
```

```
56686778_Synechococcus_elonga       RGNALYFSRSAIPYPRHHFGLYAFRRDFLAQYRQLPPTPLERCESLEQLR
82738953_Pseudomonas_putida         NGNALYFSRSPIPYPRKHVGVYAYRRAVLSEYSRLPQPMIEHTEKLEQLR
48766205_Rhodospirillum_rubrum      RGRALYFSRATVPHGPHHIGLYAYRRTTLGAFVSLPPGVLERREKLEQLR
77690215_Rhodopseudomonas_palu      LLRALYFTRATAPYGDHHIGLYAYRRAALERFVSLPPSPLEQREKLEQLR
84712599_Polaromonas_naphth         RQTALYFSRAPIPAARRHVGIYAYRVGFLRQFPQLPQAPLEQLESLEQLR
72117710_Ralstonia_eutrop           GGRALYFSRAPIPWSRRHIGLYAYRAGFLRRFPTMAAAPLEQTEALEQLR
82736692_Pseudomonas_putida         NGLALTFSRAPLPWARRHIGMYAYRVGFLHDFVSWGPCWLEQAEALEQLR
68056808_Haemophilus_influe         DGYVLYFSRSVIPYDRRHIGIYAYRAGFIKQYVQWAPTQLENLEKLEQLR
32448211_Rhodopirellula_baltic      DHRAITFSRAAVPHPRQHIGLYAYRREFLLWFATQPPGRLEQIEKLEQLR
32261715_Helicobacter               HNEAIYFSRSPIPFCRGHLGLYGFSAKSLQEFCSLPKSPLEEIEKLEQLR
60493187_Bacteroides_fragil         FGYALAFSKAMIPVIRRHIGLYSYTYDALKKYFEVEASPYELPEGLEQMR
83859819_Oceanicaulis_alexan        AMNALYFSKAIIPFKRKHIGLYAYRFDTLSELVKLDPSPLELTESLEQLR
15835072_Chlamydia_murida           TGRALYFSRSVIPNNFLHIGVYAFRRAFLSEYVKIPPSSLSLAEDLEQLR
29834703_Chlamydophila_caviae       NGKALYFSRSPIPHILLHIGVYAFRRNALFNYIESSPTPLSQAEDLEQLR
46400100_Protochlamydia_amoebo      QGNALYFSRALIPSNKRHLGLYVYRPSFIINYQKLPSTPLQLEEDLEQLK
7688437_Zea_mays                    LGYAIYFSRGLIPFNKLHLGIAGFDSKFLKIYPELPPTPLQMEEDLEQLK
51854266_Oryza_sativa               QGYAIYFSRGLIPFNKLHLGIAGFDSKFLKIYPELPPTPLQLEEDLEQLK
30695393_Arabidopsis_thalia         RGYAIYFSRGLIPYNKLHLGIQSFDSKFLKVYSELQPTPLQQEEDLEQLK
24197538_Leptospira_interr          NGKAIYFSRSLIPSQFRHLGIYGYDRDFLLQYNSLPKSNLEESESLEQLR
19714354_Fusobacterium_nuclea       NDYAIYFSRSVIPYPRKHIGIYGYKRDFVIEYSKMLATPLEEIESLEQLR
24195250_Leptospira_interr          NWDLLYASREPIPSRKKQLGVIAFRNDFLQTFAALAPTPLEIIESVDMNR
84517633_Prochlorococcus_marin      SKNLLYSSRAPIPSNKKHICIYAFNRLHLEAFSQIKKTTFEDEEDLEINR
67935837_Chlorobium_phaeob          AGYALYFSRSPIPYQRRHIGLYAFRADVLHAFASLAPSMLELAESLEQLR
                                        :  ::   *    :. :  :    :           .  * ::  :

34397752_Porphyromonas_gingiv       WLEYGYRIRVLQTQQSTIGIDTPEDMEK
48853699_Cytophaga_hutchi           WLESGYSIHTAVTTIETVGIDTPEDLLR
33635764_Prochlorococcus_marin      LIEAGHTISTFAVEGTSLSVDTVEQLEE
2983277_Aquifex_aeolic              LLENGIKIKVLITENYYHGVDTEEDLKI
77545625_Pelobacter_carbin          ALENGYAIRVVETDRVSLGVDTPEDLVR
39983882_Geobacter_sulfur           ALENGCRIRVVETAHESIGVDTPNDLEK
67930978_Solibacter_usitat          ALENGYRIRVVETEYESLGVDTPEDLER
56686778_Synechococcus_elonga       VLEQGYRIRVVPCADKVIEVNTADDLER
82738953_Pseudomonas_putida         LLAAGYSIRAYRVEPTGPGVDTPECLEK
48766205_Rhodospirillum_rubrum      ALENHMRIEAALVDTVPLGVDTAEDLER
77690215_Rhodopseudomonas_palu      ALEAGMRIDVGIVDSVPRGVDTPADLET
84712599_Polaromonas_naphth         ALWHGYRIAVHITEHAPPGVDTPEDLER
72117710_Ralstonia_eutrop           AMWHGERIAVLETAAAPPGVDTAADLER
82736692_Pseudomonas_putida         ALWHGVRIHIEDAIEAPVGVDTPEDLER
68056808_Haemophilus_influe         VLYNGERIHVELAKEVPVGVDTAEDLEK
32448211_Rhodopirellula_baltic      AIEAGKTIVVAPVEASAPGIDTLEDFRA
32261715_Helicobacter               ALYQGKTIAMAVVQTQSVGIDTPQDYQR
60493187_Bacteroides_fragil         FLHNRIPVKMIDVDYRNSGVDSPEDIER
83859819_Oceanicaulis_alexan        ALENGIPIRVVLTEYRGYSVDSPRDAEV
15835072_Chlamydia_murida           VLESGRSIYVHVVQNATPSVDYPEDISK
29834703_Chlamydophila_caviae       ILEHGGSIHVCVVEAKSPSVDYPEDINK
46400100_Protochlamydia_amoebo      VLEHGYRIKVAIVDQANIGVDTPEDIHK
7688437_Zea_mays                    VLENGYRMKVIKVDHDAHGVDAPEDVEK
51854266_Oryza_sativa               VLENGYRMKVIKVDHDAHGVDAPEDVEK
30695393_Arabidopsis_thalia         VLENGYKMKVIKVDHEAHGVDTPDDVEK
24197538_Leptospira_interr          AIEAGYGIGIYLSKEAGLSVDTPADLEI
19714354_Fusobacterium_nuclea       VLENGYKIKVLETTHSLIGVDTQENLEQ
24195250_Leptospira_interr          AVEHGYKVRMVLTEGIMIGVDVPGDVSR
84517633_Prochlorococcus_marin      FLELDIRVKCIELKNSGKAVDNESDLID
67935837_Chlorobium_phaeob          LLENGYRIRCVKTTRDAPGVNTYEDLEL
                                     :     :           ::
```

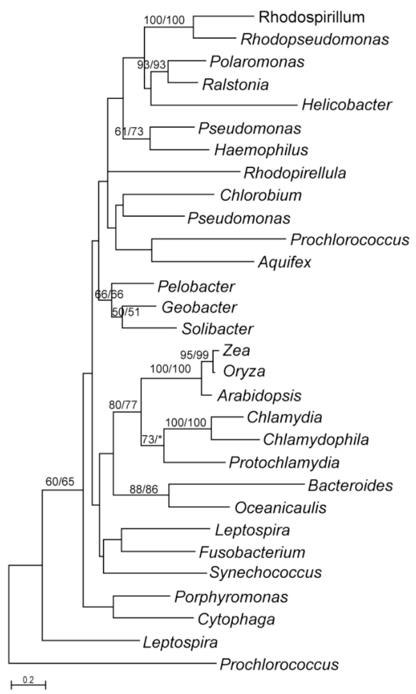

Figure 10. Phylogeny of CMP-KDO synthetase, showing sequences of green plants and chlamydiae form a monophyletic group.

---

11. ATP/ADP translocase

See the following for phylogeny:

1. Wolf, Y. I., Aravind, L. & Koonin, E. V. Rickettsiae and Chlamydiae: evidence of horizontal gene transfer and gene exchange. Trends Genet 15, 173-5 (1999).
2. Linka, N. et al. Phylogenetic relationships of non-mitochondrial nucleotide transport proteins in bacteria and eukaryotes. Gene 306, 27-35 (2003).
3. Greub, G. & Raoult, D. History of the ADP/ATP-translocase-encoding gene, a parasitism gene transferred from a Chlamydiales ancestor to plants 1 billion years ago. Appl Environ Microbiol 69, 5530-5 (2003).
4. Amiri, H., Karlberg, O. & Andersson, S. G. Deep origin of plastid/parasite ATP/ADP translocases. J Mol Evol 56, 137-50 (2003).
5. Schmitz-Esser, S. et al. ATP/ADP translocases: a common feature of obligate intracellular amoebal symbionts related to Chlamydiae and Rickettsiae. J Bacteriol 186, 683-91 (2004).

---

12. 12. Glycerol-3-phosphate acyltransferase. **Note this gene is only present in chlamydiae and plastid-containing eukaryotes.**

CLUSTAL X (1.83.1) multiple sequence alignment

```
15834812_Chlamydia_murida           PEPLYQKFQICHQTYIEAATRKIIFPPYHKKIRTPYQFGIDFFSVLIDDQ
33236837_Chlamydophila_pneumo       PEPLYQKFSVFHQNYIDAATKKFIFPPYHKKIRAPFRLSIDFFSLVIDDK
46400593_Protochlamydia_amoebo      KPPFAAIIEHFHDSYLLATKHSYQFEIFHQSIRSPYQFGLDFIRPLINFE
CM1432_Cyanidioschyzon_merolae      STRACSVLRGWYESYEKAALAAFHFKPYHQAVSGPTAFGNRFSRPLCDLQ
7576943_Elaeis_guinee               SSDIANGLEELYYNYRNAVLQSFTFSPHHQAIREPYMFGQNYIRPLIDFR
37537152_Oryza_sativa               PADVAANLEELYYNYKDAVMQSFTFPPYHKAVREPYMFGQNYIRPLVDYR
30692655_Arabidopsis_thalia         PANVAAGMEELYWNYKNAVLSSYTFNPYHKAVREPYMFVHTYIRPLIDFK
154139_Chlamydomonas_reih           PPQLEPAWDYFYDNYKKAVTSSYTFPSFHTRLIEPYDFGQRYVATLIDFQ
23619128_Plasmodium_falcip          ITDHINTFKGFLRKYLYEIKKHYEFPNIHRYDQTLYEWSLKFWSELIDKK
                                                    .*          *    *               :   : : .

15834812_Chlamydia_murida           KSQVLHSNRLDQIQEYICSGHNVVLLANHQTESDPQLMYCLLASHPQLME
33236837_Chlamydophila_pneumo       NSRILNLHRLKEIEEYIARGDNVVLLANHQTECDPQLMYYALKTHPELME
46400593_Protochlamydia_amoebo      KSTVLGREQLAKIYQQMAQGDNVILLANHQTEPDPQILSLMLNIDKKLAT
CM1432_Cyanidioschyzon_merolae      RSYLDPSSSFACIRAQLAAGDNVCLVGNHQSEADPFAIYHMFESGAALAE
7576943_Elaeis_guinee               RSYIGNISIFSDMEEKLQQGHNIVLMSNHQTEADPAIIALLLRTNSHIAE
37537152_Oryza_sativa               NSYVGNISIFQDMEQKLQQGHNVVLMSNHQTEADPAIIALLLRSNPWISE
30692655_Arabidopsis_thalia         NSYVGNASIFSELEDKIRQGHNIVLISNHQSEADPAVISLLLAQSPFIGE
154139_Chlamydomonas_reih           NSVLGFRERFDRVQELLDQKHNVVILANHQTEADPGVFAHMLKTHPKLAT
23619128_Plasmodium_falcip          NSKFLGISNIKKIEEWRDKGHNIIIFSNHHIEADANIIKYFFQDSEQISR
                                    .* .      :  :       .*: :..**: * *.  :   :     :

15834812_Chlamydia_murida           NMIFVAGDRVTSDPLARPFSMGCDLLCIYSKRHINHPPELKEEKLNHNQK
33236837_Chlamydophila_pneumo       NMIFVAGDRVTSDPLARPFSMGCDLLCIYSKRHIATPPELREEKLLHNQK
46400593_Protochlamydia_amoebo      EMIFIAGHRVISDPLAIPMSMGRNLLCIYSKKHISHPPEKKTQKVSHNQR
CM1432_Cyanidioschyzon_merolae      SILYMAGDRVRDDPLAAPFSVGRSMLTVYSKKHILDEPDRRSEKQAHNRK
7576943_Elaeis_guinee               TMVFVAGDRVLTDPLCKPFSMGRNLLCVYSKKHMDDVPELIEMKRRANTR
37537152_Oryza_sativa               NIVYVAGDRVVTDPLCKPFSMGRNLICVYSKKHMNDFPELVDMKRRANTR
30692655_Arabidopsis_thalia         NIKCVAGDRVITDPLCKPFSMGRNLICVYSKKHMNDDPELVDMKRKANTR
154139_Chlamydomonas_reih           DVIYVAGDRVVTDPMCKPFSMGRNLFCVHSKKHMDDAPELKAAKMETNRK
23619128_Plasmodium_falcip          NIIFIGGHKIRVDPLSRPFSVTANLLCIYSKKYIENPPHLKEEKTLFNHK
                                     :  :.*.::  **:. *:*:  .:: ::**:::   *.    *   * :

15834812_Chlamydia_murida           SMRTLKMLLSEGSKFIYVAPAGGRDRKNSQELYPAEFHPDSIEMFRLLAK
33236837_Chlamydophila_pneumo       SMQILKTLLNEGGKFIYVAPAGGRDRKNAERLYPSEFSPESIEVFRLLAK
46400593_Protochlamydia_amoebo      TMRKMSELLSEGGRCIYVAPSGGRDRRNSEQITIAPFDPQSLEMFWLMAQ
CM1432_Cyanidioschyzon_merolae      TLREMERLFADGGRCVWFAPSGGRDRRSPERVTCASFDPDAIELFRLSAE
7576943_Elaeis_guinee               SLKEMALLLRGGSQIIWIAPSGGRDRPDPSEWHPAPFDVSSVDNMRRLVE
37537152_Oryza_sativa               SLKEMALLLRGGSQIIWIAPSGGRDRPDPLEWHPAPFDASAVDNMRRLLE
30692655_Arabidopsis_thalia         SLKEMATMLRSGGQLIWIAPSGGRDRPNPSEWFPAPFDASSVDNMRRLVE
154139_Chlamydomonas_reih           TLVAMQRKLNEGGTLMWIAPSGGRDRPNANEWVPDNFDPAAVELMRNLVQ
23619128_Plasmodium_falcip          SLNVLRNLLNKGKQIIWLAPSGGRDRKGADKIHISPFDPKIIQTFNVFAK
                                    ::  :   :  *   ::.**:***** .. .     *    :: :    :

15834812_Chlamydia_murida           SSGKPTHFFPFAMKTYDILPPPPTIEETIGEHRVISFAPIAFNFGEELRA
33236837_Chlamydophila_pneumo       ASNQTTHFYPFALKTYDILPPPPKIENAIGEQRAIFFAPVFFNFGAELRA
46400593_Protochlamydia_amoebo      QADHPTHFYPLSLHTYDLMPPPKHVEKELGEKRTAQFTPVHLAFSSEIRA
CM1432_Cyanidioschyzon_merolae      KAGRPCHYYPMAIGTFNMLPPPDQVEKEKGEQRVVSYARLWMSLMPQVRA
7576943_Elaeis_guinee               HSSVPGHIYPLSLLCYEVMPPPQQVEKQIGERRTISFHGVGLSVAPELFS
37537152_Oryza_sativa               HSGVPGHIYPLSLLCYEVMPPPQKVEKEIGEQRVISFHGVGLSVTEEIFS
30692655_Arabidopsis_thalia         HSGAPGHIYPMSLLCYDIMPPPPQVEKEIGEKRLVGFHGTGLSIAPEIYS
154139_Chlamydomonas_reih           RAKQPGHLMPMSMFSYPMMPPPKTVDKSIGERRLTAFTGVGISLCEELLA
23619128_Plasmodium_falcip          RSKVKTHFIGLALNTYNICPPPNTIDVEIEKERTCNYSPVYLNLGQDIIT
                                     :    *   :::  : : ***  ::    :.*   :    : .  :: :

15834812_Chlamydia_murida           SRVFSIVTDLYKEI
33236837_Chlamydophila_pneumo       EKVFSIVKNLYEEL
46400593_Protochlamydia_amoebo      EFIWKIVRRNYELF
CM1432_Cyanidioschyzon_merolae      ENLYHLVVQKYTQM
7576943_Elaeis_guinee               QALYNSVGEQYNVL
37537152_Oryza_sativa               ESLYNSVVDQYNAL
30692655_Arabidopsis_thalia         QALYKSVNEQYEIL
154139_Chlamydomonas_reih           KAAHDAVKESYAVL
23619128_Plasmodium_falcip          TYLYNYVNQLYKQI
                                           *    *  :
```

13. Isoamylase

CLUSTAL X (1.83.1) multiple sequence alignment

```
19714344_Fusobacterium_nuclea       YNFNHYINLGANGCSFAIYAKNVNSLSLNIFHILSKLGDIWSIFLENIKE
32398065_Rhodopirellula_baltic      KAEGSPFPLGASSFNFSLYSRHATAVHLLLYVELSKSGPVWHCRVRSEHP
42525620_Treponema_dentic           FFQGKASPLGAKGVNFSVFSRNAKEIVLHLFYKLDKTGDVWHVFVSGLKS
29144251_Salmonella_enteri          LAIGEATPHGATGVNFTLFSAHAERVELCVFYDLPRRGDVWHGYLAGARP
48765636_Rhodospirillum_rubrum      LDAGEPWPLGAMGVNIAVFSEAATQIDLCLFLPLPRTGDIWHGFLPGGGP
1707700_Sulfolobus_solfat           LRPGDPYPLGSNGVNFSLFSENAEKVELLLYIEVKKTGDIWHVFVPGLRP
39936707_Rhodopseudomonas_palu      LTAGTDSRLGATGTNFALFSANAHKVELCLFIELPRTEDVWHGYLNDVSP
6457937_Deinococcus_radiod          LRPGSPFPLGATGTNFALYSENATGVELCLFFPLTQTAFVWHGYLPGIQP
48894700_Trichodesmium_erythr       LWPGKPYPLGSSGTNFAIFSENATSVELCLFLTLTVSNFIWHGYLPGISP
57116886_Mycobacterium_tuberc       VWPGNAYPLGATGTNFSLFSEIAEKVELCLIIPLDVDGYVWHAYLPNITP
8246833_Streptomyces_coelic         VWSGHPYPLGAAGTNFALFSEVAERVDLVLVVPLPVDGFVWHCYLPGVGP
73538060_Ralstonia_eutropha         LLPGKPYPLGAQGVNFAVFSANASRIDLCLFMPLPCTDEIWHGYLPNAAL
48784580_Burkholderia_fungor        LLPGSPYPLGASGVNFAVFSANAQKIELCLFFTLPCTDEVWHGYLPNAHP
48763460_Rhodospirillum_rubrum      VWPGQPFPLGATGTNFALFSAHAEKVELCLFIVLPYTDEIWHGYLPDIRP
29141918_Salmonella_enteri          IRSGYSHTLGANGVNFAIFSAHAERVELCLYLELPYTDEIWHGYVTKLQP
39936374_Rhodopseudomonas_palu      VREGLPYPLGSCGTNFALFSANATKVEVCLFIELPYTDEVFHGYIPDIGP
71737201_Pseudomonas_syringae       IREGLPFPLGASGVNFALFSANATKVELCLFIELPYTDETYHGYLPDAHP
48785916_Burkholderia_fungor        IAEGTPFPLGATGVNFALFSAHATKVELCLFIELPYTDEVWHVFVPNLKP
29840170_Chlamydophila_caviae       FYPGSPLPLGATRYRFALFSSQATQVVLALSIVLSRTGAVWHIEVEGISD
15604761_Chlamydia_tracho           VRSTIPLPLGAKRYRFSLFSSQAQQVTLVLLIPLSRTGAIWHIEIAGISS
53756174_Methylococcus_capsul       YSTGSPLPLGVHDANFALFSRHGSRVRLLLFIDLDRTGDIWHVAVHGAHR
87311865_Blastopirellula_marin      LQFTHILPYGAVGVQFVVFSRNATAMRLLMYIDFDRWGDIWSIFVPGVSA
32446352_Rhodopirellula_baltic      LQFAYSPPFGATGVQFSVFSRSATEMRLLLYIDFDRWGDVWSLHVPGLEA
48837599_Methanosarcina_barker      IERGYPHPLGATGVNFSIYSEHADYVELLLFVKVSKTFHFWHVYVRGLKP
48893487_Trichodesmium_erythr       ILPGNTFPIGATGVNFSIYSKNATAIELLLFILLDRTHHYWHIFISGIGA
16329290_Synechocystis_sp.          VHLGQTFPLGATGVNFCLFSKHAERVTLLLFIELHRTFYYWHVFVKGLKA
CM4050_Cyanidioschyzon_merolae      VRRGHPLPYGVSGTNFAIFTDEEEHVALILYIELDRTGHVWHVQIAPNVE
34098817_Arabidopsis_thalia         ISDGLPSPFGPTGVNFSVYSTNSVSATICLIIQLDRTGHVWHVFLRGDFK
46360122_Ostreococcus_tauri         PRRGDASALGATTVNFAVYTSAATAVSLVLWIELDKTGSVWHVALPRCAE
22136708_Arabidopsis_thalia         VSSGEVSPLGVSGINFALFSQNATSVTLCLSLVLDKTGDTWHICVEDLPL
46446740_Protochlamydia_amoebo      ITPGSPFPFGANKVNFALYAKNIEKISLCLFIELEKTGNVWHIAIESLPP
82748503_Clostridium_beijer         IRPGFYLENGAVGVNFTIHSQSATSCKLLLFIPFPRIGNVYSMIVLGLDI
48894542_Trichodesmium_erythr       LRSGKPFPFGATGVNFSIFSSYATSCTLVLFIPFPRIGNVYCMIVFDLDV
16330244_Synechocystis_sp.          LRCGQPFPFGATGVNFSIYSSHSTACTLVLFIPFPRIGNVYCMVVFDLDF
                                             *     : :.:       : :   .      :   :

19714344_Fusobacterium_nuclea       GYNWEING-----------MAILDPYALAYTGLARVGTPKKDMIIYESHI
32398065_Rhodopirellula_baltic      DYAYRVDGP-APQGDFDFQKILLDPFARGVFFAPLGIL-GSDLVIYEMHV
42525620_Treponema_dentic           WYLYTADGEFSPSALFDENNYLLDPYARLISSFPKCVVPLQKCVIYEAHV
29144251_Salmonella_enteri          GYGYRVHGPWQPAQRFNPAKLLLDPYARQVEGAPKSVVPWGKTVIYEAHV
48765636_Rhodospirillum_rubrum      GYGLRAHGPYDPGRRFNPHKLLIDPAARRLDGVPKAVVP-GRLLIYEAHV
1707700_Sulfolobus_solfat           GYAYRVYGPYKPELRFNPNKVLIDPYAKAINGVPKSVVPLKDTVIYEVHV
39936707_Rhodopseudomonas_palu      GYGYRVHGPYDPDRRFNAHKLLLDPYAKRLNGMPKVVVRWEDTIVYEAHV
6457937_Deinococcus_radiod          GYGYRVHGEYAPEKRFNPNVVLLDPYAKALDGAPLGLVPFHQSVIYEAHV
48894700_Trichodesmium_erythr       GYGFRVHGPYEPSQRFNPNKLLIDPYAKAIDGMPKSVVPNHESVIYEINL
57116886_Mycobacterium_tuberc       GYGFRVHGPFDPAARCDPSKLLLDPYGKSFHGTMTSVVPYHETVIYEAHV
8246833_Streptomyces_coelic         GYGYRVHGPWAPAVRCNPAKLLLDPYTRAVDGTMLGVVPYSESVIYEAHV
73538060_Ralstonia_eutropha         GYGFRAFGPYDPARRFNPHKLLLDPYARRLSGMPKAVVPWQDTVIYEAHL
48784580_Burkholderia_fungor        GYGFRAHGPYQPQHRFNPHKLLLDPYARKLVGMPKCVVPWGETIVYETHV
48763460_Rhodospirillum_rubrum      GYGYRVYGPYDPNARFNANKLLIDPYAKSLKGMPKCEVPWHETVIYEMHP
29141918_Salmonella_enteri          GYGYRVYGPYDPENRFNPNKLLIDPYARELVGTPKCRVPWSNAIIYETHV
39936374_Rhodopseudomonas_palu      GYGYRVYGPYEPENRFNPNKLLLDPYARAHAGVPKCVVPWDQAIIYETHV
71737201_Pseudomonas_syringae       GYGYRVYGPYDPKNRFNHNKLLIDPYAKQLVGVPKSKVPWDKTIFYETHV
48785916_Burkholderia_fungor        GYGYRVHGPYEPEKRFNPNKLLLDPYAKAHIGVPKCKVPWERVIFYEAHV
29840170_Chlamydophila_caviae       QYAFRVDGPTNTTAKFDFKKYLADPYAKNLHSYLKNE-PKEESIIYEMHV
15604761_Chlamydia_tracho           EYAYKLRGTDLSSQKFATDSYIADPYSKNIYSYLKHE-PKENYFIYEMHV
53756174_Methylococcus_capsul       GYAFQVDGPHEPHLRFDPQAVLLDPYATALVTVAKALVHWSELVIYEAHV
```

```
87311865_Blastopirellula_marin      GYHFQAEGPYDPSRLFDGRARLIDPYAKALAGPPKCVVDLSESIIYEMHV
32446352_Rhodopirellula_baltic      GYHFQASGPWEPENRFDSTARLIDPYAQALAGPPKCVVDVSESIIYEMHV
48837599_Methanosarcina_barker      GYAYRIGGPFDPSRRFDGDKVLIDPYSKG-NNSMRSVVELNETIIYELHV
48893487_Trichodesmium_erythr       GYAYRVYGPFNPEKRFDGTKVLLDPYARIVVGALKGVVPYAKTVIYEMHV
16329290_Synechocystis_sp.          GYAYRVDGPHEPEKRFDPDKVLLDPYAKAIVGALRSVVPYAASIIYELHV
CM4050_Cyanidioschyzon_merolae      HYAWRI-GDRPTRW----SNIVLDPYAKCVFSRPICGVPFKDLIIYELHV
34098817_Arabidopsis_thalia         DYGYRFDGKFSPEEYYDSSNILLDPYAKAIISQMACMVPQKDLVIYEMHV
46360122_Ostreococcus_tauri         DYGYRVDGPYEPEARFDKSKILLDPYAKFTVSVPKKLRPMRDLVVYEAHA
22136708_Arabidopsis_thalia         NYGYRVDGPGEWQQRFDRSILLLDPYAKLVKGFYGTYDPEKDLVIYEMNV
46446740_Protochlamydia_amoebo      YYAFRVP-------VENSNYLLIDPYAKSIYSSPLGRIPSKDLIIYEMHI
82748503_Clostridium_beijer         EYAYSVDGPYIPERVFNSSKYLLDPYSKAIAGQYKSRVPMKDLIIYELHV
48894542_Trichodesmium_erythr       EYGYRMDGPNNFHEWFDKSKILMDPYAKIIGGHRGRIPPPQDQIIYEMHV
16330244_Synechocystis_sp.          EYGYRMEGPNNFQQWFDPSKVLLDPYAKVVSGHRGRLSPLEDMVIYEMHV
                                     *                   : **                  ..** :

19714344_Fusobacterium_nuclea       GLFTKSSSNTGATYSAFEEKIPYLKNLGINVVEFLPIFEWDKNVWGYNPI
32398065_Rhodopirellula_baltic      RGFTKPNSGVQGTFLGVVDKIPYLVELGVTAVELMPVFQFDGDYWGYMPL
42525620_Treponema_dentic           KGFSFLNDKIRGKYSGLVELIPYLKDLGITSLELLPVFDFDKNYWGYSTI
29144251_Salmonella_enteri          KGLTYLHPELRGTYKALGHPVAYFKQLGITALELLPVAQFATNYWGYNPM
48765636_Rhodospirillum_rubrum      KGLTRTHPQVRGTFDGVAGSIAHLKHMGVTALELLPVWAFATNYWGYNPV
1707700_Sulfolobus_solfat           KGFTKLRLDLRGTYEGLASEISYLKDLGITTVELMPVFHFITNYWGYDPI
39936707_Rhodopseudomonas_palu      KGLTQLRDDVRGTFGGLACPIAHLKRLGVTTIELLPVQGFVVNYWGYNTI
6457937_Deinococcus_radiod          KGLTMTHPDVRGTYAGVATPLDYLRDLGITAIEFLPVHQHVTNYWGYSTL
48894700_Trichodesmium_erythr       KGFTKLHPEIRGTYAGLAHPIAYLQSLGITAVELMPIHHFLSNYWGYDSI
57116886_Mycobacterium_tuberc       KGMTQTHPSIRGTYAGLAHPIDHLNELNVTAVELMPVHQFLRNYWGYNTF
8246833_Streptomyces_coelic         RGLSRTHPDVRGTYAGLAHPVDHLTSLGVTAVELMPVHQFVSNYWGYNTI
73538060_Ralstonia_eutropha         RGVSMLRDDVRGTFAALCDPLDHLLRMGITAVELLPIHAILRNYWGYNTL
48784580_Burkholderia_fungor        RGASMLRADLRGTFAALASPIDHLLKLGVTAVELLPVHAFLRNYWGYNSA
48763460_Rhodospirillum_rubrum      RGFTMLHPGVRGTFDGLAHRTDYLTDLGVTAIELLPVHAYVTNYWGYNSI
29141918_Salmonella_enteri          KGFTQLNSAIRGTFEGMGHKVDYIKSLGITSVELLPVHWFPKNFWGYNSL
39936374_Rhodopseudomonas_palu      KGFTKLHPDVRGTYAGFGTKVDYISSLGVTAVELLPIHTFVVNYWGYNTI
71737201_Pseudomonas_syringae       RGITMRHPEVRGTFAGLGSAVDHIRKLGVTSVELLPIHAFVTNYWGYNSI
48785916_Burkholderia_fungor        RGFTKRHPEVRGTFAGLGQQLDYIKNLGVTSVELMPIQTFVTNYWGYNTI
29840170_Chlamydophila_caviae       RSFTWNSSKVPGTFLGIIEKIDYLKKLGINAIELLPIFEFDCNYWGYASV
15604761_Chlamydia_tracho           RSFTRPSSQVPGTFLGIIEKIDHLKQLGVHAVELLPIFEFDCNYWGYSSV
53756174_Methylococcus_capsul       RGLSIPSSAVPGTYLGVIDKIPYFKRLGITALELMPLQAFNRNYWGYNTI
87311865_Blastopirellula_marin      KGFTAETSGVPGTYAGVVEKIPYLKSLGVTAVELMPVHEFPNNYWGYDSM
32446352_Rhodopirellula_baltic      RGFTKKTAKVPGSYLGVIEKIPYLKSLGVTSVELMPVHEFPPNYWGYDPM
48837599_Methanosarcina_barker      GGFTRPTSGASGTFSGVIDKIPYLKELGITAVELMPVFDFDKQYWGYDPI
48893487_Trichodesmium_erythr       GGFTRPNSGVRGTFAGLIEKIPYLKELGITAVELLPVQQFDINYWGYSQI
16329290_Synechocystis_sp.          GGFTRPNSGLRGTYAGLIEKIPYLKELGITAVELLPVHYFDTNYWGYSTI
CM4050_Cyanidioschyzon_merolae      RGLTKTTSP-AGTYLGVIEKIPYLKQLGINAVELMPCAEFNCQFWGYSPI
34098817_Arabidopsis_thalia         RGFTRESSKIPGTYQGVAEKLDHLKELGINCIELMPCHEFNVNFWGYSTI
46360122_Ostreococcus_tauri         RGLTALETKAPGTYAAIEEALPYLKQLGVNAIELMPCHEFNRNFWGYSTV
22136708_Arabidopsis_thalia         RAFTAESSGMGGSYLGFIEKIPHLQDLGINAVELLPVFEFDVNTWGYSTV
46446740_Protochlamydia_amoebo      RGLTQQSSQVPGTYLGVIEKIPYLKELGINAVELMPIYEFNVNYFGYSTV
82748503_Clostridium_beijer         RGFTKPSSKVPGTFAGLAEKIPYLKELGINAVELMPIFEFDCDYWGYNPV
48894542_Trichodesmium_erythr       RSFTAPSSGVPGTFAAIREKIPYLKELGVNAVELMPIYEFDYNYWGYSTV
16330244_Synechocystis_sp.          RGFTKPSSGVRGTFAGILSKIPYLQELGVNTIELMPIFEFDVNYWGYSTV
                                       :       ..: ..     ::  :.:  :*::*      : :**

19714344_Fusobacterium_nuclea       NFFALTKKYINEFKKLIFSLHKNGIEVILDVVYNHTAEGGTGGYNFKAMG
32398065_Rhodopirellula_baltic      NFFAPHHAYRDEFCTMVKALHAAGIEVILDVVYNHTCEGGHRGYCWKGID
42525620_Treponema_dentic           AFFAPKALYVNEFKFMVREFHKAGIEIILDVVFNHTAEGNENGFSFKGFD
29144251_Salmonella_enteri          AMFALHPAWLDEFRDAVKALHRAGIEVILDIVLNHSAELDLDGFSLRGID
48765636_Rhodospirillum_rubrum      CLTAPHAAYPDSFRRMIDCLHGADIAVILDVVLNHSGEGDEYGLSFRGLD
1707700_Sulfolobus_solfat           NFFSPECRYVLSFKKMVNELHNAGIEVIIDVVYNHTAEGNHLGLSFRGID
39936707_Rhodopseudomonas_palu      SFFAPEARYLDSFRTTVARLHDAGIEVLLDVVYNHTAEGNHLGLSFRGID
6457937_Deinococcus_radiod          NFFAPDVRYVPEFKNMVRALHDAGIEVILDVVYNHTAEGNHMGMSFKGID
48894700_Trichodesmium_erythr       NYFAPYSGYVREFKQMVKALHNGGIEVILDVVYNHTGEGNHLGLSFKGID
57116886_Mycobacterium_tuberc       GFFAPHHQYVAEFKTMVRSLHEAGIEVILDVVYNHTAEGNHLGINFRGID
8246833_Streptomyces_coelic         GFFAPHNGYVSEFKSMVKTLHEAGLEVILDVVYNHTAEGNERGLSFRGID
73538060_Ralstonia_eutropha         SYFAPEPSYLHELKVAVRRLHAAGIEVILDVVYNHTCEGNELGVSWRGLD
48784580_Burkholderia_fungor        AFFAPEPSYLDEMRIAVRQLHAAGIEVILDVVYNHTCEGNEMGVSWRGLD
48763460_Rhodospirillum_rubrum      GFLAPHATY-SAFKAFVQKMHDAGLEVILDVVYNHTAEGNHLGLSFKGID
```

```
29141918_Salmonella_enteri          GFFAPATRYIQGFRDMVRAFHDAGVEVILDVVYNHTAEGNELGLSFKGID
39936374_Rhodopseudomonas_palu      GFFAPDPRYLREFKEMVARLHDVGLAVILDVVYNHTAEGNELGLSFKGID
71737201_Pseudomonas_syringae       AFFAPDPRYIAEFKEMVAHLHHAGLEVILDVVYNHTAEGNELGLSMRGID
48785916_Burkholderia_fungor        GFFAADPRFVGEFKEMVDRFHNANLEVILDVVYNHTAEGNERGISFKGID
29840170_Chlamydophila_caviae       NFFSPCRRYIREFKTLVKALHKAGIELILDVVFNHTGLDNTTCLPW--ID
15604761_Chlamydia_tracho           NFFCPSRRYAREFKTLVKALHRAGIEVILDVVFNHTGFEGTSCLPW--ID
53756174_Methylococcus_capsul       AFQAPHAGYVEEFKRMVKALHEADIEVLLDVVFNHTAEGDETGLNFRGLD
87311865_Blastopirellula_marin      AFFAPHRGYVREFKEMVKALHQAGIEVILDVVYNHTCEGNEMGLGFKGLE
32446352_Rhodopirellula_baltic      AFFAPHRGYVNEFKQMVKALHSAGIEVILDVVFNHTCEGNEQGLSFKGLE
48837599_Methanosarcina_barker      CFFAPHSGYMEEFRDMVRALHKAGIEVVLDVVFNHTAEGDNLGFSFKGID
48893487_Trichodesmium_erythr       AFFAPHHSYMDEFRDMVKALHKEGIEVILDVVFNHTAEGNENGVSFRGLE
16329290_Synechocystis_sp.          GFFAPHQGYVDEFRDMVKALHKAGIEVILDVVFNHTAEGNEKGLSFRGID
CM4050_Cyanidioschyzon_merolae      SFFAPMNRYVREFKTMVRELHRHGIEVILDVVFNHTGEFGDDGYHFKGLA
34098817_Arabidopsis_thalia         GFFSPMIRYINEFKILVKEAHKRGIEVIMDVVLNHTAEGNEKGFSFRGVD
46360122_Ostreococcus_tauri         NFFSPMTRYAREFKRMIRECHRAGIEVIMDVVFNHTAEGNEQGLSFRGLD
22136708_Arabidopsis_thalia         NFFAPMSRYSKEFKEMVKALHSAGIEVILDVVYNHTNEADDKYTSFRGID
46446740_Protochlamydia_amoebo      NFFSPMNRYLVEFKTMVKELHRHGIEVILDVVYNHTFEGNQMGQSFRGLD
82748503_Clostridium_beijer         CFFAPNTSYGDELKSLIKKFHEDGIEVILDVVFNHTAEGNEYGISFKGFD
48894542_Trichodesmium_erythr       GFFAPKAGYMDELKTLVKDLHKNGIEVILDVVFNHTAEGNENGISFRGID
16330244_Synechocystis_sp.          NFFAPKAGYIDELKNLVKELHKVGISVILDVVFNHTAEGNERGISFRGLD
                                       .    :   :   :   *  .: :::*:* **:   .        .

19714344_Fusobacterium_nuclea       ENIFYTKDRENYFTNFSGCGNTLNCNHKVVKDMIIQSLLYWYLEVGVDGF
32398065_Rhodopirellula_baltic      SSTAYMMTGNAPYANHSGTGNTLHTANRAVRRMIVDSLRFWDSQMHVDGF
42525620_Treponema_dentic           NSIYYHLEDKFYYKNFSGCGNSLKTSEIPVIKFILDCLRYWVTEMHVDGF
29144251_Salmonella_enteri          NRSYYWIRDDGDYHNWTGCGNTLNLSHPGVVEYACECLRYWMETCHVDGF
48765636_Rhodospirillum_rubrum      NAAYYRLDPPRRYLNDSACGNTLRMEHPQVLRLAMDALRYWVGVMGVDGF
1707700_Sulfolobus_solfat           NTAYYMLQPKRYYLDFTGTGNTLNLSHPRVIQMVLDSLRYWVTEMHVDGF
39936707_Rhodopseudomonas_palu      NASYYWLKPPRYYDDFTGCGNSMNLTHPRVLQMVMDSLRYWVEVCHVDGF
6457937_Deinococcus_radiod          NPTYYRLVAQRFYFDYTGTGNSLNVRHPQTLQLIMDSLRYWVTEMHVDGF
48894700_Trichodesmium_erythr       NAAYYRLVEPRYYMDFTGCGNSLNVRHPQILKLIMDSLRYWVTEMHVDGF
57116886_Mycobacterium_tuberc       NTAYYRLMDLRFYKDFTGTGNSLNARHPHTLQLIMDSLRYWVIEMHVDGF
8246833_Streptomyces_coelic         NASYYRLVDWQHYYDTTGTGNSLLMRHPYVLQLIMDSLRYWVTEMHVDGF
73538060_Ralstonia_eutropha         NASYYQLVPERHYLNDTGCGNMLNLPHPRVLQMVLDSLRYWVQCFHVDGF
48784580_Burkholderia_fungor        NASYYRLIPERHHINDTGCGNTVNLPHPRVLQMVMDSLRYWSTAFNMDGF
48763460_Rhodospirillum_rubrum      NRSYYYLMGLRHYNNFTGTGNALELRHPRVLGMVMDSLRYWATAMGVDGF
29141918_Salmonella_enteri          NFSYYRTMPHRYYINDTGTGNTVNTSHPRVLQMVMDSLRYWAESMHVDGF
39936374_Rhodopseudomonas_palu      NASYYRLLPKRYYINDTGTGNTVNLTHPRVIQMVNDSLRYWAGEMHIDGF
71737201_Pseudomonas_syringae       NASYYRLMPKRYYINDSGTGNTLDLSHPCVLQMVTDSLRYWASEMHVDGF
48785916_Burkholderia_fungor        NASYYRLMPPRYYINDTGTGNTLNLSHPRVLQMVTDSLRYWVTEMKVDGF
29840170_Chlamydophila_caviae       LSSYYMVNSQGEFANYSGCGNTVNTNYTPTTQWILDSLRYWVQEMHVDGF
15604761_Chlamydia_tracho           LESYYMVNDHGDLMNFSGCGNTVNTNTPTTLKWILDALRYWVQEMHVDGF
53756174_Methylococcus_capsul       NSIYYLLEERRHYRNYSGCGNTVNCNHPVVRSYILDCLRYWVVEMHVDGF
87311865_Blastopirellula_marin      NQVYYMMANGSHYKNYSGCGNTVNGNHPIVREMIFHSLRHWVHNYHIDGF
32446352_Rhodopirellula_baltic      NQVYYILSEGQHYCNYSGCGNTINGNHPVVREMIFHCLRHWVHNYHIDGF
48837599_Methanosarcina_barker      NSIYYLLEPEQYYSNYSGCGNTVSCNHPISQKLIVDCLKYWAKEMHVDGF
48893487_Trichodesmium_erythr       NSTYYILEKRGYYSNYTGCGNTFNTNNPFVHRLIVDCLCYWVREMHVDGF
16329290_Synechocystis_sp.          NRTYYILDEKSSYSNYSGCGNSVKANHPVVGGLILDSLRYWVSEMHVDGF
CM4050_Cyanidioschyzon_merolae      LSTYYILDRKNQFANYSGCGNSLSANHVVTAEFIHECVRYWALEMGVDGF
34098817_Arabidopsis_thalia         NSVYYMLAPKGEFYNYSGCGNTFNCNHPVVRQFILDCLRYWVTEMHVDGF
46360122_Ostreococcus_tauri         NRVYYMVAPEGQFYNYSGCGNTMNCNHPVVREFILECLRYWVLEYHIDGF
22136708_Arabidopsis_thalia         NKVYYMLDPNNQLLNFSGCGNTLNCNHPVVMELILDSLRHWVTEYHVDGF
46446740_Protochlamydia_amoebo      KHAYYMIDEQGNYLNFSGCGNTFNANHPIVKEFIIQSLRYWVTEMRVDGF
82748503_Clostridium_beijer         NNVYYMLTPDGKYYNSSGCGNTLNCNHPIVHRMILDCLRYWVTEYRVDGF
48894542_Trichodesmium_erythr       NKTYYMLTPEGYYFNFSGCGNTINCNNPIVRNVVLDCLRYWASEYHIDGF
16330244_Synechocystis_sp.          NKTYYMLTPEGYYFNFSGTGNTLNCNNPIVRGMVLDCLRYWTAEFHIDGF
                                        *         : :. ** .            ..: .*     :***

19714344_Fusobacterium_nuclea       RFDLAPVLGRDSNQWARHSLLHELIEHPILSHAKLIAESWDL-GGYFVGA
32398065_Rhodopirellula_baltic      RFDLASVFTRNSGSINLDPIVSEIGTDSDLSDDRLIAEPWDADGQFQLGK
42525620_Treponema_dentic           RFDLAPVLARDKGSIDLNFMIQAIADDSVLRSTKIIAEAWDAAGAYMVGK
29144251_Salmonella_enteri          RFDLASVMGRTP-FRQDAPLFAAIKACPVLSTVKLIAEPWDIGGGYQVGN
48765636_Rhodospirillum_rubrum      RFDLAVTPARHKAFDPSGPFLSAIAQDPLLSRALVIAEPWDVGGGYQAGR
1707700_Sulfolobus_solfat           RFDLAAALARELSVNMLNTFFIALQQDPILSQVKLIAEPWDVGGGYQVGN
39936707_Rhodopseudomonas_palu      RFDLATTLARGSGFDRNSGFFTAIRQDPILAGVKLVAEPWDLGGGYQVGA
```

```
6457937_Deinococcus_radiod           RFDLASTLARGLEVDQLSGFFTIIHQDPIISQVKLIAEPWDVGGGYQVGN
48894700_Trichodesmium_erythr        RFDLASALARELEVDSLAAFFDIVHQDPVISNVKLIAEPWDVGGGYQVGN
57116886_Mycobacterium_tuberc        RFDLASTLARELDVDRLSAFFDLVQQDPVVSQVKLIAEPWDVGGGYQVGN
8246833_Streptomyces_coelic          RFDLAATLARQFEVDRLSAFFDLIQQDPVISRVKLIAEPWDVGGGYQVGN
73538060_Ralstonia_eutropha          RFDLGATLGRDHGFDPGASFFDAILQDPVLSRVKLISEPWDLGGGYQLGN
48784580_Burkholderia_fungor         RFDLGVTLGREQGFDPGSGFFDALRQDPILSQRKLISEPWDIGGGYQLGN
48763460_Rhodospirillum_rubrum       RFDLATTLARVEPFTEHAGFLDAVAQDPVLSHVKLIAEPWDTGGGYQVGA
29141918_Salmonella_enteri           RFDLGTILGREPGFDQRGGFFDAVTQDPVLAKLKLIGEPWDIGGGYQVGG
39936374_Rhodopseudomonas_palu       RFDLGTILAREHGFDEQSGFLKSVMQDPLLSSVKLIAEPWDCGGGYQVGG
71737201_Pseudomonas_syringae        RFDLATILGRYHGFDERHSFLVACRQDPVLRQVKLVAEPWDCGGGYQVGG
48785916_Burkholderia_fungor         RFDLATILGRELGFDEGGGFLDSCRQDPVLSSVRLVAEPWDCGGGYQVGG
29840170_Chlamydophila_caviae        RFDLASVFSRDPGNPVPFPILQAISYDPVLSETKIIAEPWDAAGLYQVGY
15604761_Chlamydia_tracho            RFDLASVFSRDPGVPLPLPILQAISSDSILSETKLIAEPWDAGGLYQLGH
53756174_Methylococcus_capsul        RFDLASILGRDRGHLVPNPLLELIAEDPILRDVKLIAEAWDAGGAYLVGR
87311865_Blastopirellula_marin       RFDLASILSRDRGNLVPNPLVEAIAEDPLLADTKIIAEAWDAAGAYQVGS
32446352_Rhodopirellula_baltic       RFDLASILSRDRGNLIPNPMVELIAEDPMLADTKIIAEAWDAAGAYQVGS
48837599_Methanosarcina_barker       RFDEGSILSLDTGKVMKYPVIWQIELDDALGYIKVIAEAWDAAALNQVGY
48893487_Trichodesmium_erythr        RFDLASVMSRSMGDPLEDPVLWAIESDPVLAGTKIIAEAWDAAGLYQVGS
16329290_Synechocystis_sp.           RFDLASVLVRDTGVPLHGNIIWAIESDPILAGTKLIAEAWDAAGLYSVGK
CM4050_Cyanidioschyzon_merolae       RFDLAAVMCRDPGQPMASPVIERLSKDPCLRHLKLIAEPWDL-GQYLVGH
34098817_Arabidopsis_thalia          RFDLGSIMSRSSGTPISCPVIDMISNDPILRGVKLIAEAWDAGGLYQVGM
46360122_Ostreococcus_tauri          RFDLASILTRASGTPLQDPLIDAISNDPVLAGTKLIAEAWDAGGLYQVGS
22136708_Arabidopsis_thalia          RFDLASVLCRATGSPLSAPLIRAIAKDSVLSRCKIIAEPWDCGGLYLVGK
46446740_Protochlamydia_amoebo       RFDLASILCRSEGTPLNPPLIEAISHDPILSQTKLIAEAWDAGGLYQVGG
82748503_Clostridium_beijer          RFDLASILGRNEGSPMNNPLLQSLAFDPILANTKLIAEAWDAGGLYQVGS
48894542_Trichodesmium_erythr        RFDLASILGRDPGAPLANPLLETLAFDPILANCKLIAEAWDAGGLYQVGS
16330244_Synechocystis_sp.           RFDLASILGRDPGYPLANPLLETLAFDPILARSKLIAEAWDAGGLYQVGS
                                     *** .              ..        :    ::.*.**  .    *

19714344_Fusobacterium_nuclea        MPSGWCEWNGAYRDTVRQFIRGDFGQVPELIKRIFGSVDIFHGYQSSINF
32398065_Rhodopirellula_baltic       FPGRWMQWNAHYRDTMQRFVRGDCGMVSDLMTRLYGSCDLFPQPQLSVNY
42525620_Treponema_dentic            FPGRWAEWNDLFRNSVREFWLQPNPDIRHLATRVTGSADLYSRPYQSINF
29144251_Salmonella_enteri           FPPPFAEWNDHFRDAARRFWLPRNLTTGEFACRFAASSDVFKAPGASVNL
48765636_Rhodospirillum_rubrum       FPKSWMEWNDLARDGLRRFWLAN-GRSGDLAAVLAGCAARYAGPRAGVSY
1707700_Sulfolobus_solfat            FPYQWAEWNGKYRDSIRRFWRGEALPYSEIANRLLGSPDIYLTPFASINY
39936707_Rhodopseudomonas_palu       FPSQWSEWNDRYRSVMRRYWSGEGSLIGEVSRRMTGSSDMFNMPRASINH
6457937_Deinococcus_radiod           FPVNWAEWNGIYRDDMRSFWKGEGGLASEIGYRITGSSDLYEKPYASINF
48894700_Trichodesmium_erythr        FPLLWSEWNGKYRDTVRDFWRGEKETLAEFAYRFTGSSDLYALPHASINF
57116886_Mycobacterium_tuberc        FPGLWTEWNGKYRDTVRDYWRGEPATLGEFASRLTGSSDLYERPSASINF
8246833_Streptomyces_coelic          FPQLWSEWNGKYRDAVRDFWRAEDHSLGEFASRLTGSSDLYQRPRASVNF
73538060_Ralstonia_eutropha          HPPGFAEWNDKFRDTVRRFWRGDSGQRGELAARLSGSADLFDRPWASVNF
48784580_Burkholderia_fungor         HPPGFGEWNDRFRDTVRRFWRGDAGLRPDLAARLTGSADLFNKPWASVNF
48763460_Rhodospirillum_rubrum       FPPGWAEWNDQYRNTIRKFWKGDDGLLPTMAGRFSASSDVFNRPWASVNF
29141918_Salmonella_enteri           FPPGWGEWNDKYRDTVREYWKGDN-VTNDFAARLLGSGDLYDRSWSSVNF
39936374_Rhodopseudomonas_palu       FPPGWAEWNDKFRDTARDYWRGEA-PAAALAPRLSASGDVFNRAWASVNF
71737201_Pseudomonas_syringae        FPPGWMEWNDKFRDTVRAFWKGDEGQLADFAARMTASGNMFNRPQASVNF
48785916_Burkholderia_fungor         FPPGWAEWNDRFRDTVREYWKGDEGKVPDLATRLTGSGDKFNRPWASVNF
29840170_Chlamydophila_caviae        FPTRWSEWNGQYRDTIKSFLNGDGHLIGTFASRISGSQDLYPSPCNSINY
15604761_Chlamydia_tracho            FPSRWSEWNGCYRDHVKAFLNGDAHQVSSFASRISGSHDIYPKPTNSINY
53756174_Methylococcus_capsul        FPGRWCEWNGVYRDDVRRYWRGDPGMAGAFASRLCGSADIYEAPVNSINF
87311865_Blastopirellula_marin       FANRWAEWNGRYRDDLRSFWRGDPGKLGALATRLAGSSDLYQQPYHSINF
32446352_Rhodopirellula_baltic       FGNRWAEWNGRYRDDVRGFWRGDAGTLGPLATRLAGSSDLYQPPSCSVNL
48837599_Methanosarcina_barker       FPGRWAEWNGYYRDEIRRFVRGDPGIVRRVASRIAGSPDLYQLPINSVNF
48893487_Trichodesmium_erythr        FIGRFAEWNGHYRDDVRQFVKSDPQIVEKLAARIMGSPDIYPEPNHSINF
16329290_Synechocystis_sp.           FVEWFAEWNGPFRDDVRRFVKGDNGAVPALASRLLGSPDIYYDINRSINF
CM4050_Cyanidioschyzon_merolae       FPHCWAEWNGRFRDTVRRFIKGDPHLIGDFATRLCGSEDLYARPWHSINF
34098817_Arabidopsis_thalia          FPHIWSEWNGKFRDVVRQFIKGTDGFSGAFAECLCGSPNLYQKPWHSINF
46360122_Ostreococcus_tauri          FPHVWSEWNGKFRDDVRNFIKGVDGYAGLFAERLCGSPNLYASPSASINF
22136708_Arabidopsis_thalia          FPNRWAEWNGMYRDDVRRFIKGDSGMKGSFATRVSGSSDLYQKPYHGVNF
46446740_Protochlamydia_amoebo       FYPRWAEWNGHYRDIVRRFIKGTSGHKTAFATALSGSQDLYGTPCCSINF
82748503_Clostridium_beijer          FPSRWCEWNGKYRDDIRRFLKGDSDLAATVAERITGSYDLYNGKNASVNF
48894542_Trichodesmium_erythr        FPARWAEWNGKYRDAIRKFIKGDC-TVGEMAQRIQGSPDLYAGPATSINF
16330244_Synechocystis_sp.           FPSRWAEWNGKYRDTVRKFIKGDAGVIGEMAQRLQGSPDLYQPPSTSINF
                                         : :**   *.  : :          .   . ..   :     .:.
```

```
19714344_Fusobacterium_nuclea       ICCHDGFTMWDLVSYNLKHNILNGENNQDGENNNHSYNHGEEGFTENPHI
32398065_Rhodopirellula_baltic      ITSHDGSTLYDLTAYNEKRNWANGNNNTDGA-LEYSWDCGWEGDGTPSEI
42525620_Treponema_dentic           VCCHDGFTLCDLLSYSEKHNEENGENNRDGSNENLSYNHGIEG-SASVEI
29144251_Salmonella_enteri          LTAHDGFTLRDCVCFNQKHNEANGEENRDGTNSNYSDNHGKEGLGGPLDL
48765636_Rhodospirillum_rubrum      ISAHDGFTLADMVSYGHRHNNANGESNRDGSAIELSANHGVEGPTNDLAI
1707700_Sulfolobus_solfat           VTSHDGFTLEDLVSYNQKHNEANGFNNQDGMNENYSWNCGAEGPTNDQNV
39936707_Rhodopseudomonas_palu      VTVHDGFTLADLFSYERKHNVANGEDNRDGSNDNHSINCGVEGPTDDPKL
6457937_Deinococcus_radiod          VTAHDGFTLRDSVTYEQKHNEANGEGNNDGHNHNITWNCGVEGPTDDPEI
48894700_Trichodesmium_erythr       VTAHDGFTLNDLVSYNEKHNEANGEDNNDGEKHNRSWNSGEEGETNDPEI
57116886_Mycobacterium_tuberc       VTAHDGFTLNDLVSYNDKHNEANGENNRDGESYNRSWNCGVEGPTDDPDI
8246833_Streptomyces_coelic         VTAHDGFTLRDLVSYNDKHNEANGEDNRDGESHNRSWNCGAEGGTKDPAV
73538060_Ralstonia_eutropha         ITAHDGFTLADLVSYNGKHNDDNGEENRDGTDNNYSANWSVEGPTDDPAI
48784580_Burkholderia_fungor        VTSHDGFTLADVTAYEQKHNEANREDNNDGHNENCSRNWGVEGESDDPAI
48763460_Rhodospirillum_rubrum      ITAHDGFTLADLVSYNGKHNEANGEDNRDGSDDNNSWNCGAEGPTDDEEI
29141918_Salmonella_enteri          ITAHDGFTLNDLVSYNDKHNEANGEDNNDGHNDNRSCNYGAEGPTDDEGI
39936374_Rhodopseudomonas_palu      ITAHDGFTLNDWASYNDKHNEANGEDNRDGHSDNRSWNCGAEGPTDDETI
71737201_Pseudomonas_syringae       ITAHDGFTLHDLVSYNDKHNEANDENNQDGSNNNLSWNHGVEGPTEDAEI
48785916_Burkholderia_fungor        IAAHDGFTLNDLVSYNDKHNEANGEDNKDGHSDNKSWNMGVEGPTDDPDI
29840170_Chlamydophila_caviae       ICSHDGFTLHDTVSYNSKHNEENGEENRDGSNANYSYNFGEEGETNNPTI
15604761_Chlamydia_tracho           ICSHDGFTLYDTVAYNDKHNEENGEYNRDGTSANYSYNFGCEGETTDPTI
53756174_Methylococcus_capsul       VTCHDGFTLNDLVSYACKHNSANGEDNRDGSDHNFSANYGCEGPTGDHGI
87311865_Blastopirellula_marin      ITSHDGFPMNDMVSYNDKHNDANGENNRDGDNHNISYNYGVEGPTRRRSI
32446352_Rhodopirellula_baltic      VTTHDGFTMNDLVSYKDKHNEANGEDNNDGDNHNISDNYGVEGPTRKKAI
48837599_Methanosarcina_barker      VTCHDGFTLNDLVSYNHKHNEANGENNRDGIENNLSWNCGVEGETEDPEV
48893487_Trichodesmium_erythr       ITCHDGFTMNDLVSYNEKHNEANCEDNRDGANHNFSWNCGIEGTTDQEEI
16329290_Synechocystis_sp.          VTCHDGFTLVDLVSYNEKHNEANGEKNRDGTNDNFSWNCGVEGETDDPKI
CM4050_Cyanidioschyzon_merolae      VTAHDGFTLYDLVSYAEKHNWDNGEENQDGESHNNSWNCGFEGDTPNPNI
34098817_Arabidopsis_thalia         ICAHDGFTLADLVTYNNKNNLANGEENNDGENHNYSWNCGEEGDFASISV
46360122_Ostreococcus_tauri         VTAHDGFTLRDCVSYNEKQNHANGEENRDGEEHNASWNCGDDGECWDPEI
22136708_Arabidopsis_thalia         VIAHDGFTLRDLVSYNFKHNEANGEGGNDGCNDNHSWNCGFEGETGDAHI
46446740_Protochlamydia_amoebo      ITAHDGFSLADLVTYNDKHNLDNGEENRDGFDHNDSWNCGIEGHSNNKKI
82748503_Clostridium_beijer         ITCHDGFTLYDLYSYNDKHNEENGWNNTDGENNNNSWNCGAEGETDDENI
48894542_Trichodesmium_erythr       ITAHDGFTLADLVSYNDKHNEANGENNNDGANDNESWNCGAEGWSDDPGI
16330244_Synechocystis_sp.          VTAHDGFTLADLVAYNGKHNYANGENGNDGANDNYSWNCGVEGPTDNPDI
                                    :  *** .: *   :  :.*  *   . **   : : : . :*      :

19714344_Fusobacterium_nuclea       ISLRKQQIKNMILILYISQGIPMLLMGDEMGRTQLGNNNAYCQDNPTTWV
32398065_Rhodopirellula_baltic      LQLRKQQVRNFFCLLMLSNGTPMFRMGDEFLQTQRGNNNPYNQDNETSWL
42525620_Treponema_dentic           ERMRMRSAKNILTTLILSAGTPMINMGDEVFRTQNGNNNAYCQDNEMSWF
29144251_Salmonella_enteri          MERRRDSIHALLATLLLSQGTPMLLAGDEHGHSQHGNNNAYCQDNALTWL
48765636_Rhodospirillum_rubrum      RSLRDRQRRNLLACLLLSLGVPMIRGGDELGQTQQGNNNVYCQDNPLNWL
1707700_Sulfolobus_solfat           VICREKQKRNFMITLLVSQGTPMILGGDELSRTQRGNNNAFCQDNEITWF
39936707_Rhodopseudomonas_palu      LGMRRQLRKNQLACLFLAQGVPLLLAGDEVGNSQAGNNNAYCQDNEIGWV
6457937_Deinococcus_radiod          NRLRGQQMRNFLATLLLGQGTPMILGGDEFGRTQGGNNNAYCQDNDISWY
48894700_Trichodesmium_erythr       LSLRNRQRRNFLVTLMLSQGVPMLLGGDEFGRTQKGNNNAYCQDNEISWV
57116886_Mycobacterium_tuberc       LALRARQMRNMWATLMVSQGTPMIAHGDEIGRTQYGNNNVYCQDSELSWM
8246833_Streptomyces_coelic         RELRGRQQRNFLATLLLSQGIPMICHGDELGRTQRGNNNAYCQDNEISWI
73538060_Ralstonia_eutropha         LALRARVAQAMLSTLLLAQGTPMLTAGDEWGRSQQGNNNAYCQDNAMSWL
48784580_Burkholderia_fungor        LATRKRVARSLIATLLMALGTPMVLAGDESLRTQHGNNNAYCQDNELSWL
48763460_Rhodospirillum_rubrum      NTLRRRQMRNMLATLLLSQGTPMLLAGDEFANSQNGNNNAYCQDNALSWL
29141918_Salmonella_enteri          NAIRERQKRNFLTTLLFSHGTPMLLAGDEFGRSQMGNNNGYCQDSEISWV
39936374_Rhodopseudomonas_palu      LSLRERQKRNMLATLLLAQGTPMLLAGDEFGRTQNGNNNAYCQDNEISWV
71737201_Pseudomonas_syringae       NTLRLRQMRNFFATLLLAQGTPMIVAGDEFARTQHGNNNAYCQDSEIGWI
48785916_Burkholderia_fungor        RQQRERQKRNLLATLLLSQGTPMILAGDEFGRTQKGNNNAYCQDNEISWV
29840170_Chlamydophila_caviae       LALRERQMRNFLLTLFLSQGIPMLQSGDEYGHSAKGNNNRWALDTDTNHF
15604761_Chlamydia_tracho           CALRERQMKNFFLALFLSQGIPMIQSGDEYGHTAYGNNNHWCLDTKINYF
53756174_Methylococcus_capsul       NAVRRRQMKNLMASLLLSRGVPMILGGDEFCRTQRGNNNAYCQDNEISWF
87311865_Blastopirellula_marin      EKTRQRQIKNMMTTLLLSQGVPMILMGDECRRTQGGNNNAYCQDNEISYM
32446352_Rhodopirellula_baltic      STIRSQQVRNMLATLLTSQGVPMIVSGDEARRTQKGNNNAYCQDTDISWF
48837599_Methanosarcina_barker      ETLRERQIKNFAAILLLSIGVPMICMGDEVRRTQKGNNNAYCQDNETSWF
48893487_Trichodesmium_erythr       EMLRLRQIKNFFTILFFSQGTPMILMGDEVRRTQLGNNNSYCQNNKSSWF
16329290_Synechocystis_sp.          NQLRLRQIKNFLTILFFSQGTPMLLMGDPVGRTQQGNNNGYCQDNELSWF
CM4050_Cyanidioschyzon_merolae      FFLRQKQMRNFLVALLISTGTPMVLMGDEYGHTRRGNNNAWCQDNELNYM
34098817_Arabidopsis_thalia         KRLRKRQMRNFFVSLMVSQGVPMIYMGDEYGHTKGGNNNTYCHDNYMNYF
46360122_Ostreococcus_tauri         VALRDRQMRNFVVALFVAQGVPMMYMGDEYGHTKCGNNNTYCHDNALNWI
```

```
22136708_Arabidopsis_thalia         KSLRTRQMKNFHLALMISQGTPMMLMGDEYGHTRYGNNNSYGHDTSLNNF
46446740_Protochlamydia_amoebo      VALRERQIRNFLLALLVSQGIPMILMGDEYAHTRDGNNNTWCQDNKLNWF
82748503_Clostridium_beijer         IKLRRKLIKNACAVLLSSQGAPMLLSGDEFGNTQFGNNNPYCQDNEISWL
48894542_Trichodesmium_erythr       NALRSRQMRNAIAIMMVSQGVPMLLMGDEIGRSKNGNNNTYCHDSELNWL
16330244_Synechocystis_sp.          LRLRARQMRNAIAILLVSQGVPMLLMGDEMGKTQDGNNNTYCHDSPFNWL
                                       *    :     :  . * *:.  **   .:  **** :  :.

19714344_Fusobacterium_nuclea       DWDREDVFLFTKNMINLRKSYSIFKKETPLEVILHGFYIAFNSYTEQLCF
32398065_Rhodopirellula_baltic      DWDRADIFNFVREVISFRKSHPSISRSRFWDVRWYGIYVMINAGRDSVEF
42525620_Treponema_dentic           DWELKDLLEFTKKLINLRKTHFSFLRKHFFDITWFDFYVMTNSYNNDITV
29144251_Salmonella_enteri          DWQQRGLTTFTAALIRLRQQIPALTGNSWWNVRWLNFLIAINATLEVTDI
48765636_Rhodospirillum_rubrum      DWGGANFAAFAARAGALRARLPQLGRASFLDVVWLRLLVLINGQPHEQSF
1707700_Sulfolobus_solfat           DWNLSKFLEFVKKMIQFYRAHPAFRRERYFDVTFYTFLIILNANPNNVKV
39936707_Rhodopseudomonas_palu      GWDGDDMVEFVGLMTDIRRRFPQLRSRRWLGVLWLTIFIVLNSGVQEIEF
6457937_Deinococcus_radiod          DWEKEELLAFTRKLIALRKAHPSLHRRKFFDIVWLRLLLLLSSSYVDLPF
48894700_Trichodesmium_erythr       NWNLLDLLDFTRELIYFRFQHPVFYRRKWFDIGWYNFMIFFNAHYELIEF
57116886_Mycobacterium_tuberc       DWSLADLLAFARKATTLRKNHKVFRRRRFFDIAWLTFLLCFNAHDHDVEF
8246833_Streptomyces_coelic         DWRLRALLDFARRLIALRADHPVLRRRRFFDLVWLLFLLLLNSHWEPADF
73538060_Ralstonia_eutropha         DWKQQVLLQMVRRLLALRLRLPSLGANHFSDIAWFDTLLLLNAGDTDVVF
48784580_Burkholderia_fungor        DWARRQMTAFVARVIALRKQHPLLRETRFLDVGWFDLLMMLNANEKTLRF
48763460_Rhodospirillum_rubrum      DWDGRSQIAFVTSLLRLRREHVVFHRTRFFDVVWLRFLLVLNASHTEVVF
29141918_Salmonella_enteri          HWDNNALREFTRHLIQLRATQPLLRRESWREIRWFN--------------
39936374_Rhodopseudomonas_palu      HWDHQHLRAFTQRLLELRRTTPTLRRSRFLDVTWINVLLVLNGFDGMVEF
71737201_Pseudomonas_syringae       NWDLAALLKFVTRVIKLRQTYPILRRSRFLDVTWLALLLVVNAHHDGVNF
48785916_Burkholderia_fungor        DWEARALIEFVRNLTTLRHRLPVLRRGRFLDARWISLLLVLNAHHDVVNF
29840170_Chlamydophila_caviae       LWDENALFDFVCNAIRFRKQHKEIFNQGFLNISWLDLFTAFYTGEEKIEI
15604761_Chlamydia_tracho           LWDRKELFSFLCQVIALRKAYTELFNTSFLTITWLNLFVAFYSGNERIEI
53756174_Methylococcus_capsul       DWRLRSFFEFVRKMIAFRARHPVLSREQFYDILWFSLCLLFNPTAEGLCF
87311865_Blastopirellula_marin      NWKLADLRRFVKALVAFRRDQPTVRQKHFLDVNWYSVLLIMNSSAAPAQF
32446352_Rhodopirellula_baltic      DWRLADLVRFVSALIDFRKNQPTIRRREYLDVSWYGVILMFNSTGDHREF
48837599_Methanosarcina_barker      DWDLHDMFRFWKLMIDFRKRHTTILRPRYFDISWHGIHVLMNMYWEPLEF
48893487_Trichodesmium_erythr       DWSQNDLLRFVKKIIHFTQELQITQLDKVLNISWHGFHIMLNAYWKPLMF
16329290_Synechocystis_sp.          DWSGSDETHFLRGIIALTQSLSLFLEESLLEMYWHGLHLIFNAYWEPLNF
CM4050_Cyanidioschyzon_merolae      DWDKRGLVRFTRLMILFRRVNTFLCRQNFVDVSWIHLLVGFNAGPERRTV
34098817_Arabidopsis_thalia         RWDKSDFFRFCRILIKFRDECESLGLNDFPRLQWHGIYVAFNTSHLATLV
46360122_Ostreococcus_tauri         DWSEDGLARFTKQVIALRKKHSAFRLDSFPNIQWHGFYIAFNSHHEPAML
22136708_Arabidopsis_thalia         QWKEQNHFRFFSEVIKFRHSHHVLKHENFLEITWHEIYVAFNAHDYFVKA
46446740_Protochlamydia_amoebo      LWDKQSVFRFFKSLITFRKNYPLLKRDTFLDVTWHGLYVAFNASHVVLTV
82748503_Clostridium_beijer         NWSLHDLFTFFKNMINFRKRHPAIKDRIEEPVSKHGVYLCINAHWETQYV
48894542_Trichodesmium_erythr       NWELAELFRFVKNCVAFRKAHPILKNQNHFDITWHGIYVAMNMHWDSLWF
16330244_Synechocystis_sp.          NWHLQAWFRFVKHCIAFRLAHPVLRNSEHFDISWHGIYVAMNMHYESLWF
                                     *       :      :

19714344_Fusobacterium_nuclea       ELPWYILTDTPKSSVIL
32398065_Rhodopirellula_baltic      GIQWRLAIDTARSICVL
42525620_Treponema_dentic           RLPWHRLIDTSRTTVVL
29144251_Salmonella_enteri          VLPWRAVPPFWQGLCVF
48765636_Rhodospirillum_rubrum      RLPWTIALR-EMSLVAL
1707700_Sulfolobus_solfat           KFPWELVISSGRTALVY
39936707_Rhodopseudomonas_palu      HLPWKELFDTPRSVLAF
6457937_Deinococcus_radiod          KMPWDLLLDTGRSVKLY
48894700_Trichodesmium_erythr       TIPWVIIIDTEHSIVVL
57116886_Mycobacterium_tuberc       VMPWTGELDTARSLLVL
8246833_Streptomyces_coelic         RLPWTALVDTQRSLVLL
73538060_Ralstonia_eutropha         RLPWQLVLDSAHSLVLL
48784580_Burkholderia_fungor        TPPWHVLLDTQHGPRLL
48763460_Rhodospirillum_rubrum      TLPWETVIDTGRSLQVL
29141918_Salmonella_enteri          -----------------
39936374_Rhodopseudomonas_palu      TLPWSLLIDTARSVLLF
71737201_Pseudomonas_syringae       TLPYECLIDTPRSLLLF
48785916_Burkholderia_fungor        TLPWTCLLDTARSLLLL
29840170_Chlamydophila_caviae       HLPYQKVADSAHMMLVA
15604761_Chlamydia_tracho           SLPYEKIVDSPYSSLVA
53756174_Methylococcus_capsul       RLPWIKAVDTDRSLVVL
87311865_Blastopirellula_marin      ILPWRMFVDTERSMRVY
```

```
32446352_Rhodopirellula_baltic      HFPWNLFVDTRHSMRIY
48837599_Methanosarcina_barker      EIPWYRAVDTGRSVVVL
48893487_Trichodesmium_erythr       ELPWYLIVNTPRAAVIL
16329290_Synechocystis_sp.          ELPWHRLVDTPRSSVVL
CM4050_Cyanidioschyzon_merolae      TVPWLRIVDTPWSSIIL
34098817_Arabidopsis_thalia         SLPWEPFVDTSYSSIIL
46360122_Ostreococcus_tauri         KLPWKLILDTDRSAVIF
22136708_Arabidopsis_thalia         LIPWFRVADTPFSSILL
46446740_Protochlamydia_amoebo      TIPWVWLVNTSYTSIAL
82748503_Clostridium_beijer         RLPWRIAVNTPRSVMIL
48894542_Trichodesmium_erythr       NIPWHIFVNTDRSIVIL
16330244_Synechocystis_sp.          ELPWHVFANTDRSVVIL
```

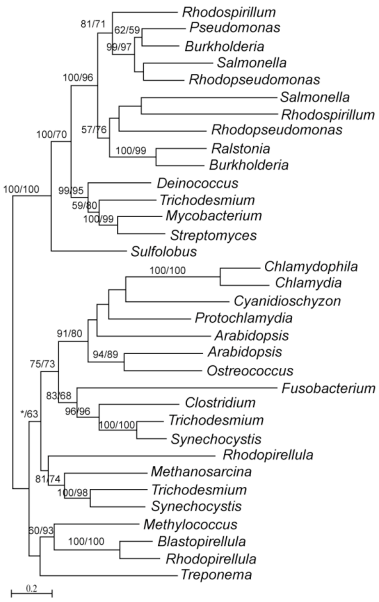

Figure 13. Phylogeny of isoamylase, showing red algal, green plant and chlamydial sequences form a monophyletic group that is distinct from cyanobacterial homologs. There are multiple homologous copies of this gene in cyanobacteria, but neither of them appears to be closely related to chlamydial, red algal and plant homologs.

---

14. Phosphate transporter

CLUSTAL X (1.83.1) multiple sequence alignment

```
94413958_Pseudomonas_aerugi         VSLVLALIFVLSFEFINGFHDTANAVATVIYTKAMSPYRAVMLSGVFNFL
15607685_Mycobacterium_tuberc       FLLLIVVVTALAFDFTNGFHDTGNAMATSIASGALAPRVAVALPAVLNLI
90588650_Flavobacterium_johnso      TLLILIIVLALIFDYINGFHDAANAIATVVATKVLTPFQAVVWAAFFNFL
116254783_Rhizobium_legumi          PLLVGLVVIALFFDFLNGLHDAANSIATIVSTRVLRPQYAVAWAAFFNFI
113868249_Ralstonia_eutrop          WVIALLVALALLFDFMNGFHDAANSIATVVSTGVLKPHHAVAMAAMCNVV
73747989_Dehalococcoides_sp.        YLLIIILLAIGF--AFVNGTNDTANAIATVVGTRVLSPRKAIIMAAVANLV
94985848_Deinococcus_geothe         IGLIVIIALALAFDFINGFHDTANAIATSVATKVLTPAQAITMAAVMNII
82749132_Clostridium_beijer         TITVLIVITVLIFDFINGFHDTATAVATSITTRALKPQTAIVICAIFNFI
30019602_Bacillus_cereus            ILTVLVVICALAFDFINGFHDTANAIATAVSTKALKPRHAIIMAAIMNFL
```

```
50256150_Cryptococcus_neofor        YLFAFGVLFAALDAYNIGANDVANSFATSISSRSLTLRQAAMLAAICEFL
82780741_Dunaliella_viridi          WLLVIGSFTSFMAAWGIGANDVANSFATSVGSKALTMFQACVIAGIFEFV
68073475_Plasmodium_berghe          WLVIASGVACFFMAFVTGANDIANTFSTSIGAKSLTIKKALLIAFFFEAL
115503941_Trypanosoma_brucei        WVIIVGGIVSFLTGCGVGMNDLANSFGTTYGSRVLNLWQIVILASICELV
5803173_Homo_sapien                 WMVILGFIIAFILAFSVGANDVANSFGTAVGSGVVTLRQACILASIFETT
21356511_Drosophila_melano          WMVVIGFLIAFVLAFGIGANDVANSFGTSVGSGVLTIRQACVLATICEIS
32267370_Helicobacter_hepati        LLLVFATIIGGYMAMSIGANDVANNVGPAVGSHAITLVGAIIIAAICEAM
15888000_Agrobacterium_tumefa       VIIIAAVALGAYMAINIGANDVTNNVGPAVGAKAMPLAVALIVAAICEIA
56695869_Silicibacter_pomero        IVVVAAAAFGAYMAINIGANDVANNMGPAVGANALSMGAAIAIAAVAESA
116250630_Rhizobium_legumi          VLVVAAAALAGYMALNIGANDVTNNVGAAVGARAMTMGQALVIAAIFEVL
15609418_Mycobacterium_tuberc       VILVLATVVGMFMAFNVGGNDVANSFGTSVGAGTLTMKQALLVAAIFEVS
59801910_Neisseria_gonorr           ALFIIATMFGVFMAFNIGGNDVANSFGTSVGAGTLTIPQALLIAAVFEVS
73852587_Emiliania_huxley           WIVVFGAFAAFFAAFGIGANDVANAYATSVGSKALTIKQACFLAVIFEFL
28569257_Tetraselmis_chui]          WVVVCGALLAVFVAFGIGANDVANAFGSSVGSKAITIKQALLIAAIFEFL
CM4066_Cyanidioschyzon_merolae      VLVCLSLCIAFLLSAALGANDVANALGTSVGTGAVSIGKALAIGAVCEFA
75906380_Anabaena_variab            LTLPIVALLAFYVAWNLGANDVANAMGTSVGSKAISLKQAIIIAGVLEFT
15618590_Chlamydophila_pneumo       PLIIFVLLCGFYTSWNIGANDVANAVGPSVGSGVLTLRQAVVIAAIFEFF
15605425_Chlamydia_tracho           LLLVCVVVGGFYTAWNIGANDVANAVGPSVGAGALTLKQAVLIAAVFEFL
15643031_Thermotoga_mariti          TILIIAGILGFIMAFSIGANDVANSMATAVGARAITVRQAALIAMFLEFL
71278581_Colwellia_psychr           TFVILAAVVGFFMAWGIGANDVANAMGTSVGSKALTIKQAIIIAMIFEFA
68545472_Shewanella_amazon          WLIAIAAAFGFLMAWGIGANDVANAMGTSVGSNAITIKQAIIIAMIFEFA
69258455_Magnetococcus_sp.          IFMFLAIIFGLYMCWGIGANDVANAMGTSVGSGAITVKQAILIAAVFEFA
51246480_Desulfotalea_psychr        AMLIMAVIFGLYMTWGIGANDLANAMGTSVGAGAITVKQAIGIAIFFEFL
46445733_Candidatus_Protoc          ALLILILITGFYMAWSIGANDVANAMGTSVGSGSLTLRQAVLIAAVLEFC
115447039_Oryza_sativa              ALAYLTLLSGFYMAWNIGANDVANAMGTSVGSGALTLRQAVLTAAVLEFS
24079961_Medicago_trunca            MLSYGTLLFGFYMAWNIGANDVANAMGTSVGSGALSLRQAVLTAAVLEFS
14521600_Pyrococcus_abyssi          WLL-LTLILGLAMAWAIGANDAANSMSTAVGAGAITPKQAVLIAGILEFT
11499387_Archaeoglobus_fulgid       LFIVVAGIVGFYVAWNIGANDAANSMATSYGSRALTLKQIIIIASVLEFT
                                                     * :*  .  ..   :  :          . :

94413958_Pseudomonas_aerugi         GVLLGGVGVAYAIVH-LLPMVFSLLAAAIAWNLGTWYFGIPASSSHTLIG
15607685_Mycobacterium_tuberc       GAFL-STAVAATIAKGLIDLVFAGLVGGIVWNLLTWLLGIPSSSSHALIG
90588650_Flavobacterium_johnso      AYWVFGFGVADTVAKTAHTVILAGVIAAICWNLLTWWLGIPSSSSHTLIG
116254783_Rhizobium_legumi          AFLFFGLHVAETLGTGIIDVIFAALMGAIIWNIITWIFGIPSSSSHALVG
113868249_Ralstonia_eutrop          AIFVFHLKVAATVGTGTIDVIFGALVGAIAWNVITWYYGIPSSSSHALIG
73747989_Dehalococcoides_sp.        G-VFTGTAVARTIGKGILGTVIAGLIAAVIWSVVATRKGLPVSLTHCLVA
94985848_Deinococcus_geothe         GA-LTGTAVAKTIATDIVPLVGAAILSAILWNLYTWWKGLPSSSSHALIF
82749132_Clostridium_beijer         GAF-SGTAVAKTVGENIVSVILGVLISAIIWNIITWYFGIPSSSSHALIG
30019602_Bacillus_cereus            GAM-TFTGVAKTITKDIVDVILAALLAAIAWNLITWYYGIPSSSSHAIIG
50256150_Cryptococcus_neofor        GGVLAGAQVASTIKNGIISAFVCALAASATWLMIATRKAWPVSTTYSIVS
82780741_Dunaliella_viridi          GAVSLGGTVVKTVKGSITDGMLGASTSVAIVLFLATYLKQAVSTTHTAIG
68073475_Plasmodium_berghe          GASLLGGTVTDSIRSKIINGMFSALVGASLWLAIATCLGLPVSTTHSIVG
115503941_Trypanosoma_brucei        GAVSLGSEVTSTISGGIANGMMCALSATFIWLLFATMMSLPVSSTHSIAG
5803173_Homo_sapien                 GSVLLGAKVGETIRKGIIDGEVSAMVGSAVWQLIASFLRLPISGTHCIVG
21356511_Drosophila_melano          GAVLIGYKVSDTMRKGILEGCVAALASSAVWLLVATFLKLPISGTHSIVG
32267370_Helicobacter_hepati        GAVIAGGEVVETIKSGIIDLMLAALASGAVWLHLATAIGAPVSTTHSLVG
15888000_Agrobacterium_tumefa       GALITGGNVVETISSGIINAMLSALLAAGLLVNISTWTRAPISTTHTVVG
56695869_Silicibacter_pomero        GALLAGGDVVSTISKGIIDAMMAALISSALWVNLATWVGAPVSTTHSVVG
116250630_Rhizobium_legumi          GATVGGGEVVKTISANIVDIMMAALMAAALWINLATWMNAPVSTTHAIVG
15609418_Mycobacterium_tuberc       GAVIAGGDVTETIRSGIVDIMLSALSAAALWLLFANRMGYPVSTTHSIIG
59801910_Neisseria_gonorr           GAVIAGGEVTDTIRKGIVDIMMSALLAAALWLLFASRKGLPVSTTHAIIG
73852587_Emiliania_huxley           GAVLAGSAVAETIRKGTADGNLCVIGAVGMWLLVATKFEMPVSTTHSCVG
28569257_Tetraselmis_chui]          GAVLLGSNVTDTVRKGIANGMLSVLVATGVWLLLASYWELPVSTTHSTVG
CM4066_Cyanidioschyzon_merolae      GAVLFGSTVTKTISTGVVSGMLCVLVGCTMWLGLATRYGLPVSSTHSVVG
75906380_Anabaena_variab            GAVLFGHEVTETLATKVANGMMTVLISCGVWLQIATSRGLPVSSSHAVVG
15618590_Chlamydophila_pneumo       GALLLGDRVAGTIESSIVSGMTAALLATGVWLQLASFFGWPVSTTHSIVG
15605425_Chlamydia_tracho           GAVLLGDRVIGTIESGLVAGMTAALLATGVWLQIASFCGWPVSTTHAIVG
15643031_Thermotoga_mariti          GAVMFGSHVSQTIVKGIVEGALSALIAASFWILIATNWGYPVSTTHSIVG
71278581_Colwellia_psychr           GAYLAGGEVTSTIRKGIIDGMISALFAAATWLLIASALGWPVSTTHSIVG
68545472_Shewanella_amazon          GAYLAGGEVTSTIRNGIIDGMIAALLAAGIWLVVASALGWPVSTTHSIVG
69258455_Magnetococcus_sp.          GAFIAGGQVTKTIRKGIIDGMLAALLAAAIWLMIASSKGWPVSTTHTIVG
51246480_Desulfotalea_psychr        GAVIAGGNVTKTIRKGIIDGMLAALLAAAVWLMIASSRGWPVSTTHSIIG
46445733_Candidatus_Protoc          GAFFFGSHVSKTVQSGIIDGMLASLGSVGMWLQLASYFGWPVSTTHSIVG
115447039_Oryza_sativa              GAFLMGTHVTSTMQKGILVGLLSSLAAAGTWLQVASSYGWPVSTTHCIVG
24079961_Medicago_trunca            GALLMGTHVTSTMQKGILVGLLSSLAAAGTWLQFASYYGWPVSTTHCIVG
```

```
14521600_Pyrococcus_abyssi          GAYFFGKTVTETIRKGIIDGSLAALLGATIWLVIATKYGLPVSTTHSIIG
11499387_Archaeoglobus_fulgid       GAVFFGKKVTHTIAKGIVPGALAALLSAGFWITISTYYHLPISTTHSIVG
                                    .       *  ::             .       :     . * ::

94413958_Pseudomonas_aerugi         SILGVGLAAIDIGLSLIFSPMAGFLVAALVLLGLKWPPFWNRLVLVLSAM
15607685_Mycobacterium_tuberc       GIVGATIA---VVSKVIVPAVVAALLATLVGAVGTWTERGFRRGQIGSAS
90588650_Flavobacterium_johnso      GFAGAAVAVLIIIAFIVLAPLLGALASYLISI---WADAALKKMQLLSSA
116254783_Rhizobium_legumi          GLVGAGLALLKTVGAIFVSPAIGFLLALVLVLAVSWVDRTFRVMQFVSAS
113868249_Ralstonia_eutrop          GLVGAAVALLKTVAFILVSPLLGFLLGSLMMVMVGWVDRWFRRLQLVSAS
73747989_Dehalococcoides_sp.        GLAMAGVALLKIIAAVGIAPIMGMAGGYLLMVALLWTNGLFSHLQIFSAA
94985848_Deinococcus_geothe         SLVGAGVAVQKTLTGLLTSPLLGFFVPILLMALLSWVTRSFRWLQIFSAA
82749132_Clostridium_beijer         ALVGGGIAFHDVVIWIFIAPIIGFTVGYILMILLNWINKIFLKLQVVAGS
30019602_Bacillus_cereus            SIAGAAIAFIKIIEALIISPIIAFVIGYIVYSIFKVTNKNFRIFQIFTAA
50256150_Cryptococcus_neofor        ALAGVGVAIATIFAGFAIAPAISAGFGATVYLITKYTEHLYSFLQVLTAC
82780741_Dunaliella_viridi          SVLGFGLVFTPVVISWFVSPVFTAILSSGFFIITRITEDVFKRMQIITAS
68073475_Plasmodium_berghe          ALLGFGLSIQSIIS-WFAAPLLAGSCSAIAFSTMRSTEIVFATLQIISAI
115503941_Trypanosoma_brucei        AIIGFALVVAPIIASWFISPVFAGAVAASLYALLRLVEYVFRYLQVFTAV
5803173_Homo_sapien                 STIGFSLVLVKIVASWFISPLLSGFMSGLLFVLIRIVHLLFHFLQVLTAC
21356511_Drosophila_melano          STIGFSLVLGTIVGSWFISPVLSGIVSILLFLAIRRISMLFSFLQILTAT
32267370_Helicobacter_hepati        GILGAGVVCLRIVSSWVISPVLGGLIAVIFLIIIKKINSLFTIPLVFSAA
15888000_Agrobacterium_tumefa       GVAGAGMALGSIAFAWVLAPFISAAIAIAILTFIKELRILFQWPLVLAAG
56695869_Silicibacter_pomero        GVMGAGIAMSAIIASWVISPLLGGVIAALFLALIKHIKALFGLPLVLSAA
116250630_Rhizobium_legumi          AVIGAGISMLEITSSWITSPLIGGLIAAGLLYLVKTLRTLFRLPLIGSAA
15609418_Mycobacterium_tuberc       GIVGAAIAIGQIVVSWVLSPVLGGLVSYLLYGVIKRTFLMFSWMQVFTAS
59801910_Neisseria_gonorr           GIVGSALCLGEIGMSWVLSPVLGGAVSYFLFSRVKKTFQMFSWMQVFTAC
73852587_Emiliania_huxley           GLVGMTIAIVGIVLSWVFSPLLSGIVAVLLFLSIRTAEYVFKYIQIFSAI
28569257_Tetraselmis_chui]          GVIGMAVTVASIVLSWIFSPVLSGIFSVILFGTVRATEESFKYLQVFTAI
CM4066_Cyanidioschyzon_merolae      SLIGLGMIVLRIVLSWIISPLMGGVVAWLLFRFIRQIEQVFSILQLLTAC
75906380_Anabaena_variab            AIAGFSWVIGLITIGWIFTPVISGAIAALFYSQIKHIESIFARFQVLSAC
15618590_Chlamydophila_pneumo       AVIGFGLVVGIILISWILSPFMGGCVAYLIFSFIRRVERIFAYLQIIVAC
15605425_Chlamydia_tracho           AVLGFGIICGRVFVSWLASPIIGGYFAFLIFSFIRKVERIFAYLQMIIAC
15643031_Thermotoga_mariti          GMMGFGLVFLFIVLSWVVSPVLGGLISFVMFKLISLVENVFKRAQILTSC
71278581_Colwellia_psychr           AIVGFAAVVVGIVGSWIITPLISGIIAFIIFNSAQKVEKVFAILMIVTAC
68545472_Shewanella_amazon          AIIGFAAVVTGIVGSWIVTPAISGFIAFLIFQSAQKVEKVFAVLMVVSAC
69258455_Magnetococcus_sp.          AIVGFAVVISTVAASWIVSPIVGGGIAYMLMISIRKVEKVFMPMMVFTAC
51246480_Desulfotalea_psychr        ALVGFAIVIGSVVASWIVSPIIGGTIAYLLVMSTRKVERVFTPMMLFTAC
46445733_Candidatus_Protoc          AIVGFGTVVCYIITSWIFSPILGGIIAYYIFSLLRKVEKIFAYLQIMTAC
115447039_Oryza_sativa              AMVGFGIVLARVSSSWVISPLMGAAVSFIVYKGIRRVYGVFGYMQVLSAC
24079961_Medicago_trunca            AMVGFGLVLARVISSWIFSPLLGAAVSFIVYKCIRRVYGVFGYMQVLSAC
14521600_Pyrococcus_abyssi          GIVGYGVVMASVVLSWILSPIVGAIFAFFIFKAIRRAEFIFRRVQVITSG
11499387_Archaeoglobus_fulgid       AMVGFGLALARIALSWVISPLFGAALAFVVFSLIRVIEHVFRYLQVLTAC
                                    .                 ..                         .  .

94413958_Pseudomonas_aerugi         AVSFVHGSNDGQKGIGLIPFWVIIAVALALGIGTMVGWKRVVLTVGEKIG
15607685_Mycobacterium_tuberc       LVSLAHGTNDAQKTMGVIPLWVIVSCAVAMAAGTYLGGWRIIRTLGKGLV
90588650_Flavobacterium_johnso      AFSLGHGGNDSQKVMGIIPSWIPLACYSAIAAGTLSGGWKIVKTMGSKIT
116254783_Rhizobium_legumi          LYSLGHGGNDAQKTMGIIPFWVVISCQSAMALGTLFGGWRIVHTMGSKIT
113868249_Ralstonia_eutrop          LYSLGHGGNDAQKTIGI-PTWVIVSCYVAIGMGTLFGGWRIVRTMGQKIT
73747989_Dehalococcoides_sp.        FMAFSHGRSDGQMPMGIIPLWIIVLSALSISAGTAIGGWRVIQTVGVRMT
94985848_Deinococcus_geothe         FMAFSHGGNDAQKTMGIIPLWVILSAAAAMGLGTAMGGWRIIRTMGFKVV
82749132_Clostridium_beijer         LMALNHGSNDAQKSMGLIPTWVIACCAIAMALGTASGGKKIIKTMGSGMA
30019602_Bacillus_cereus            LQAYTHGTNDAQKAMGIIPFWVQLSCAIAMGLGTSVGGWKIIKTVGGQIM
50256150_Cryptococcus_neofor        TNSFAHGSNDLANAVGPFPVWIFVAGGLMLVIGLATYGYNIMSVLGNRLT
82780741_Dunaliella_viridi          AVAFVHGANDVANGVGPLPRWILVIGAAGIVFGLAMYGYRIIATLGVDLV
68073475_Plasmodium_berghe          LGVIAQSANDTANAIGPFQWYILLFGGLSMSLGLSILGYRVIKTVGMKLI
115503941_Trypanosoma_brucei        CASFAHGANDVSNAIAPFPLWILVLGGAGLVVGLATLGVRIMRLLGERIT
5803173_Homo_sapien                 FGSFAHGGNDVSNAIGPLPVWLLFYGGVGICTGLWVWGRRVIQTMGKDLT
21356511_Drosophila_melano          FGSFAHGGNDVSNAIGPLPIYILIYGGIGISVGLWLWGRRVIETIGNDLT
32267370_Helicobacter_hepati        LLSFAHGANDVANAIGPLPLWIMLVGGLGISLGLALYGPRLIRTVGSEIT
15888000_Agrobacterium_tumefa       LMSFAHGANDVSNAIGPVPLWVMLVGAFGLSCGILLYGPRLIRLVGEQIT
56695869_Silicibacter_pomero        LLSFAHGANDVANAVGPLPFWVMVIGAFGISFGLFLFGPKLIRMVGSQIT
116250630_Rhizobium_legumi          LLSFAHGANDVSNAVGPLPLWVMLIGAFGISVGLLLFGPRLIRLVGEEIT
15609418_Mycobacterium_tuberc       GFAFSHGSNDIANAIGPFPAAAMVTFGVALCAGLWFIGRRVIATVGHNLT
59801910_Neisseria_gonorr           GFAFSHGANDIANAIGPFPPIAMLTFGIALIVGLWFVGKEVIKTVGTSLA
73852587_Emiliania_huxley           FDSFAHGANDVANAMGPFSYWILAIGGIGIGIGLLLYGYKIMQEIGVKLA
```

```
28569257_Tetraselmis_chui]          CDSFSHGANDVANSIGPFPIWILVLGGFGIVLGLATYGYKIMCAIGVKMC
CM4066_Cyanidioschyzon_merolae      FVSFSHGSNDVSNAIGPFFPPWALVLGGLGISFGLGVWGRPVMDTVGKKIT
75906380_Anabaena_variab            FVAFAHGSNDVGNAIAPLPLWILVLGGVGIVGGLAVWGEKVIATIGENII
15618590_Chlamydophila_pneumo       FMAFAHGSNDVANAIAPV--RLMAFGGIGLVIGLAIWGWRVIETVGCKIT
15605425_Chlamydia_tracho           FMSFAHGSNDVANAIAPV--VFMSLGGLGLVCGLATWGWRVIDTIGKKIT
15643031_Thermotoga_mariti          YVSFSHGANDVANAAGPVPFLALLLGGIGISLGVFFLGQKVMETVGEKIT
71278581_Colwellia_psychr           AMAFAHGSNDVANAIGPLAWWILPLGGFGIVAGLALFGHKVIATIGQGIT
68545472_Shewanella_amazon          CMAFAHGSNDVANAIGPLVWWILPLGAFGIVFGLAIFGKRVMQTIGKNIT
69258455_Magnetococcus_sp.          AMAFAHGSNDVANGIGPLPVWILVLGGVGIVIGLATMGHKVMQTIGTKIT
51246480_Desulfotalea_psychr        SMAFAHGSNDVANGIGPLPIWILLLGGAGIVLGLITLGYRVMLTVGKKIT
46445733_Candidatus_Protoc          MMAFAHGANDVANAIGPLPTWALALGGSGIVIGLATWGWRVIETIGKKIT
115447039_Oryza_sativa              FMSFAHGGNDVSNAIGPLPTEVLAWGGFGIVAGLTMWGYRVIATIGKKIT
24079961_Medicago_trunca            FMSFAHGGNDVSNAIGPLPIDVLAWGGFGIVAGLMMWGYRVIATIGKKIT
14521600_Pyrococcus_abyssi          YVALAHGANDVANAIGPVPRWILALGGLGIAIGVATYGYRVMETVGKKIT
11499387_Archaeoglobus_fulgid       YMAFAHGSNDVANATGPIPFWVLFFGGLGIAVGIATWGYRVIETVGKQIT
                                          :. .*      .                :   *        ::  :*   :

94413958_Pseudomonas_aerugi         KMTYAQGMSAQITAAAAIGMANIYSLPVSTTHVLSSGVAGTMVAGLHGGT
15607685_Mycobacterium_tuberc       EIKPPQGMAAESSSAAVILLSAHFGYALSTTQVATGSVLGSGVGEVRWGV
90588650_Flavobacterium_johnso      KVSSFEGVAAETAGALTLYFTEHLKIPVSTTHTITGSIIGVGLTAVRWGV
116254783_Rhizobium_legumi          RLNPMQGFCAETGGALTLFGATWLGIPVSTTHTITGAIVGVGAAAVRWGL
113868249_Ralstonia_eutrop          KLKPVGGFCAETGGAITLFFASALGVPVSTTHTITGAIVGVGSAAVRWGV
73747989_Dehalococcoides_sp.        NLRPINGFAAETAAATVIQIAASIGIPVSSTHAISSAIMGVGATAVRWGV
94985848_Deinococcus_geothe         DLRPVDGFVAETSAALIIETASRLGIPVSTTHTISASIMGVGTTKVKWQV
82749132_Clostridium_beijer         KLTPVNGFAAQTGGAAVIFLATLFHAPVSTTHIISTTVMGVGASSVKWGV
30019602_Bacillus_cereus            KIRPVNGVAADLSSSLVIFGATFIHLPVSTTHVISSSILGVGASGVKWGT
50256150_Cryptococcus_neofor        MHSPSRGFSMELGSSITVLLASQYGIPVSTTMCITGATAGVGIVSINWRA
82780741_Dunaliella_viridi          VMTPNRGYSVELAAAAIIALASTYGLPVSTTQVVTGGKIGVGMCGVNWLL
68073475_Plasmodium_berghe          K-TPSRGFTIELISGLVVLFFSICGIPLSSTHCAVSSVIGLGLVCVNLKL
115503941_Trypanosoma_brucei        KITPSRGFSAELSAALVVSLCSAFGIPVSSTHCITGAVVAISIMKVRWMM
5803173_Homo_sapien                 PITPSSGFTIELASAFTVVIASNIGLPVSTTHCKVGSVVAVGWIAVDWRL
21356511_Drosophila_melano          KITSSTGFTIEVGAAITVLLASKIGLPISTTHCKVGSVVFVGHVSVDWHL
32267370_Helicobacter_hepati        ELDKIRAFCIAMSAALTVLLASQLGLPVSSTHIAIGAIFGVGFLLVKRNA
15888000_Agrobacterium_tumefa       KLNPMRAFCVSVATALTVLVASRFGLPVSTTHTAIGAVFGVGFFLVRRSH
56695869_Silicibacter_pomero        KLNPMRAYCVALSAAITVIVASWLGLPVSSTHIAVGGVFGVGFYLVRRSH
116250630_Rhizobium_legumi          KLNPMRAYCVALSTAFTVIVASWLGLPVSTTHIAVGSVFGVGFFLVRRSH
15609418_Mycobacterium_tuberc       TMHPASGFAAELSAAGVVMGATVLGLPVSSTHILIGAVLGVGIVSTNWGL
59801910_Neisseria_gonorr           EMHPASGFTAELSAASVVMGASLMGLPVSSTHILVGAVLGIGLVNANWKL
73852587_Emiliania_huxley           VITPSRGVCIELGSAVVIITGSYMGIPLSTTHAQVGATVGVALLGINTKV
28569257_Tetraselmis_chui]          RITPSRGFAIELGAAIVIVIGSQLGIPLSTTHCQVGATIGVGLLGVNWKL
CM4066_Cyanidioschyzon_merolae      HLVPTRGFCVELSTALTVLMATQIGMPVSTTHTLIGSIVAMGLASVNRRV
75906380_Anabaena_variab            ALQPSSGFCAELATATTILLASRMGLPVSTSHALVGGVVGIGLVSVKFQT
15618590_Chlamydophila_pneumo       ELTPSRGFSVGMGSALTIALASILGLPISTTHVVVGAVLGIGLAAINLNI
15605425_Chlamydia_tracho           ELTPSRGFSVGMSSAITIAAASSLGFPISTTHVVVGSVLGIGFAAINLRI
15643031_Thermotoga_mariti          TLTNSRGFTVDFSTATTVLLASSLGLPISTTHVVVGAVTGVGFAMVNVGV
71278581_Colwellia_psychr           HLTPSRGFAAELAAASTVVIASGAGLPISTTQTLVGAVLGVGMAAINLGV
68545472_Shewanella_amazon          HLTPSRGFAAELAAASTVVIASGTGLPISTTQTLVGAVLGVGMAAINIGV
69258455_Magnetococcus_sp.          ELTPTRGYCATLASAVTVVLASKTGMPVSTTQIAVGAVMGVGFAALDMRV
51246480_Desulfotalea_psychr        ELTPSRGFCAELAAAITVVIASRTGLPVSTTHILVGSVLGVGLAALDLRV
46445733_Candidatus_Protoc          ELTASRGFAAEFGAATTIVIASRFGLPISTTHTLVGAVLGVGFAAVNLTT
115447039_Oryza_sativa              ELTPTRGFAAEFAAASVVLFASKLGLPISATHTLVGAVMGVGFARVRAET
24079961_Medicago_trunca            ELTPTRGFAAEFAAASVVLFASKLGLPISGTHTLVGAVMGVGFANVRSET
14521600_Pyrococcus_abyssi          ELTNTRGFTIDFSAATVVLIASWLGMPISTTHTVVGAVIGVGLAAINKSI
11499387_Archaeoglobus_fulgid       ELTYTRGFSAEFATATTVLLASNFGMPVSTTHTLVGSVIGVGLASVNLKI
                                           .         .   :          .:*  :

94413958_Pseudomonas_aerugi         VRNILMAWVLTLPTSMALSAGLF
15607685_Mycobacterium_tuberc       AGRMVVAWLVTLPLAGLVGAFTY
90588650_Flavobacterium_johnso      TVSLVWAWILTIPISAILAGLVY
116254783_Rhizobium_legumi          AGNIVIAWIVTMPAAAAISAAFY
113868249_Ralstonia_eutrop          AGNIVWAWVLTIPASAFMAAIAW
73747989_Dehalococcoides_sp.        AGNIVGAWLLTFPICGALGFLVA
94985848_Deinococcus_geothe         AGRIVTAWFTTIPICIALGWVIH
82749132_Clostridium_beijer         AQNIVWAWILTIPITAALSAFII
30019602_Bacillus_cereus            AKRMLITWVITLPISASLAALFY
```

```
50256150_Cryptococcus_neofor        FGWIFLGWVLTVPIAGTAAGCLT
82780741_Dunaliella_viridi          FIRTFWGWVGALVTGAILSALLF
68073475_Plasmodium_berghe          FRTIFLSWIIITVSFSASVTAAIF
115503941_Trypanosoma_brucei        VGKMYLGWIFTLLITAAISALLF
5803173_Homo_sapien                 FRNIFVAWFVTVPVAGLFSAAVM
21356511_Drosophila_melano          FRNIAYAWIVTVPVTALLSAGMM
32267370_Helicobacter_hepati        INKIIASWLITVPVSAVLGGVTY
15888000_Agrobacterium_tumefa       FMTIIAAWVVTVPASATLAALLY
56695869_Silicibacter_pomero        FMTIVAAWVITVPAAALMSGVIF
116250630_Rhizobium_legumi          FMTIIAAWIVTVPVSAALAAMIY
15609418_Mycobacterium_tuberc       MKPIVLAWVITLPSAAILASVGL
59801910_Neisseria_gonorr           MKPIGLAWVITLPAAAVLSMASY
73852587_Emiliania_huxley           LSKAGFGWIVTLIVAGLLAGL--
28569257_Tetraselmis_chui]          VGRVVIGWVMTLVIVGLTTSGLY
CM4066_Cyanidioschyzon_merolae      LLNILLSWFVTVPVSAALTALCF
75906380_Anabaena_variab            LQGIATAWLVTVPVSAVLSAAIF
15618590_Chlamydophila_pneumo       IKDIVLSWFITLPAGALLSILFF
15605425_Chlamydia_tracho           IKDIVLSWFITVPAGAALSIVFF
15643031_Thermotoga_mariti          LKNIVISWLLIVPTVAATSAAVY
71278581_Colwellia_psychr           VRNIVVSWVITLPVGAGLSIVFF
68545472_Shewanella_amazon          VRNIVVSWVVTLPAGAGLSIIFF
69258455_Magnetococcus_sp.          IGGIFLSWLVTLPAGGLLAAAIF
51246480_Desulfotalea_psychr        ILNIVISWVVTLPAGAFMAMLFF
46445733_Candidatus_Protoc          TRDILVSWIVTVPIGALLAIILI
115447039_Oryza_sativa              VREIVASWLVTIPVGAVLSIFYT
24079961_Medicago_trunca            VKEICASWAVTIPVGATLSVIYT
14521600_Pyrococcus_abyssi          VRDIIISWFVTVPVAGLISAIIF
11499387_Archaeoglobus_fulgid       VQKIIFSWIVTVPVAAAMTISLY
                                           *   .
```

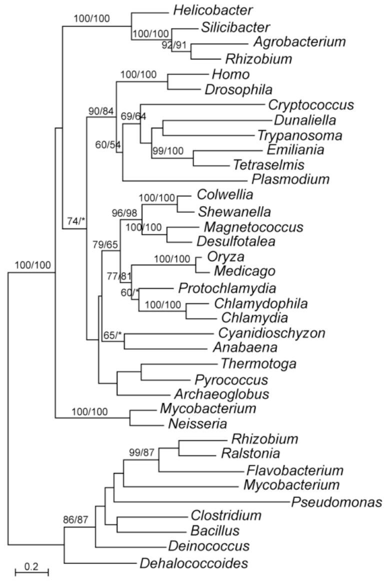

Figure 14. Phylogeny of phosphate transporter. There appears to be an ancient gene duplication and multiple subsequent genes losses, and gene transfers in the evolution of this gene. One of the paralogous copy (upper part of the tree) shows that chlamydial and vascular plant sequences form a monophyletic group, with which the red and green algal homologs are not particularly related. *Medicago* and other vascular plant sequences (*Solanum*, accession number AAT35816; *Spinacia*, accession number CAD36013) are experimentally annotated to be phosphate transporter localized in the

chloroplast inner membrane. Red algal *Cyanidioschyzon* sequence groups with cyanobacterial *Anabaena* homolog with insufficient support, but contains weak mitochondrion-targeting signal as predicted by SignalP. All other eukaryotic sequences are likely of either mitochondrial or nucleocytoplasmic origin.

---

15. Hypothetical protein

CLUSTAL X (1.83.1) multiple sequence alignment

```
7671441_Arabidopsis_thalia          LPIRVITVGKKRSEGVRLLVDEYKIKLKPYCSFEDSLVRAQVEDEEVAMK
50928573_Oryza_sativa               MPMRVLTVGKKRSRGAQLIVEEYKEKLGYYCDIEDTLIKVQVEAEDMAML
17936651_Agrobacterium_tumefa       MRISIFAVGRLKSGPEKDLAARYIERLAKTGPARVIEVGETRKREEAAMK
50365295_Mesoplasma_florum          MNIKIVCFGKLDKKFFIDSFNEYANRISKYANLQVIELKVVNKNINSDLD
33634821_Prochlorococcus_marin      CAFRIIAVGKVRKGWIQDGLAMYQKRLPGL---IITEVRASMPREAEAIA
68555467_Ralstonia_metall           MQLAIVAVGHKMPAWIETGFNEYTKRMPPELRIELREVKTVMQREAARIG
34101826_Chromobacterium_viola      MKITILAVGTKMPRWVDEAYTDYAKRFGRDITLELKEIKKGIAAEHERLA
48728976_Pseudomonas_fluore         MRLRLIAVGSRMPKWVEEGWHEYAKRLPSELAMELVEIPRFIRQEGEAMA
15836940_Xylella_fastid             MKCLLIATGERVPTWVAQGFAEYHKRLSYWLPLELVEIEQAIEDEGRRVA
48854617_Cytophaga_hutchi           MKLQLWTIGKTNDAYLKEGCAQYTKRLPHYLPFEYLEIPDVLKKEEEKLD
37520906_Gloeobacter_viola          MRIKLVAVGRLREEAYERACAEYARRLAAYARLELVEVRAGLLKEGQALD
66798153_Deinococcus_geothe         MRLHLITVGEPKLAYARAGWDEYEKRLRRYHKVQVTRVGRTGAQASEAIA
55980197_Thermus_thermo             MRLRVVAVGRPRLAYARLGVEEYARRMRRYAPLDLVFV-----RKGEELP
68177625_Desulfuromonas_acetox      MKLTILCVGKLSLPFLKEGRDEYRQRLKRYLPVDEIELKFVRNHEAEALA
94967055_Acidobacteria_bacteri      MKLRVVWIGKTKESAIQTLTGEYLKRLSRYVATEGLEIGEALLKLKDRPG
39998298_Geobacter_sulfur           MKLRVLWVGKTQEEWVRRGIDEYAGRVRRYAPLEIGEARAMRARECERLD
15643607_Thermotoga_mariti          MRVRIAVIGKL-DGFIKEGIKHYEKFLRRFCKPEVLEIKEIVRKETEDLN
CM2810_Cyanidioschyzon_merolae      LHVTILAQGKSKFQWIEEGFREYTKRLAPFVTVEQVWVRALVKRTRQ--E
46400983_Protochlamydia_amoebo      LKLRILSVGKTKEKWLEDAFNEYQKRLKANLQIE-----CLWAKDSYQLE
34763429_Fusobacterium_nuclea       MNINIICVGKIKDKYINDGIAEFSKRMTSFVTLNIIELKISIEKESLEIK
68270163_Moorella_thermo            LHLTIVAVGRVREKYLVAGIEEYLKRLRPYARVRILEAPQILASEGRGIR
51894437_Symbiobacterium_therm      MRIRLVTVGKVKEKYLQDGVQEYLKRLRPYARVEIVTVPQVMQREGERLR
67876341_Clostridium_thermo         MRITIIAVGKIKEKYLKEGINEYSKRLSRYCKLEIIEVEQVKKREAERVK
45358600_Methanococcus_maripa       MNVTIISVGKIKEKYLSDAIIEYSKRISRYSKLDIIEVAKILEKEAERIK
68195425_Enterococcus_faeciu        MNIKIISVGKLKEKYLIQGINEYVKRLNAYAKIELIEVPQVKEKEGERIA
68055317_Exiguobacterium_sp.        MQITIITVGKLKEKYLKQGIAEYTKRLGAYCSIQEIEVPQVKKKEGERIA
16799398_Listeria_innocu            MNIQIVTVGKLKEKYLVQGIAEYLKRLSAYAKVSIVEVPQVKDKEGVRIA
16081075_Bacillus_subtil            MNINIVTIGKLKEKYLKQGIEEYTKRLSAYAKIDIIELPIIKDKEGDRIS
                                         :    *                :   .

7671441_Arabidopsis_thalia          LIGSWVVVLDERGRDIDSEQMAELLISFCIGGAYGHGTQVRKRANVTIRL
50928573_Oryza_sativa               QLKPFVVVLDENGKDVTSEQVADLVLTFCIGGPYGFGLQVRERADATIRL
17936651_Agrobacterium_tumefa       HLADVLVLLDERGKALDSPAFASLFLVIAIGGADGLDSALYDKATAVLNL
50365295_Mesoplasma_florum          KLKAEIICMDVSSKNYSTEEFMSIIIVFVIGPSDGFSDKFLQQNYKKVSF
33634821_Prochlorococcus_marin      ALNSVLVPLSEEGEALTSVSFAKRLLAFVIGGADGLSAELKNSTQWQLSL
68555467_Ralstonia_metall           SLSKRIVALDERGKDWTTVQLAEQLVAFLIGGADGLDPALKARAHTLLRI
34101826_Chromobacterium_viola      AIPPRLVVMDERGKNWTSVKLAEGLVVFVIGGADGLSAELKQRADVLLQL
48728976_Pseudomonas_fluore         KVGQRIVTLEVHGKPWSTEQLAVELVNFMVGGPEGLAPEVCARADQRWSL
15836940_Xylella_fastid             ALPKYAVTLDVKGKSLNSEQLAKRMLVFLIGGPEGHSQEVLNISNERWSL
48854617_Cytophaga_hutchi           RLQDQLILLDEKGNEFTSTEFGQYILIFLIGGPYGFSDAVYKRANGKIAL
37520906_Gloeobacter_viola          QLHPHAVLLDSGGKQFTSVELADWLPVFIVGSSHGVAPMVRERAQMVWSL
66798153_Deinococcus_geothe         RAAGPLILLDPRGEQFTSESLSAYLLAFAVGGPEGHTDDLRARAHRLWSL
55980197_Thermus_thermo             KAEGRKVVLDERGRLLTTEELYRRLVAFLVGGAEGHPEAVREEADLLLSL
68177625_Desulfuromonas_acetox      RIPAYVIGLDEKGRRLSSEKLAANLVCLIIGGAYGLTEALRQRCDSLLSL
94967055_Acidobacteria_bacteri      RTAPVLVLMDERGKQVGSEELANFLLIFAIGPSDGWQKETLKSATQVLSM
39998298_Geobacter_sulfur           KLVPRLILLDERGDQLTSPEFAAYILAFAIGGAYGFADEFRRRADRVIAL
15643607_Thermotoga_mariti          RILPFVMVMDKRGEEVSSEEFADFLITILIGGPYGLNEEIFAKAHRVFSL
CM2810_Cyanidioschyzon_merolae      RASSQVVALDQRGKVYTSEEFAEVFVTFVIGGASGLPQAVYQHAHELLSL
46400983_Protochlamydia_amoebo      WTQKLIICLDPTGRLLTSEAFATFFLTIVIGGAEGLPLELKQHS-ILISL
34763429_Fusobacterium_nuclea       QISKYNILLDLDGKEINSENMSKYIINFIIGGSNGVNKELKNLVDMKLKF
68270163_Moorella_thermo            LIPPFTVALDREGVMLSSEELAGRLVALIIGGTLGLATFILQQADLRLSF
51894437_Symbiobacterium_therm      ALDQHVVVLDGRGKNFSSEELAAFLLAFVIGGSLGLDPAVLARAGTTLSL
```

```
67876341_Clostridium_thermo          RLKELLVVLDVRGSKMSSEELARKLVTFVIGGSLGIDKELLNMADFSLSL
45358600_Methanococcus_maripa        YLKKFLITLEILGKELTSEDLAKKIITFVIGGSLGLSKNISEISDFKLSF
68195425_Enterococcus_faeciu         KIKEYVYALAIEGKNPTSESFAKQILVFVIGGSLGLSEAVMKRSNAQISF
68055317_Exiguobacterium_sp.         KIGPHVYALAIEGKQRTSEQFATELIAFVIGGSLGLAPEVMQRANDTISF
16799398_Listeria_innocu             KIPDHVIALAIDGKMKSSEEFAADLVAFVIGGSLGLSEAVLKRSNEQISF
16081075_Bacillus_subtil             KISPHVIALAIEGKMKTSEELADTIVTFVIGGSLGLSDTVMKRADEKLSF
                                             :   .    :  .   .  : :* . *              :

7671441_Arabidopsis_thalia           SSMVLNHQIALVVLMEQLYRSWTILKGQNYHH
50928573_Oryza_sativa                SSMVLNHQVALIVLMEQLYRAWTIIKGQKYHH
17936651_Agrobacterium_tumefa        GKLTWPHQIVRILIAEQLYRAVTILSGHPYHR
50365295_Mesoplasma_florum           GNITLPHQLFRIILAEQIYRAFKIMNNEKYHK
33634821_Prochlorococcus_marin       SAMTLPHELARLLLVEQLYRAQTITQGGKYHR
68555467_Ralstonia_metall            SSLTLPHGMVRVLLAEQLYRAWSVTQNHPYHR
34101826_Chromobacterium_viola       SAMTLPHGMVRVMLAEQIYRAYSILNNHPYHR
48728976_Pseudomonas_fluore          SPLTLPHPLVRILIGEQLYRAWTVLSGHPYHK
15836940_Xylella_fastid              GPLTLPHMLVRLIVVEQLYRAATILTNHPYHR
48854617_Cytophaga_hutchi            SKMTFSHQMVRLFALEQLYRACTIIKGEKYHH
37520906_Gloeobacter_viola           SKLTFPHELARVIVLEQLYRAVTILAGHPYHH
66798153_Deinococcus_geothe          SLLTLPHDLAMILLLEALYRAATISAGEPYHR
55980197_Thermus_thermo              SPLTLQHELALLVLMEQLYRVLTLRAGHPYHR
68177625_Desulfuromonas_acetox       SDMTMTHQMARMFLYEQLYRAMTIIRREPYHN
94967055_Acidobacteria_bacteri       GKMTLPHELARVVLLEQLYRGYTILTGHPYHG
39998298_Geobacter_sulfur            SRMTFTHQMVRVVLLEQIYRAFTIIGNEPYHH
15643607_Thermotoga_mariti           SKMTFTHGMTVLIVLEQIFRAFKIIHGENYHY
CM2810_Cyanidioschyzon_merolae       STLTFTHQLARLVLMEQIYRAIEIYNHTPYHK
46400983_Protochlamydia_amoebo       SLLTFTHQITRLILIEQIYRATEILKNSQYHK
34763429_Fusobacterium_nuclea        SHFTFPHQLMRLILLEQVYRWFAISNNIKYHK
68270163_Moorella_thermo             SRFTFPHQLMRLILLEQLYRAFKIQRGETYHR
51894437_Symbiobacterium_therm       GRMTFLHQMVPLILLEQIYRAFKINRGEKYHK
67876341_Clostridium_thermo          SDMTFPHQLTRLILLEQLYRSFKIINGEPYHR
45358600_Methanococcus_maripa        SKMTFPHQLMRVILLEQIYRSFRIISGEPYHK
68195425_Enterococcus_faeciu         GKMTYPHQLMRLILVEQIYRAFRINAGAPYHK
68055317_Exiguobacterium_sp.         SKMTFPHQLMKMILCEQIYRAYRINRNEPYHK
16799398_Listeria_innocu             GRLTLPHQLMRLVLVEQVYRAFRIVRGEPYHK
16081075_Bacillus_subtil             SKMTFPHQLMRLILVEQIYRAFRINRGEPYHK
                                     . :.  * :  :.  * ::*   :     **
```

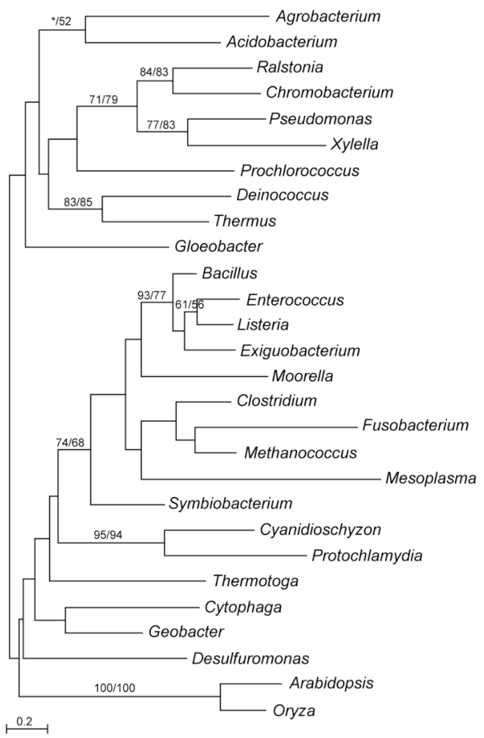

Figure 15. Phylogeny of hypothetical protein, showing red algal and *Protochlamydia* sequences form a monophyletic group. The protein sequence length of this gene is short (179 amino acids for *Cyanidioschyzon*) and the support values for many branches are low.

---

16. Beta-ketoacyl-ACP synthase (*fabF*)

CLUSTAL X (1.83.1) multiple sequence alignment

```
89298548_Tetrahymena_thermo         LKRVVVTGIGAVSPIGLNASSSWESLIAGKSGIISTSTNKLANPDNLKKW
92908594_Mycobacterium_sp.          FPSVVVTAVEATTSLAADIESTWKGLLAGESGIRVLEPVRIGGHLTRIDM
15643565_Thermotoga_mariti          MKRVVITGMGIVSPFGVGKERNLEGLRETKVTIDRISPVQIAAEVNPKLV
71367081_Nocardioides_sp.           SKRVVVTGLGTTSPVGGDVPSTWDALVNGRSGVRFLTPVKIAGRIERVKA
66820316_Dictyostelium_discoi       LKRVVVTGMGIVSPLGVGIKYNWDKLINGESGIKRFEPSKVVARVPSQVK
CM2363_Cyanidioschyzon_merolae      RKRVVVTGVGIVSCFGSDVDLFYQKLLKGESGVRPIDETRIAAWIAPKLA
18410739_Arabidopsis_thalia         QRRVVVTGMGVETSLGHDPHTFYENLLQGNSGISQIEPTRIAGEIAPKLS
116055175_Ostreococcus_tauri]       KKRVVITGMGAVTAHGDDVDAMYEKLLRGESAITSIEGTKFAGEINKKQA
15834771_Chlamydia_murida           KKRVVVTGMGIVSCLGNEVESFYDSLLAGISGVRTITATRFAGWIDKKQA
29840610_Chlamydophila_caviae       KKRVVVTGLGVVSCLGNEIDTFYDNLLAGISGVRTITATRFAAWIDKKQA
46446872_Protochlamydia_amoebo      KKRIVITGMGIVSCFGNDVDAFYQNLLAGQSGIQPITPTRFAGIIDKKQA
116061551_Ostreococcus_tauri]       KKRVVITGMGLCSVFGNNYDTYYDKLLSGQSGVAPIDPTKFAAQIDKKND
15237422_Arabidopsis_thalia         KKRVVITGMGLVSVFGNDVDAYYEKLLSGESGISLIDPTRFGGQIDGKNE
108882523_Aedes_aegypti             KRRVVITGLGVVSPVGCSVSKAWTSILSGVSSVGRLTPCRVAAQIGKSEL
8923559_Homo_sapien                 HRRVVITGIGLVTPLGVGTHLVWDRLIGGESGIVSLVPCSVAAYVSKSDI
34802538_Brachyspira_hyodys         ERRVVITGLGIVSSLGNDVKTFWDNLLNGVSGIKTLSITKIGAEADKKML
42525557_Treponema_dentic           MRRVVITGLGAVTPIGNTLDETWEGIKAGKCGIGNITKIQIAAEVDKKDA
95007196_Toxoplasma_gondii          RPRVVCTGLGVVSGVGIGIDEFWKNLIAGTNVIDKVTTCQIACEVDPKEV
32470845_Rhodopirellula_baltic      SRRVVITGLGTVTPLGCDVETLWSALLAGKSGIHELSKVHFGGDIEPKEA
85075673_Neurospora                 MRRVVVTGLGAITPLAVGIRPTWRRLLDSHSGIVSVATSTVGGLVEPSDQ
67917496_Chlorobium_limico          RKRVVVTGIGILSPIGLGKDEFWTSLVQGRSGAAPITATTFACELDRKSA
```

```
110637801_Cytophaga_hutchin         LKRVVVTGLGSLTPIGNTVPEYWDSLIKGVSGAALITKTKFACEVEKKEA
89889944_Flavobacteria_bacter       LKRVVITGMGALTPIGNNLTEYWDALKAGKSGCAPITKTKFACEIDRKEA
57237499_Campylobacter_jejuni       MKRVVVTGIGMINALGLDKESSFKAICNGESGVNKITPVQIAAEVDGKEV
76259633_Chloroflexus_aurant        DQRIVVTGLGVISPFGVGVEPFWAGLVAGRSAIGRITPCQLAGQVDAKEA
76258447_Chloroflexus_aurant        DRRVVVTGMGAISPLGLSVAELWQGIVAARSGVGPITDTRFAAEVDRKEA
46198353_Thermus_thermophi          MRRVVVTGLGALTPIGVGQEAFHKAQLAGKSGVRPITPVRIAAEVDRKEL
94984545_Deinococcus_geothe         LRRVAITGLGPVTPIGTGAQAFAQAQRAGKSGVGPITASKIAGEVDAREA
22325473_Arabidopsis_thalia         HRRVVVTGLGMVTPLGRGVETTWRRLIDGECGIRGLTSSKVAAFVNSKA-
102192140_Rickettsia_canade         NKRVVITGLGLVTPVGLNVSTSWKNIVNGVSGIKTITACKIAGFIQDNDV
83591758_Rhodospirillum_rubrum      MRRVVVTGLGMVTPLGCGVKTNWTSLVQGKSGIRPIQPARVAGQISSKDR
106895571_Clostridium_sp.           KRRVVITGMGCITPIGSSIDKFWDSLIKGVSGISNITPTQIAGEVEKREV
53688239_Nostoc_puncti              IRRVVVTGMGAITPLGNTIKEYWEGLIAGRSGIDLITICRIAGEVERKDV
23124097_Nostoc_puncti              RKRVVVTGVGAITPIGNTATEYWDGLLSGRNGIDYITDCRIAGEVERKDA
16329903_Synechocystis_sp.          KKRVVVTGLGAITPIGNTLQDYWQGLMEGRNGIGPITACRFGGEVDRKEA
67920030_Crocosphaera_watson        LKRVVVTGLGAITPIGNTLTDYWQGLMNGKSGIAEITACRIAGEVDRKEA
116622648_Solibacter_usitat         ERRVVVTGVGLISPVGIGTENTWKAITAGKSGIASITDCQIAGEVERKDV
116620290_Solibacter_usitat         SRRVVVTGVGLLTPLGIGTQTTWDALLASKSGIAGITSCRIAGEVEKKEI
47574643_Rubrivivax_gelati          RRRVVVTGLGLVTPLGNTVDESWSRLVAGQSGIATVTACHIAGEVPAKEA
82736706_Pseudomonas_putida         RRRVVVTGMGMLSPLGTDVPSTWQGILAGRSGIGPIESTRFGGSVSAKEA
29653839_Coxiella_burnet            KRRVVITGLGVVSPLGNKVSDMWQALLAGKSGVKPITPTQIAAEVDLKSI
                                       :. *.:   .  .                        .

89298548_Tetrahymena_thermo         KVTNNTNSYAMAAANEAIEDSGIESLQNQAGVNIGVMSSNISKLTEIVRE
92908594_Mycobacterium_sp.          RRMSYVQRMSKLVSGRLWETAGREVDPDRFAVIIGTGLGGGEKIVETYDA
15643565_Thermotoga_mariti          RRTDRFVHFALVSAKEAVEDAGIEPYADRTATIIGSGMGGFLTLDSENNK
71367081_Nocardioides_sp.           RRLDRSSQFAMVAAMEAWKDAGLDVDRDRLGVAMASGIGGVTTLLDNYDA
66820316_Dictyostelium_discoi       LASSDFIKYAIVATDEAIKDSKLEKLQERIGVCIGSGTGSMEEIISANEK
CM2363_Cyanidioschyzon_merolae      RRLDSVLKYTIVAGKRALEHAGLALRKDRVGVIVGSGMGGLETFAEGCEK
18410739_Arabidopsis_thalia         KRMDKFMLYLLTAGKKALADGGVEFDKTKCGVLIGSAMGGMKVFYDAIEA
116055175_Ostreococcus_tauri]       RRVDPYIAYMIVAAKRALENARVYIDKSLSGCLIGSAMGGMQTFATATEN
15834771_Chlamydia_murida           RRVDPFITYAVVAAKKAIAMSRWPANPHRCGVIIGSGMGGLRTLDEGIEK
29840610_Chlamydophila_caviae       RRVDPFITYAVVAAKKAIAMSRWPADPLRCGVIIGSGMGGLQTLDEGMER
46446872_Protochlamydia_amoebo      RRVDKSIAYTMVAGKKSLESANLRLQKERCGILIGSGMGGMTMFADGVQT
116061551_Ostreococcus_tauri]       RRLDDCLRYALVSGNKALEDAGLNIDKQRIGVLVGSGMGGLTVFQDGVKA
15237422_Arabidopsis_thalia         RRLDDCLKYCIVAGKKALESANLTIDKRKAGVLVGTGMGGLTVFSEGVQN
108882523_Aedes_aegypti             KTMARATTFALIAAKEALGMARWEEDVEACGVAIGMGMVDLTDVCETNEA
8923559_Homo_sapien                 KSMSSPTIMAIGAAELAMKDSGWEADQVATGVAIGMGMIPLEVVSETALN
34802538_Brachyspira_hyodys         RRLDPFINFGIYAAHHAFKQAGIGFDPLRAGCVLGSGIGGMTTLLSNHQV
42525557_Treponema_dentic           RKMARFTQFAVAAASQAMKDAGLNIDANRTGIILGNGIGGFEIYQEAFKK
95007196_Toxoplasma_gondii          KRNDRYTHFAVAGARLAMEDAGLNVDPERFGIVVGSGIGGLETMEQEMKV
32470845_Rhodopirellula_baltic      KRLDRFTQFAVHAGAQAVQDSGIKTDRTRCGVILGSGIGGLNEIEEQIER
85075673_Neurospora                 RRMSLFAQYSVAATEMALQDAGWQEDLEATGVCMGSGIGNLDDFYNTSVT
67917496_Chlorobium_limico          DRMDPYCQYAVIAAEQALKDSGLAIDPLRIGVVHGSGIGGMTTYDQQFRT
110637801_Cytophaga_hutchin         RKMDPFTQYAVVVADEAIKDAGIDLDHDRIGVIWGAGIGGLKTFEDEVSA
89889944_Flavobacteria_bacter       RRMDRFAQYALVAGDEAIADSGLSIDKYRVGVIWGAGIGGLETFQEEVKS
57237499_Campylobacter_jejuni       KKIDRFIQLGIKAAREAMQDAGFELDKEEFGIVSASGIGGLPNIEKNSII
76259633_Chloroflexus_aurant        RRMSRASQMAVAAARMALDHARLRMARDEIGVLIASGTTSMPDVEQAVTT
76258447_Chloroflexus_aurant        RRTDRFVQFAVAATREALRDAELPANRDEIGVFIASGIGGIGTLSEQLDV
46198353_Thermus_thermophi          RRLDRFVQYALIAAQLALEDAGLDLDPERVGTLVGTGIGGMETWEAQSRV
94984545_Deinococcus_geothe         RKLDRYVQLALAAAELAVRDSGLELRGERTGTLLGSGIGGVKTFEEQSAV
22325473_Arabidopsis_thalia         --VANFIGYAVCAADEALRDAEWEEEKERTGVSIGGGIGSICDIVEAAQL
102192140_Rickettsia_canade         NRMDKFIHYGVAAATEAVEDSGWEKSRDRTGVVLGSGIGGLKMIEDTSIK
83591758_Rhodospirillum_rubrum      RRMDDFIVYGLAAAKEAVEDSGWEESRERTGVMIGSGIGGLTSIAETALI
106895571_Clostridium_sp.           KRMDRFSQFAVAASKMAVADGNLAIDKNRFGVILGSGIGGMETMEEQSKK
53688239_Nostoc_puncti              KHISRFAQFGISASLQAIADAGFDSNATQVGVIVGNGMGGLYTVEEQEKV
23124097_Nostoc_puncti              KRMDRFAQFGVSAAKQAIANAQLELNAEQVGVMIGSGVGGIKVLEDQQTI
16329903_Synechocystis_sp.          KRMDRFCHFAVCASQQAINDAKLELNADEIGVLIGTGIGGLKVLEDQQTI
67920030_Crocosphaera_watson        KRMDRFAQFGVSASLQALADSKLDLNAEQIGVIIGTGVGGIKVLEDQQEV
116622648_Solibacter_usitat         KKLGRFAQFAVAASQMALTDAGLETNAERVGVLIGAGIGGFEVIEREYRT
116620290_Solibacter_usitat         KKMGRFIQFAIAAADFALKDSGLPEIAEEVGVYIGSGIGGFEVIEREHQT
47574643_Rubrivivax_gelati          RHMDTFIHYGLAASLQAVKDAGLEEQAERIGCLVGSGIGGLPLIEETHAE
82736706_Pseudomonas_putida         RKLDLFIQYGLAAGFQAVRNAGLDANRERIGVAMGSGIGGLTNIEETSRT
29653839_Coxiella_burnet            RKTDVFVQFAMESARQAWEDSGLETNAPRVGVAIGSGIGGMPWIEKNYDA
                                                        .         .   .
```

```
89298548_Tetrahymena_thermo         TEGFQNLNRLTILQILTNMATANVSLKYQLKGPSGTASTACATGASAIGD
92908594_Mycobacterium_sp.          MNGPRKVSPLAVQMIMPNGAAAVAGLELGARAGVITPVSACSSGSEAIAH
15643565_Thermotoga_mariti          FLGPSRVSPFLIPMILIDMASGVVAMEYGLKGPNFSSVSACASSLHAVAL
71367081_Nocardioides_sp.           LRGPRRVSPLAVPMLMPNAPAAQLSLYVGARAAVNTPVSACASGNEAISL
66820316_Dictyostelium_discoi       ISGGGKVSAYFIPKILINEASGIISILHKAKGPNMSIVSACATGSHCIGE
CM2363_Cyanidioschyzon_merolae      LSGVRRMSPFFIPYSITNMGSALLAIETDFHGPNYSLSTACATSNYAIHN
18410739_Arabidopsis_thalia         LRSYKKMNPFCVPFATTNMGSAMLAMDLGWMGPNYSISTACATSNFCILN
116055175_Ostreococcus_tauri]       LVGHRKMNPFCIPFAITNMGSALTAMDVGFMGPNYSIASACATGNFCILN
15834771_Chlamydia_murida           LSGNRKLSPFFIPYIITNMAPALIAMDYGLMGPNYSISTACATANYCIDA
29840610_Chlamydophila_caviae       LIGNKKLSPFFIPYIITNMAPALIAMDFGLMGPNYSISTACATSNYCIDA
46446872_Protochlamydia_amoebo      LVGPRKVSPFFVPFILTNMCGALLGIDIGFMGPNYSISTACATANNSIIS
116061551_Ostreococcus_tauri]       LVGPKKITPFFIPYAITNMGGAMLAIEKGFMGPNYSISTACATANYAFHA
15237422_Arabidopsis_thalia         LIGHRRISPFFIPYAITNMGSALLAIDLGLMGPNYSISTACATSNYCFYA
108882523_Aedes_aegypti             LKGYNRVSPFFVPRILPNMPAGQLSIKYGFRGPNHSVSTACATGAHSIGD
8923559_Homo_sapien                 FQGYNKVSPFFVPKILVNMAAGQVSIRYKLKGPNHAVSTACTTGAHAVGD
34802538_Brachyspira_hyodys         LLGPGRVSPFFVPMQIINMTPGLISMEYGMNGPNYSTVTACASSNHSIGL
42525557_Treponema_dentic           YFAPDRIPPMTVPLLIPNEAAGNISMQFGIKGPSWTLATACASGTDALGN
95007196_Toxoplasma_gondii          LQGPRRVSPFLIPAMIANTASGILAIELGAKGPNHGAVSACATSGHAAGV
32470845_Rhodopirellula_baltic      MLGPDRVSPFTVPKMMVNAAGGNISIRYGLKGPNYAVATACASATNAMGD
85075673_Neurospora                 YDGYKRVSPLFVPKILINLGAGHIAMRYGFRGPNHSATTACTTGAHSIGD
67917496_Chlorobium_limico          YLGPRRISPFFIPMLIPDIAAGQISMKFGLMGPNHATASACATSLHAIID
110637801_Cytophaga_hutchin         FSGTPRFNPFFIPKMIIDISAGYISIRNGFRGPNFSTVSACASSSNAILD
89889944_Flavobacteria_bacter       FAGTPRFNPFFIPKMIADIAPAHISIKYGFMGPNYTTVSACASSANAMLD
57237499_Campylobacter_jejuni       CSGPRKISPFFIPSALVNMLGGLISIEHGLKGPNISCVTACAAGTHAIGE
76259633_Chloroflexus_aurant        LIGGMKISPFFIPAALPNMPSSQVAIHFGLRGYTTAICTACAAGAQAIGE
76258447_Chloroflexus_aurant        LRGPGRISPFLIPAMITNLAAGQVSIVTGARGPSYCVTSACASSAHAIGE
46198353_Thermus_thermophi          FLGPNRISPFFIPMMIANMASAHIAMRYGFTGPSSTVVTACATGADALGS
94984545_Deinococcus_geothe         LFGPSRISPMFIPMMIANMATGHVAMRYGATGPSSTVVTACATGTGAIGD
22325473_Arabidopsis_thalia         ICRLRRLSPFFIPKILVNMASGHVSMKYGFQGPNHAAVTACATGAHSIGD
102192140_Rickettsia_canade         FYNNGKVSPFFIPASLINLLSGLVSIKYGFSGPNQAAVTACSTGAHAIGD
83591758_Rhodospirillum_rubrum      LKGPRKVSPFFIPSALINLVSGHVSIRYGFKGPNHAVVTACSTGAHAIGD
106895571_Clostridium_sp.           LLGPGRISPFFIPMMISNMGSSQISIALGAKGPNQTVVTACASATNAIGE
53688239_Nostoc_puncti              LLGSKRCSPFTVPMTIANMAAGLIAIYTGAKGPNFCTVSACAAGGNAIGE
23124097_Nostoc_puncti              YLGPDRCSPFMIPMMIANMAAGLTAIHTGAKGPNSCPVTACAAGSNAVGD
16329903_Synechocystis_sp.          LLGPSRCSPFMIPMMIANMASGLTAINLGAKGPNNCTVTACAAGSNAIGD
67920030_Crocosphaera_watson        YLGPSRCSPFMIPMMIGNMASGLTAINTGAKGPNTCTVTACAAGSNAIGD
116622648_Solibacter_usitat         LMGPSRISPFFIPATIINLAAGQVSIRTGAKGPNSAVATACTTGAHAIGD
116620290_Solibacter_usitat         LLGPRRISPFFIPATIINLASGYVSIKSGAKGPNSATATACTTSAHSIGD
47574643_Rubrivivax_gelati          YAGARRISPFFVPASIINMISGHLSIQCGFTGPNLAIVTACTTGLHCIGQ
82736706_Pseudomonas_putida         LHGPRRISPFFVPGSIINMISGFLSIHLGLQGPNYAIATACTTGTHCIGM
29653839_Coxiella_burnet            LLGPRKISPFFIPGAIINMASGMVSIKYDLKGPNISIVTACTTGLHNIGH
                                          .      :       :   .  .:      .       :**::.

89298548_Tetrahymena_thermo         AFRNIQFGDAKIMIAGGSEDSLSPTNAYASMKLQAMTTKKPEFASRPFDA
92908594_Mycobacterium_sp.          GWRQIVMGDADFAVVGGVEGGIEALPIAAFSMMRAMSTRDPAGASRPFDK
15643565_Thermotoga_mariti          GALLIRHGYADVAVVGGTEATIAPLPITGFANMRALSRRDPKRASRPFDK
71367081_Nocardioides_sp.           AVDQIRLGRADVVLAGGTEAAIHPLPMAAFANMMALSKTDPTAVSRPWDV
66820316_Dictyostelium_discoi       SFRKIKYGEVDAMICGGTEASINSVSMIGFSRMKALSTKNPSSSSRPFDE
CM2363_Cyanidioschyzon_merolae      AYMHIARGDADMMLCGGSEAAVLPIGLGGFIACRALSKREPHRASRPWDK
18410739_Arabidopsis_thalia         SANHIIKGEADVMLCGGSDAVIIPIGLGGFVACRALSQRDPTKASRPWDT
116055175_Ostreococcus_tauri]       SVMHIQNGEANLMLAGASDAAVIPIGMAGFSAAKALSTRAPAAASRPWDA
15834771_Chlamydia_murida           AYQHLVEGRADVIVCGGTEAAINRVGLAGFIANRALSERAPEQASRPWDR
29840610_Chlamydophila_caviae       AYQHLVSGRSDMIVCGGTEAAVNRVGLTGFIANRALSERAPQEASRPWDR
46446872_Protochlamydia_amoebo      AAQHIRNGEADLMLCGGVEAAVMPMGLAGFCACKALSQRNPAKASRPWDQ
116061551_Ostreococcus_tauri]       AASHIRKGDADIMVAGGSEAPIIPVGLGGFVACRALSQNDPEGASRPWDK
15237422_Arabidopsis_thalia         AANHIRRGEADMMIAGGTEAAIIPIGLGGFVACRALSQRDPQTASRPWDK
108882523_Aedes_aegypti             AFRFVQRGDADVMVCGGAEACISPLGIAGFCRLRALSTSDPQRSSRPFDD
8923559_Homo_sapien                 SFRFIAHGDADVMVAGGTDSCISPLSLAGFSRARALST-DPKLACRPFHP
34802538_Brachyspira_hyodys         GYKHIKDNEADIMVVGGSEATINPLTIAGFNNARALSTRDPAKASRPFDK
42525557_Treponema_dentic           ALDLVRSGRLDVCVSGGTEATITGFGISGFTILQTLASGDPAKACCPFDK
95007196_Toxoplasma_gondii          ALRYLQMGEADIVICGGAEASITPLSFAGFNALKAMAQGDPKRASRPFDK
32470845_Rhodopirellula_baltic      ALRSIRGGETDVVITGGTEAAITRMGLAAFQNMKALSTREPQRASRPFDS
85075673_Neurospora                 ASRFIAFGDADVMVAGGSESCIHPLTFAGFGRSRSLSTADPTSACRPFDK
67917496_Chlorobium_limico          AWMILQLGMADYMVCGGSEAPITPMSIGGFNSSRALSTARPEKASRPYDR
110637801_Cytophaga_hutchin         AFNYIRLNKANIIIAGGSEAAVVESGIGGFNACRALSERDPATASRPFDK
```

```
89889944_Flavobacteria_bacter        AVNYIRLGHCDVIVTGGSEAAVAQAGMGGFNAMHALSTRSPETASRPFDA
57237499_Campylobacter_jejuni        AYKSIALGNAKKMLVIGAEAAICPVGIGGFASMKALSTRDPEHASRPFDK
76259633_Chloroflexus_aurant         AAEIIRRGDAEVMLAGGAEAPITALSLGAFCVMRALSTREPTRASRPFDA
76258447_Chloroflexus_aurant         AAETIRRGWAKAIIVGGSEASITPIGVAGFNAMRALSTRDYAHASRPFDA
46198353_Thermus_thermophi           ALRMIQLGEADLVLAGGTEAAITPMAIGAFAVMRALSTREPEKASRPFTL
94984545_Deinococcus_geothe          AARYIQLGLADVMIAGGSEAAVTPIAVGGFSNMKALSTREPEKASRPFSA
22325473_Arabidopsis_thalia          ATRMIQFGDADVMVAGGTESSIDALSVAGFSRSRALSTKSPQEASRPFDC
102192140_Rickettsia_canade          AMRMIKHGYVDVMVAGGAEAPITPVGVAGFVAARALCTKNPEKASRPWDK
83591758_Rhodospirillum_rubrum       ASRLIIHGDADVMVAGGAESAVCRIGIAGFAAARALSTHTPEEASRPYDK
106895571_Clostridium_sp.            AFRTIQNSDADIMITGGTEASITPLSIAGFCSMKAMSTRDPTKASRPFDA
53688239_Nostoc_puncti               AFRSIQLGHALGMICGGTEAVITPLSIAAFGSAKTLSTAEPTRASRPFDR
23124097_Nostoc_puncti               AFRLIQGGYAKAMICGGTEAAVTPLSMAGFAACKALSFRDPAHACRPFDR
16329903_Synechocystis_sp.           AFRLVQNGYAKAMICGGTEAAITPLSYAGFASARALSFRDPLHASRPFDK
67920030_Crocosphaera_watson         AFRLVQRGYAKAMICGGTEASVTPLSLAGFASARALSTREPQKASRPFDK
116622648_Solibacter_usitat          AFKLIQRRAADAMICGGTEAAITPLGVAGFCAMRAMSTRDPERASRPWDR
116620290_Solibacter_usitat          SFRMIQRGDAVAMICGGTEAAVTPMGIGGFAAMRALSTRDPERASRPWDK
47574643_Rubrivivax_gelati           SARMIEAGDADAMIAGGAESTVSPLGIGGFAAARALSTRDPATASRPWDR
82736706_Pseudomonas_putida          AARNIAYGEADVMIAGGAEMAACGLGMGGFGASRALSTREPSRASRPWDK
29653839_Coxiella_burnet             AARMIAHNDADAMIAGGTEMASTPLGIGGFAAVRALSTREPEKASRPWDK
                                     .   :        :  . :         .     ::        . *:

89298548_Tetrahymena_thermo          DRSGFVLGEGAGILVLEELQHALSRNAKIYAELVSYGQSSDGYHLTRPIE
92908594_Mycobacterium_sp.           NRDGFVFGEAGAMMIIETEEHALARGAKPLARLMGAGITSDAFHMVAPAA
15643565_Thermotoga_mariti           DRDGFVMGEGGAVLILEAEEVAKSRGAKILAEIKGVGMTDDAYHFSAPDP
71367081_Nocardioides_sp.            ARDGFVLGEGAGVLVLESEEHARARGARIYATVLGAGITADSHDIAQPDP
66820316_Dictyostelium_discoi        GRDGFVMGEGSGILILEEYEHALKRGANIYCEITGYGSTGDAHHISAPNS
CM2363_Cyanidioschyzon_merolae       HRDGFVMGEGSGVLVLESLEHARARGAPILCEYLGGAYTNDAFSVTEPVP
18410739_Arabidopsis_thalia          NRDGFVMGEGAGVLLLEELEHAKKRGATIYAEFLGGSFTCDAYHMTEPHP
116055175_Ostreococcus_tauri]        NRDGFVMGEGAGVLVLESLESALARGAPILAEVLGGFATCDAHHMTEPRP
15834771_Chlamydia_murida            DRDGFVLGEGAGVLVLETLDNALKRGAPIFAEVLGTYTTCDAFHITAPRD
29840610_Chlamydophila_caviae        DRDGFVIGEGAGVLVLETLENALKRGAPIYAEILGSYTTCDAFHITAPRD
46446872_Protochlamydia_amoebo       GRDGFVLGEGAGVLVMESLDHALAREAPILAEYLGGGLSCDAYHMTEPRS
116061551_Ostreococcus_tauri]        SRDGFVMGEGAGVLIMESLESARKRGARIHAEYLGGAITCDAHHMTDPRA
15237422_Arabidopsis_thalia          ARDGFVMGEGAGVLVMESLEHAMKRGAPIVAEYLGGAVNCDAHHMTDPRA
108882523_Aedes_aegypti              ARDGFVMGEGSAIFVLEELNHAKARNAEILGEILGYGLSGDASHLTAPRE
8923559_Homo_sapien                  KRDGFVMGEGAAVLVLEEYEHAVQRRARIYAEVLGYGLSGDAGHITAPDP
34802538_Brachyspira_hyodys          GRDGFVIAEGAGILILEEYEHAKKRNANILAEVCGYGFTADANHITAPLE
42525557_Treponema_dentic            KRSGFVMGEGSGILILEEYEHAKKRGAKIYAEFAGYGASSDAYHLTSPDP
95007196_Toxoplasma_gondii           KRAGFVMGEGAGIMVFETEEHAVRRGAPIYGEVAGYGASCDAHHITAPAP
32470845_Rhodopirellula_baltic       DRDGFVLAEGAGLLVFEELEHAKARGAKIYAEVLGYGTTSDAGHITAPDP
85075673_Neurospora                  DRAGFVVSEGAAVLILEELEHAKRRGARILAELTGYGCSGDAHHMTAPRD
67917496_Chlorobium_limico           DRDGFVMGEGAGTLILETLESAKARGAKIYGEIAGVGATADAYHLTAPHP
110637801_Cytophaga_hutchin          DRDGFVMGEGAGALVLEEYEHAKARGAKIYCEVIGGGMSADAYHLTAPHP
89889944_Flavobacteria_bacter        TRDGFVLGEGAGALVLEDYEYAKARGAKIYAEVIGGGFSSDAYHITAPDP
57237499_Campylobacter_jejuni        ERDGFVMGEGAGALVFEEYEEAKKRGATIYAELIGFGESADAHHITSPTL
76259633_Chloroflexus_aurant         LRDGFVPGEGAAVLVLERLSSAQRRGAPILAELIGYGVSADAYHVTAPDP
76258447_Chloroflexus_aurant         ERDGFVMGEGAAVLILEDLEFALARGARILAEVVGYGATSDAYHISNLAE
46198353_Thermus_thermophi           SRDGFVMGEGAGVLVLEAYEHAKKRGARIYAELVGFGRSADAHHITEPHP
94984545_Deinococcus_geothe          SRDGFVLGEGAGVVILEDLEKAQARGATIYAEIVGYGTSADAHHVTLPAP
22325473_Arabidopsis_thalia          DRDGFVIGEGSGVIVLEEYEHAKRRGAKIYAELCGYGMSGDAHHITQPPE
102192140_Rickettsia_canade          DRSGFVMGEGAGVVVLEEYEHAVNRGAKIYGEVIGYGSTGDAYHMTAPHP
83591758_Rhodospirillum_rubrum       GRDGFVMGEGAGIVVLEEYEHAKARGAHIYAEILGYGMSGDAYHITAPAE
106895571_Clostridium_sp.            NRDGFVMGEGAGILLIEELSHALKRDARIYGEIVGYGTTSDAYHITSPSP
53688239_Nostoc_puncti               DRNGFVMGEGAGILFLEELEHALGRGAKIYAEIVGYGLTCDAYHMTAPTP
23124097_Nostoc_puncti               DRNGFILGEGSGILILEELQHALSRGAHIYAEMIGYGMTCDAYHITSPVP
16329903_Synechocystis_sp.           DRDGFVMGEGSGILILEELESALARGAKIYGEMVGYAMTCDAYHITAPVP
67920030_Crocosphaera_watson         DRDGFVMGEGSGILILEELEHALARGATIYGEMVGYAMTCDAYHMTAPVP
116622648_Solibacter_usitat          DRDGFVIGEGGGILILEERESALRRGAPIVAEIVGYGMSSDAYHLSAPLE
116620290_Solibacter_usitat          DRDGFVVGEGAGIIVLEALEHATARGAKILAEIVGYGMSADAFHVTAPPD
47574643_Rubrivivax_gelati           DRDGFVLGEGAGVMVLEEYEHAKARGARIYAELAGFGMGADAFHMTAPNV
82736706_Pseudomonas_putida          GRDGFVLSDGAGALVLEELEHAKARGATIYAELVGFGMSGDAYHMTSPPD
29653839_Coxiella_burnet             GRDGFVLGEGAACVVVEELEHAKKRNATIYAEIIGFGMSGDAYHMTRPDP
                                      * **: .:... ...*  . *  * *       .     *.  .

89298548_Tetrahymena_thermo          TGEGGLRAMQNAINHAQINSNQINLINCHATSTPAGDLAEIVAINKFFCL
```

```
92908594_Mycobacterium_sp.          DGLRAGHAMKRAMETAGIDPKDVNHVNAHATATPIGDTAEANAIRVAGAA
15643565_Thermotoga_mariti          EGRGAAEAMKLALKESSLAVEDIDYVSCHATSTPAGDEAELKAIKKVLVA
71367081_Nocardioides_sp.           EGRGGSRAILRALAEADISPGDIAHVNAHATSTPQGDIAEGLMLHATLVV
66820316_Dictyostelium_discoi       DGNGPLRSMNLALLESGLNSSHIDYINAHATSTPLGDGIECTAVKNFYII
CM2363_Cyanidioschyzon_merolae      EGTEVARCIRLALADASVEPAQVSYVNAHATSTPLGDIAEYRAMSSVLIT
18410739_Arabidopsis_thalia         DGAGVILCIERALASAGISKEQINYINAHATSTHAGDIKEYQALAHCFLK
116055175_Ostreococcus_tauri]       DGVGVNMAIDKALELANVKADDVNYINAHATSTPAGDLAELRVLQKKFAK
15834771_Chlamydia_murida           DGEGITACILGALNKAGIPKERVNYINAHGTSTPLGDLSEVLALKKAFLR
29840610_Chlamydophila_caviae       DGEGITSCILGALESSGIPKERVNYINAHGTSTPLGDISEVLALKKAFLR
46446872_Protochlamydia_amoebo      DGEGVALCIRRALQDAGIQPEEINYINAHATSTPAGDMAEVNALKKVFIK
116061551_Ostreococcus_tauri]       DGLGVSTCIELSIKDAGITVSDINYINAHATSTLVGDVAEVNAIRKVFVK
15237422_Arabidopsis_thalia         DGLGVSSCIERCLEDAGVSPEEVNYINAHATSTLAGDLAEINAIKKVFIK
108882523_Aedes_aegypti             DGTGAMLAMGRALKDANLKPTDIGYINAHATSTPIGDAIEARSIRTLFLA
8923559_Homo_sapien                 EGEGALRCMAAALKDAGVQPEEISYINAHATSTPLGDAAENKAIKHLFLA
34802538_Brachyspira_hyodys         DGAMGARAMSLAIESAKISPDKISYVNTHGTSTPVGDIAEIKAIKKALIK
42525557_Treponema_dentic           SGDGGALAITNALADAGVKPEEVQYYNAHGTSTPINDPAETAMIKKAFMK
95007196_Toxoplasma_gondii          DGSGLSRCLENAIADANIDKRDIGYINAHGTSTPMNDRIETKAFKKVFLL
32470845_Rhodopirellula_baltic      EGLGAAAAMRAAIQDSGRAASEIDYINAHGTSTPLGDKAETRAIHTVLVA
85075673_Neurospora                 DGSGAFLSMKRALKNAGVKPGDVDYINAHATGTAVGDAAEAIAIRSLMIT
67917496_Chlorobium_limico          EGAGAVNAMKAALSIANITPEHIGYINTHGTSTPLGDIAEITAIKKVFLS
110637801_Cytophaga_hutchin         EGLGAKNVMLNAISDAEIAKEDVDYINVHGTSTPLGDIAEVTAIQAVFLN
89889944_Flavobacteria_bacter       EGRGVIAVMKNTLQNAGINPEDVDHINTHGTSTPLGDVAELKAIKAVFIS
57237499_Campylobacter_jejuni       D--GPLRAMKKALNMAG-NP-KVDYINAHGTSTPVNDKNETAAIKELFPL
76259633_Chloroflexus_aurant        DGEGAALAIRRALQQARLAPQQVDYINAHATSTPAGDIAETRAIKQVFVP
76258447_Chloroflexus_aurant        DGEGVARAIRLALQRGALAPEDVDYINAHATSTPAGDPVETTAIKQVFPP
46198353_Thermus_thermophi          EGKGAALAMARALKDAGIAPEQVGYINAHGTSTPAGDRAEVLAIKRVFLM
94984545_Deinococcus_geothe         EGRGAQVAMRMALATAGVNPEQVGYINAHGTSTYFNDLHETQAIKHVFLA
22325473_Arabidopsis_thalia         DGKGAVLAMTRALRQSGLCPNQIDYVNAHATSTPIGDAVEARAIKTVFLA
102192140_Rickettsia_canade         EGRGAYRAMRDAMLDAVITPDMIDYINAHGTSTNLGDAIELNAVQKLFIL
83591758_Rhodospirillum_rubrum      DGNGGFRAMRAALANAKLTVEDIDYVNAHGTSTPLGDEIELGAVKRLFLT
106895571_Clostridium_sp.           NGEGGARAMSKALQDADITYEKIDYINAHGTSTPYNDEFETAAIKDVFLV
53688239_Nostoc_puncti              SGEGVMRAMELALKDGGIFPNEVSYINAHGTSTLANDAIETLAIKKALIP
23124097_Nostoc_puncti              GGLGAARAIELALKDASITPEQISYINAHGTSTPANDSTETSAIKKALVA
16329903_Synechocystis_sp.          DGRGATRAIAWALKDSGLKPEMVSYINAHGTSTPANDVTETRAIKQALIA
67920030_Crocosphaera_watson        DGAGATRAMELALKDGGISPEQVTYINAHGTSTAANDVTETKAIKKALIA
116622648_Solibacter_usitat         DGDGILRVMRNALDDAGLVPEQIEYLNAHATSTPLGDRGEACAIMRLFLA
116620290_Solibacter_usitat         DGDGAFRVMRNAIKDAGLSPEQIDYINAHGTSTDVGDKAETMAIKRAFVA
47574643_Rubrivivax_gelati          D--GPKRAMKAALRSAGIEAGQVQYLNAHGTSTPLGDLNESNAIKLAFLV
82736706_Pseudomonas_putida         SGEGAARCMANALRDAGIQPEDVSYINAHGTSTPAGDVAEVAAIKRVFLA
29653839_Coxiella_burnet            EAEGFTTCMKNSLRDAGIAPERVDYINAHGTSTPAADPLEARAIKKTFLA
                                            :   :  .      :   . *.*.*   *  *   .

89298548_Tetrahymena_thermo         VTANKGAIGHTFGAAGAIESIFTILSIKNGISPLIKNLQNPISHDIKYAL
92908594_Mycobacterium_sp.          VYAPKSALGHSIGAVGALESILTVLALRDGVIPPTLNYETPDYGDYQYAI
15643565_Thermotoga_mariti          INSSKALIGHLLGAAGASELVLAILQMQNSFVHGMPNLDNPIQMEIKNFI
71367081_Nocardioides_sp.           VTSTKSMTGHLLGGAGALEAIATVLALHHRVAPPTINLDNQDPLGDIAAL
66820316_Dictyostelium_discoi       MSSNKGSIGHLLGAAGSVESIFSILSLYNNIIPPTLNLENPSSKPIRNVL
CM2363_Cyanidioschyzon_merolae      MNSTKSLIGHALGAAGALEATATIMAIRTGEVHPTANVEEPESLNIEVAI
18410739_Arabidopsis_thalia         VNSTKSMIGHLLGAAGAVEAVATVQAIRTGWVHPNINLENPDRLDIKAAL
116055175_Ostreococcus_tauri]       INSTKSLIGHLLGAASAVEAVVCIKAIMSGEVHPNINLETPDKLDVKVAL
15834771_Chlamydia_murida           MNSTKSLIGHCLGAAGGVEAVATIQAIQTGKLHPTINIENPIDWDVDVAM
29840610_Chlamydophila_caviae       MNSTKSLIGHCLGAAGGVEAVATIQAIQTGKLHPTINLENPIDWDIDVAM
46446872_Protochlamydia_amoebo      MNATKSMIGHLLGAAGGVEAIATIKAILEHQIHPTINLENPERFIVKRAI
116061551_Ostreococcus_tauri]       MNATKSMIGHCLGAAGGMEAIACIMAIKTGSLHPTLNQQEQEAHKVTAAI
15237422_Arabidopsis_thalia         INATKSMIGHCLGAAGGLEAIATVKAINTGWLHPSINQFNPEQHEVDVAI
108882523_Aedes_aegypti             VSSTKGAHGHLLGAAGNLESLFTVLACREGKLPPTINLENVSEGKRRIAL
8923559_Homo_sapien                 VSSTKGATGHLLGAAGAVEAAFTTLACYYQKLPPTLNLDCSEKTEKRFIL
34802538_Brachyspira_hyodys         INSTKSMTGHALGAAGGIEAIATIMSMIEGKVHPTINVEEQDDYKIEYAI
42525557_Treponema_dentic           VSSTKSMIGHCLGAAGALEAIFCIKAMEEGFYPPTINLTEPDKGEINCAA
95007196_Toxoplasma_gondii          ISSTKSMTGHCLGATGGIEACVSAKVLETGIVPPTINQEETDPTPFEAVL
32470845_Rhodopirellula_baltic      ISSTKSALGHSLGASGGIEAVILCQTIQNNVIAPTINLDTPDEQKVDLAM
85075673_Neurospora                 VSSTKGAIGHLLGAAGAIESVFSILAIAEGAIPPTLNLTSPNQKEVKVAM
67917496_Chlorobium_limico          ISSSKSMTGHLLGAAGAVESIVCLLAMQNSTVPPTINNDNLDPRAIEYAL
110637801_Cytophaga_hutchin         ISSTKSMTGHLLGAAGAIESIATILAIKNQIIPPTINNHNRDKREVNVAM
89889944_Flavobacteria_bacter       INSTKSMTGHLLGAAGAIESIASVMAINEGIIPPTINHTTVDKREVNVAL
```

```
57237499_Campylobacter_jejuni     ISSTKGQTGHCLGAAGAIEAVVSVMALRDGVVPPTINQLVKDKVDLKVVM
76259633_Chloroflexus_aurant      ISSIKSMIGHLTGAAGAVEAAATILALHHGQIPPTINLEHPDQAAIQVAI
76258447_Chloroflexus_aurant      VSASKSQFGHLLGAAGAIEAVVTILAMQNNLLPATINLQQPDPARIEVAL
46198353_Thermus_thermophi        VSSTKSMIGHLLGAAGAVEAIATVQALYHGVIPPTINLEDPDEAKVDYAL
94984545_Deinococcus_geothe       VSSTKSMTGHLLGAAGAVEAIAVAQALKDGILPPTINLTDPDEQQVEYAL
22325473_Arabidopsis_thalia       FSSTKGATGHLLGAAGAVEAIFSILAIHHGVAPMTLNVKNPDKMLVRTAM
102192140_Rickettsia_canade       MSSTKSSIGHLLGAAGSVEFIFSALAIRDQIAPPTLNLEKPMDTKIDYVL
83591758_Rhodospirillum_rubrum    MSSTKSAIGHLLGAAGSVEAIYSILAIRDQVAPPTLNLHDPSERPIRKAL
106895571_Clostridium_sp.         MNSTKSMTGHLLGAAGAVEAIACILSINKSMVHPTINYETPDTLNIDYAL
53688239_Nostoc_puncti            ISSTKSMTGHLLGAAGGIEAVATVKSVALDLIPPTINLEQPDTQRVDVAL
23124097_Nostoc_puncti            ISSTKSMTGHLLGGSGGIEAVATVLAIANDQIPPTINLENPDVQKVEVAI
16329903_Synechocystis_sp.        VSSTKSMTGHLLGGSGGIEAVATVMAIAEDKVPPTINLENPDALIVDVAL
67920030_Crocosphaera_watson      VSSTKSMTGHLLGGSGGIEAVATLMAIANDRIPPTLNLDNPDPLEIDVAL
116622648_Solibacter_usitat       VSSTKSMTGHLLGGSGSLEAGLAVLAIRDQVAPPTINLENRDPLPITYAM
116620290_Solibacter_usitat       VSSTKSMTGHLLGGAGGLEAGITVMAIHDQVAPPTINHECPDKMPIEYAL
47574643_Rubrivivax_gelati        VNSTKSMTGHLLGGAGGIESVFTVLAIRDQVSPPTINIFNQDEMKIEHAV
82736706_Pseudomonas_putida       VSSTKSMTGHLLGAAGAVEAIFSVLAINSQMAPPTINLDEPDSMPIDVVL
29653839_Coxiella_burnet          VSSTKSMTGHMLGAAGALETVISVLAIRDNTAPPTINLENPDEMKIDTVM
                                  . : *.  **  *. .  *                 *

89298548_Tetrahymena_thermo       KNSFGFGGVNVSLLFKKF
92908594_Mycobacterium_sp.        NNSFGFGGHNVALAFGRY
15643565_Thermotoga_mariti        KNSFGFGGHNVSIVVGRY
71367081_Nocardioides_sp.         NNSFGFGGANVAVAFGSV
66820316_Dictyostelium_discoi     KNSFGFGGTNCSLIFSKI
CM2363_Cyanidioschyzon_merolae    STSFGFGGHNSVCVFAPF
18410739_Arabidopsis_thalia       SNSFGFGGHNSSIIFAPY
116055175_Ostreococcus_tauri]     SNSFGFGGHNSAVLFAPY
15834771_Chlamydia_murida         SNSFGFGGHNSTILFSRY
29840610_Chlamydophila_caviae     SNSFGFGGHNSTILFSRY
46446872_Protochlamydia_amoebo    SNSFGFGGHNASVIFAPY
116061551_Ostreococcus_tauri]     SNSFGFGGHNSCVIFAPF
15237422_Arabidopsis_thalia       SNSFGFGGHNSVVAFSAF
108882523_Aedes_aegypti           KNSFGFGGTNAALCFAEY
8923559_Homo_sapien               TNSFGFGGTNATLCIAGL
34802538_Brachyspira_hyodys       SNSFGFGGHNASIVFKRI
42525557_Treponema_dentic         SGSLGFGGHNGVAVFKKI
95007196_Toxoplasma_gondii        SDTLGFGGHNAALLLRRY
32470845_Rhodopirellula_baltic    SNSFGFGGHNACVLFGRF
85075673_Neurospora               SNSFGFGGTNASLVFSKF
67917496_Chlorobium_limico        NNGFGFGGHNATLIFKNG
110637801_Cytophaga_hutchin       CNTFGFGGHNCSIVFRKI
89889944_Flavobacteria_bacter     SNTFGFGGHNCCIAFRKL
57237499_Campylobacter_jejuni     SNSFGFGGTNGCVVFKKV
76259633_Chloroflexus_aurant      SNSFGFGGVNAVLAFRKM
76258447_Chloroflexus_aurant      SNSFGFGGHNVCLAFRKY
46198353_Thermus_thermophi        SNSFAFGGHNAVLVFKRV
94984545_Deinococcus_geothe       SNSFAFGGQNATLLFKRV
22325473_Arabidopsis_thalia       SNSFGFGGTNASLLFASI
102192140_Rickettsia_canade       SNSFGFGGTNASLVIKKI
83591758_Rhodospirillum_rubrum    SNSFGFGGTNASLIFGGA
106895571_Clostridium_sp.         SNSLGFGGHNATIIFKKY
53688239_Nostoc_puncti            SNSFGFGGHNVSLAFKKF
23124097_Nostoc_puncti            SNSFGFGGHNVTLAFKKY
16329903_Synechocystis_sp.        SNSFGFGGHNVTLAFKKY
67920030_Crocosphaera_watson      SNSFGFGGHNVTLVFRKY
116622648_Solibacter_usitat       SNSFGFGGTNGCLIFHSH
116620290_Solibacter_usitat       SNSFGFGGTNGCLIFKRY
47574643_Rubrivivax_gelati        KNNFGFGGTNGTLVFKRV
82736706_Pseudomonas_putida       SNSFGFGGTNGSLVFRRF
29653839_Coxiella_burnet          SNSFGFGGTNGTLVLSRV
                                     :.*** *    .
```

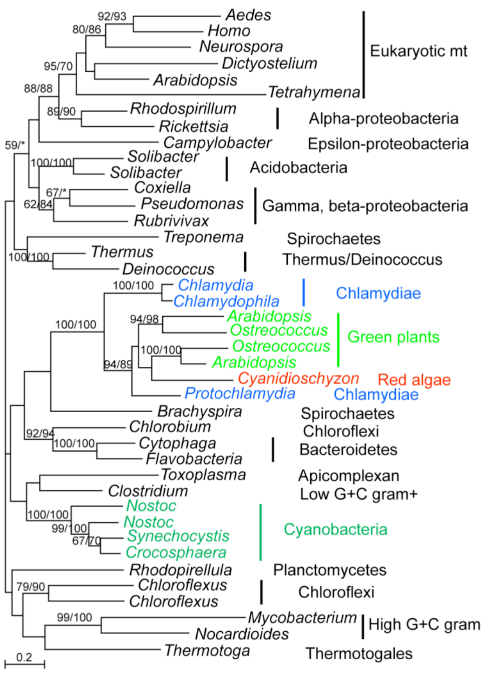

Figure 16. Phylogeny of Beta-ketoacyl-ACP Synthase (*fabF*). Note that sequences of primary photosynthetic eukaryotes form a monophyletic group with those of chlamydiae (in particular *Protochlamydia*) whereas cyanobacterial homologs form another distinct group. Mt=mitochondrial precursor

---

17. Malate dehydrogenase

CLUSTAL X (1.83.1) multiple sequence alignment

```
71069968_Giardia_lambli              VLRVCISGAAGQICYSVLFRIAAGDMLGYDQPVHIVMLEVPAALKAAEGV
62185314_Chlamydophila_abortu        TVSVAVTGGTGQIAYSFLFALAHGDVFGSDCGIDLRIYDLPGLERVLSGV
15605100_Chlamydia_tracho            TVSVAVTGGTGQIAYSFLFSLAHGDVFGLDCGIDLRIYDIPGTERALSGV
67464753_Entamoeba_histol            PLHVLVTGAAGQIGYNLLFLIAHGLMFGPNQTVYLHLYDIM--VEAMEGV
52139822_Lycopersicon_escule         LINVSVSGAAGMIANHFLFKLASGEVFGPDQPVTLKLLGSERSIQALEGV
11182080_Scherffelia_dubia]          TINVVVSGAAGQISNHLLFMLASGSVFGNDQPIALRLLGSERSKEALEGV
116059121_Ostreococcus_tauri]        TITVAVSGAAGQISNHLLFKIASGSVFGADQPVILRLLGSERSRTALEGV
46447406_Protochlamydia_amoeba       PIKIAISGGAGQIAYSLLFRLASGELFGPNQLIELQVLEVPNALSALEGV
89306547_Tetrahymena_thermo          PIKVTVTGAAGNIGYAFVFFAGQGRLFGQDQPIDLTLLELPNSKRPMMGT
1054961_Trichomonas_vigina           PIHILITGAAGQIGYALTFRIAKGDLCG-DRKVVLHLLEIPFGMKALEGC
89296341_Tetrahymena_thermo          EINVCVTGAAGQIGYAFLPLLLTGQCFG-DKKINLRLLDVPQAESILQGV
24528077_Galdieria_sulphu            PINVCVTGAAGQIAYSLLPLIAGGKVFGPQQQVSLRLLEIEAALPSLQGV
15077032_Trypanosoma_brucei          CKRVAVTGAAGQIGYSLLPLIAAGRMLGFDQRVQLQLLDISPALKALEGI
CM3065_Cyanidioschyzon_merolae       PLRVAVTGAAGQIGYQLVTRIASGEMLGKNQPIHLSCIETPQGMKALRGV
95926834_Coxiella_burnet             HVKVAVTGAAGQIGYALLFRLASGQAFGLDTTVDLHLLEIEPALPALKGV
24214839_Leptospira_interr           TVKVAVTGAAGQIGYSLLFRIASGQMFGADTAVEIQMLELEAAIPAAKGV
Acanthamoeba_castella                PVRVTVTGAAGAIGYAMLFRIASGEMLGKHQPVILQLLELEPAMKALEGV
30248777_Nitrosomonas_europa         PIRIAVTGAAGQISYSLLFRIAAGDMLGSSQPVILQLLDIPESGKVLDGV
Seculamonas_ecadoriensis             PVKVAVTGAAGNIGYALLPRIASGEMLGKDQPVILHLLERTEGLNALKGV
71276528_Xylella_fastid              LVRVAVTGAAGQIGYSLLFRIAAGEMFGKDRSVILQMLELPKAQAALKGV
84360329_Burkholderia_dolosa         AKRVAVTGAAGQIAYSLLFRIANGDLLGKDQPVILQLLDLPQAQAAVKGV
15805354_Deinococcus_radiod          PVRVAVTGAAGQIGYSLLFRIAAGDMLGQDQPVILQLLEITPALKALNGV
71365666_Nocardioides_sp.            PLKVAVTGAAGQIGYSLLFRLASGSLLG-DRPIELRLLEITPALKALEGV
15827536_Mycobacterium_leprae        PLKVAVTGAAGQIGYSLLFRLASGSLLGLDRPIELRLLEIEPALKALEGV
29829979_Streptomyces_avermi         PVNVTVTGAAGQIGYALLFRIASGQLLGADVPVKLRLLEITPALKAAEGT
```

```
111224976_Frankia_alni             PVNVTVTGAAGQIGYALLFRIASGQLLGADTPVRLRLLEIPQAVRAAEGT
116058088_Ostreococcus_tauri]      PVNVVITGAAGQIGYALAPMVCAGAATGREKAIALRLLDVPFAEKALRGV
52139818_Lycopersicon_escule       PITVLVTGAAGQIGYALVPMIARGAMLGLDQPVIIHMLDIEPAAESLKGV
24583394_Drosophila_melano         PIRVVVTGAAGQIAYSLLYMIARGEVFGKDQPIVLHLLDIPPMVGVLEGV
5174539_Homo_sapien                PIRVLVTGAAGQIAYSLLYSIGNGSVFGKDQPIILVLLDITPMMGVLDGV
66814308_Dictyostelium_discoi      VINVLITGAAGQIAYSLIFNVCKGDMFGLDQRIKLHLLDIPQMVDSLKGI
Malawimonas_jakobiformis           PTRVCITGAAGQIGYNLVFAVARGDMLGPKQPVILHLLDIPPMMDVLNGT
                                      : ::*.:* *   .      *   *    : :             *

71069968_Giardia_lambli            AMELVDCAFPLLSGFTLTSDNAEAFKDVDYCLLFGAFPRKAGMERAELLS
62185314_Chlamydophila_abortu      RMELDDGAYPLLQSLRVTTSLEDAFDGIDAAFLIGAAPRGPGMERSDLLK
15605100_Chlamydia_tracho          RMELDDGAFPLLQRVQVTTSLHDAFDGIDAAFLIGSVPRGPGMERRDLLK
67464753_Entamoeba_histol          KMELADCCFPLVKGVVASNKTEVAFKDVECAILVAGMPRKVGMERKELIG
52139822_Lycopersicon_escule       AMELEDSLFPLLREVSIGIDPYEVFQDAECSLLIAPKPRGPGMERSGLLD
11182080_Scherffelia_dubia]        AMELEDSLYPLLREVVIGIDPLEVFAEADWALLIGAKPRGPGMERADLLD
116059121_Ostreococcus_tauri]      SMELEDCLFPLLREVDIGIDARTVFAGADWALLIGAKPRGPGMERGDLLE
46447406_Protochlamydia_amoeba     KMEIEDCAFPLLSSIKICSDPYQAFEDIDYALLIGAKSRGPGMERRDLLQ
89306547_Tetrahymena_thermo        VMELDDCAYPLLANIRTTTSIDEAFVGCQVAVLVGAKPRGPGMERKDLLS
1054961_Trichomonas_vigina         VMELQDCAFPNVAGIVWTDKVEEAFKGVDVAFLVGSFPRKDGMDRSDLLA
89296341_Tetrahymena_thermo        ELELQDGAYPLLKSIKTGSNESILFQDVDVAVFIGGFPRKPGMERKDLLT
24528077_Galdieria_sulphu          VMELEDCAFPLLHSVFTTSDAREAFRSCDVAVLLGAFPRKQGMERKDLLE
15077032_Trypanosoma_brucei        RAELMDCSFPLLDGVVITDEPKVAFDKADIAILCGAFPRKPGMERRDLLQ
CM3065_Cyanidioschyzon_merolae     VMELEDCAFPLLKSVSATDQVDKGFADADFAILVGAKPRGPGMERRDLLA
95926834_Coxiella_burnet           VMELEDCAFPLLRNMVVTSDPRVAFNDVNWALLVGAAPRKAGMERKDLLE
24214839_Leptospira_interr         IMELEDCAFPLLQKVTVSSDLDTAFKEINWALLVGSVPRKAGMERGDLLK
Acanthamoeba_castella              IMELKDCAFPLLHGVTASSDVNKAFEGADFAMLVGAKPRTKGMERGDLLN
30248777_Nitrosomonas_europa       LMELQDCAFPLLTDIIVTHDPMIAFDQADIAILVGARPRGKGMERKDLLQ
Seculamonas_ecadoriensis           EMELQDCAFPLLKGIVCTDKAEKAFEDIDFAMLVGAKPRGKGMERADLLR
71276528_Xylella_fastid            MMELEDCAFPLLAGMVVTDNPDIAFKDADAALLVGSRPRGPGMERKDLLM
84360329_Burkholderia_dolosa       VMELDDCAFPLLAGVVITDDPKVAFKDADVALLVGARPRSKGMERKDLLS
15805354_Deinococcus_radiod        VMELRDGAFPLLADVITSDDPMVAFKDADYALLVGAMPRKAGMERGDLLG
71365666_Nocardioides_sp.          VMELDDCAFPNLAGVQIGDDAEQIFDGVNLALLVGARPRGPGMERGDLLS
15827536_Mycobacterium_leprae      VMELDDCAFLLLAGVEIGADPNKVFDGVNLALLVGARPRGPGMERGDLLE
29829979_Streptomyces_avermi       AMELDDCAFPLLQGIDITDDPNVAFDGTNVGLLVGARPRTKGMERGDLLS
111224976_Frankia_alni             ALELEDSAFPLLAGVDVFDDAKRAFEGTNVALLVGARPRTKGMERGDLLS
116058088_Ostreococcus_tauri]      MMELEDAAFELVESVSAHVDPEEAFVDADVCIMVGGFPRKAGMERKDVMG
52139818_Lycopersicon_escule       KMELIDAAFPLLKDVVATTNVVEACKGVNVAVMVGGFPRKEGMERKDVMT
24583394_Drosophila_melano         VMELADCALPLLVEVVPTTDPAVGFKDVSAAFLVGAMPRKEGMERKDLLS
5174539_Homo_sapien                LMELQDCALPLLKDVIATDKEDVAFKDLDVAILVGSMPRREGMERKDLLK
66814308_Dictyostelium_discoi      VMEIQDGAYPLIADTVITADVKEAFTGVHYAILVGAMPRREGMERADLLK
Malawimonas_jakobiformis           VMELHDCAFPMLVGVVATDKVEEAFKDIDVCLMVGAMPRKEGMERKDLLM
                                     *: *     :       .           .: .  .*  **:*  ::

71069968_Giardia_lambli            KNKGIFQIQGAAINEHAKPTCRILVIGNPANTNALVLSTQLTKIPKTNVT
62185314_Chlamydophila_abortu      RNGEIFSLQGSVLNTSAKRDAKIFVVGNPVNTNCWIAMSQAPRLNRRNFH
15605100_Chlamydia_tracho          KNGEIFATQGKALNTTAKRDAKIFVVGNPVNTNCWIAMNHAPRLLRKNFH
67464753_Entamoeba_histol          INTRIMKEQALALKNFANPHVRVLVVANPANTNALVVANNAG-IDVKQIT
52139822_Lycopersicon_escule       INVQIFAEQGKALNAVASRNVKVIEAGNPCNTNALICLKKAPDIHAKNFH
11182080_Scherffelia_dubia]        INGRIFVDQGQALNRVAKKTCKVLVVGNPCNTNALIAMLNAPNIPRKNFH
116059121_Ostreococcus_tauri]      MNGKIFVEQGKALNDVAKPTCKVIVVGNPCNTNALIALSHAPNLDPKNFH
46447406_Protochlamydia_amoeba     ENSKIFVNQGQALNAVAKSSAKIFVVGNPCNTNCLIALNNAPSLKRENFY
89306547_Tetrahymena_thermo        ENGKIFKAQGEAIDRYADRDCKVLVVGNPANTNCLIVQRYAPSIPKQNFT
1054961_Trichomonas_vigina         KNGGIFTVQGKALNDYAKPTVKVLVVGNPANTNCLIAQASAPKLQNKNWC
89296341_Tetrahymena_thermo        INGNIFKKQGQALDTVAKKTCKSLVVANPANTNCLILAETAKSIPKQNFS
24528077_Galdieria_sulphu          KNAGIFKEQGEALNTEAGANVRVIVVGNPANTNAYILKHFAPNIPDKNIT
15077032_Trypanosoma_brucei        TNAKIFSEQGRVLGEVASPNCRVCVVGNPANTNALILLRESKGLNPRFVT
CM3065_Cyanidioschyzon_merolae     SNAQIFAEQGRALNKVASREVRVLVVGNPANTNALVAAANAPDIWAEQIT
95926834_Coxiella_burnet           KNGSIFAGQGKAINENAASDVRIFVVGNPCNTNCLIAMNNAPDIPKDRFY
24214839_Leptospira_interr         INGGIFVNQGKAIEKNAASDVRILVVGNPCNTNCLIAMNNAKGISQDRWF
Acanthamoeba_castella              ENANIFSVQGKALNKVANRDLRVCVVGNPANTNALIASANAPNIHPRRFT
30248777_Nitrosomonas_europa       TNGEIFREQGRALNQVVKRDAKILVVGNPANTNTLITMKNAPDLSPENFS
Seculamonas_ecadoriensis           DNGQIFVGQGKALNAAAKRTAKVIVVGNPANTNCLVAAANAPDMDPRNFS
71276528_Xylella_fastid            ENAKIFTAQGAALNKVARRDVKVLVVGNPANTNAYIAMKSAPDLNPKHFT
84360329_Burkholderia_dolosa       ANAEIFTVQGAALNEVASRDVKVLVVGNPANTNAYIAMKSAPDLPKKNFT
15805354_Deinococcus_radiod        ANGGIFKPQGEALGAVASRNVKVLVVGNPANTNALIAQQNAPDLDPKCFT
```

```
71365666_Nocardioides_sp.          ANGAIFTAQGKALNKVAADDVRIGVTGNPANTNALIAMTNAPDIPQARFS
15827536_Mycobacterium_leprae      ANGAIFTAQGKALNAVAAADIRVGVTGNPANTNALIAMTNAPDIPRERFS
29829979_Streptomyces_avermi       ANGGIFKPQGKAINDNAADDVKILVVGNPANTNALIAQAAAPDVPAERFT
111224976_Frankia_alni             ANGGIFKPQGEAINSGAAEDIRVLVVGNPANTNALIAQTHAPDVPAERFT
116058088_Ostreococcus_tauri]      KNVAIYREQARALATKAKPGVKIVVVANPANTNANILRKFAPEIPAANVT
52139818_Lycopersicon_escule       KNVSIYKAQASALEQHAAPDCKVLVVANPANTNALILKEFAPSFPAKNIT
24583394_Drosophila_melano         ANVKIFRTQGQALDKFAKKDVKVLVVGNPANTNALVCSSYAPSIPRENFS
5174539_Homo_sapien                ANVKIFKSQGAALDKYAKKSVKVIVVGNPANTNCLTASKSAPSIPKENFS
66814308_Dictyostelium_discoi      ANAAIFKVQGKALAEHANKNVKVLVVGNPANTNALIAQVSANGIPKENFT
Malawimonas_jakobiformis           ANAGIFKVQGKALNDFASRDVKVLVVGNPANTNCLLAMQAAPEIPRHNFT
                                    *  *   *. .:   .    :    .** ***          .

71069968_Giardia_lambli            AMSRLDHNRAVGQVAGKLGVRTNRISNVWVAGNHSNTMVPIVDCGDKQPV
62185314_Chlamydophila_abortu      SMLRLDQNRMHTMLAHRAEVPLDEVSHVVIWGNHSAKQVPDFTQAGKPAV
15605100_Chlamydia_tracho          AMLRLDQNRMHSMLSHRAEVPLSAVSQVVVWGNHSAKQVPDFTQADRPIA
67464753_Entamoeba_histol          CLTRLDQNRAIAQIASKLNCKVEDVSDAFVWGNHSEKQCPDISHAPKRVA
52139822_Lycopersicon_escule       ALTRLDENRAKCQLALKAGVFYDKVSNMTIWGNHSTTQVPDFLNAGFPVK
11182080_Scherffelia_dubia]        ALTRLDENRAKCQLALKSGKFYTSVTNVTIWGNHSTTQVPDFVNAGKPAT
116059121_Ostreococcus_tauri]      ALTKLDENRAKCQLAIKAGVFYETVTNVAIWGNHSTTQVPDFVNAGKKAT
46447406_Protochlamydia_amoeba     AMTRLDQNRATFFLSQKSQVSTKDVSCVTIWGNHSATQVPDFVNAQKPVE
89306547_Tetrahymena_thermo        AMTRLDQNRADSILASKIGVHSRQIRNLIIWGNHSATQVPDASQGVTAAR
1054961_Trichomonas_vigina         AMTRLDHNRMVGALAAKFGVTPEKIHKVCIWGNHSNTQVPDTTHATVKVA
89296341_Tetrahymena_thermo        ALTRLDHNRAISQIALKAGCSITDVKNVIIWGNHSTTQYPDVNHGGKRIR
24528077_Galdieria_sulphu          ALTRLDQNRAVSLVARKLGVPLSKVSDVYVWGNHSSTQYPDVLHGGKGKL
15077032_Trypanosoma_brucei        ALTRLDHNRATAQVAERARARVEEVKNCIIWGNHSGTQVPDVNSAGKPAR
CM3065_Cyanidioschyzon_merolae     AMSRLDQNRAITQLALKMHCDVAEVERVIIWGNHSTTQYPDLSHAGEWIR
95926834_Coxiella_burnet           AMTRLDQNRAIGQLALKAGVDVPSVKNMIIWGNHSSTQYPDFYHAGKPAT
24214839_Leptospira_interr         AMTKLDENRAKSQLASKAGVPVKEVTHLGIWGNHSATQYPDFYNTGKPVT
Acanthamoeba_castella              AMTRLDHNRGIAQLADKLKCKVTDIERFAIWGNHSATQYPDISHTGKWAK
30248777_Nitrosomonas_europa       GMLRLDHNRALSQVAMKLNQPVSHIRKMIVWGNHSSTQFPDLSHAHQKVI
Seculamonas_ecadoriensis           AMTRLDHNRALAQLALKTGASVSDIKNFAIWGNHSPTMYPDISVAGKKAS
71276528_Xylella_fastid            AMLRLDHNRALSQLSTKLSKPVANIEKLIVWGNHSPTMYPDYRFAGTPII
84360329_Burkholderia_dolosa       AMLRLDHNRALSQLAAKSGKPVASIEKLAVWGNHSPTMYPDFRFAGESLL
15805354_Deinococcus_radiod        AMVRLDHNRALSQLAEKTGAAVSDIKNVTIWGNHSSTQYPDLSQAGKPAL
71365666_Nocardioides_sp.          ALTRLDHNRAISQLAAKTGAAVTDIKKMTIWGNHSATQYPDVFHAGRNAA
15827536_Mycobacterium_leprae      ALTRLDHNRAIAQLATKTGSAVTDIRKMTIWGNHSATQYPDVFHAGKNAA
29829979_Streptomyces_avermi       AMTRLDHNRALTQLAKKTGSTVADIKRLTIWGNHSATQYPDIFHAGKNAA
111224976_Frankia_alni             AMTRLDHNRAIAQLAKKLGVPSAEIRKITIWGNHSATQYPDIFHAGRSGA
116058088_Ostreococcus_tauri]      CMTRLDHNRALAKLGGKSGRATRDVKNVIIWGNHSSTQYPDVNHAGKTAR
52139818_Lycopersicon_escule       CLTRLDHNRALGQISEKLNVHVGDVKNVAIWGNHSSTQYPDVNHAEKPVR
24583394_Drosophila_melano         AMTRLDQNRATSQIAAKLGVPISAVKNIIIWGNHSSTQYPDAGQAVKSVV
5174539_Homo_sapien                CLTRLDHNRAKAQIALKLGVTANDVKNVIIWGNHSSTQYPDVNHAEVGVY
66814308_Dictyostelium_discoi      CLTRLDQNRAKSQIALKAGVNVKDVHNVIIWGNHSSTQYPDYRCGKTPIS
Malawimonas_jakobiformis           CLTRLDMNRAVSQISQRLGTSVDKVKNVIIWGNHSNTQYPDVNHGSTALR
                                    : :** **    :. :       :    : **** .  *

71069968_Giardia_lambli            KPMLPADWIKGEFVPCVRGRGTAVIEARGHSSSASAANAAIDHVRDWHMG
62185314_Chlamydophila_abortu      EVIGDRDWLENIMLPSIQNRGSAVIEARGKSSAASAARALAEAARSIFLP
15605100_Chlamydia_tracho          ETIADRDWLENIMVPSVQSRGSAVIEARGKSSAASAARALAEAARSIYQP
67464753_Entamoeba_histol          DLV-EESWLES-FNKTVQERGAKVIEMRKASSAASAAKAIVDHFRDWCLG
52139822_Lycopersicon_escule       DVIRDSKWLEEEFTEKIQKRGGVLIQKWGRSSAASTAVSVVDAMRSLVTP
11182080_Scherffelia_dubia]        SVITDTAYLKEKFTPLVQTRGGALIKKWGRSSAASTAVSIADAIKSLVVP
116059121_Ostreococcus_tauri]      DVITDREWLESDFTPAIQTRGGLLIKKWGRSSAASTAVSIADHIRNLVNP
46447406_Protochlamydia_amoeba     TIIPDRQWLEKDFIESVQKRGAAIIQARGKSSAASAASALLDAMRDRILP
89306547_Tetrahymena_thermo        GAVSNDKWLTTEFVETVAKRGAAIIQMREKSSAASAASAACDHIHDWFIG
1054961_Trichomonas_vigina         DKL-PKEYLEGEFAQMIATRGGAVIKMRGASSAASAANAALTCVKDWLYG
89296341_Tetrahymena_thermo        QFINDEAYLNNAFIERVQKRGGEVLAARKNSSVMSAANAVKDHLHDWYFG
24528077_Galdieria_sulphu          SYLLDKSYLEETFIPTIQKRGAAVIAARKMSSAMSAANAVGDHLHDWLLG
15077032_Trypanosoma_brucei        AAVDNDAFFDNEFITIVQERGAEIMKLRGLSSALSAAKAIVDHVHDWMLG
CM3065_Cyanidioschyzon_merolae     PRI-DEDWYRNEFIPRIQKRGAEVIEARGASSAASAANGAIDHMRDLWLG
95926834_Coxiella_burnet           EVIRDKNWLLNDFIPVIQQRGAAVIKARGASSAASAANAALDSVWSLINT
24214839_Leptospira_interr         DVISDHEWLKGDFIKNVQQRGAEIIKARGASSAASAANGVVDTVRGIITP
Acanthamoeba_castella              DLV-DEKWVKDTFIPDVQQRGAAIIAARGSSSAASAANAAIEPMRDWVKG
30248777_Nitrosomonas_europa       DLIKDQTWVENSFIPTVQNRGAVVIEARGLSSAASAANAIIDHMRDWIFG
Seculamonas_ecadoriensis           SVV-DKEWLVKEFIPVIQQRGKAIIDARGLSSAASAASSAIDHIRDWFLG
```

```
71276528_Xylella_fastid             EAINDQAWNANSFIPTVSKRGAAIIEARGLSSAASAANAAIDHMRDWLLG
84360329_Burkholderia_dolosa        KLINDDAWNRDTFIPTVGKRGAAIIEARGLSSAASAANAAIDHVRDWVLG
15805354_Deinococcus_radiod         EQV-DRTWYENDYIPTVAKRGAAIIEARGASSAASAASAAIDHMHDWALG
71365666_Nocardioides_sp.           EVVGDQDWIESTFIPTVAKRGAAIIEARGSSSAASAASATIDAARDWLFG
15827536_Mycobacterium_leprae       EVVGDQAWIEDYFIPTVAKRGAAIIDARGASSAASAASATIDAARDWLLG
29829979_Streptomyces_avermi        EVVNDEKWLAEDFIPTVAKRGAAIIEARGASSAASAANAAIDHVYTWVNG
111224976_Frankia_alni              EAVGDQKWIAEEFIPRVAKRGAEIIEVRGASSAASAASAAIDHVYTWVNG
116058088_Ostreococcus_tauri]       EVIGDDAYLDGEFVDAVRRRGAEIIEARQLSSALSAASSVCDHVYDWLNG
52139818_Lycopersicon_escule        ELVANDQWLNTEFITTVQQRGAAIIKARKLSSALSAASSACDHIHDWVLG
24583394_Drosophila_melano          DAINDNGYLQGSFVETVQKRGAAVIAARKMSSAMSAAKAACDHMHDWWNG
5174539_Homo_sapien                 EALKDDSWLKGEFVTTVQQRGAAVIKARKLSSAMSAAKAICDHVRDIWFG
66814308_Dictyostelium_discoi       TAIKDEKWLQGEFISTVQKRGAAVIAARKLSSAASAAKAITDHMHDWVLG
Malawimonas_jakobiformis            AAVNDDAWLNGEFIKTVQNRGAAVIKARKASSAGSAAKAICDHMHDWLLG
                                      :    :        :  **  ::     **  *:* .

71069968_Giardia_lambli             TKPGAFVSCSLLEEPYGIAPGIFYSMPCSGGKWQIVK-LDLPKDVLDEMK
62185314_Chlamydophila_abortu       K-DGEWFSTGVCSDPYGIPEDLIFGFPCRSGDYEIVPGLLWDTFIKNKIQ
15605100_Chlamydia_tracho           K-EGEWFSSGVCSDPYGLPEDLIFGFPCRTGEYEVIPRLPWDAFIRGKMQ
67464753_Entamoeba_histol           TNKEKIVCMGVYSEQYGIPKGIFFSMPVVGGEYHIVDDLVLSESTRTMLK
52139822_Lycopersicon_escule        TPEGDWFSTGVYTNPYGIAKDIVFSMPCRDGDYELVKDVLMDDYLRSRIK
11182080_Scherffelia_dubia]         TPAGDWFSTAVCTDPYGIQEGLVFSMPCRDGDYEVVPGLVIDDYLREALR
116059121_Ostreococcus_tauri]       TAPGDFFSSAVLSNPYGIPDGLCYSFPCRDGSYEIVQGLEINDWLRERMD
46447406_Protochlamydia_amoeba      TPTGQWFSTALLSDPYGIEEGLIFSFPCRNGELSIVSGLKWDAFLEEKIK
89306547_Tetrahymena_thermo         TQSGQIVSMAVISDSYGVPEGLCFSYPVTNGKWQIVQGLQWDELTKKRIE
1054961_Trichomonas_vigina          TAEGDFVSMAIPPDPYGIKQGTIFSFPVTDGEVHVVEGLELNDWVKGRLE
89296341_Tetrahymena_thermo         TKSGEFVSMAVYSDSYGVPKGLIFSYPVTNFNYKIVQGLNIDEFSKEKIR
24528077_Galdieria_sulphu           SDR--IVSMSVASDSYGIEKGTIFSFPLRGGTYEIVKGLQLDDFSQRYIK
15077032_Trypanosoma_brucei         TPSGTHVSMAVYSDPYGVPGGLIFSFPVTGGEWQIVSGLNVTPAISERIK
CM3065_Cyanidioschyzon_merolae      AGNK-WLSMSVPSDSYGIDACLWYSVPCLGAHFRRILELPVDEFSASMME
95926834_Coxiella_burnet            TPADDNYSVALCAQQYGVDEGLIFSFPCRNGVVSVIEEIEHNEFGQQKLK
24214839_Leptospira_interr          TAPGDCFSAAVVSDSYGAEKGLIFGFPLKGKKVEIIQGISLNDFAKEKFK
Acanthamoeba_castella               T-NGQWTSMGVWTGDYGTSPDIYYSFPVVDGEYTIVQNVPVDKFSAERMQ
30248777_Nitrosomonas_europa        TRDDDWITMGILSDSYKIPKGVIYGFPVVNGGRKIVQGLEISPFSRTRLD
Seculamonas_ecadoriensis            S-NNEWTSMAVISNEYGCDKGLYTSFPVVNGDWEIVKGLTVDEFSRQRID
71276528_Xylella_fastid             S-NGKWITMGVPSDSYGIPEGMIFGFPVTNGEYSIVKDLPIDTFSKTYID
84360329_Burkholderia_dolosa        T-NGKWVTMGIPSDSYGIPEDIIYGVPVTNGEYKRVEGLEIDAFSREKMD
15805354_Deinococcus_radiod         TKDGEWVSMGIPSDSYGIPEGLIYGFPVRDGKYEIVQGLDVSDFSRGKMD
71365666_Nocardioides_sp.           SADADWVSMAVVSDSYGVPEGLISSFPVTDGDWEIVQGLEIDDFSRAKID
15827536_Mycobacterium_leprae       TPEGNWVSMAVVSDSYGVPEGLISSFPVTDGDWTIVRGLGIDDFSRGRID
29829979_Streptomyces_avermi        TADGDWTSMGIPSDSYGVPEGLISSFPVTDGRYEIVQGLEINEFSRARID
111224976_Frankia_alni              TPEGDWTSAAIPSDSYGVPEGLISSFPVTGGKFEIVQGLELDAFSREKID
116058088_Ostreococcus_tauri]       TKEGEWTSMGVVSDSYGVPEGLVYSFPVTGGKWQIVQGLTIDERSRKLMD
52139818_Lycopersicon_escule        TPKGTWVSMGVYSDSYGIPAGLIYSFPVTKGEWSIVQGLKIDEFSRAKMD
24583394_Drosophila_melano          TAPGQFVSMGVFSDSYDSPKDVIFSFPVENKQWKIVSGLTLSDFAKTKLS
5174539_Homo_sapien                 TPEGEFVSMGVISDSYGVPDDLLYSFPVVNKTWKFVEGLPINDFSREKMD
66814308_Dictyostelium_discoi       TAEGEYVSMGVYSDSYGVPEGLIFSFPVKNGKYTIVQGLQMDDLSKNLIN
Malawimonas_jakobiformis            TAPGEYVSMGVVSDSYGIPEGLVYSFPVT---------------------
                                             .:    *        . *

71069968_Giardia_lambli             LSEAELMKEAADALK
62185314_Chlamydophila_abortu       ISLDEISQEKANVSL
15605100_Chlamydia_tracho           ISLDEILQEKASVSL
67464753_Entamoeba_histol           NSEEELLEEKKLEIA
52139822_Lycopersicon_escule        KSEDELLAEKRCVAH
11182080_Scherffelia_dubia]         KTEDELVKEKECVGH
116059121_Ostreococcus_tauri]       KSAEELVNEKGCVGS
46447406_Protochlamydia_amoeba      LTEQELKEEREMVSS
89306547_Tetrahymena_thermo         VTTKELIEERDLALQ
1054961_Trichomonas_vigina          ATEKELIGEKETAWK
89296341_Tetrahymena_thermo         ITTEELLSEKADAFS
24528077_Galdieria_sulphu           ITADELYAEKAEALA
15077032_Trypanosoma_brucei         ATTTELEEERREVST
CM3065_Cyanidioschyzon_merolae      RTRKELIEERDAVRH
95926834_Coxiella_burnet            ETLDELREERDAVEA
24214839_Leptospira_interr          ITHEELVSERNEVKE
```

```
Acanthamoeba_castella                KTNDELVSEKNGVGE
30248777_Nitrosomonas_europa         IAYDELTQELDSIKH
Seculamonas_ecadoriensis             ASLKELKEERDAVAS
71276528_Xylella_fastid              KTLAELEEERASIAH
84360329_Burkholderia_dolosa         ATLAELLEERDGVAH
15805354_Deinococcus_radiod          ATAQELEEERDEVRK
71365666_Nocardioides_sp.            ASTAELADEREAVKE
15827536_Mycobacterium_leprae        KSTAELADERMAVKQ
29829979_Streptomyces_avermi         ASVKELEEEREAVRA
111224976_Frankia_alni               ASVRELAEEREAVRA
116058088_Ostreococcus_tauri]        ESATELTEEFELAEA
52139818_Lycopersicon_escule         ATAKELAEEKSLAYS
24583394_Drosophila_melano           VTGKELQEEKDEALS
5174539_Homo_sapien                  LTAKELTEEKESAFE
66814308_Dictyostelium_discoi        LTTEELVAEKTTALQ
Malawimonas_jakobiformis             ---------------
```

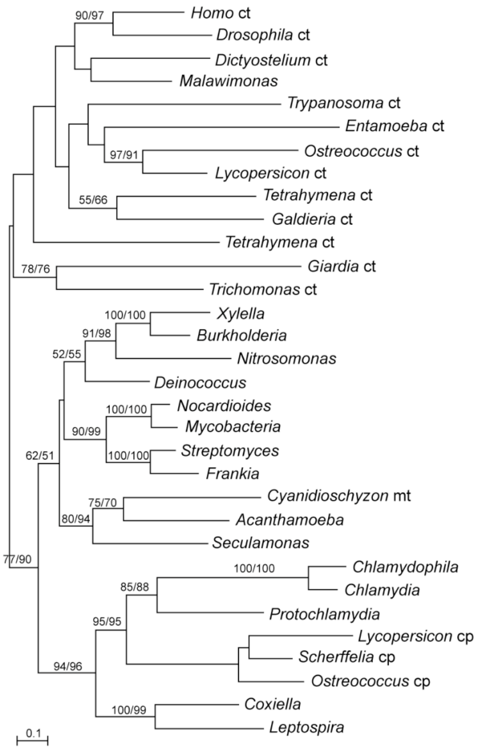

Figure 17. Phylogeny of Malate dehydrogenase, showing that green plant chloroplast precursors form a strongly supported group with chlamydial homologs. Cp = chloroplast, ct=cytoplasmic, mt=mitochondrial, based on the original sequence annotations from GenBank and *Cyanidioschyzon* genome database. The sequences of *Acanthamoeba*, *Malawimonas*, and *Seculamonas* were obtained from the Taxonomically Broad EST Database (TBestDB). No hits to cyanobacterial homologs were statistically significant (Evalue<e-8) in our search of the GenBank non-redundant protein sequence database.

---

18. Sodium:hydrogen antiporter

CLUSTAL X (1.83.1) multiple sequence alignment

```
118166492_Mycobacterium_avium      IAVFVVAYALIASDRVNKTFVALAGAAVVITLPLIWDVIFLLLGMMIIVS
14521887_Pyrococcus_abyssi         LGVFISVYAAIISEKIHRTVAAMLGASIMLFLKIVLDTILLLAGMMVIVN
57640815_Thermococcus_kodaka       LAIFIGVYGLIMSERIHRTVAAMVGASLVLLIKIVLDTILLLAGMMVVVN
108803861_Rubrobacter_xylano       VAVFAVVLALFALELVHRTPAVLGGAALLIIAGAIWETLGLLVGMMILVG
108803863_Rubrobacter_xylano       MSVFAVVLALIGAERVDRTVAALLGAAVIVSLGVVWNTIGLLAGMMVIVA
71279344_Colwellia_psychr          LILVIISFLMIITEHINKAKTTLFFGTLCWILAFIATLWLFLMSAMTFVA
109672731_Campylobacter_concis     LIIFVVGYFFIAAEHIDKAKPAIFIGTFMFLLIGVAQIVFFLMVAMTFIE
77464578_Rhodobacter_sphaer        LVIFVAAYILVVFEHLRKSKPVMLAAGLIWLLIGLGELFLFLLVAITYVN
71280641_Colwellia_psychr          IAIFVLAYTLVILEHLRKSKPVLLAAGLIWILIAAAELFFFLLVAMTYIN
95931201_Desulfuromonas_acetox     LIIFVGAYSLVIMEHMRKSKPVMLAAGVIWVLVAFAELFLFLLAAMTYIN
95929830_Desulfuromonas_acetox     LILFVAAYMLVILEHLRKSKPVLMAAGIIWVLVAIGELFLFLLVAMTYIN
86144878_Vibrio_sp.                LAIFVVAYIVVMMEKLRKSKPVLLAAGLIWIIIGFAELLLFLLVAMTYIS
113947356_Shewanella_baltic        VCVFVLAYVLVMAEHLRKSKPVLVAAGIIWGMIGFAELLLFLLVAMTYIN
16752011_Chlamydophila_pneumo      CALFLFGYLAIVFERVNKSAIALAMGGLMWLVCFSSQVIFFLFSAMAIVE
15834867_Chlamydia_murida          CALFLFGYLAIVFERVNKSAVSLAMGGLMWLVCFSAQVIFFLFAAMAIVE
CM1485_Cyanidioschyzon_merolae     WIVFCTGYAGIILEEFHKAGAALLMAVASWTALALAPLLFFLLGAMSIVE
18402254_Arabidopsis_thalia        ALLFGIGYAGIIFEAFNKSGIGLLMAVSLWVVRSISEIVFFLLGAMTIVE
15222822_Arabidopsis_thalia        ALLFGIGYVLIIFESFSKSGIALLMAVSLWVVRSISQIVFYMLGAMTIVE
86134192_Tenacibaculum_sp.         IIVFVLGYLAITLEKLDKLIPALVMMAVCWAIVALAEILVFLLGAMTIVE
86131526_Cellulophaga_sp.          ILIFIIGYLSITLEKLDKTVPALIMASLIWAFLAVAEILIFLIGAMTIVE
118056239_Pseudomonas_mendoc       ALVFVLGYLCIALEKIDKAASALLTAVVAWTLLVLSEILFFLMGAMTIVE
90423709_Rhodopseudomonas_palu     IAVFVVAYAAIALEGINKSATALLGAGVLWILYSMAEIIFFLMGAMTIVE
90422565_Rhodopseudomonas_palu     AIVFVVAYAAIALEKINKTASALIGAGLLWSVYAVAQVVFFLIGAMTIVE
89900927_Rhodoferax_ferrir         SVIFVLSYLAIALEKISKSASALMGAGWLWTIYALAQIVFFLMGAMTIVE
88936799_Geobacter_uraniu          IVIFIIAYTAIALEKINKSASALIAAGLLWTIYALAQIVFFLMGAMTIVE
71906322_Dechloromonas_aromat      AVVFVIAYAAIALEKVNKAASALLGAGLLWTIYALAQIIFFLMGAMTIVE
                                     :.      .  : . :    :                 :   :  :

118166492_Mycobacterium_avium      VLRQTGVFEYVAIWSILLVLVTALASALLDNVTTVLLIAPVTLLVCDRLA
14521887_Pyrococcus_abyssi         ISRESGLFEYIAIKVLLFSVTTAVVSAFLDNVTTVLLLTPMLLYITRRMG
57640815_Thermococcus_kodaka       VARESGLFEYIAIKTLLFSVVTAVVSAFLDNVTTVLLLTPMLLYITKKMG
108803861_Rubrobacter_xylano       ILKHTGLFTYLAIRSVYLGLITALLSAFLDNVTTVLLLFPVTLVIARILD
108803863_Rubrobacter_xylano       ILDRTGIFEYLAIKSVILALVTAFLSAFLDNVTTVILMVPVTFLIADALG
71279344_Colwellia_psychr          YLNSKGFISALVQRIFVIAIFAFVFSSFADNVTATLVSIAVVMNLQLEVK
109672731_Campylobacter_concis     ALVERDVFNALKYNLWLTGILAFFISPVADNLTTALILSTVLLTIDRNNT
77464578_Rhodobacter_sphaer        TLEERRGFEVLRAKLLLTGALSFFLSGILDNLTTALVMGTVVMALGSHSP
71280641_Colwellia_psychr          ALLERDVFNSLRDWLWITGILAFFISPIADNLTTALIMCAVVLAVGKDEP
95931201_Desulfuromonas_acetox     AMEERNVFQALRSWLWLTGLLAFLISPIADNLTTALLMGAVVMAVGGSNQ
95929830_Desulfuromonas_acetox     AMEERNVFQALRSWLWITGLLAFFISPIADNLTTALLMGAVVMAVGGSNQ
86144878_Vibrio_sp.                AMEERRLFDALQAWMWITGILAFFISPIADNLTTALLMCAVVLKVAGSNP
113947356_Shewanella_baltic        AMEERNLFDAIRGWLWLTGFMAFFISPVADNLTTALLMCAVVMKVGAGQR
16752011_Chlamydophila_pneumo      LIDAHKGFSVIVKFCWALIGLSFFLSAALDNLTSIIIIISILKRLVKARE
15834867_Chlamydia_murida          LIDAHKGFSIVVRCCWVLLTLSFFLSAALDNLTSIIIIVSILKRLVKARE
CM1485_Cyanidioschyzon_merolae     IVDGHRGFRTVTDSIWIISLITFFMSAILDNLTTTIVMVSILRQLVPPSA
18402254_Arabidopsis_thalia        IVDAHQGFKLVTDNIWVVGFVTFFLSSILDNLTSTIVMVSLIRKLVPQSE
15222822_Arabidopsis_thalia        IIDAHQGFKLVTDCIWVIGFATFFLSSVLDNLTSTIVMVSLLRRLIPPSE
86134192_Tenacibaculum_sp.         IIDYFDGFSTIKNYVWMFSVLAFILSAIIDNLTATIVLISILQKIVKTRE
86131526_Cellulophaga_sp.          IIDLHRGFEVLKGAVWIIGILAFILSAIIDNLTATIVLITLLRKLIVNRE
118056239_Pseudomonas_mendoc       LIDAHEGFKVITDRIWLVGLITFFLSAALDNLATTIVMISLLRKLIADKH
90423709_Rhodopseudomonas_palu     VIDAHNGFEIITSQIWLVGLVTFFLSAVLDNLTTTIVMTSLTKKLLDDRD
90422565_Rhodopseudomonas_palu     VIDAHNGFEVITSMIWLIGLVTFFLSAALDNLTTTIVMISLIKKLLGKSD
89900927_Rhodoferax_ferrir         VVDAHEGFLAITSRIWMIGFVSFFLSSILDNLTTTIVMISLVKKLLDKRD
88936799_Geobacter_uraniu          VVDAHNGFNVITSRIWLIGIVTFFLSSVLDNLTTTIVMISLLKKLLDRHE
71906322_Dechloromonas_aromat      VIDAHNGFDVITARIWLVGFVTFFLSAILDNLTTTIVMISLMRKLLDEHE
                                          :  :          : . *   **::: ::  .:   :

118166492_Mycobacterium_avium      IAAAPFLMEVFASNVGGAATLVGDPPNIIIASRGGLSFNDFLVHLAPIVV
14521887_Pyrococcus_abyssi         VDPVPYLLEIFASNIGGTATLIGDPPNIMIASAAKLSFNEFIMNMTPIAF
57640815_Thermococcus_kodaka       VNPIPFLLEVFASNIGGTATLIGDPPNIMIGSAANLSFNEFILNMGPIAL
108803861_Rubrobacter_xylano       QDPLPFLLEVLCSNIGGTATLIGDPPNIIIGTATGLSFNAFIANLVSPVA
108803863_Rubrobacter_xylano       MSPVPFLLQVFASNIGGASTLIGDPPNILIGSAAGLSFMDFVVNMAPVVV
71279344_Colwellia_psychr          KRLKYATLIIFSVNSGGVSLITGDVTTLMIFLENKVTISNLL-ILILPSF
109672731_Campylobacter_concis     NFLVAGAIIVVAANAGGAWSPFGDITTLMVWAAGKSPFLDFF-ALFPASF
```

```
77464578_Rhodobacter_sphaer         RFVTIGCIIVVAANAGGAFTPFGDITTLMVWQKGRLDFFEFF-AILIPSL
71280641_Colwellia_psychr           KFIAMSCIIVVGANAGGAFSPFGDITTLMVWQKGMVQFSEFF-HLFLPSV
95931201_Desulfuromonas_acetox      RFVVMACIVVVGANAGGAFSPFGDITTLMVWQKGMVDFGEFF-ALFLPSL
95929830_Desulfuromonas_acetox      RFVVMACIVVVGANAGGAFSPFGDITTLMVWQKGIVQFGQFF-VLFVPAL
86144878_Vibrio_sp.                 KFVNLACVIVIAANAGGAFSPFGDITTLMVWQAGYVSFSEFI-PLFIPSV
113947356_Shewanella_baltic         KFITLACIIVVAANAGGAFSPFGDITTLMVWQKGLVHFGDFF-NLFIPAL
16752011_Chlamydophila_pneumo       DRLLLGAICVIAVNAGGAWTPLGDVTTTMLWINNKITSWGIIRALFVPSL
15834867_Chlamydia_murida           DRLLLGALCVIGVNAGGAWTPLGDVTTTMLWINDKISTAGIITSLFLPSV
CM1485_Cyanidioschyzon_merolae      RRLVGAAV-VVAANCGGVATPLGDVTTTMLWIGGQITTVATIRDLLVPSF
18402254_Arabidopsis_thalia         YRKLLGGVVVIAANAGGAWTPIGDVTTTMLWIHGQISTLPTMKDLFLPSV
15222822_Arabidopsis_thalia         YRKLLGAVVVIAANAGGAWTPIGDVTTTMLWIHGHISTFSTIKNLFLPSA
86134192_Tenacibaculum_sp.          DRIWFAGLIIIAANAGGAFSPIGDVTTTMLWIGDKVSTGMLFIYLFLPSL
86131526_Cellulophaga_sp.           QRLWFAAMVVIAANAGGAWSPIGDVTTTMLWIADNVTAMGLIKFVIIPSV
118056239_Pseudomonas_mendoc        ERWFYAGIVVIAANSGGAWSPIGDVTTTMLWIGNQITATGIVVKLIVPSL
90423709_Rhodopseudomonas_palu      DRLFFAAMVVIAANAGGAWSPMGDVTTTMLWIGGQLSAVAIVRDVVLASM
90422565_Rhodopseudomonas_palu      DRLFFAAMIVIAANAGGAWSAIGDVTTTMLWIDGQITPLSIMKGLFLPSL
89900927_Rhodoferax_ferrir          DRLFFAGIIVIAANAGGAWTPIGDVTTTMLWIGGQITTLAIMKGLFLASL
88936799_Geobacter_uraniu           DRLFFAGIIVIAANAGGAWSPIGDVTTTMLWIGGQITTLAIMKGVFLASF
71906322_Dechloromonas_aromat       DRLFFAGIIVIAANSGGAWSPIGDVTTTMLWIGGQITSIEIIKGVLLPSL
                                           : :.  * **.    ** .. ::           .  :

118166492_Mycobacterium_avium       IVVGVLIALLPRLFPGAFTVDPERVAIRDPRLLVTCGVVLLAVFAAFHGP
14521887_Pyrococcus_abyssi          LDLFIMVALIYLFYRREFKNVEYHVDIRDKQLFRKSVVTIAFVVLAFHDT
57640815_Thermococcus_kodaka        VDLFATIGIIYLAYRAEMNSHKENEEIRDPSLFRKSITVLIGVVIGFHDK
108803861_Rubrobacter_xylano        ATLLLTLAILWAVYGRRMAAAEEQRKIRDGRLLVRAGLVTAATVGAFQDL
108803863_Rubrobacter_xylano        LSLVPALAFLYFVFRGEFRSDKRAREIRDPVLLRRSLIVLSLVIAAFHGL
71279344_Colwellia_psychr           LGVLTLASLLSMKLSGEVKLAQTRIKAKSDIVIALIFVLTISGTLLANVL
109672731_Campylobacter_concis      IGWFVTAFLLSRIVPNTAPHFDVANEKKGGKAVIFIGAFTIFCAVMMHQL
77464578_Rhodobacter_sphaer         VNWAVPAAILFFAVPKGTPDAQAETIKPGALGVAILFAATIVTAVSFKNF
71280641_Colwellia_psychr           VNFVIPALIMQFFLPTGNPSSETSDVKKGGLIIVGLFILTILTAVSFHNF
95931201_Desulfuromonas_acetox      VNWLVPALIMNFMISKETPQALDEMVKFGAKRVMVLFLCTIVTAVSFHNF
95929830_Desulfuromonas_acetox      VNWLVPAFIMNFMISKEKPEAADEAVKFGAKRIMVLFLMTIVTAVSFHNF
86144878_Vibrio_sp.                 MNYLVPALIMSYFVPTTQPDTVHQHVKRGARRIVFLFIMTIATAVAFHAV
113947356_Shewanella_baltic         VNFALPAAIMSVFIPHFVPEPEKEHVKPGAKRIVGLFLLTIATAVCAHSF
16752011_Chlamydophila_pneumo       VCVLVAGFCGQFFLRKRGSTLIAKDVPPKSLWIIFIGLGSLLMVPVWKAC
15834867_Chlamydia_murida           VCVVVAGVCGQLLLKKRRCSGLLEDLLPKSNLIAFVGFGSLLMVPMWKAV
CM1485_Cyanidioschyzon_merolae      ASLLVSVLVFSPMVAQAAAKDAIDPVAPRGRLIFFSGVASLLFVPIFKAG
18402254_Arabidopsis_thalia         VSLAVPLALMSLTVNGKEQDPKDVLAAPRGKLVFGVGLGALVFVPVFKAL
15222822_Arabidopsis_thalia         ISLVVPLALMSLTVHGMGLNTPPTPLAPRGKLVFGVGFGALLFVPLFKSL
86134192_Tenacibaculum_sp.          LCMVVPSFIASMLFKGDIDFDPNEVDSKYSGRMLLIGLGAIVFVPVFKTV
86131526_Cellulophaga_sp.           ICFVIPFFIATYLFKGDIVVDTQEDELLSSKTMLFLGLGMIVSVPIFKTI
118056239_Pseudomonas_mendoc        ICLLVPLIIMSFRLKGEVIPPRLKESPFERNLVFCLGLGALIFVPIFKTI
90423709_Rhodopseudomonas_palu      VSLLVPLVMVTWRLRGRAFVPKPIDAVFERNLIFFLGLGILVLVPAFKAV
90422565_Rhodopseudomonas_palu      VNLIVPLLVVSYLLRGRDIVLPTRQDQLERNVTFYLGLAILIGVPAFKSM
89900927_Rhodoferax_ferrir          TNLLVPLAVTSFVLGARPVVPPPRRQAFERNLMLFLGLAILVGVPVFKTV
88936799_Geobacter_uraniu           LNMVVPLAVTSYFLRGRQVISPSKNEPFEQNLMFAMGIGILVCVPIFKTV
71906322_Dechloromonas_aromat       VSLLVPLLITAYRLRGRDVVAPARVGVFERNLMFALGLGVLVAVPAFKTL
                                                                                   :

118166492_Mycobacterium_avium       LHLEPSLVALLGAGILVVASRLQPADYLSGVEWDTLLFFAGLFVMVGALV
14521887_Pyrococcus_abyssi          IGIEPAVVALTGASILLLWSRVPPEVALEKVEWATLFFFGGLFIIVGGLV
57640815_Thermococcus_kodaka        LGVEPAVIALTGASILLLWSRASPEHALEKVEWATLFFFGGLFIIVGSLV
108803861_Rubrobacter_xylano        TGLSPAVVALAGAAVAMIVCGTRVERCLEEVEWPTILFFVGLFVMVGALE
108803863_Rubrobacter_xylano        LHLEAATIALFGAAGLMLYARANVEEVLREVEWPTLLFFVGLFVLVGGLE
71279344_Colwellia_psychr           FHIPPVLTFLFGLSIMFLVANQDALNYIREVEYDALLFFLGVLLLVGILK
109672731_Campylobacter_concis      FHLPAMWGMMFGFSLLSLYTPMNVFHYMSKIENNTLFFFFGILAAVGALH
77464578_Rhodobacter_sphaer         LNLPPALGMMLGLGYLQLWSVLDSFHQIQRVEWDTLLFFFGIIFAVGGLG
71280641_Colwellia_psychr           LHLPPVFGMMLGLGYLKFFGEFDIFDKIAKSEWDTLFFFYGVILAVGGLG
95931201_Desulfuromonas_acetox      LHLPPAAGMMLGLGYLGMFSGFDLFRKIARAEWDTLLFFYGVILCVGGLG
95929830_Desulfuromonas_acetox      LDLPPAAGMMLGLSYLGLFSGFDLFRKIARAEWDTLLFFYGVIMCVGGLS
86144878_Vibrio_sp.                 LHFPPVMGMMMGLAYLQFFGPFDVFRRVSHAEWDTLLFFYGVVMCVGGLS
113947356_Shewanella_baltic         LGMPPVLGMMTGLGFLQFFGPFDVFSRISRAEWDTLLFFYGVVLCVGGLG
16752011_Chlamydophila_pneumo       LGLPPFMGALLGLGLVWLTSHLRVPHILTKIDISSITFFIGILLAVNALS
15834867_Chlamydia_murida           LGVPPFIGALLGLGLVWLVSHLRMPHILTRIDISSVTFFIGILLAVNALA
CM1485_Cyanidioschyzon_merolae      TGLPPYMGMLLGLGFMWLLTELRASRALARIDQSSILFFLGILMSVGALE
```

```
18402254_Arabidopsis_thalia         TGLPPYMGILLGLGVLWILTKLKVPQALSRIDTQGALFFLGILLSVSSLE
15222822_Arabidopsis_thalia         TGLPPYMGILLGLGVIWILTHLKLPHALSRIDSQGALFFLGILLSMSSLD
86134192_Tenacibaculum_sp.          THLPPYVGMMLSLGIVATCAHSPVHSSLSKIEMPSILFFLGILMAVAALE
86131526_Cellulophaga_sp.           THLPPYMGMMLALGVVWLVSLYSAHKALSRIEISSILFFLGILMAVAGLE
118056239_Pseudomonas_mendoc        THLPPYMGILFGLSVLWVTTPLSVVGVLRRIDITSVLFFLGILLAVSALS
90423709_Rhodopseudomonas_palu      TGLPPFMGMLFGLGILWLGAHLTLAHALTRIDLSSVVFFLGILLAVATLE
90422565_Rhodopseudomonas_palu      TGLPPFMGILFGLGVLWVVGRPSWVDALMRIDMSSIVFFIGILLAVATLE
89900927_Rhodoferax_ferrir          THLPPFMGILFGLGMLWMVGHLTLVRALTRIDMSSIVFFIGILMAVAVLE
88936799_Geobacter_uraniu           THLPPFMSILFGLGILWLVGHLTLVRALKQIDMSSIVFFIGILLAVATLE
71906322_Dechloromonas_aromat       THLPPFMGILFGLGVLWLVGHLTLVHALSKIDMSSLVFFIGILLSVATLE
                                      . .    : . .             :   :     ** *:.  :  *

118166492_Mycobacterium_avium       KTGVVKHLARLAITATGTATMVVVISGIVDNVPYAATMAPVVAAVLWWSL
14521887_Pyrococcus_abyssi          ETGLIEDVGKWIVSHISAILMIAFLSAIIDNIPFTATMIPLIKYPLWWAL
57640815_Thermococcus_kodaka        ETGLITQVANWMVSHIHAVLIIAFSSAIVDNIPFTATMIPLIKYPLWWAL
108803861_Rubrobacter_xylano        ATGVVGAIADALASASDTAALIGAASAVIDNIPFTATMVPVIEEPLWWSL
108803863_Rubrobacter_xylano        VTGFVGRVAELLTGVSDATALVGFASAVIDNIPFTATMIPVIQEPLWWSL
71279344_Colwellia_psychr           EIGVLNGFTQ-LYQYLPVNYLVGFMSSVIDNVPLTAALLKSGVITEWLML
109672731_Campylobacter_concis      FAGFLNYAVS-LYDKFGVNIGVGFLSAVVDNVPVMSAVLKANPMSQWLLV
77464578_Rhodobacter_sphaer         VLGYLTLASELLYTDLGANIIIGALSAIVDNIPLMFAVLTMDPHGEWLLI
71280641_Colwellia_psychr           FIGYLGMASEFMYGELGANILVGLLSAIVDNIPVMFAVLTMNPHTQWLLV
95931201_Desulfuromonas_acetox      QFGYLAMASQAMYTGLGANVLVGILSAIVDNIPVMFAVLTMEPHGHWLLV
95929830_Desulfuromonas_acetox      QFGYLASASQFMYHDLGANVLVGILSAIVDNIPVMFAVLTMEPHGQWLLV
86144878_Vibrio_sp.                 LLGYLELASGVMYSQWDANVMVGILSAIVDNIPVMFAVLSMEPMGNWLLI
113947356_Shewanella_baltic         FMGYLSLVSEAMYSHWDANVAVGLLSSVIDNIPVMFAVLTMNPLGQWLLV
16752011_Chlamydophila_pneumo       FANLLTDFSLWMDKIFSVAIVIGLLSSVLDNVPLVAATMGMYTDTLWKLI
15834867_Chlamydia_murida           YSHVLQDLSVSMDAVFSAILL-GLVSSVLDNVPLVAATIGMYDDPLWKLI
CM1485_Cyanidioschyzon_merolae      SGGALRMLAQWLDSHLAIPTVIGLASSVIDNVPLVAATMGMYESELWQLI
18402254_Arabidopsis_thalia         AAGILREIANYLDANIPIASAIGVVSAIIDNVPLVAATMGMYDSEFWQLI
15222822_Arabidopsis_thalia         AAGILKVIANYLDAHIAIASIIGVVSAIIDNVPLVAATMGMYDSEFWQLI
86134192_Tenacibaculum_sp.          SLGILFNFADSLKTSVPVVILLGVGSAVIDNVPLVAASLGMFSNPLWHFI
86131526_Cellulophaga_sp.           SLGTLRYVAEILNGAVPVVVILGILSAIIDNVPLVAASMGMYTSLLWHFI
118056239_Pseudomonas_mendoc        SAGHLIQVATYLKDGLGIAISIGLLSAVVDNVPMVAGAMKMYPGNFWEML
90423709_Rhodopseudomonas_palu      HSHILTALAAWLDQTVGIVLILGLVSAIVDNVPLVAASIGMYSSFLWQFI
90422565_Rhodopseudomonas_palu      HTHILAMLAKWLDFNVGIVIVLGLASAVVDNVPLVAASIGMYSSFLWEFI
89900927_Rhodoferax_ferrir          HTHILTALAKSLDELVGIVMVIGLASAVVDNVPLVAASMGMYTSFLWEFV
88936799_Geobacter_uraniu           HSHILTNLARLLDQTVGIVTIIGMISAIVDNVPLVAASMGMYSSFLWEFI
71906322_Dechloromonas_aromat       HTHILTALAQWLDHSVGIVMIIGIASAIVDNVPLVAASMGMYSHFLWEFL
                                        :                    *.::**:*             *  :

118166492_Mycobacterium_avium       ALGTDFGGNLTAIGASANIVLLGIARFTRKGVVVTAVSVALSALYLWLR
14521887_Pyrococcus_abyssi          SLGACLGGNGTAIGASANVVVLGIAYFLKVGMIIMLLTVGAGSLVLIAR
57640815_Thermococcus_kodaka        SLGACLGGNGTAIGASANVVVIGIAHFLKVGMTIMVTTVAIGTAIIWLR
108803861_Rubrobacter_xylano        SLGACLGGNATLIGASANLVVAGMSEFTKVGLPLTALALAVSTAYVLLF
108803863_Rubrobacter_xylano        AIGADFGGNATLIGASANVVVAGMSEFMAYGVPVTLISLAVATPYVALR
71279344_Colwellia_psychr           TYATGVGGSILVIGSAAGIIAMSKVK-LRNAIYLIIAYTVGYGVYFAAN
109672731_Campylobacter_concis      TLTAGIGGSMISFGSAAGVGVMGKLKHMKYAWMVVLGYIVSLVWYVQFE
77464578_Rhodobacter_sphaer         TLTCGVGGSMLAIGSAAGVALMGQAKHLKWTWAIALGYFASIGAHLVIN
71280641_Colwellia_psychr           TLTAGVGGSLLSVGSAAGVALMGQARHLKWTPVIALGYAASIYVHLLIN
95931201_Desulfuromonas_acetox      TLTAGVGGSLLSIGSAAGVALMGTARHLVFTPAIALGYAASIALHMWIN
95929830_Desulfuromonas_acetox      TMTAGVGGSLLSIGSAAGVGLMGTARHLVYTPIVALGYAASIAVHMLIN
86144878_Vibrio_sp.                 TLTAGVGGSLLSIGSAAGVALMGAAHHLKWMPVIMIGYGVSIAAHLWLN
113947356_Shewanella_baltic         TLTAGVGGSLLSIGSAAGVALMGQARHLRWTPVIAFGYVASILVHMWLN
16752011_Chlamydophila_pneumo       AYAAGTGGSILIIGSAAGVAFMGLEKFKRISWIALASYFGGLFSYFVLE
15834867_Chlamydia_murida           AYAAGTGGSILIIGSAAGVAYMGMEKVKHASWIALISYFGGLAVYFLME
CM1485_Cyanidioschyzon_merolae      AYCAGTGGSILIIGSAAGVAYMGMEKLRNASLAALAGYFAGILVYISLH
18402254_Arabidopsis_thalia         AFCAGTGGSMLVIGSAAGVAFMGMEKFRKVSGFAFAGYAAGIAAYLAVH
15222822_Arabidopsis_thalia         SFCAGTGGSMLITGSAAGVIFMSMEKFRKVSGFAFAGFTAGIMTYLAVH
86134192_Tenacibaculum_sp.          AFSAGTGGSMLIIGSAAGVVAMGMEKLKKISWLALVGFLVGSVSFIILR
86131526_Cellulophaga_sp.           AYSAGTGGSMLIIGSAAGVAAMGMEKLKKITWLAFVGFAAGAIAFVLLT
118056239_Pseudomonas_mendoc        AYCAGTGGSCLIIGSAAGVAAMGMEKLKKITGLAFIGYLAGAVTYMLMF
90423709_Rhodopseudomonas_palu      AYCAGTGGSILIIGSAAGVAAMGIEKLRRVSVLALLGYFAGAAVYVMQY
90422565_Rhodopseudomonas_palu      AYCAGTGGSILIIGSAAGVAAMGLERVRRISGLALLGYFAGCAVYIVQY
89900927_Rhodoferax_ferrir          AYCAGTGGSILIIGSAAGVAAMGLEKMRKISGLALIGYFAGAAVYIVQN
88936799_Geobacter_uraniu           AYCAGTGGSILIIGSAAGVAAMGLEKVKKISGLALVGYFAGIATYIVQY
```

```
71906322_Dechloromonas_aromat       AYCAGTGGSILIIGSAAGVAAMGMEKVKKISALALFGYLAGAGVYLLQY
                                    :     **.    *::*.:    .                      .
```

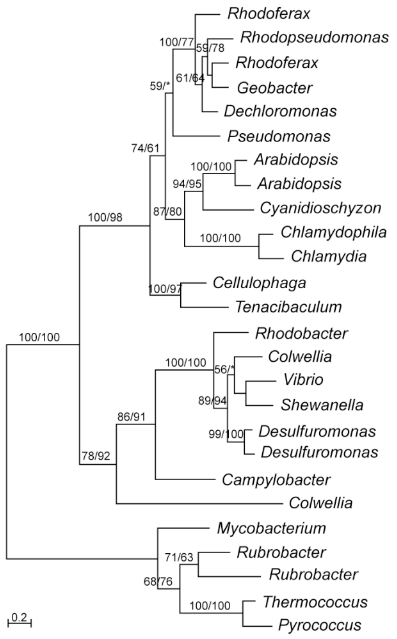

Figure 18. Phylogeny of Sodium:hydrogen antiporter, showing that red algal, green plant and chlamydial sequences form a monophyletic group. The *Arabidopsis* sequences (GI 18402254) are annotated as chloroplast precursors, but no identifiable cyanobacterial homologs were found in GenBank non-redundant protein sequence database using *Caynidioschyzon*, *Arabidopsis* (GI 18402254) and *Chlamydophila* (GI 16752011) as queries.

---

19. 4-hydroxy-3-methylbut-2-en-1-yl diphosphate synthase (*gcpE*)

CLUSTAL X (1.83.1) multiple sequence alignment

```
32471760_Rhodopirellula_baltic      TRPVTIGSITIGDGHPIAVQSMTATKTQNIDATVEQAEALHARGAGVVRI
17986553_Brucella_melite            SVGVSVGGVIVGGSAPVVVQSMTNTDTADVDSTVAQVAALHRAGSEIVRI
15805413_Deinococcus_radiod         TVSVNVGGVMVGSAHPIVVQSMTNTDTANAEATAIQVAQLARAGSEIVRV
116620316_Solibacter_usitat         AVVVDVGGVKVGGTHPIVVQSMTNTDTADVASTVNQVMALARAGSELVRV
42526774_Treponema_dentic           PRTIHIGKLTLGGTSPILLQTMWKESLLDLLSIVKSLNELEQLGCDIVRF
15606681_Aquifex_aeolic             TRQIRVGNVKIGGDAPIVVQSMTSTKTHDVEATLNQIKRLYEAGCEIVRV
15645249_Helicobacter_pylori        TKQIFIGGVAIGGDAPISTQSMTFSKTADIESTKNQIDRLKLAGADLVRV
68234882_Frankia_sp.                SRQIHVGNVPVGGDAPVSIQSMCTTLTSDVNATLQQIAQLTAAGCQIVRV
15643653_Thermotoga_mariti          RKSVKVGKVVIGGEAPVSVQSMTTTKTADVEKTVSQIKSLERAGCEIVRV
73748214_Dehalococcoides_sp.        STEIRLGNLTIGGSAPISVQSMTKTDTRNIPATIAQIKELEECGCEIIRL
51892639_Symbiobacterium_therm      TRAVKVGRVQIGGGAPITVQSMTKCDTRDVPEVLRQIRALEEAGCDIVRV
34763966_Fusobacterium_nuclea       TRVVKVGNLKIGGNNPIIIQSMTNTNSADVEATARQINELEKAGCQLVRM
16079562_Bacillus_subtil            TRPVKVGPLTIGGNNEVVIQSMTTTKTHDVEATVAEINRLAEAGCQIVRV
110803791_Clostridium_perfri        TRKVKIGNIYVGGDFRVSIQSMTNTDTKDVESTVKQIKELQEAGCDIVRC
26987589_Pseudomonas_putida         SRKIWVGNVPVGGDAPIAVQSMTNTDTNDVAATVAQIQRLVDAGVDIVRV
```

```
50122142_Erwinia_caroto            STRIYVGKVPVGDGAPIAVQSMTNTRTTDVEATVNQIKALERVGDIVRV
90579365_Vibrio_angust             SSRIYVGNVPIGDGAPIAVQSMTNTRTTDVEATVAQIKALEKVGADIVRV
46156442_Haemophilus_somnus        STKIYVGNVPIGGDAPIAVQSMTNTRTTDIEATVAQIKALERVGADIVRI
99080703_Silicibacter_sp.          SRQIHVGNVPVGGDAPIAVQTMTNTLTTDIKGTIAQVQAAADAGADIVRV
83592086_Rhodospirillum_rubrum     SRKIRVGSVFVGGDAPVSVQTMTNTLTTDVAATVAQIRRAQEAGADIVRV
56551076_Zymomonas_mobili          SRKIMVGNVAVGGDAPISVQTMTNTPTVDAQATIAQIKRCEAVGVDLIRV
78188327_Chlorobium_chloro         TREVPFGKIALGGYQPIRVESMTNTHTMDTAATVEQCRRLYEAGCEIIRL
78187812_Pelodictyon_luteol        TREVPFGPISLGGYLPIRVESMLNTHTSDTRASVEQCRRLHDAGCEIIRL
53715645_Bacteroides_fragil        TSEVNIGAVPLGGPNPIRIQSMTNTPTQDTEACVAQAKRIVDAGGEYVRL
33861233_Prochlorococcus_marin     TRTVMVGDIAIGSDYPVRVQSMINEDTMDVDNSYLAIKRLHEVGCEIVRL
78184752_Synechococcus_sp.         TRTVMVGSVPIGSEHPIAVQSMINEDTLDIDGSVAGIIRLVDAGCEIVRV
CM2113_Cyanidioschyzon_merolae     TRAVRVGDVYIGGGNPVVVQSMINEDTLDIEGCVASIRRLHEAGCEVVRL
75906658_Anabaena_variab           TRPVKVGNVTIGGGYPVVVQSMINEDTLDIDGSVAAIRRLHEIGCEIVRV
113477916_Trichodesmium_erythr     TRSVKVGDITIGGGHPVVVQSMINEDTLDIERSVAAIRRLHEIGCEIVRV
67925456_Crocosphaera_watson       TRPVKVGNITIGGGFPVVVQSMINEDTLDIEGSVAAIRRLHEIGCEIVRV
Cyanophora_paradoxa                TRSVRVGNIYIGSEHPVVVQSMINEDTLDVYAAADAIRRLHEAGCEIVRV
Glaucocystis_nostochinera          --------------HPVVVQSMINEDTLDVYAAADAIERLHRAGCEIVRV
15618288_Chlamydophila_pneumo      THTVRIGNLYIGSDHSIKTQSMTTTLTTDIDSTVEQIYALAEHNCDIVRV
34540704_Porphyromonas_gingiv      TSVAQIGDTPLGGDFPIRIQSMANVSTMDTQASVDQAARIIDAGADYVRF
110638588_Cytophaga_hutchi         SSVVTIGKVPMGGDYPIRLQSMTTVDTMDTMGSVEQIIRMVEAGCEYVRV
76788770_Chlamydia_tracho          TLPVRIGDLFVGSEHSIKIQSMTTTATTDVDGTVRQICALQEWGCDIVRV
46446374_Candidatus_Protoc         TRIVNVGNVGIGGNHPIRIQSMTTSSTRDVEATIEQVIRLADQGCEIVRV
24215859_Leptospira_interr         TREVKVGDVKVGGNNPIVIQSMINSDTTDTQGSVKQILELERAGCEIVRL
23508024_Plasmodium_falcip         TREVVIGNVKIGGNNKIAIQTMASCDTRNVEECVYQIRKCKDLGADIVRL
30697397_Arabidopsis_thalia        TRTVMVGNVALGSEHPIRIQTMTTSDTKDITGTVDEVMRIADKGADIVRI
61742128_Chlamydomonas_reinha      TRTVMIGKVPVGSQHRIALQTMTTTDTRNVQLTVDQVKKCADAGADIVRI
Bigelowiella_nutans_cp             -------AVEITGD-------------ESEEDLAKLASISPPPVVAILN
116059551_Ostreococcus_tauri]      TRTVHVGNVKIGSEHAIVKQTMTTSDTRDVEKTVAEVIRCADAGADMVRI
Isochrysis_galbana                 -----------------LVVSLTGKETE-----EEVAALKNIQHDFVLL
                                                                               :

32471760_Rhodopirellula_baltic     AVDSDKDAEALAEIRKQANLAVDLQENFRLAEKVAPHVDKIRYNPGHLYH
17986553_Brucella_melite           TVDRDESAAAVPKIRERVPLVGDFHYIGKLLADHPACLAKYRINPGNVGF
15805413_Deinococcus_radiod        TVNTREAAAAVPELVQRVPIVGDFHYNGLLLREYPETLAKYRINPGNVGA
116620316_Solibacter_usitat        TVNTEAAAAAVPKIVDAVPIIGDFHYNGLLLKKYPECLAKYRINPGNSDI
42526774_Treponema_dentic          AVPDMDSAEQFVKLTRLMPLVADIHFDYKLALRCMDGTAKIRINPGNIGS
15606681_Aquifex_aeolic            AVPHKEDVEALEEIVKKMPVIADIHFAPSYAFLSMEKVHGIRINPGNIGK
15645249_Helicobacter_pylori       AVSNEKDALALKELKKVLPLIADIHFHYKFALIAAQSVDAIRINPGNIGS
68234882_Frankia_sp.               AVPSQDDADALPTIARKIPIIADIHFQPKYVFAAIDACAAVRVNPGNIKQ
15643653_Thermotoga_mariti         AVQDEEDAKAIRRIKEQIPLVADIQFDYRLAILSIENADKIRINPGNMS-
73748214_Dehalococcoides_sp.       AIPDMEAASALKSIRPKIPIVADIHFDYRLALASLSAVDGLRLNPGNIGD
51892639_Symbiobacterium_therm     AAPTMEAAECFKEIRKGIPLVADVHFDYRIALKVLEAIDKLRINPGNIGA
34763966_Fusobacterium_nuclea      TINNIKAAEAIKEIKKRLPLVADIHFDYRLALLSIENIDKLRINPGNIGS
16079562_Bacillus_subtil           ACPDERAANAIADIKKRIPLVVDIHFDYKLALKAIEGADKIRINPGNIGR
110803791_Clostridium_perfri       AVLDMDAACAIKDIVAKIPLVADIHFDYKLALKAIENVSAIRINPGNIGS
26987589_Pseudomonas_putida        SVPDMDAAEAFGKIKQRVPLVADIHFDYKIALRVAELVDCLRINPGNIGR
50122142_Erwinia_caroto            SVPTMDAAEAFKLIKQQVPLVADIHFDYRIALKVAEYVDCLRINPGNIGN
90579365_Vibrio_angust             SVPTMDAAEAFKLIKQQIPLVADIHFDYRIALKVAEYVDCLRINPGNIGR
46156442_Haemophilus_somnus        SVPTMDAAEAFKSIKQQIPLVADIHFDYRIALKVAEYVDCLRINPGNIGR
99080703_Silicibacter_sp.          SVPDEASARALKEIVREVPIVADIHFHYKRGIEAAEAAACLRINPGNIGD
83592086_Rhodospirillum_rubrum     SCPDEESTAALKAIISQVPIVADIHFHYKRAIEAAEAAACLRINPGNIGS
56551076_Zymomonas_mobili          SCPDKESTAALKDIVRAVPIIADIHFHYKRALEAADAAACLRINPGNIGS
78188327_Chlorobium_chloro         TVPTEKDAENLRAIRDQTPLVADIHFSVRAAMKAVEFVENIRINPGNYAA
78187812_Pelodictyon_luteol        TVPTEKDAENLRAIRDQTPLVADIHFSARAAMKAVEFVENIRINPGNYAT
53715645_Bacteroides_fragil        TTQGVKEAENLMNINIGVPLVADVHFSPKVADVAAQYAEKVRINPGNYVD
33861233_Prochlorococcus_marin     TVPSLAHAKAVGDIKEKTPLVADVHHNGKIAMEVAKHVDKVRINPGLFVF
78184752_Synechococcus_sp.         TTPSIGHAKAMGKIKAEVPLVADVHHNGKIALEVAQHVDKVRINPGLFVF
CM2113_Cyanidioschyzon_merolae     TVPSMAHAHAVGFIKQRVPLVADVHHNGNIALEVAKHVDKVRINPGLYVF
75906658_Anabaena_variab           TVPSIAHAVALAEIKQKVPIVADVHHNGKIALEVAKHIEKVRINPGLYVF
113477916_Trichodesmium_erythr     TVPSIAHAKSLGEIKQKVPLVADVHHNGKIALEVAKQVDKVRINPGLYVF
67925456_Crocosphaera_watson       TVPSMGHAKALATIKEKVPLVADVHHNGNIALEVAKHVDKVRINPGLYVF
Cyanophora_paradoxa                TTPSLAHAKAVRLIKERVPLVADVHHNGNIALEVAKHVDKVRINPGLYVY
Glaucocystis_nostochinera          TVPSLAHAKAVKLIKERVPLVADVHHNGKIALEVAQHVDKVRINPGLYVF
15618288_Chlamydophila_pneumo      TVQGIKEAQACEKIKERIPLVADIHFFPQAAMLVADFADKVRINPGNYID
34540704_Porphyromonas_gingiv      TAQGVREAVNLQHIHAGIPLVADIHFSPKAAEEALLHVEKVRINPGNFVE
```

```
110638588_Cytophaga_hutchi          TAPSVKEAQNLENIKNEVPLVADIHFTPNAAELAAKIVEKVRINPGNYAD
76788770_Chlamydia_tracho           TVQGLREVHACEHIKDRIPLVADIHFFPQAAIHVVDCVDKVRINPGNYVD
46446374_Candidatus_Protoc          TVQGIKEADACEHIKKGIPLVADIHFYPPAAMRVVDFVDKVRINPGNFVD
24215859_Leptospira_interr          TVPSQADADNLPSIRQEVPLVADIHFTPSVAMKAVEYVEKVRINPGNFAD
23508024_Plasmodium_falcip          TVQGVQEAQASYHIKEKIPLVADIHFNPKIALMAADVFEKIRVNPGNYVD
30697397_Arabidopsis_thalia         TVQGKKEADACFEIKDKIPLVADIHFAPTVALRVAECFDKIRVNPGNFAD
61742128_Chlamydomonas_reinha       TVQGKKEAEACMKIREQVPLVADIHFQPTVAMMVADAFEKIRVNPGNFAD
Bigelowiella_nutans_cp              PSPDASRVHASRRVFDNIPVV---HYFATNGLETKEK-------------
116059551_Ostreococcus_tauri]       TVQGMQEAKACKAIKETTPLIADIHFAPKVAMMVAECFDKIRVNPGNFAD
Isochrysis_galbana                  EVD---------------PSLSRLHASRLFGILTAHG-------------
                                                            :

32471760_Rhodopirellula_baltic      EKVRYLIDTAGSNNCAVRIGVNCGSVDPAKKEKYTPMLESALEHCELVDS
17986553_Brucella_melite            KQFADIVEMAIRYDKPVRIGVNWGSLDQELLTTLEAIVQSALISANLAEE
15805413_Deinococcus_radiod         ENFATMIEVAKEFDKPVRIGVNWGSLDQQVLARLDAMIVSALDSARFAEE
116620316_Solibacter_usitat         DNFRTMIEVAIENRKPVRIGVNWGSLDSALLTRMDAMVASAVQSARAAER
42526774_Treponema_dentic           EKTEEVIRKAKDTGTAIRIGVNSGSLPSDLKKKIDTLTEAAARELEIFEK
15606681_Aquifex_aeolic             EIVREIVEEAKRRGVAVRIGVNSGSLEKDLLEKYEALAESALRWSEKFEK
15645249_Helicobacter_pylori        EKIKAVVDACKEKNIPIRIGVNAGSLEKQFDQKYKGMVESALYNAKLLED
68234882_Frankia_sp.                DKVGEIARAAKAAGTPIRIGVNAGSLDKRLLEKYEALVESALWEASLFEE
15643653_Thermotoga_mariti          DRLKDVVAAAKGKGIPIRVGANVGSIKRRTSERWKDLAESALEEVRLLEK
73748214_Dehalococcoides_sp.        ERVKAVVKSAKEREIPIRIGVNAGSLPKDLPPELQKMVKAAMGHIKILEG
51892639_Symbiobacterium_therm      WKVEEVVRACKDRGVPIRIGVNAGSLEEEFLEKYDGMVESALKHVAILEE
34763966_Fusobacterium_nuclea       ENVKKVVEAAKEKNIPIRIGVNSGSIEKEILEKYDALVESAMYHIRLLEK
16079562_Bacillus_subtil            EKVEAVVKAAKDKGIPIRIGVNAGSLEKRILEKYDGMVESALHHIKILED
110803791_Clostridium_perfri        EKVEAVVKACKEKNIPIRIGVNSGSLSKELLAKYDALVESALEHVKILEE
26987589_Pseudomonas_putida         DRVRAVVDAARDRGIPIRIGVNAGSLEKDLQKKYAALVESALRHVEHLDR
50122142_Erwinia_caroto             ERIRSVVDCARYYNIPIRIGVNGGSLEKDIQEKYEALLESAMRHVDILDR
90579365_Vibrio_angust              DRIRSVVECARDKNIPIRIGVNGGSLEKEIQQKYEALVESAMRHVEILDR
46156442_Haemophilus_somnus         DRIRAVVDCAKDKNIPIRIGVNAGSLEKDLQEKYEALLESALRHVEILDR
99080703_Silicibacter_sp.           KRVAEVIKAARDHNCSIRIGVNAGSLEKHLLEKYDAMVESGLDHIKILQD
83592086_Rhodospirillum_rubrum      ERVREVVRAAKDHGCSMRIGVNAGSLEKELLEKYEAMVESALSHARILED
56551076_Zymomonas_mobili           ERVAEVVRAAKANGCAIRIGVNAGSLEKELLEKYDALVESALNHIKLLQD
78188327_Chlorobium_chloro          QEFLPLVEKARSLGASMRIGTNHGSLSDRVVSRYEGMVEAALEFARMCEE
78187812_Pelodictyon_luteol         QEFLPLVEKARSLGVSMRIGTNHGSLSDRIVSRRDGMVEAALEFARICED
53715645_Bacteroides_fragil         DRFVPFLNICKENHTAVRIGVNHGSLSDRIMSRYEGMVESCMEFLRICVD
33861233_Prochlorococcus_marin      KRFTPLVEVLKSENKALRIGVNHGSLSERMLFTYLGMTESAMEFVKICDE
78184752_Synechococcus_sp.          ETFEPLVKLLREQNKALRIGVNHGSLAERMLFTYKGMVESAMEFVRICDD
CM2113_Cyanidioschyzon_merolae      ETLKPLILSLREQDKAMRIGVNHGSLSERMLFTYEGMVESALECIRVCRE
75906658_Anabaena_variab            ETLAPLVITLRDQGKAMRIGVNHGSLAERMLFTYEGMVESALEFIRICES
113477916_Trichodesmium_erythr      QTLKPLVVSLRDQNKAMRIGVNHGSLSERMLFTYEGMVESALEFIKICES
67925456_Crocosphaera_watson        QTLEPLVISLRDQGKAMRIGVNHGSLAERMLFTYEGMVESALEFIHICES
Cyanophora_paradoxa                 QQLEPLVTSLREQDKALRIGVNHGSLAERMLFTYEGMVESAIEFIKICES
Glaucocystis_nostochinera           ETLEPLVVSLRDQNKALRIGVNHGSLAERMLFTYEGMVESALEFIRICES
15618288_Chlamydophila_pneumo       EKFAPLVEKCKRLGKAMRIGVNHGSLSERIMQKYEGMVASAIEYIAVCEK
34540704_Porphyromonas_gingiv       ERFGSFVEDAKRLGRAIRIGVNHGSLSERMLTRYEGMVQSCMEYLDVCHE
110638588_Cytophaga_hutchi          EKFIPLVKICKEYGTAMRIGTNHGSLSDRILSRYLGMVESALEFLRICED
76788770_Chlamydia_tracho           NKFSPLVEKCKRLGKAMRIGVNHGSLSERVTQRYEGMVYSALEYAEVCVA
46446374_Candidatus_Protoc          EKFTPLVEKCKRLNRAMRIGTNHGSLSDRIMNRYFGMVESALEFARICRK
24215859_Leptospira_interr          EVFSPLVLRCKELGVSMRIGTNHGSLSDRIMNRYQGMVESALEFIRIAES
23508024_Plasmodium_falcip          EKFVPLIEKCKRLNRAIRIGTNHGSLSSRVLSYYLGMVESAFEFSDLCIE
30697397_Arabidopsis_thalia         QVFTPLVEKCKKYGRAMRIGTNHGSLSDRIMSYYRGMVESAFEFARICRK
61742128_Chlamydomonas_reinha       ETFTPLVLKCKELGRAIRIGTNHGSLSARILSYYRGMVESAFEFADVCRK
Bigelowiella_nutans_cp              --------------MVLELGSDAGAL---LVDGY-GAIVGPNWHLE----
116059551_Ostreococcus_tauri]       EVFTPLVLKCKERGVAMRIGTNHGSLSARTLSRYMGMVESAFEFARICRK
Isochrysis_galbana                  -------------------------IDSAVIHHYSDMA------------
                                                             :

32471760_Rhodopirellula_baltic      LGFHNFVVSLKDSDPSKVVQVNTLFAEKDVALHLGVTEAGMPPDGIIKTR
17986553_Brucella_melite            IGLDKIILSAKVSQVQDLIAVYTMLAQRNHALHLGLTEAGMGTKGIVASS
15805413_Deinococcus_radiod         LGLDKILISVKVSSAPELWQVYRQLAPLDYPLHLGLTEAGMGMKGIVASS
116620316_Solibacter_usitat         HGLDQIILSAKVSGVQDLIDVYRALAAVDYPLHLGLTEAGLGSKGIVATT
42526774_Treponema_dentic           ADFKDAVVSMKASNVRETVMANEIFAKKDNPLHLGVTEAGPLIQGIVKST
15606681_Aquifex_aeolic             WGFTNYKVSIKGSDVLQNVRANLIFAERDVPLHIGITEAGMGTKGIIKSS
15645249_Helicobacter_pylori        LDFTNFKISLKASDVIRTIEAYRMLRPLIYPFHLGVTEAGNLFSSSIKSA
```

```
68234882_Frankia_sp.             HDFRDIKISVKHNDPVIMIQAYRLLAQADYPLHLGVTEAGPAFQGTVKSA
15643653_Thermotoga_mariti       EGFYDIIVSVKSSDVLETIKANEYIAEKEYPIHLGVTEAGVSETAIVKSS
73748214_Dehalococcoides_sp.     LDFGLIKVSLKAFDVPTTIEAYTQIASLPYPLHVGITETGTPKTGLVRSA
51892639_Symbiobacterium_therm   LDFHDIVISIKSSRVDQMIEAYRKLSEKDYPLHVGVTEAGTPFAGTIKSA
34763966_Fusobacterium_nuclea    FEFFDIIVSLKSSNVKMMVEAYRKISSLDYPLHLGVTEAGTKFQGTIKSA
16079562_Bacillus_subtil         LDFHDIIVSMKASDVNLAIEAYEKAAKADYPLHLGITESGTLFAGTVKSA
110803791_Clostridium_perfri     LDFHDIVISMKSSNVETMIESYRIASQKNYPLHLGVTEAGTPWRGTIKSA
26987589_Pseudomonas_putida      LDFQDFKVSVKASDVFMAVEAYRLLAKQVQPLHLGITEAGGLRSGTVKSA
50122142_Erwinia_caroto          LNFDQFKVSVKASDVFLAVQSYRLLAARDQPLHLGITEAGGARSGAVKSA
90579365_Vibrio_angust           LNFDQFKVSVKASDVFLAVESYRQLAKQIQPLHLGITEAGGARAGSVKSA
46156442_Haemophilus_somnus      LNFDQFKVSVKASDVFLAVESYRLLAKAKQPLHLGITEAGGARAGAVKSA
99080703_Silicibacter_sp.        HDFHEFKISVKASDVFMSAAAYQMLADADAPIHLGITEAGGLMSGTIKSA
83592086_Rhodospirillum_rubrum   NDFTEFKISVKASDAFLAVAAYKALAEADYPLHLGITEAGGLRGGTVKSA
56551076_Zymomonas_mobili        QDFHEFKVAVKASDVFLAVASYKALAKADCPLHLGITEAGGLIGGTVKSA
78188327_Chlorobium_chloro       VGYYDLLFSMKSSNVRVMIQAYRLLVAKAYPLHLGVTEAGDGDEGRIKSA
78187812_Pelodictyon_luteol      AGYYDILFSMKSSNVRVMIQAYRLLVQRAYPLHLGVTEAGDGDEARVKSA
53715645_Bacteroides_fragil      EKFTDVVISIKASNTVVMVKTVRLLVSVSFPLHLGVTEAGDGEDGRIKSA
33861233_Prochlorococcus_marin   LDFHNIIISMKASRAPVMLAAYRMIADRNYPLHLGVTEAGDGDYGRIKST
78184752_Synechococcus_sp.       LDFHNIVISMKASRAPVMLSAYRLMADTNYPLHLGVTEAGDGDYGRIKST
CM2113_Cyanidioschyzon_merolae   LDFHNLVISMKASKVPVMMAAYRLLAKRDYPIHLGVTEAGDGEYGRIKSS
75906658_Anabaena_variab         LDFRNIVISMKASRVPVMVAAYRLMAKRDYPLHLGVTEAGDGEYGRIKST
113477916_Trichodesmium_erythr   LKYYNLVISLKASRVPVMLAAYRLMVKRPYPLHLGVTEAGDGDYGRVKST
67925456_Crocosphaera_watson     LDFYNLIVSLKASRVPVMLAAYRLMVKRDYPLHLGVTEAGDGEYGRIKST
Cyanophora_paradoxa              LDFYNIIISMKASRVPVMLSAYRLMAKRNYPLHLGVTEAGDGD-------
Glaucocystis_nostochinera        LNFYNIVISLKASRVPVMLAAYRLMVKKEYPLHLGVTEAGDGDYARIKSA
15618288_Chlamydophila_pneumo    LNYRDVVFSMKSSNPKIMVTAYRQLAKDLYPLHLGVTEAGMGVDGIIKSA
34540704_Porphyromonas_gingiv    HEFDDVVISMKASNTLVMTAAVRLLVERHYPLHIGVTEAGDGEDGRIKSA
110638588_Cytophaga_hutchi       LEYYNIVISMKASNTQVMVQAYRLLVQKPYPLHLGVTEAGEAEDGRIKSA
76788770_Chlamydia_tracho        MDYHDVIFSMKSSNPKVMVAAYRSLAYESYPLHLGVTEAGSGTAGIVKSA
46446374_Candidatus_Protoc       NDYHNFLFSMKASNPQVMIQAYRLLTQADYPLHLGVTEAGEGEDGRIKSA
24215859_Leptospira_interr       LGYYDIIVSMKASNPQVMVQAYRMLASRDYPLHLGVTEAGDGNDGRIKSA
23508024_Plasmodium_falcip       NNFYNLVFSMKASNAYVMIQSYRLLVSKMFPIHLGVTEAGFGDNGRIKSY
30697397_Arabidopsis_thalia      LDYHNFVFSMKASNPVIMVQAYRLLVAEDYPLHLGVTEAGEGEDGRMKSA
61742128_Chlamydomonas_reinha    HDYHNFVFSMKASNPLVMVQAYRLLAEEDYPLHLGVTEAGEGEDGRMKSA
Bigelowiella_nutans_cp           ----------------------------EKPLINAPEGEQKFDLSYLKST
116059551_Ostreococcus_tauri]    HDYHNFVFSMKASNPLVMVQAYRLLSHEDYPLHLGVTEAGEGEDGRMKSS
Isochrysis_galbana               ----------------------------ELALQMGTQ-------------
                                                             .:  .

32471760_Rhodopirellula_baltic   IAFEQLIGKGIGDTVRVSLTLPNPEEIDAGRVRSVVKFDDSKLNIISCPS
17986553_Brucella_melite         AAMGILLQQGIGDTIRISLTPEPTREVQVQELLQTMGFRQFVPIVAACPG
15805413_Deinococcus_radiod      VALAPLLTEGIGDTIRVSLTPEPKLEVEVQQILQSLGLRQFLPQVTSCPG
116620316_Solibacter_usitat      AALAPVLQDGIGDTIRTSLTPMPTEEVIVQQILQSLGIRSFTPQVTACPG
42526774_Treponema_dentic        IAFYRLLEQNIGSTIRVSLSDSCENEVIAREILTECGKRQGGIRLISCPR
15606681_Aquifex_aeolic          VGIGILLYMGIGDTVRVSLTDDPVVEVETYEILKSLGLRRRGVEIVACPT
15645249_Helicobacter_pylori     MALGGLLMEGIGDTMRVSITGELENEIKVRAILRHSGRLKEGINWISCPT
68234882_Frankia_sp.             VAFGALLSEGIGDTIRVSLSAPPIEEIKVSAILESLGLRERKLEIVSCPS
15643653_Thermotoga_mariti       IAIGHLLLKNIGDTIRVSISGDPVREVIVKKILIALGLRE-GVEVIACPT
73748214_Dehalococcoides_sp.     VGIGNLLYMGIGDTIRVSLTAPPQEEVFAYEILKSLNLRQRGPILVSCPT
51892639_Symbiobacterium_therm   VGIGTILAEGIGDTIRVSLSTDCVEEVRVYEILKALGLRTRGINIISCPS
34763966_Fusobacterium_nuclea    IGIGTLLVDGIGDTLRVSLTENPVEEIKVKEILKVLDLSDEGVEIISCPT
16079562_Bacillus_subtil         AGLGAILSKGIGNTMRISLSADPVEEVKVRELLKSFGLASNAATLISCPT
110803791_Clostridium_perfri     IGIGTLLAEGIGDTIRVSLTGDPVEEIKVKEILKNFGYVKEGIEFISCPT
26987589_Pseudomonas_putida      VGLGMLLAEGIGDTIRISLAADPVEEVKVYDILKSLHLRSRGINFIACPS
50122142_Erwinia_caroto          IGLGLLLSEGIGDTLRISLAADPVEEVKVFDILKSLRIRARGINFIACPT
90579365_Vibrio_angust           VGLGMLLSEGIGDTLRISLAADPVEEIKVFDILKSLRIRSRGINFIACPT
46156442_Haemophilus_somnus      VGLGMLLAEGIGDTLRVSLAADPVEEIKVFDILKSLRIRSHGINFIACPT
99080703_Silicibacter_sp.        IGLGQLLWMGIGDTLRVSLSADPVEEVKVFEILKSLGLRHRGVNIISCPS
83592086_Rhodospirillum_rubrum   IGIGSMLWAGIGDTIRVSLSAQPEEEVKVFEILKSLNLRHRGVRVVSCPS
56551076_Zymomonas_mobili        LGIGNLLWDGIGDTLRVSLSADPEQEVRVYDILKTLDLRTRGVRVVSCPS
78188327_Chlorobium_chloro       MGIGALLEDGLGDTIRVSLTEDPVNEVPVYNILQGARIRMSKTEFISCPG
78187812_Pelodictyon_luteol      MGIGALLEDGLGDTIRVSLTEDPVNEVPVFNILQGARARMSKTEFISCPG
53715645_Bacteroides_fragil      LGIGALLADGLGDTIRVSLSEEPEAEIPVFGILQAGRIRTSKTEYISCPG
33861233_Prochlorococcus_marin   AGIGTLLAEGLGDTIRVSLTEAPEKEIPVYSILQSLGLRKTMVEYISCPS
78184752_Synechococcus_sp.       AGIATLLAEGLGDTIRVSLTEAPEKEIPVYSILQSLGLRKTMVEYIACPS
```

```
CM2113_Cyanidioschyzon_merolae      IGIGGLLAEGIGDTIRVSLTEDPVKEIPVYGILQALGLRRTMVEYVACPS
75906658_Anabaena_variab            AGIATLLADGIGDTIRVSLTEAPEKEIPVYSILQALGLRKTMVEYVACPS
113477916_Trichodesmium_erythr      AGIGTLLAEGIGDTIRVSLTEAPEKEIPVYSILQALGLRKTMVEYVACPS
67925456_Crocosphaera_watson        AGIGTLLAEGIGDTIRVSLTEAPEKEIPVYSILQALGLRKTMVEYVACPS
Cyanophora_paradoxa                 --------------------------------------------------
Glaucocystis_nostochinera           MGIGSLLADGIGDTIRVSLT------------------------------
15618288_Chlamydophila_pneumo       VGIGTLLAEGLGDTIRCSLTGCPTTEIPVFGTLQNAGVRLVKTEYISCPM
34540704_Porphyromonas_gingiv       VGIGSLLADGIGDTIRVSLSEDPEHEIPVFGILQATRLRMSRTEYISCPS
110638588_Cytophaga_hutchi          VGIGTLLEDGLGDTVRVSLTEAPEFEIPVFGILQAARTRMTKTEYISCPS
76788770_Chlamydia_tracho           VGIGTLLSEGLGDTIRCSLTGSPINEIPIFLILQSANIRSVTVEYVSCPG
46446374_Candidatus_Protoc          MGIGSLLIDGIGDTIRVSLTEDPWHEINPFSILQAARLRMSKTDFISCPS
24215859_Leptospira_interr          IGIGSLLEDGLGDTIRVSLTEDPVLEVPVYDLLQGTRLRLTKTEYISCPS
23508024_Plasmodium_falcip          LGIGSLLYDGIGDTIRISLTEDPWEELTPLNILQDTRIRLFKTDYIACPS
30697397_Arabidopsis_thalia         IGIGTLLQDGLGDTIRVSLTEPPEEEIDPFNLLQGCRMRNTKTEYVSCPS
61742128_Chlamydomonas_reinha       IGIGALLMDGLGDTIRVSLTEDPEFEMDPFGLLQGCRMRNIKTEYVSCPS
Bigelowiella_nutans_cp              -----------------------------FGLLQGSRMRSTKTEFVSCPS
116059551_Ostreococcus_tauri]       IGIGALLLDGLGDTIRVSLTEASELEIEPFGLLQGSRMRNTKTEYVSCPS
Isochrysis_galbana                  --VGGLLCDGLGDGVFIDPTDHSAFPLAAFGLLQGSRMRNTKTEFVSCPS


32471760_Rhodopirellula_baltic      CSRVFIDLAAKVKEMTTFAQEY-SITIAVMGCRVNGPGETDDADLGLWCG
17986553_Brucella_melite            CGRTQEDIRRNMPLWREKYPGVEALSVAVMGCIVNGPGESKHADIGITGE
15805413_Deinococcus_radiod         CGRTQDFIRESMPEWKAKYPGVEDMQVAVMGCVVNGPGESKHANIGITGE
116620316_Solibacter_usitat         CGRTQTYLRVQMPSWKATHPGVETMKVAVMGCVVNGPGESKHSNIGITFE
42526774_Treponema_dentic           CGRKGFDVQAFVKRWQTKLLSEKDISIAVMGCVVNGPGEGKHADLGITGA
15606681_Aquifex_aeolic             CGRIEVDLPKVVKEVQEKLSGVKPLKVAVMGCVVNAIGEAREADIGLACG
15645249_Helicobacter_pylori        CGRIEANLVDMAIKVEKRLSHIKPLDISVMGCVVNALGEAKHADMAIAFG
68234882_Frankia_sp.                CGRAQVDVYTLANEVSAGLEGMEPLRVAVMGCVVNGPGEAREADLGVASG
15643653_Thermotoga_mariti          CGRAEIDVENMAKMIEENFFHVQRLKIAVMGCVVNGIGEGKDADLGVAGL
73748214_Dehalococcoides_sp.        CSRTEVDIVGIASRVQEALNKIDPIRVAVMGCAVNGPGESKEADLGIACG
51892639_Symbiobacterium_therm      CGRVQIDLVKVANEVERRLQHIDPLNVAVMGCVVNGPGEAAEADVALFGG
34763966_Fusobacterium_nuclea       CGRTEIDLIGLAKQVEEEFETEKKFKIAVMGCVVNGPGEAREADYGIAAG
16079562_Bacillus_subtil            CGRIEIDLISIANEVEEYISKIKPIKVAVLGCAVNGPGEAREADIGIAGA
110803791_Clostridium_perfri        CGRTQIDLINIAKEVEERLSSCKNIKVAVMGCVVNGPGEAREADIGIAGG
26987589_Pseudomonas_putida         CSRQNFDVVKTMNELEGRLEDLLPLDVAVIGCVVNGPGEAKEAHVGLTGG
50122142_Erwinia_caroto             CSRQEFDVIGTVNALEQRLEDLIPMDVSIIGCVVNGPGEALVSTIGVTGG
90579365_Vibrio_angust              CSRQEFDVIGTVNELEQRLEDIVPMDVSVIGCVVNGPGEAEVSHLGIAGG
46156442_Haemophilus_somnus         CSRQEFDVIGTVNALEQRLEDIVPMDVSIIGCVVNGPGEALISDLGVTGG
99080703_Silicibacter_sp.           CARQGFDVIKTVETLEERLEHIKPMSLSIIGCVVNGPGEALMTDVGFTGG
83592086_Rhodospirillum_rubrum      CARQGFDVIKTVEVLESRLSHIQPITLSIIGCVVNGPGEARETDIGLTGG
56551076_Zymomonas_mobili           CARQGFDVVKTVKALEERLAHIAPISLSILGCVVNGPGEARETDIGVTGG
78188327_Chlorobium_chloro          CGRTYFDLEKATAAIKLRMGHLKGLKIGIMGCIVNGPGEMADADFGYVGA
78187812_Pelodictyon_luteol         CGRTYFDLERAAASIKTRLSHLKGLKIGIMGCVVNGPGEMADADFGYVGA
53715645_Bacteroides_fragil         CGRTLFNLQSTIARVKEATSHLKGLKIGIMGCIVNGPGEMADADYGYVGA
33861233_Prochlorococcus_marin      CGRTLFNLEEVVDKVRKATSHLTGLDIAIMGCIVNGPGEMADADYGYVGK
78184752_Synechococcus_sp.          CGRTLFNLEDVLHKVRNATSHLKGLDIAVMGCIVNGPGEMADADYGYVGK
CM2113_Cyanidioschyzon_merolae      CGRTLFNIEEVLHKVRDATKHMPGLSIAVMGCIVNGPGEMADADYGYVGK
75906658_Anabaena_variab            CGRTLFNLEEVLHKVRESTKHLTGLDIAVMGCIVNGPGEMADADYGYVGK
113477916_Trichodesmium_erythr      CGRTLFNLENVLHEVREATKHLTGLDIAVMGCIVNGPGEMADADYGYVGK
67925456_Crocosphaera_watson        CGRTLFNLEEVLHQVREATKHLTGLDIAVMGCIVNGPGEMADADYGYVGK
Cyanophora_paradoxa                 --------------------------------------------------
Glaucocystis_nostochinera           --------------------------------------------------
15618288_Chlamydophila_pneumo       CGRTLFDLEEVTTRIRKRTQHLPGLKIAIMGCIVNGPGEMADADFGFVGS
34540704_Porphyromonas_gingiv       CGRTLYNLQETVARIKAATAHLKELKIGIMGCIVNGPGEMADADYGYVGA
110638588_Cytophaga_hutchi          CGRTLFDLQETTAMIRKRTDHLKGVKIGIMGCIVNGPGEMADADYGYVGV
76788770_Chlamydia_tracho           CGRTLFDLLAVSQRIRERTQHLPGLKIAVMGCIVNGPGEMADADFGYVGS
46446374_Candidatus_Protoc          CGRTLFNLQDVTKRIQSRTSHLPGVKIAIMGCIVNGPGEMADADFGYVGS
24215859_Leptospira_interr          CGRTLFDLQETTARIKSRTGHLKGVKIAVMGCIVNGPGEMADADFGYVGA
23508024_Plasmodium_falcip          CGRTLFNIQETTKKIMKLTGHLKGVKIAVMGCIVNGIGEMADAHFGYVGS
30697397_Arabidopsis_thalia         CGRTLFDLQEISAEIREKTSHLPGVSIAIMGCIVNGPGEMADADFGYVGG
61742128_Chlamydomonas_reinha       CGRTLFNLQEVTDQIRTRTGHLPGVAIAVMGCIVNGPGEMADADFGYVGG
Bigelowiella_nutans_cp              CGRTLFDLQETTQAIAERTGHLPGVAIAVMGCIVNGPGEMADADFGYVGG
116059551_Ostreococcus_tauri]       CGRTLFDLQEVTAQIQEKTGHLPGVAIAVMGCIVNGPGEMADADFGYVGG
Isochrysis_galbana                  CGRTLFDLQEVTAQISERTGHLPGVAIAIMGCIVNGPGEMADANF-----
```

```
32471760_Rhodopirellula_baltic      VNLKRGPEALFGYDEILPKLKQELDDL
17986553_Brucella_melite            APVFVDGKKVLRGPGIAEDFQKMVADY
15805413_Deinococcus_radiod         APVYQDGKLLLKGPRIAEEFQELLEKY
116620316_Solibacter_usitat         APVYVDGRLMLKGDRIVAEFLEILNEY
42526774_Treponema_dentic           VIIFKHGAITDLKKLTEEEKIKAVDKA
15606681_Aquifex_aeolic             AWLFKHGKPIVDESEMVDELLKEIQNM
15645249_Helicobacter_pylori        GLIIKEGKVILAEKDLFETFVIEVENL
68234882_Frankia_sp.                GQIFVRGKVIVPEAQIVETLIEEAMRL
15643653_Thermotoga_mariti          VIFVKG--EIVSKEFVLERLKYYLNEL
73748214_Dehalococcoides_sp.        GLLFRKGEKIVPEDELVDALLREIASL
51892639_Symbiobacterium_therm      GMLYVGGERVTTEEEMVEALVELVEQK
34763966_Fusobacterium_nuclea       GILFKKGEIVVSENNLLEELKKMISED
16079562_Bacillus_subtil            GLLFRKGKIVVPEETMVEELKKEIDIL
110803791_Clostridium_perfri        GLIFRKGEIIVKEEDLVEELIKIIETI
26987589_Pseudomonas_putida         NLIYIDGKPALTNDNLVDELEKLIRQK
50122142_Erwinia_caroto             SGFYEDGVRQFDNEQMIDQLEAKIRAK
90579365_Vibrio_angust              SAFYEDGIRQFDNDNVIDQLESKIRAK
46156442_Haemophilus_somnus         SGYYLDGKRQFDNEDLINQLEAKIRAK
99080703_Silicibacter_sp.           GMVYLAGKASMSNDQMIDHIVEEVEKK
83592086_Rhodospirillum_rubrum      NKVYVSGIPDINNEAMVDHLVALVEKK
56551076_Zymomonas_mobili           HMVFLSGVTDVEDAKMLDHIVSLVEAK
78188327_Chlorobium_chloro          ISLYVGKECVLPEDEAVDKLTMLIKAH
78187812_Pelodictyon_luteol         ISLYVGKECVLPEVEAVDRLIDLIREH
53715645_Bacteroides_fragil         ISLYKQKECIIPEEEAVEKLIELIKAN
33861233_Prochlorococcus_marin      IALYRRKEEIVPEDEGVDALIRLIKDD
78184752_Synechococcus_sp.          IALYRGREEIVPEKEGVQALIQLIQED
CM2113_Cyanidioschyzon_merolae      ISLYRGREEIVPENQGVAELIKLMKED
75906658_Anabaena_variab            ISLYRGREEIVPEDKGVEELINLIKAD
113477916_Trichodesmium_erythr      ISLYRGREEIVHESQGVEELINLIKAD
67925456_Crocosphaera_watson        IALYRGREEIVHEEQGVEELINLIKAD
Cyanophora_paradoxa                 ---------------------------
Glaucocystis_nostochinera           ---------------------------
15618288_Chlamydophila_pneumo       IDLYVKHTCVIPMEDAEEELIRLLQEH
34540704_Porphyromonas_gingiv       IDLYKQKICVIPQEQAVQQLIELIKEN
110638588_Cytophaga_hutchi          IALYRGQTVVVHSDKAVDELINIIKED
76788770_Chlamydia_tracho           IDLYVKHKCVIPIENAEEELVQLLKEH
46446374_Candidatus_Protoc          IDLYVGKECVIDFADADDRLVNLIRAH
24215859_Leptospira_interr          VHLYRGKEIVVPSEIADEKLVQLIKDN
23508024_Plasmodium_falcip          IDLYYGKELVIPEEEACDKLIELIKKH
30697397_Arabidopsis_thalia         IDLYVGKTVVIAMTEATDALIGLIKEH
61742128_Chlamydomonas_reinha       IDLYVGKEVVIPMESACDQLIELIKEH
Bigelowiella_nutans_cp              VDLYVGKEMVIPNAEACDALIELIKEH
116059551_Ostreococcus_tauri]       IDLYVGKEVVIASEVACDELIELIKEH
Isochrysis_galbana                  ---------------------------
```

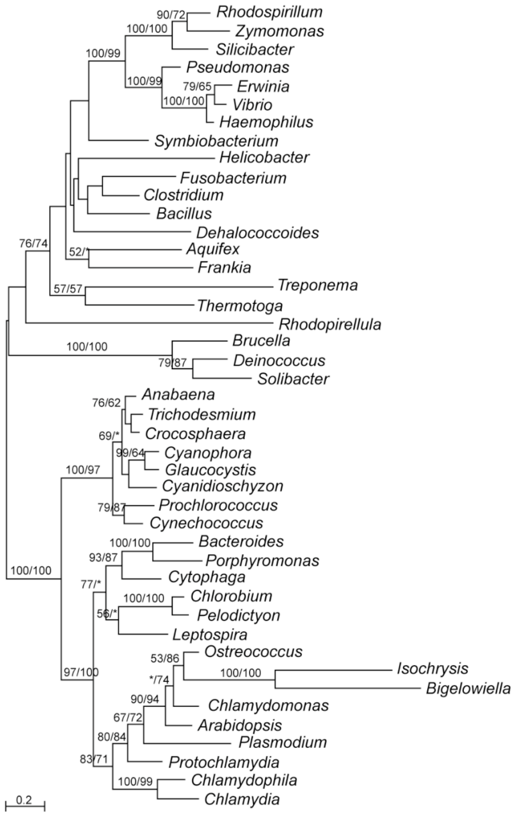

Figure 19. Phylogeny of 4-hydroxy-3-methylbut-2-en-1-yl diphosphate synthase (*gcpE*). Note that sequences from green plants, apicomplexans, chlorarachniophytes, and hyptophytes from a strongly supported monophyletic group with homologs from chlamydial (in particularly *Protochlamydia*) whereas those from cyanobacteria, red algae and glaucophytes form another distinct group (see text for more discussions). *Cyanophora*, *Glaucocystis*, *Bigelowiella*, and *Isochrysis* were obtained from the Taxonomically Broad EST Database (TBestDB). This gene is part of the DXP pathway that is mainly limited to bacterial and plastids of photosynthetic eukaryotes.

---

20. Sugar phosphate isomerase

CLUSTAL X (1.83.1) multiple sequence alignment

```
85089259_Neurospora_crassa          LLNTASTALSCITDLYFHRAVDAIVNGKLVIIGVGKSGHIAKKLVATFNS
53711463_Bacteroides_fragil         LLQKEAQAVLNIPVTDYEKAVELIVKGKLVTSGMGKAGQIAMNIATTFCS
15618437_Chlamydophila_pneumo       ILGKQKEAVDFFFQAFAMQLAEKILSGWVFFSGVGKSGCVARKLVATLQS
15605124_Chlamydia_tracho           IFHKQKQVISQYFASFVHQLTEKLLQGSLFFSGIGKSGCIARKLVATMQS
113476650_Trichodesmium_erythr      VIEQEISAISKLCESIWLKAVLRLRKGKLVVSGIGKSGSISQKIAASFTS
32476096_Rhodopirellula_baltic      QIRLVRETMLREAEAIAAEAAAWISEGSIVLTGVGKAGLIAQKLVATLAS
102191805_Rickettsia_canade         VISSEASALEKLSENIFNTIIEFLLTGRVIFTGIGKSGYIARKIAASFSS
90573271_Clostridium_diffic         VMTTEMNAIRCLIDEAYESIVSEIAEGKVIFMGVGKSAHIGKKLAATFAS
87123156_Synechococcus_sp.          CLQEEAAAIAAAAERLVEGALALLEKAKLVITGVGKSGIVARKIAATFSS
33864177_Prochlorococcus_marin      CLQEEAAAIAVAAERLVEKALVLLERAKLVITGVGKSGIVARKIAATFSS
46447416_Candidatus_Protoc          ILDKQHLYTNHYFETLIEKLVELLLEKSIFFTGVGKSGLVAKKIALTMVS
Prototheca_wickerhamik              LFQEQQEYLNHFFTNLVEIFVKTCLTGTIVCTGVGKSGFIAQKICQTLVS
115444351_Oryza_sativa              LFGAQRRHLDHFFDRLAAAFAQALVPGAVFFTGVGKSGIVARKLAQTLAS
```

```
15232565_Arabidopsis_thalia                LFKSQQDLLNHFFKHLTLDFSRILLTGTVFFTGVGKSAFVANKVSQTLVS
116185416_Vibrio_sp.                       TFKHQANALNEMAKSIYQKAISYIIQGHVIVCGMGKSGHVGKKISATLAS
116621618_Solibacter_usitat                AMRIEAESIARAAERLLVRAVELILPGKVVVTGIGKSGHIARKIVATLCS
110636722_Cytophaga_hutchi                 VILNESEAIKNLVNHIFQHIIDAILKGRVVITGIGKSAIIGNKIVATLNS
34762344_Fusobacterium_nuclea              IYDTEIKSLEMRMNKLFVKVVRKIYKGKVVVTGIGKTGIIGKKISATFAS
67918831_Chlorobium_limico                 ILEQEAGALRQIAERLFASAVTAMHSGKIIISGMGKSGIIAQKIAATMAS
116329828_Leptospira_borgpe                AIDTEIESILHFRKNLIKQAIELILKGKLIVTGVGKSGDVGKKISSTLSS
15606685_Aquifex_aeolic                    VIREEIKGLERLLSSLFSKAVEILREGKVILTGIGKSGHIARKISSTLSS
109673529_Campylobacter_curvus             VLKIEAQELLRHASNLIEEAVNLMYKGKVIVTGVGKSGHIGAKIAATLAS
Rhizopus_oryzae                            ------EAIYLMFKAL---------GGKIVVTGVGKSGKIGEKIVATMLS
15964325_Sinorhizobium_melilo              TLTTATNGIKALADHLLVDAVELMGDGRVVVSGVGKSGHIGRKIAATLAS
16264297_Sinorhizobium_melilo              TIATAADGINALAGCLFVDAIELVARGRVVVAGVGKSGHIGRKIAATLAS
88940199_Acidiphilium_cryptu               VLATEAAALNALAATLFTAAVTTLAAGRVVVTGMGKSGHVARKIAATLAS
83372399_Rhodobacter_sphaer                VIEAETTALTMLGASLFGAAVETILRGRVIVSGMGKSGHIGRKITATLAS
110635071_Mesorhizobium_sp.                TLSTEQAALAALAEALFAEAVEMIASGRVIVTGVGKSGHIGTKIAATLAS
116747458_Syntrophobacter_fuma             VLRIESEGILHLLDHIFARAVLWIYGGRIIVTGIGKSGIVGRKIVATLSS
39996991_Geobacter_sulfur                  VIRIEAEALTRLADTIFEKAVRLILRGRVVVTGMGKSGLIGQKIASTMAS
47573351_Rubrivivax_gelati                 ALAVEAQALGALQQRIFARAVAAMLRGRVVVMGMGKSGHVGRKIAATLAS
94415571_Pseudomonas_aerugi                TIGLERDAVDSLLARIFVRACELLLKGRVVVVGMGKSGHIGKKIAATLAS
116185625_Vibrio_sp.                       VLDIEVAALQELDTYFFEKACELILSGKVVVMGIGKSGHIGNKIAATLAS
68544435_Shewanella_baltic                 VIDIEKSALDNLYQYVFAEACELILTGKVIVMGMGKSGHIGNKISATLAS
                                                                             :.   *:**:.  :.  ::   :: *

85089259_Neurospora_crassa                 LAIQAVFLHPTEALHGDLGQISRNDTLMLITFSGKTPELLLLLPHLDQ-S
53711463_Bacteroides_fragil                TGIPSVFLHPSEAQHGDLGILQENDLLLLISNSGKTREIVELTQLAHNLG
15618437_Chlamydophila_pneumo              LSERALFFSPVDLLHGDLGLVSPGDIVCLFSKSGETQELLDTVPHLKSRR
15605124_Chlamydia_tracho                  FGEKAFFLS-GDLLHGDLGVVSPGDIVCLFSNSGETREILEWIPHLKNRP
113476650_Trichodesmium_erythr             TGIPAIFIHPTEASHGDLGLLDSSDILLVLSASGQTSELLDIMQYASRLK
32476096_Rhodopirellula_baltic             TGSPAHFLHPIEAVHGDLGRVQSKDLVIAFSNSGRSEEVVRVVEYLKHQA
102191805_Rickettsia_canade                TGMPAFYLHPAEASHGDLGMVTRDDLVIMISNSGETKELFNIIEYCNNSS
90573271_Clostridium_diffic                TGTPSFFVHATEAVHGDLGMIESKDITILISNSGNSMEVVNCIKYIKAIG
87123156_Synechococcus_sp.                 IGLMALYLNPLDALHGDLGVVAPDDVCLLLSNSGETSELLEVLPHLKRRG
33864177_Prochlorococcus_marin             IGLMALYLNPLDAMHGDLGVVAQEDVCLLLSNSGETAELLEVLPHLKRRG
46447416_Candidatus_Protoc                 TGTKALYLSPTDAVHGDIGIVSQDDIFIMLSKSGESDELLNLVPPIRNKG
Prototheca_wickerhamik                     TGTKALYLSPTDALHGDIGILTPHDLLVCFSKSGGTDELLRLVPYAKAKG
115444351_Oryza_sativa                     LGFTRAFLSPVDALHGDIGSVFPGDLLVLLSKSGASDELLALAPCARAKG
15232565_Arabidopsis_thalia                LSFRSSFLSPLDALHGDIGALSPRDVLVFFSKSGATEELLRLVPCARAKG
116185416_Vibrio_sp.                       TGTPSFFLHPSEAFHGDLGMITKEDVIVLISNSGETDEVLQLIPSLKSFG
116621618_Solibacter_usitat                TGTAAVFLHPAEAAHGDLGIYTPGDPTIVISKNGASSELQSLVPMLRQFR
110636722_Cytophaga_hutchi                 TGTPALFMHAADAIHGDLGMIQGGDVVICISKSGNTPEIKVLVPLIKNRG
34762344_Fusobacterium_nuclea              TGTTSIFMNSTEGLHGDLGIINQEDIVLAISNSGESDEILAIMPAIKNIG
67918831_Chlorobium_limico                 TGTTAMFLHPADAAHGDLGIVSEGDVVICLSKSGTTEELNFILPALRRIG
116329828_Leptospira_borgpe                TGTPSIFLHPADAAHGDAGIISIEDVIIAIGKSGESEELLNLIPTIKNIG
15606685_Aquifex_aeolic                    TGTPSVFLHPAEALHGDMGLLDSKDALIAISNSGESTEVLYVLQYAKALN
109673529_Campylobacter_curvus             TGTPSFFIHPTEAMHGDLGMIDKNDVVLAISFSGESDELVKILPHVKRFG
Rhizopus_oryzae                            TGTCAAFLHPVEALHGDLGVILPNDVVLALSFSGNTEEILLLIPSLKYRN
15964325_Sinorhizobium_melilo              TGTSAHFVHPTEASHGDLGMITAQDALVLLSWSGETAELANMLTYAKRFK
16264297_Sinorhizobium_melilo              TGTSAYFVHPTEASHGDLGMITAEDLLILLSWSGETVELGNVLTYAKRFN
88940199_Acidiphilium_cryptu               TGTPALFVHPAEASHGDLGMIVPGDAVIALSNSGEAAELAAIVSHVRRFA
83372399_Rhodobacter_sphaer                TGTPAQFVHPAEASHGDLGMVTRDDVALVLSNSGETPELADIIAHTRRFD
110635071_Mesorhizobium_sp.                TGTPAFFVHPVEANHGDLGMIAPDDAIIALSWSGESAELKGIVAYARRFS
116747458_Syntrophobacter_fuma             TGTPALFIHPVEAMHGDLGMVRAGDIVLALSNSGETDELNIILPSLKNIG
39996991_Geobacter_sulfur                  TGTPALFLHPAEGVHGDLGMIMKGDVVIAISNSGETEEVVRILPIIKRLG
47573351_Rubrivivax_gelati                 TGTPAMFVHPAEASHGDLGMVTPSDIVLAISNSGESDELAAILPVLKRLG
94415571_Pseudomonas_aerugi                TGTPSFFVHPAEASHGDMGMITEDDVVLALSNSGSTAEIVTLLPLIKRLG
116185625_Vibrio_sp.                       TGTSAFFVHPGEAAHGDLGMISPGDIVLAISNSGESNEILSLFPVLKRLN
68544435_Shewanella_baltic                 TGTPAFFVHPGEASHGDLGVLADNDVILAISNSGESSEILTLMPVIQRMG
                                            .     :.    :   *** *      *     :   .* :  *:

85089259_Neurospora_crassa                 LPLILLTSRPETVRQRPDTILLPAPIEPETKSFGVSPTTSTTVALSVGDA
53711463_Bacteroides_fragil                LKFIVITGNPDSPLASESDVCLSTGHPAEVCTLGMTPTTSTTVMTVIGDI
15618437_Chlamydophila_pneumo              AILVAITSMPYSNLAALSDLVVILPSVAELDPFNLIPTNSTTCQMIFGDF
15605124_Chlamydia_tracho                  VFLVGITAAPCSSLAAFSDFVIVLPKLEELDPFNLMPTTSTTCQLLFSDL
113476650_Trichodesmium_erythr             SSIILITKNPNSSLAHFADIILQIPDLPEACINGLAPTISTTCQLVLGDA
32476096_Rhodopirellula_baltic             CGIIAVTADRENPLAELADHVVPIGRHREACPDGLAPTSSTSVMLAVGDA
```

```
102191805_Rickettsia_canade         IKIAAMTMNKNSTLAKRSDFLLIVPEYSEASVIGV-PTISSLIMLSLGDA
90573271_Clostridium_diffic         SKTIAFTSNRNSVLAKECDYALIYPAKDEADHLNLAPTTSSTITLVLGDS
87123156_Synechococcus_sp.          TARIALVGRAESSLARGSDVVLEASVDREVCPLNLAPTASTAVAMAIGDA
33864177_Prochlorococcus_marin      TARIALVGKPDSSLARGSDVVLEASVDREVCPLNLAPTASTAVAMAIGDA
46447416_Candidatus_Protoc          GILVAVVCNPQSRLAAACHYVITLPFQEELCPFDMAPTMSTIFQGLFGDL
Prototheca_wickerhamik              AKLVSVSSIVGSKMDAVCDLSIHLPLKRELCPFDLAPVTSTVIQMLFGDT
115444351_Oryza_sativa              AHLISLTSAASGPLAAVCDLNVHLPLQAEVCPFGLAPVTSTAIQMVFGDT
15232565_Arabidopsis_thalia         AFLVSLTSVSGNPLAGVCDMNVHLPLQRELCPFNLAPVTSTAIQMVFGDT
116185416_Vibrio_sp.                NKVISITGRIDSTMSRNSDATLLLAQIQESCPNNLAPTTSTTLTIALGDA
116621618_Solibacter_usitat         SPLVGILGNAHSPLGAEVDVLLDASVEREADPHNLAPTASAVTALALGHA
110636722_Cytophaga_hutchi          TILIGMVGNVDSYLAVQSDYVLNVTVEREACPNNLAPTTSTTATLVMGDA
34762344_Fusobacterium_nuclea       AYIIAMTGNINSRLAKASDLYINTHVEEEGCPINLAPMSSTTNALVMGDA
67918831_Chlorobium_limico          VAIIALTGNPRSYLARNADIVLDTGIDQEACPFDLAPTSSTTAMLAMGDA
116329828_Leptospira_borgpe         AKLISMTANVESKLAKESDIVLITPVLKEACPLELAPTSSTTIALILGDA
15606685_Aquifex_aeolic             IPVIGITGNEKSSLAKYSDVVLKIPVDREACPFNLAPTVSSTVTLALGDA
109673529_Campylobacter_curvus      VKIISMARSKASSLGKFSDAFICLDIVREACPLNVAPTASTTLTLALGDA
Rhizopus_oryzae                     IPIIGLGGNSNSKLAKECAAWIDGCVEREADDLIPAPTSSTTLALALGDA
15964325_Sinorhizobium_melilo       VPIVSICSNRESTLARNSEIALVLPKVPEACPHGLAPTTSAMLQLAVGDA
16264297_Sinorhizobium_melilo       VPVISVTSNADSTIARNSTIPVVLPKVPEACPHGLAPTTSAMLQLAVGDA
88940199_Acidiphilium_cryptu        LPLVAITSRAESTLARAADLVLLLPAAPEAGPIGMAPTTSTTMQMALGDA
83372399_Rhodobacter_sphaer         IPLIGVASRAQSTLLRQSDVALLLPPAPEACGNGIVPTSSTTMTLALGDA
110635071_Mesorhizobium_sp.         IPLIAMTSGSRSALARESSVVLCLPKVQEACPHGLAPTSSTLVQLAAGDA
116747458_Syntrophobacter_fuma      TRIIAFTGDTSSTLAQYSDLTVYTGVPREACPMGLVPTASTTAMLAMGDA
39996991_Geobacter_sulfur           ATLVSMSGNPSSTLAKAGDVFLDISVTEEACPLGLAPTASTTATLAMGDA
47573351_Rubrivivax_gelati          VMLIAITGRADSNLARHAELVLDSAVAQEACPLNLAPTASTTAQMALGDA
94415571_Pseudomonas_aerugi         ITLISMTGNPESPLAKAAEVNLDASVGQEACPLNLAPTSSTTVSLVLGDA
116185625_Vibrio_sp.                IKIISMTGKPESNMAKLSDLHLQITVPQEACPLGLAPTSSTTATLVMGDA
68544435_Shewanella_baltic          VPVIAVTGKPESNMARLSKVHLCIEVPEEACPLGLAPTSSTTATLVMGDA
                                         .               :      *       *  *:      ..

85089259_Neurospora_crassa          LAMVAHELHPSVSKVFAKNHPGGAIGAALR
53711463_Bacteroides_fragil         LVVQTMKRTEFTIEEYSKRHHGGYLGEKSR
15618437_Chlamydophila_pneumo       LAMLLFHSRGVSLSTYGKNHPSGQVGMKAN
15605124_Chlamydia_tracho           LAMTLLRCRKISLSDYGSNHPSGQIGLKAN
113476650_Trichodesmium_erythr      LVVTLMSLRGFTSEDFKQFHPGGNLGA-LL
32476096_Rhodopirellula_baltic      IAVLASRLCGFTPNDFARFHPGGALGRKLT
102191805_Rickettsia_canade         LMTVIHEKRGFTKDDFKVYHPGGTIGANLT
90573271_Clostridium_diffic         IACALSKSSNFGSSDFYKYHPGGSLGEKLK
87123156_Synechococcus_sp.          LAAVWMERRGISPADFALNHPAGSLGKQLT
33864177_Prochlorococcus_marin      LAAIWMERRNISPADFAFNHPAGSLGKQLT
46447416_Candidatus_Protoc          VTAALMRRKNFSLNDYALNHPSGRIGKRMT
Prototheca_wickerhamik              VAIALMQAK---------------------
115444351_Oryza_sativa              VVAAIMEARRLSRDQYASNHPAGKIGKSLI
15232565_Arabidopsis_thalia         IAVALMAARNLSKEEYAANHPAGRIGKSLI
116185416_Vibrio_sp.                LAVALMKMRQFMPNDFARFHPGGSLGRRLL
116621618_Solibacter_usitat         LAIALMCARNFTPEEFGKFHPGGQLGRNLR
110636722_Cytophaga_hutchi          LAVALLECRNFSSEDFAQLHPGGALGKQLY
34762344_Fusobacterium_nuclea       LAGCLMKLRNFSPQNFAMYHPGGSLGRKLL
67918831_Chlorobium_limico          LAITLMQAKQFTPRDFALTHPKGALGRRLT
116329828_Leptospira_borgpe         IAMCLMELKNFKKENFALYHPAGRLGKRLS
15606685_Aquifex_aeolic             IAMTLMKLKGFSQEDFAKRHPAGALGRKLR
109673529_Campylobacter_curvus      LAVCLMKRRGFKKEDFANFHPGGSLGKRLF
Rhizopus_oryzae                     IALTLAKLRTFTVDGFALNHPGGSLGRRLL
15964325_Sinorhizobium_melilo       LAIALLERRGFSAEDFKTFHPGGKLGAQLR
16264297_Sinorhizobium_melilo       LAIALLERRGFSAEDFKTFHPGGKLGSQLL
88940199_Acidiphilium_cryptu        IAVALLARRGFTAADFGLFHPGGKLGARLR
83372399_Rhodobacter_sphaer         LAVALMEHRQFTPEHFRVFHPGGKLGARLA
110635071_Mesorhizobium_sp.         LAIALLEARGFTPDHFRTFHPGGQLGAKLT
116747458_Syntrophobacter_fuma      LAVALLRLRNFQERDFHRFHPGGHLGERLQ
39996991_Geobacter_sulfur           LAVALLIERGFRPEDFALFHPGGSLGKKLL
47573351_Rubrivivax_gelati          LAVALLDARGFKEEDFARSHPGGSLGRKLL
94415571_Pseudomonas_aerugi         LAIALLEARGFTAEDFAFSHPGGALGRRLL
116185625_Vibrio_sp.                LAVALLQARGFSAEDFALSHPGGALGRKLL
68544435_Shewanella_baltic          IAIALLQAKGFTRDDFAMSHPGGALGRKLL
                                    :
```

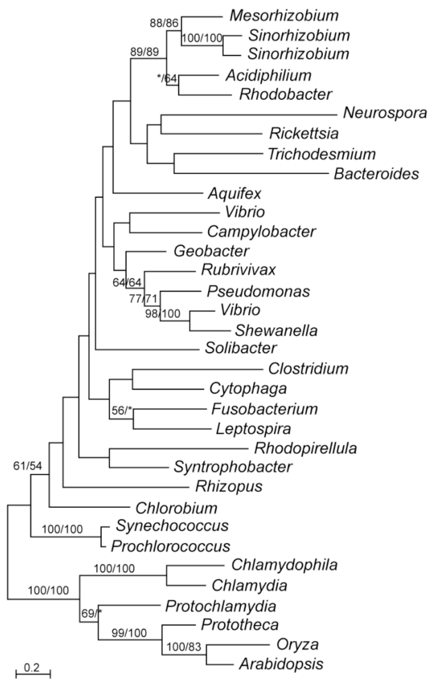

Figure 20. Phylogeny of Sugar-phosphate Isomerase, showing green plant and chlamydial sequences form a strongly supported monophyletic group. Sequences of *Prototheca* and *Rhizopus* were obtained from the Taxonomically Broad EST Database (TBestDB). No red algal homologs were identified in our search of the *Cyanidioschyzon* genome and the Taxonomically Broad EST Database.

---

21. Metal P-type ATPase

```
CLUSTAL X (1.83.1) multiple sequence alignment


42525989_Treponema_dentic          CAETLDATALSLSYATGDLAGTIAMMLEMGEILEDYTRRKSYENLTRSFL
114563186_Shewanella_frigid        NMDVSVSIAILGAYIASCVVSMFTFFLLLGRYFEQTARQKASVSSSNLHK
19704525_Fusobacterium_nuclea      NADTLSSSAIISSILLGKEALTIMFLEEVSELLTVYTMEKTRGAIKDMLS
16082160_Thermoplasma_acidop       NMDLLVSLGILTAFFFSVFSDFIVSLILTGSYVESLVKAKASDAGNRLLS
70606661_Sulfolobus_acidoc         NMDVLVSLSSNTAWIYSLISSSLITFILIGKLLEAFLKEKISEDVIKLTQ
84489487_Methanosphaera_stadtm     ----LICIAMVASLMIGEIAGEVVFIMALGALLEDYTVRKSKQGLRDLIN
78189332_Chlorobium_chloro         NMDVLIALGSLASYLYSIASAMIITSILLGRFLEAGSRLKANNAIAELAE
71417434_Trypanosoma_cruzi         TMDTLVAIGAGCSYLYSLGAGMLLAFMLLGRFLEARARRSTSDALISLMN
15606387_Aquifex_aeolic            GMMTLVGLAISVAYIYSVYTTLIVVM-LWGHYVEMKSVLGAGRALEELVK
6320475_Saccharomyces_cerevi       TMDTLVCVSTTCAYTFSVFSIMIISYISIGKYLETLAKSQTSTALSKLIQ
42573159_Arabidopsis_thalia        NMNTLVGLGALSSFSVSSLPVMLIAFVLLGRNLEQRAKIKATSDMTGLLS
75907770_Anabaena_variab           NMNTLVGLGTLTAYTASVVPVMMLGFILLGRTLEQQARGKAAAAFRQLLA
16331210_Synechocystis_sp.         NMNTLVALGTGSAYLTSCIPVMLLGMLLLGRTLESKARQKAKSALTELLA
15643086_Thermotoga_mariti         NMDTLIFFGAVTSWVTALLGAMIVAFHITGRFIESYLRDRASKEIKALLK
```

```
46445712_Candidatus_Protoc          NMDVLVVLGSTAAYLFSFYSALIITLILFGRWLEAVTKGKTSKALERLYH
45549362_Drosophila_melano          NMDVLISMVTTISYVYSVAPPMLLIFISLGRWLEHIAKGKTSEALSKLLS
55743071_Homo_sapien                NMDVLIVLATSIAYVYSLVPPMLFVFIALGRWLEHLAKSKTSEALAKLMS
115444827_Oryza_sativa              NMDVLVALGTNAAYFYSVYSAMLISFILLGKYLEVVAKGKTSDALSKLTE
56962006_Bacillus_clausi            NMDVLVALGTSAAYFYSVYSAVLITLIMVGKLLESKAKTKSTKAITTLVK
116330313_Leptospira_borgpe         NMDVLVVLGTSAAYFYSVYSSILITLILFGKFLEHIAKGKSSKAIQSLVG
118060384_Pseudomonas_mendoc        NMWTLIGLGTAAAYLYSVAAAVIISLTLLGQMLELKARSQTSAAIKSLLR
110635001_Mesorhizobium_sp.         NMWTLISIGVGTAYVYSVIAAVIVALVFLGQVLELRARERTGSAIRALLD
87306843_Blastopirellula_marin      NMFTLIAVGTGAAYLYSVLAAVIIALVLLGQVLELRARKKTSGAIRELIA
52841258_Legionella_pneumo          NMFTLIAMGIGVAWIYSMVAAVITTLVLLGQVLELKAREQTGSAIRALLK
53713321_Bacteroides_fragil         NMDTLVALSTSIAFLFSVFAVVIIAFVLTGKLMEERAKGNTSTAIRKLMG
78188927_Chlorobium_chloro          NMDTLIGIGTLCAYLYSATTIVVIGFVLLGKYLEARSKMKTGEALEALIT
67921918_Crocosphaera_watson        DMNSLVTLGTGAAFLYSLFAVVIITLILLGRLLETRARSKTSEAIGNLMG
21228430_Methanosarcina_mazei       DMNLLIAAGTGSAYLISVAVAFLIIFIVFGRYLETRARGRTSEAIRKLMG
28210589_Clostridium_tetani         NMDSLIAIGTLSAYLYGIFAAVILTLITLGKYLEAVTKGKTSEAIKKLMN
89340248_Mycobacterium_flaves       TMDTLISLGTVSAFLWSLYAAGVTTFILAGRYFEKRSKRQAGAALRALLN
83374897_Rhodobacter_sphaer         DMNSLVAVGTFAAWAYSTVAAVIVVLILLGRLMEARARGQAGAAIAGLLR
94972021_Deinococcus_geothe         DMNALVMIGTTAAFLYSLVSGVVITLILLGKYFEAIAKGRSSEAMKKLLS
76258102_Chloroflexus_aurant        TMDTLIALGSSAAYFYSLAAALIITLILVGKYLEARAKGQTSAAIKALIG
71366115_Nocardioides_sp.           SMDTLVALGTGVAYGYSAFALIVMALILMGRWLELKAKKRTAASIKALVG
CM4147_Cyanidioschyzon_merolae      SVDVLMNLAALLLLAVGHLGTLLFVLFSVSRAAEGAIRARARRDLDALRD
92875650_Medicago_trunca            NINILVLLAVCGTAALQDFGGVIIFLFSIAQWLETRATHKALVAMSSLTN
15224717_Arabidopsis_thalia         DINILVIITVIATLAMQDFAAAVVFLFTISDWLETRASYKATSVMQSLMS
115469636_Oryza_sativa              NIHVLMALAAFASIFMGNSGGLLLAMFNLAHIAEEHFTSKSMIDVRELKE
63253545_Arabidopsis_cp             NIHVLMALAAFASVFMGNAGGLLLAMFNLAHIAEEFFTSRSMVDVKELKE
89897922_Chlamydophila_felis        NIDILMTSAAFGSIFIGGAGSLLLVLFAISEALGQMVSGKAKSTLASLKH
15605460_Chlamydia_tracho           NIDILMTSAAFGSIFIGGAGALLLVLFAISESLGAMVSGKAKSTLASLKH
53712588_Bacteroides_fragil         SEFTLMSVATLGAFYIGEYGVAVMLFYSVGELFQDKAIDKAKRNISALLD
42526892_Treponema_dentic           DENFLMSVATIGAFILGDWGAAVMLFYNLGEVVQHSAVEKSRRSIIDLMD
28211582_Clostridium_tetani         DENFLMAVATIGAIAIKEYAVAVMLFYKIGEVFQDRAINHSRKSIASLVD
19703603_Fusobacterium_nuclea       DENFLMTIATLGAIIIGEYAVAVMLFYEVGELFQGYAINKSRKSIADMMD
46445921_Candidatus_Protoc          SINLLMFIAVVGAFYLGEYGTIVIILFTLAERLEDVGIAKSQSAIDSFLN
83815585_Salinibacter_ruber         EVDFLMLVAAGGAAVLGKWGAFLLFLFSLGHALEHYAMRRARRAIESLAD
94972049_Deinococcus_geothe         TINTLISVAAIGAIAIGEAGALVVFLFAIGELLENVAAGRARAGIQALAA
77462336_Rhodobacter_sphaer         TIEGLMTVAAAGALFIGAAAALVIFLFAVGEVLEGVAASRARDGIRALSR
84488951_Methanosphaera_stadtm      TPAVLMCIAAVASFLIGHPGAAVTFLYFIAEFLEDYSEQRAQHSIKSLVE
89340253_Mycobacterium_flaves       GVGTLMTIAAIGAVALGQFAALLGILFSIAEGLEHYAVSRTRRGLRSLLS
71368538_Nocardioides_sp.           GVGTLMTIAAVGAVILGEVAAILAFLYSISEGLEEYAVARTRHGLRALLS
76258037_Chloroflexus_aurant        EVDLLMVLAALGAAYLGEFGAILLFLFSLSNVLQVYAMRRTEQAITALMQ
75910063_Anabaena_variab            DVDLLMIVAALGAASLGLWGAILILIFAISGALEGYAMRHTERSIRSLMS
16331905_Synechocystis_sp.          DVDLLMIVAALGAAGLGLWGAVLILIFAISGALEGYAMQRTERSIQGLMS
56962051_Bacillus_clausi            DMKTLMTLAVIGGAIIGEWVAVVVILFAISEALERFSMDRARKSIQSLMD
52841244_Legionella_pneumo          GIEFLMSLAAIVAGIMGQWAATLVFLYSISEAAEGYSGERARHAIRALMD
118065328_Roseiflexus_casten        SINALMTIAAVGAVIIGAHAGLVMVLFAIGEAIEGFTAARARDAIRSLMT
87311031_Blastopirellula_marin      DMNVLMCVAILGACLLGDFAAMVAFLFSTSLLLEQWSVARARRSISDLMT
110347018_Mesorhizobium_sp.         DMNLLMVVAVAGAIGIGAWAATVSFFFALALALEAWSLGRARRAVAALME
118058132_Pseudomonas_mendoc        NINALMSIAVTGAVLTGQWAAMVMVLFTLAELIEARSLQRARNAIASLMD
114564618_Shewanella_frigid         NMNALMSIAVTGAMLIGHWAAMVMVLFALAEVIEAKSLDRARNAIRGLLD
                                                          .      .

42525989_Treponema_dentic           NTKETVHVLEISANLLQAGDTVILRIGSVIPADGTVVSGEASVNQASMTG
114563186_Shewanella_frigid         LVPLTAHLYEIAAKQLVIGDIILVKPGEVIAADAEVVNGLSSVNEAMLTG
19704525_Fusobacterium_nuclea       VGENYVWKERVPIEEIQKDDIIVVQTGEKISVDGKIIRGEALIDQSSITG
16082160_Thermoplasma_acidop        LIPETVHLVDIESSKISPENLVEVRPGETVPVDGIVERGSADVDSSMITG
70606661_Sulfolobus_acidoc          VK---ARKIEINSNELKVGDLVVVKTGDIIPADGVVESGEATVDESIYTG
84489487_Methanosphaera_stadtm      LKPQTGRLLEIKVQEIKKEDKLRVLPGEVIPVDGIIISGDTSVDQSVMTG
78189332_Chlorobium_chloro          LQPHEALRVRVAVATIQSGEVIEILAGNRIPLDGKVVEGEAEVNEAMLSG
71417434_Trypanosoma_cruzi          LVPPVSIVVTMPSSLLEKGMRVRVLAGDRFPVDGIVVEGMSDVDEQMVTG
15606387_Aquifex_aeolic             LIPSKAHLVDVPVEKLKKGDLVLVRPGEKIPTDGTVVEGNTHVDESMLTG
6320475_Saccharomyces_cerevi        LTPSVCSIIEIPIELLQVNDIVEIKPGMKIPADGIITRGESEIDESLMTG
42573159_Arabidopsis_thalia         VLPSKARLLEVPCNSLSVGDLVVILPGDRVPADGVVKSGRSTIDESSFTG
75907770_Anabaena_variab            LQPQIARLIEIPAEQVKVGEWLQVLPGDKIPVDGEVRFGQTTLDESMLTG
16331210_Synechocystis_sp.          LQPSLARLVEIPVEQVRVGEWVQVLPGEKIPVDGILVAGKTLVDESLLTG
15643086_Thermotoga_mariti          LQAKEARVIMMPIEAVKEGFIVLVKPGERIPVDGVIVEGQSSIDESVVTG
46445712_Candidatus_Protoc          LQPKIARIEEVDVASIQVGDIFMVRPGDHIPVDGWVIEGESSINESLLTG
```

```
45549362_Drosophila_melano          LKAADALLVVISVDYVQRGDILKVIPGAKVPVDGKVLYGHSSCDESLITG
55743071_Homo_sapien                LQATEATVVQVPMELVQRGDIVKVVPGGKFPVDGKVLEGNTMADESLITG
115444827_Oryza_sativa              LAPETACLLEISTQLLQRNDVIKIVPGEKVPVDGVVIKGQSHVNESMITG
56962006_Bacillus_clausi            GQAKTALKVHIAVDSIQVGDLLLVKAGEKVPVDGKIVKGITTIDEAFITG
116330313_Leptospira_borgpe         LQPKSARIIKIPLFAVRPGDLLLIKAGETIPVDGTVEEGSSSVDESMLTG
118060384_Pseudomonas_mendoc        LAPKTARRIDIPLTHVHQGDHLRVRPGEKVPVDGTVLEGESAVDESMLTG
110635001_Mesorhizobium_sp.         LAPKTARRLDIPLEEVHSGDRLRVRPGESVPVDGTVIDGRSSVDESMITG
87306843_Blastopirellula_marin      LAPPVAHRVDVDLDEVHAGDLLRVRPGEKIPVDGRVTEGESSVDESMLTG
52841258_Legionella_pneumo          LVPESAHRIEVSLDNVAVGDLLRVRPGEKIPVDGEVQEGRSFVDESMVTG
53713321_Bacteroides_fragil         LQPRVARVLDILIDQLQTGDLVVVRPGEQIPVDGRLSEGESYVDESMISG
78188927_Chlorobium_chloro          LQAKSALVEEIPIGQVRRGDTIMVRPGEKIPVDGIITDGTSAVDEAMVTG
67921918_Crocosphaera_watson        LQAKTARVIDIAVEDVIIGDIVLVRPGEKIPVDGVIIEGQSTLDESMITG
21228430_Methanosarcina_mazei       LRAKTSRILEVPVEEVVVGDIVIVRPGEKIPVDGIVVEGSSAVDESMITG
28210589_Clostridium_tetani         LAPKTATVISISVDEVEVEDIVIVKPGARLPVDGIIVEGITSIDESMLTG
89340248_Mycobacterium_flaves       LGAKDVSVLKIAIEELMVGDEFVVRPGEKIATDGVVTSGSSAVDASMLTG
83374897_Rhodobacter_sphaer         LQPKTARVSEVPIESIGPGAILLIRPGERIPLDGVVVAGESAVDESMLTG
94972021_Deinococcus_geothe         LQAKTARVVELPTDEVLVGDLISVRPGEKIPVDGEVVQGASFVDESMITG
76258102_Chloroflexus_aurant        LQPKTARVVDVPLTEVRVGEMIIVRPGEKIPVDGVIVSGESTIDESMLTG
71366115_Nocardioides_sp.           LAPTTARVLDVPIAEVRVGDHVRVRPGEKLPVDGVVAEGRSAVDESMLTG
CM4147_Cyanidioschyzon_merolae      SVPEYAWRLQVPVTELTPGDLILVKAGEVVPCDCQIARGDAYVSLEPITG
92875650_Medicago_trunca            MAPQMAIVARVDVNDVKMNTILAVKAGDAIPLDGIVVEGKCEVDEKMLTG
15224717_Arabidopsis_thalia         LAPQKAIIAEVEVDEVKVDTVVAVKAGETIPIDGIVVDGNCEVDEKTLTG
115469636_Oryza_sativa              NHPEFALLLKVPVHDLEVGSHILVRAGEAVPVDGEVYQGSSTVTIEHLTG
63253545_Arabidopsis_cp             SNPDSALLISVPVHSVKVGSYVLVGTGEIVPVDCEAYQGSATITIEHLTG
89897922_Chlamydophila_felis        LAPTIAWVVKTPINQVLVGNIIRVKSGEIVPLDGEIIHGSSSINLMHLTG
15605460_Chlamydia_tracho           LAPTVAWVVKVLVQNVKVGEIIRVKSGEVVPLDGKIIQGASSINLMHLTG
53712588_Bacteroides_fragil         VRPEKAVVVTVDPRSVLINEIIEIKAGERVPLDGVMLDEVAAFNTAALTG
42526892_Treponema_dentic           LRPDFARLYLVDPASVKIGSLVLVKAGEKIPLDGVIYEGSAELDTSSMTG
28211582_Clostridium_tetani         IRPEFANVKKVSPEQVEIGDIIIVKPGEKIPLDGEVIEGESYLDTSILTG
19703603_Fusobacterium_nuclea       IKPEYANVIKVDPDEVQINEIIEIKPGERVPLDAIIIKGESTLDTSALTG
46445921_Candidatus_Protoc          KIPKTVFIKPIDISQVKIGTVVLIKPGQMIPLDGIVKSGSSFVDESAITG
83815585_Salinibacter_ruber         LAPDTALVREVSVDQLRVGDTVIVKPNERIPADGFVTQGESAVNQAPVTG
94972049_Deinococcus_geothe         LAPKTALLLEVPVEQLQVGQLVRVQPGGRVPADGTITEGDSNLDDSPVTG
77462336_Rhodobacter_sphaer         LVPETARLEEVPAAGLQPGQIVRVRPGERIPADGEILEGQGGLDESPVTG
84488951_Methanosphaera_stadtm      IAPEKARLKTVSIDEVKVGEIVIVKPGDKVPLDGKVIEGVSTINQSSITG
89340253_Mycobacterium_flaves       LVPPKASVLTVAPGDLVVGDVMVIRPGERAATDGTIRSGQTSLDLSAITG
71368538_Nocardioides_sp.           LVPAEATILRVSPADLVIGDTLLVRPGEQVATDGVIRTGRTALDVSALTG
76258037_Chloroflexus_aurant        LRPDRVSIRDVAIEEVQVGDVMLLRPGDRVPLDGVIERGAGTFDESALTG
75910063_Anabaena_variab            LTPDTATIVTIAISQLQVGDEIVVKPGELIPTDAVIVSGYSTINQAAITG
16331905_Synechocystis_sp.          LTADVARVLTIPISELKMGDQVLVKPGELVPTDGLVIEGFSTLNQASITG
56962051_Bacillus_clausi            IAPNEALIKTVHVGDIVVGDIMIVKPGQKIAMDGIVVHGHSSVNQAAITG
52841244_Legionella_pneumo          LAPKTALIRRIPIEEIHIGDEFIVLPGESLATDGEVISGHSSVNQAPITG
118065328_Roseiflexus_casten        LAPETALRLRVPVAELEVGDVIIVRPGERVPMDGVVRSGATHVDQAPITG
87311031_Blastopirellula_marin      QTPPTAHVREKPLAEINVGQICLVKPGERAPLDGVITTGSPAINQAPVTG
110347018_Mesorhizobium_sp.         LAPPTARVKDVPAAEVRVGARIIIRPGDKVPLDGRVAAGESEVNQAPITG
118058132_Pseudomonas_mendoc        LAPPRATRREVDVQAVAVGDVLRVRPGERIGLDGEVIAGASTVNQAPITG
114564618_Shewanella_frigid         LTPEQATVQEVGAKQITIGARVRVKPGERIALDGEVLEGRSAVNQAPITG
                                                   :       :  .     *               :*

42525989_Treponema_dentic           EGLPVHKVSGDTVFASTVVEEGEIHVCVKACGRETRVSKIADMIDRSQSL
114563186_Shewanella_frigid         EQMPIGKQINDTVYAGTINVEQPLQVKVTALGQDQLVAEIIRLQELASNN
19704525_Fusobacterium_nuclea       EYMPIKKSEGEEVYAGTIIKNGNISIIAEKVGDDRTVSRIIKLVEDANSN
16082160_Thermoplasma_acidop        ESLPITVNPGSKVSSGMKDLNGTIVVRVQAVGPNSTVGKIYSFIKMASSG
70606661_Sulfolobus_acidoc          EALPTTKKKGDPVIGGSIVQTGALTVYVTRSGNRAYISQVVSAIKEAEFT
84489487_Methanosphaera_stadtm      ESLPLDKTVNDEVYAGTLNLYGAIDIISTKIGKDSSLEKLIRMVKEADEK
78189332_Chlorobium_chloro          ESTPVLKGMDSEVFAGSTAMNGRLRVRVSGNASTTLLAQIIRTVEEAQAR
71417434_Trypanosoma_cruzi          EAITKAVKVDSAVIGGTTNITAMLVVEATKIGEETMLSQILRLVQEAQNT
15606387_Aquifex_aeolic             ESRPVPKKPGDKVIGGSVNLEGSIKVRVEKTGEETYLKQVIKLVKEAQES
6320475_Saccharomyces_cerevi        ESILVPKKTGFPVIAGSVNGPGHFYFRTTTVGEETKLANIIKVMKEAQLS
42573159_Arabidopsis_thalia         EPLPVTKESGSQVAAGSINLNGTLTVEVHRSGGETAVGDIIRLVEEAQSR
75907770_Anabaena_variab            EAVPVMKQPGDIVTAGTINQSGAIAIQATRTGNDTTLAHIVALVEAAQTR
16331210_Synechocystis_sp.          ESLPVAKNVDDAVIAGAWNQSGAIIIAATHIGAETTLARIIQLVETAQTQ
15643086_Thermotoga_mariti          ESIPVLKKENDEVIGGSLNVSSPIKIKVTKVGEDTFLSQMVKLIQEAQGS
46445712_Candidatus_Protoc          ESLPVYKKQGSKLYAATINETGYLKGQASSVGSDTILAQILRAVEKAQSS
45549362_Drosophila_melano          ESMPVAKRKGSVVIGGSINQNGVLLVEATHTGENTTLAQIVRLVEEAQTS
```

```
55743071_Homo_sapien                EAMPVTKKPGSTVIAGSINAHGSVLIKATHVGNDTTLAQIVKLVEEAQMS
115444827_Oryza_sativa              EARPIAKKPGDKVIGGTVNDNGCIIVKVTHVGSETALSQIVQLVEAAQLA
56962006_Bacillus_clausi            ESLPQDKTVGSFVYGSTMNITGAIVVEATTNGNETVLDDIVRVVREAQQS
116330313_Leptospira_borgpe         ESIPVEKTISSPLYGSSLNQNGILKLRASKVGKDTLLSEIIRVVRETQGS
118060384_Pseudomonas_mendoc        EPLPVTKRVGDALIGATLNTHGSLVMEAQKVGAETMLSQIVQMVARAQRS
110635001_Mesorhizobium_sp.         EPVPVEKVEGDLLTGGTLNKNGSLIMRAEKVGKDTMLSQIVEMVAKAQRS
87306843_Blastopirellula_marin      EPNPVKKSTGEEVIGGTVNGTGSFLMEAEKVGEETVLSHIVDMVANAQRS
52841258_Legionella_pneumo          EPIPVAKEASAKVIGATINQTGSFVMKALHVGSDTMLARIVQMVSDAQRS
53713321_Bacteroides_fragil         EPIPVEKKVGDRVLAGTINQKGAFVIKASGVGSETVLARIIRMVQEAQGS
78188927_Chlorobium_chloro          ESLPVDKKAGDGVIGGTMNKQGSLTFIAAKVGSDTMLARIIRMVEDAQSS
67921918_Crocosphaera_watson        ESIPVEKQTGDEVIGATINKTGSFKFEARKVGKDTTLSQIIKLVEEAQNS
21228430_Methanosarcina_mazei       ESIPVEKGEGDTVIGATINRMGSFRFRATKVGADTALAQIIRLVEAAQTT
28210589_Clostridium_tetani         ESIPVEKNKDDKVIGGSINKNGYIKYRATKVGKDTTLAQIIKLVEEAQGS
89340248_Mycobacterium_flaves       ESVPVEVAAGDTVVGATVNAGGRLVVRASRVGSDTQLAQMAELVERAQTG
83374897_Rhodobacter_sphaer         EALPVAKAAGADVTGGTVNGTGALTMRATRVGADTVLARIVAMVGEAQGA
94972021_Deinococcus_geothe         EPVPVSKQPGAPVVGGTINQNGALTFRATRIGADTALAQIIKLVETAQGS
76258102_Chloroflexus_aurant        ESLPVEKRSGDPVFGATINRSGSFQMRATRIGKDSALAQIIRLVQEAQGS
71366115_Nocardioides_sp.           ESVPVEKVAGDAVIGATVNTTGSLVVRTTAVGADSTLAQIVRLVEEAQAS
CM4147_Cyanidioschyzon_merolae      ESVPRRVRDGELIAAGARTVDGSLYLRVQRPVHESAVARIARLVTNAQQN
92875650_Medicago_trunca            ESFPVTKESDSLVWAGTINMNGYISVRTTVLAKDTVVARMSRLVEEASSR
15224717_Arabidopsis_thalia         EAFPVPKQRDSTVWAGTINLNGYICVKTTSLAGDCVVAKMAKLVEEAQSS
115469636_Oryza_sativa              ETKPLERTVGDAIPGGARNLEGMMIVKVTKSWEDSTLNRIVQLTEEGQLN
63253545_Arabidopsis_cp             EVKPLEAKAGDRVPGGARNLDGRMIVKATKAWNDSTLNKIVQLTEEAHSN
89897922_Chlamydophila_felis        EKIPKSCQVGSIIPAGAHNLEGSFDLKVLKTGADSTIAHIINLVIQAQKS
15605460_Chlamydia_tracho           EKIPKSCGIGDTIPAGAHNLEGSFDLQVLRIGAESTIAHIINLVVQAQSS
53712588_Bacteroides_fragil         ESVPRDISRGEEVLAGMIVTDKVIRMKVTKPFDKSALARILELVEDASER
42526892_Treponema_dentic           ESLPRTAEKGSPVLAGFVNLTGVITVKTTASLENTAASKMLELIESAQNR
28211582_Clostridium_tetani         ESVNRKVEKGDIVLSGSINNTSLLTVKVTKTFGQSAVSKILDLVENASSR
19703603_Fusobacterium_nuclea       ESLPVEVREGATILSGCININALIIAKVTKEYFDSTVNKVLDLVENAAAK
46445921_Candidatus_Protoc          EPLAKDKRTGDELFAGSLNKQGFIEMEVSKTASDSTIEKIRELTFHATKH
83815585_Salinibacter_ruber         ESVPVDKTPVETVYAGTINGSGVLEVRVTKRAGESTLSRVVQMVTEAEAE
94972049_Deinococcus_geothe         ESVPVHKSAGDTVYAGSINTDGVLTVQVDRGASDNTIARIIHLVEEAESS
77462336_Rhodobacter_sphaer         ESIPVTRGPGEAVFAGAVATDAALRIRVTRAAKDNTIARIVRLVEEAEEA
84488951_Methanosphaera_stadtm      ESIPVTKTIKDKVFAGTVNQDGYLEIEVTKASKDSVISKIVTLVKQSQLN
89340253_Mycobacterium_flaves       ESVPVEAGPGSDVFAGAINGGGAIEVEVTALTADSSLARIVHIVEQAQER
71368538_Nocardioides_sp.           ESVPVEAGVGDTVYAGSINGTGVLEVEVTTTAEDNSLARIVNIVEAEQSR
76258037_Chloroflexus_aurant        ESMPVHKGPGMTVLAGTLNQTGALEVRVTKPASESTLARIIAMVSEAQSR
75910063_Anabaena_variab            ESLPVEKTVGEEVFAGTLNGYGALKLRVHKPAASSLIQRVIRLVEQAQTQ
16331905_Synechocystis_sp.          ESMPVEKAIGDEVFAGTINGNGVLRLKIHQPPESSLIQRVIRLVQQAQTE
56962051_Bacillus_clausi            ESVPVEKAVDDEVFASTLNEEGLLEIEVTKLVEDTTISKIIHLVEEAQGE
52841244_Legionella_pneumo          ESIPVEKTIGSKVFSATINGEGTLTVRATKKFADNTLSRIIFLVEKAQDK
118065328_Roseiflexus_casten        ESVPVVKTPGAHVYAGSINGEGVMEVVVTHPASENTISRMIRLVQEAQDR
87311031_Blastopirellula_marin      ESIPVEKNVGDEIYAGTINGGSSFQFEVTHAAGDTMLDRIAKMIDDAHKQ
110347018_Mesorhizobium_sp.         ESVPVFKAEGDDVFAGTINGEGAIEVVTTKAANDTTLAQIIRMVGSAQSR
118058132_Pseudomonas_mendoc        ESLPLDKGVGDTLFAGTINQAGSLEYRVTAAARDTTLARIIHAVEEAQGS
114564618_Shewanella_frigid         ESLPVEKSPGDSVFAGTINESGSFEYRVTALANNSTLARIIHAVEAAQGS
                                    *           : ..       .               :

42525989_Treponema_dentic           KAASQIKSERTADKLVFYNFLAGLTYAFTAKAASTLLVDYSCAMKLSAPV
114563186_Shewanella_frigid         KPAIALVADRLARYFSATILIAALTYIVWWITLSVLVATCPCALALATPT
19704525_Fusobacterium_nuclea       KADIQNYADTFSAQLIPLNFLAGIVYASTTKAMSMLVIDYSCGIRLSTAV
16082160_Thermoplasma_acidop        RTKIQRIADVFSSYFVPVVLAATASFLFWLSFVSVIVIACPCAIGLAGPI
70606661_Sulfolobus_acidoc          KLPIQKLVDKVSSIFVPFVIISVLSSIFWLIGIAVLASACPCGFGLATPT
84489487_Methanosphaera_stadtm      QAPTQRIADKWATWLVPVALIAIITYI--ERAVTILVVFCPCALILATPT
78189332_Chlorobium_chloro          KAPIQSLADKTAAYFVPAIILATVTFLYWMNAVSVLVIACPCALGLATPL
71417434_Trypanosoma_cruzi          KPEIQRIADQIAAYFVPFVVFSVGVFFTWDFFISSVVAACPCALGLATPT
15606387_Aquifex_aeolic             KTKLQSLADRAAFYLTVIAVLGSISLLFWERAVTVMVTACPHALGLAIPL
6320475_Saccharomyces_cerevi        KAPIQGYADYLASIFVPGILLAVLTFFIWQTATSVVIVACPCALGLATPT
42573159_Arabidopsis_thalia         EAPVQQLVDKVAGRFTYGVMLSAATFTFWQLSCSVLVVACPCALGLATPT
75907770_Anabaena_variab            KAPIQKLADTVAGYFTYGILASVLTFIFWKLAIAVMVVACPCALGLATPT
16331210_Synechocystis_sp.          KAPMQRLADQVAGWFAYGVLIALVTLGFWKLSVSVLVVACPCALGLATPT
15643086_Thermotoga_mariti          KVPIQALADRITMWFVPTIILAITSALVWFVFVATIVIACPCALGLATPM
46445712_Candidatus_Protoc          KAPIQKFVDKISAVFVPCVLISCLTILIWINAVSVLVIACPCVLGLATPA
45549362_Drosophila_melano          KAPIQQLADRIAGYFVPFVVVSSITLIAWKCALSVLAIACPCALGLATPT
55743071_Homo_sapien                KAPIQQLADRFSGYFVPFIIMSTLTLVVWQTSITVLCIACPCSLGLATPT
```

```
115444827_Oryza_sativa              RAPVQKLADRISRFFVPTVVAAFLTWLGWQFGISVLVVACPCALGLATPT
56962006_Bacillus_clausi            KAPIQRFADRISAIFVPIVVFALFTFFVWEAAIAVLVISCPCALGLATPT
116330313_Leptospira_borgpe         KAPIQRIADQISGIFVPVVVISILTLFLWEKAIAVLVIACPCALGLATPI
118060384_Pseudomonas_mendoc        KAPMQRMADAVAGYFVVGVIIAILTFFGWINAVAVLIIACPCALGLATPM
110635001_Mesorhizobium_sp.         RAPIQGLADRVSYYFVPTVVVAIIAFFVWVSAVSVLIIACPCALGLATPM
87306843_Blastopirellula_marin      RAPIQRIADQVAGYFVPAVLISLLTFAVWVNAVAVLIIACPCALGLATPM
52841258_Legionella_pneumo          RAPIQRLADTVSGWFVPAVIVAVLSFIVWIAAVSVLIIACPCALGLATPM
53713321_Bacteroides_fragil         KAPVQRIVDRVTGIFVPVVLIAVLTFVIWLSAVSVLVIACPCALGLATPT
78188927_Chlorobium_chloro          KAPIQQLADKAAAIFVPTVLLAALTCILWMGTVGILVVACPCALGLATPT
67921918_Crocosphaera_watson        KAPIQKIADQVTAWFVPAVMIAVISFICWVTTMSVLIIACPCALGLATPT
21228430_Methanosarcina_mazei       KAPIQRIADVFAGNFIVTVHIALLAFFFWLIAITVLVISCPCAVGLATPA
28210589_Clostridium_tetani         KAPIARMADVIAGYFVPIVILAIISSLAWTIFISVLVIACPCALGLATPT
89340248_Mycobacterium_flaves       KAEVQRLADRISGVFVPIVIIAVITLGAWTAAVAVLVIACPCALGLATPT
83374897_Rhodobacter_sphaer         KLPVQALVDRITLRFVPVVMVAALTVLVWVAGVSVLIIACPCAMGLATPV
94972021_Deinococcus_geothe         KPPIQGLADRVVAVFVPVVLIAALTFLLWITTVAVLIIACPCAMGLATPT
76258102_Chloroflexus_aurant        KAPVQALVDRVSAVFVPIVIIALITFLGWIFAVAVLVIACPCALGLATPT
71366115_Nocardioides_sp.           APPMQRLADRVSAWFVPAVLVALLTFLGWGTTVAVLIIACPCALGLATPT
CM4147_Cyanidioschyzon_merolae      KPTLERWIDRFGQLYSRFVVGALLVFIVAERALGFMVVASPCALIIGAPV
92875650_Medicago_trunca            KSLAQRFIDNFAKYYIPVVVSASIAVVPAHLALVVLLSGCPCALILSTPV
15224717_Arabidopsis_thalia         KTKSQRLIDKCSQYYTPAIISACVAIVPVHLALVVLVSGCPCGLILSTPV
115469636_Oryza_sativa              KPKLQRWLDEFGEHYSRVVVSLVVALLGPYRGLGLMVAASPCALAVA-PL
63253545_Arabidopsis_cp             KPKLQRWLDEFGENYSKVVVSLAIAFLGPYRALGLMVAASPCALAVA-PL
89897922_Chlamydophila_felis        KPRLQQRLDKYSSAYALTIFSIAIAILVPYRALAFLISASPCALIIAIPI
15605460_Chlamydia_tracho           KPKLQQRLDRYSSTYALTIFSACIAIGGSYRALAFLIAASPCALIIAIPI
53712588_Bacteroides_fragil         KAPAELFIRKFARVYTPIVIFAFLIVLIPYKALVFLVISCPCALVISIPL
42526892_Treponema_dentic           KARVERFITSFAKIYTPIVTAVLLSVLPPSRGLVFLVISCPCAFVISVPL
28211582_Clostridium_tetani         KAPTENFITKFARYYTPVVVAIALAVLPPYRALVFLVISCPCALVVSIPL
19703603_Fusobacterium_nuclea       KSTSERLITRFAKIYTPIVIAVLLAILPPFRALSFLVVSCPCAFVISVPL
46445921_Candidatus_Protoc          KAKTQQFIEIFSQFYTPFVIATFWTFFNWADALALLVIACPCALVISTPI
83815585_Salinibacter_ruber         KSPTERFTDRFERVFVPAVLLVGLLLFAWYRAMAVLVAASPCALAIATPS
94972049_Deinococcus_geothe         KAPTARFIDRFSRWYTPAAMAFLFAVLPPYKGVALLLIACPCALVLSVPA
77462336_Rhodobacter_sphaer         RAPTERFIDRFSRWYMPLIVAVAVAVLPPYRGLALLLIGCPCALVISVPA
84488951_Methanosphaera_stadtm      RSHTETYVEKIAKYYTPAIITICVAIIPPYRALSLMVISCPCAFLISTPI
89340253_Mycobacterium_flaves       KGAGQRLADRIARPLVPAIMAAGIAAVGAERALVVLVAASPCALAIAVPL
71368538_Nocardioides_sp.           KGNAQRLADRIAKPLVPGIMLAAIIAVAGERALVVLVAASPCALAISVPV
76258037_Chloroflexus_aurant        KARSQSFLERFEQRYAIGVIVALFILLMPYRGMVLLTVASPCALVISVPA
75910063_Anabaena_variab            APPSQQFIEVFERGYAKVIVGILLATLPPYRALTFLVVASPCALMAAIMP
16331905_Synechocystis_sp.          APPSQQFIERFECGYAKVIVGLLLGTLPPYRALIFLVVASPCALMASIMP
56962051_Bacillus_clausi            RAPAQAFVDKFAKYYTPIIMAALVAIIPPYQGLAVLVVGCPCALVISTPI
52841244_Legionella_pneumo          KGRSQRFIERFGNLYSPFVLVGILIATLPIRATVFIVAAAPCALVISIPI
118065328_Roseiflexus_casten        RAPVQRMIDRFAGWYTPAVVAALMATVPPYRSLALLVVACPCALVISTPV
87311031_Blastopirellula_marin      RAPSQQWVEKFSAYYTPAMMAAAVMILPTYNGLILLVIACPCALVIATPV
110347018_Mesorhizobium_sp.         RAPSEQWVEKFARVYTPVVMAVAIFLAPPYRALVLLVIACPCALVISTPV
118058132_Pseudomonas_mendoc        RAPTQRFVDRFSRIYTPTVFALAVAVLPPYRALVLLVVACPCALVISTPV
114564618_Shewanella_frigid         RAPTQRFVDQFARWYTPVVFAIAVALLPPYRALVLLVVACPCALVISTPV
                                                                       :    .  .  .

42525989_Treponema_dentic           CVLSAMKNCAEHGITVKGGKFLEDFARADTIVFDKTGTLTESQPSVKKII
114563186_Shewanella_frigid         AVTCATAIFTKLGVIPRKAGIFEKLTKIDHVVFDKTGTLTCGQLSIGEVQ
19704525_Fusobacterium_nuclea       AFSAAINTAAKNGILVKGSNFIEELSKSETVIFDKTGTITEGKPKVQSIE
16082160_Thermoplasma_acidop        TLLIASEGSFEAGILVKNAGAMDRVTKVNRVVFDKTGTLTLPEMYVLNYS
70606661_Sulfolobus_acidoc          AIMIGIRKLAKTGIIVRNGDALQRLNETKRVIFDKTGTITSGKIKIIKTE
84489487_Methanosphaera_stadtm      AIMAAIGQATKHGVLIKSGEALEKMGNVNSIAFDKTGTLTYGNLEVSDII
78189332_Chlorobium_chloro          ALLVATTATSRHGLLVKGGDVMENLSTITTVVLDKTGTITTGKPSLTDCF
71417434_Trypanosoma_cruzi          AIMVGTGVGAKNGILVKSGKSLEVVRQSRCIVFDKTGTITFGKLEVVFKK
15606387_Aquifex_aeolic             VVSISTSYSARNGILVRNRLALEKAKDIDVVVFDKTGTLTEGKLGVTEVI
6320475_Saccharomyces_cerevi        AIMVGTGVGAQNGVLIKGGEVLEKFNSITTFVFDKTGTLTTGFMVVKKFL
42573159_Arabidopsis_thalia         AMLVGTSLGARRGLLLRGGDILEKFSLVDTVVFDKTGTLTKGHPVVTEVI
75907770_Anabaena_variab            AILVGTGIGAERGLLIKGGDVLEKAHQLDTVVFDKTGTLTTGNPVVTDCL
16331210_Synechocystis_sp.          AILVGTSLGAEQGILIKGGNILEILQRTTVMAFDKTGTLTQGNLQLTDAV
15643086_Thermotoga_mariti          ALVTGTGLAAKKGLLIRNAEAIQTSKDVGVVLTDKTGTLTEGSPKVVDHN
46445712_Candidatus_Protoc          VLVVASGVGAQYGIFFKQASSIEQSEKIKFLFLDKTGTLTKGIPSVMGIY
45549362_Drosophila_melano          AVMVATGTGAINGVLVKGATALENAHKVKTVVFDKTGTITHGTPMTSKVT
55743071_Homo_sapien                AVMVGTGVAAQNGILIKGGKPLEMAHKIKTVMFDKTGTITHGVPRVMRVL
115444827_Oryza_sativa              AVMVATGKGASQGVLIKGGNALEKAHKVKAIIFDKTGTLTVGKPSVVQTK
```

```
56962006_Bacillus_clausi            SIMAGSGRAAEAGILFKGGEHLELANRVDTVVLDKTGTITEGKPVLMHVF
116330313_Leptospira_borgpe         SILTGSGKAATLGILFRTAEALEIAHKVDTIVFDKTGTLTLGKHILKDLE
118060384_Pseudomonas_mendoc        SVMVATGKAASSGVLFRDASAIENLCKIDTLIVDKTGTLTEGRPVFHSAE
110635001_Mesorhizobium_sp.         SIMTATGRGAQAGVLIKDAAALERFAKVDTLIIDKTGTLTEGKPRLTDMV
87306843_Blastopirellula_marin      SIMVGVGRGAAEGVLIKDAEVLERLESVNAIVVDKTGTLTAGKPKLTEFE
52841258_Legionella_pneumo          SIMVGVGKGAQSGVLIKNAEALERMEKVNTLVVDKTGTLTEGHPKLTRIV
53713321_Bacteroides_fragil         ALMVGIGKAASNHILIKDAVALEQMRKVDVVVLDKTGTLTEGHPTVSGWL
78188927_Chlorobium_chloro          AIIVGIGKGAAEGILIRNAESLEKLSRVNTVVFDKTGTITSGKPSLHAVV
67921918_Crocosphaera_watson        SIMVGTGKGAENGILIKGADSLELAHKIKAIVLDKTGTLTQGKPIVTNYI
21228430_Methanosarcina_mazei       AIMVGTGKGAENGILIKGGEALERAHKLDTIVFDKTGTLTEGTPKLTDIF
28210589_Clostridium_tetani         AIMVGTGKGADYGVLIKSGEALETTHKIDTVIFDKTGTITEGDPKVTDII
89340248_Mycobacterium_flaves       ALLVGTGRGAQMGVLIKGPEVLESTRKVDTVVLDKTGTVTTGKMALVDVI
83374897_Rhodobacter_sphaer         SIMVGTGRAAELGVLFRKGEALQRLSEIERVGFDKTGTLTEGRPQVTDVL
94972021_Deinococcus_geothe         SIMVGTGKAAELGVLFKGGGALEGLQDVRVVAVDKTGTLTKGKPELTDLV
76258102_Chloroflexus_aurant        AIMVGTGTGASHGILIRNAEALERAASLHAVVFDKTGTITHGRPAVTDVV
71366115_Nocardioides_sp.           AVMVGTGKAAEMGILVGDGAALETARRLTAVVLDKTGTITRGHPDLAEVV
CM4147_Cyanidioschyzon_merolae      AYTSALGVLARRGVLVKGGATLEASARCDKVAFDKTGTLTKGEPELHQIQ
92875650_Medicago_trunca            AIFCALTKAAISGLLLKGGDYLETLSRIKTVAFDKTGTITRGEFSVTDFS
15224717_Arabidopsis_thalia         ATFCALTKAATSGLLIKSADYLDTLSKIKIVAFDKTGTITRGEFIVIDFK
115469636_Oryza_sativa              AYATAISSLASKGILLKGGHVLDALSACQSIAFDKTGTLTTGKLMCKAIE
63253545_Arabidopsis_cp             AYATAISSCARKGILLKGAQVLDALASCHTIAFDKTGTLTTGGLTCKAIE
89897922_Chlamydophila_felis        AYLSSINACAKHGVLLKGGVVLDRLASCDSIVMDKTGTLTTGELTCIGCD
15605460_Chlamydia_tracho           AYLSAINACAKHGVLLKGGVVLDRLVSCNSVVMDKTGTLTTGDLTCSGCE
53712588_Bacteroides_fragil         GYFGGIGAASRLGILFKGGNYLDAITRVNTVVFDKTGTLTKGVFEVESCR
42526892_Treponema_dentic           GYFGGIGGAAKRGILIKGADYIDALSKADAVVFDKTGTLTKGVLKVMTLM
28211582_Clostridium_tetani         GFFGGIGAASRNGILVKGGNYLEALENTEIVVFDKTGTLTEGVFEVSEIK
19703603_Fusobacterium_nuclea       SFFSGIGAASRAGVLIKGGNYLETLSKVDTVVFDKTGTLTKGVFNVQKVV
46445921_Candidatus_Protoc          SIFSAIGHASSKGALIKGGKYLEAMGQVNAMAIDKTRTLTKGQPIVTHVI
83815585_Salinibacter_ruber         AVLSALARAGQGGVLVKGGGPLEHLGRLGAVAFDKTGTLTEGAPRVTDVR
94972049_Deinococcus_geothe         AMTSGISAGARRGLLIKGGAALETIGSVSTVAFDKTGTLTENKPQVTDVI
77462336_Rhodobacter_sphaer         AIASALASGARRGLLMKGGAVIEAAAAVRQVAFDKTGTLTRGRPEVTDVV
84488951_Methanosphaera_stadtm      GMVSSITSATRQGVLIKGSSYVEEMRNIKAVIFDKTGTLTEGKLEIMDID
89340253_Mycobacterium_flaves       TVVAAIGAASRHGALIKGGAAVEELGRIKVVALDKTGTLTRNQPRVVEVV
71368538_Nocardioides_sp.           TVVAAIGAASRIGALVKGGAALEALGRIRAVALDKTGTLTRNQPSVVEVA
76258037_Chloroflexus_aurant        ALLSAIAAGARRGVLFKGGVHLEELSRVRVIAFDKTGTLTFGKPVLTDLV
75910063_Anabaena_variab            TLLSGIANGARQGILFKNGAQLENIGKVRAIAFDKTGTLTTGQVQVSQVI
16331905_Synechocystis_sp.          ALLSGIANGARQGILFKNGAQLERIGRVRVIAFDKTGTLTTGKPEVVNIL
56962051_Bacillus_clausi            SIVSAIGNAAKKGVLVKGGVYLEEMASLKAIAFDKTGTLTKGIPVVTDFA
52841244_Legionella_pneumo          TLVAALGTASRNGILLKGGLYLEQLVKIKVIALDKTGTLTQGKPKVTDII
118065328_Roseiflexus_casten        SVVSALSAAARAGVLIKGGAALEALGGIRAVAFDKTGTLTTGKPTLVMVR
87311031_Blastopirellula_marin      SIVCGLTAAARNGVLIKGGLALETFGRVDVLAMDKTGTITTGVPAVTQVA
110347018_Mesorhizobium_sp.         TIVAALAGAAKQGVLVKGGTHLETPAHLKAIALDKTGTLTEGRPQVVEIV
118058132_Pseudomonas_mendoc        TIVSGLAAAARKGILIKGGVYLESGRHLALLALDKTGTLTHGKPVQTDCL
114564618_Shewanella_frigid         SIVSGLAAAARHGILIKGGVYLEEGRKLRWLALDKTGTITHGKPAQTDFV
                                        .                .:       .  *** *:*

42525989_Treponema_dentic           VLKIAACLEEHFPHSLARAVVREASYILAHGLASLRIGSAAGIIAIEDPV
114563186_Shewanella_frigid         VLSLAAAIEQGSLHPIALAFKPFYDHHIGLGIRAVCIGTAIAKIEMLDEL
19704525_Fusobacterium_nuclea       MIGLAGAAEEQSSHPLATAIMSEIKTVVSRGVETIRVGSKIGLIGVSDPP
16082160_Thermoplasma_acidop        ALMMAAAIESHSNHPVARAIVQYAKEMPGSGIEGISVISGRGVLKIGSKI
70606661_Sulfolobus_acidoc          DIQLASALESLSNHPIAKAIAQLHTEFQGQGVYGIIVGKKEGKIWISDEI
84489487_Methanosphaera_stadtm      LLKYVTISEVKSEHPIGKAIVKYSKMIPGKGVSAILSGTIVGIIGLSDVL
78189332_Chlorobium_chloro          FIQHAASLEALSEHPTARAITAAWKAITGKGVSGWYAGSKVGIIGMIDEL
71417434_Trypanosoma_cruzi          VMKVVGFVELLSNHPVAKAVADGISKVKGGGTEAVHVGNLRLLVALADEP
15606387_Aquifex_aeolic             FLKFVASVENHSEHVIARAIVSYAKAFPGKGVCGVCVGTLAGIIALADRI
6320475_Saccharomyces_cerevi        VLACIKATESISDHPVSKAIIRYCEYVLGKGIVSICIGNEFGLFEINDEV
42573159_Arabidopsis_thalia         VLMLAAAVESNTTHPVGKAIVKAATEEPGSGAVAVTVGTLAAVIRFEDKV
75907770_Anabaena_variab            LIQLAAAVESGTHHPLARAIQQAAHTEPGMGVSAVLLGNGAGLIGVQDTT
16331210_Synechocystis_sp.          LLTLAASVEQGTRHPLAQGLISSAETEAGQGVQGLLVGNQQGFLALRDTL
15643086_Thermotoga_mariti          LIRIVASIERNSNHPLAKAISELSEEIPGEGVRAYFVGKPVGFLAIEDPI
46445712_Candidatus_Protoc          VLQIAASLENCSSHPLAKAILNEANSFIGKGVTGYYLGSALGMITIMDEL
45549362_Drosophila_melano          ALTIVGAAEQNSEHPIASAIVHFAQAVPGCGIRVVLIGNRVCMFAVSDMV
55743071_Homo_sapien                VLAVVGTAEASSEHPLGVAVTKYCQAVPGCGIGCVLIGNRCGMIAIADAV
115444827_Oryza_sativa              LCDLAAGAEANSEHPLSKAIVEYTEVHPGAGVSAVLVGNKCGALSVSDPL
56962006_Bacillus_clausi            WLSYVNEIEKQSNHPLAEAVTVGLEELAGMGMKAVLIGNRAGFLSLADEL
```

```
116330313_Leptospira_borgpe         LLTLAASAEQGSEHPLSKAIVESAETIPGGGISAIVIGTDSAILSLADRI
118060384_Pseudomonas_mendoc        VLRLAASLDQGSEHPLAHAIVDHAESGSGIGVRGLQLGNTAGLLAVSDPI
110635001_Mesorhizobium_sp.         LLSLAAGLEKGSEHPLAEAIVEGAEAATGKGVSGVALGNLFGLIAVADPI
87306843_Blastopirellula_marin      VLRMAAAVEQNSEHPLARSIVDGADSIPGGGVSGVLIGNAAGLVAVADPI
52841258_Legionella_pneumo          ALALAAALEHQSEHPLANAIVHAAEAPTGKGVVGVAIGNAVALLVVEDPI
53713321_Bacteroides_fragil         FKNVLLAAELKSEHPLAGAIVSSLESITGKGIKVYWVGSHLAVVAIADQI
78188927_Chlorobium_chloro          LLTLAAMLESRSEHPLAQAIVEAASAQEGVGVTGITIRKPLGVLALSDTI
67921918_Crocosphaera_watson        ILGIAAAIEENSEHPLAEAIVNYAEAMGGQGVQGVQIGTQKGLFAIADAV
21228430_Methanosarcina_mazei       VLFTAASAEKGSEHPLGEAIVRGARSIPGKGIEAVLLGTRMGLVAVADTL
28210589_Clostridium_tetani         ILAIAASAEKGSEHPLGEAIVRELQAVPGHGIRALLLGNKQGIIAVADVL
89340248_Mycobacterium_flaves       LLRLAGALENASEHPIAHAIATAAANVEGKGVQGVVVGRERGVLVVADTV
83374897_Rhodobacter_sphaer         LLVLAAAVEARSEHPLAGAIRRAASSRPGHGAEAVRVGSLAGLIGVADRV
94972021_Deinococcus_geothe         VLQLVAAAEEQSEHPIARAIVDAAEAVPGYGLEAVQVGADAAVIAVADPI
76258102_Chloroflexus_aurant        LLQIAAAAESRSEHPLGVAIVKAAQAVSGAGVEAVLVGTLWGVIALADTV
71366115_Nocardioides_sp.           LLALVAAAEVGSEHPVGEAIVRGADSVPGHGVVAVLVGNTAGSVTVADTV
CM4147_Cyanidioschyzon_merolae      VLALAAGLEAHVVHPLGRAVAASVKVCAGAGLEGVRLGRLVAMLVFWDQM
92875650_Medicago_trunca            LLYWISSIESKSSHPVAGALVDYAQNFPGEGIFGIYIGNKVGVFSLVDAC
15224717_Arabidopsis_thalia         LLYWVSSVESKSSHPMAATIVDYAQNFPGEGIYGIFIGNKAGFFNLSDAC
115469636_Oryza_sativa              ALAVAAAMEKGTTHPIGRAVLDHSECLPGRGVVAASIGSVVTLFHFEDEP
63253545_Arabidopsis_cp             ALAVAAAMEKGTTHPIGRAVVDHSEYFPGRGLTAASLGSIVTLIHLEDQP
89897922_Chlamydophila_felis        FFPSILALEQSSMHPIAQAIVIYLCVIPGKGVRGAFVGKICALFYFKDTP
15605460_Chlamydia_tracho           FYSCVLAMEQSSSHPIAQAIVNYLTTIPGEGVSGAFVGRVVSLFYFRDVP
53712588_Bacteroides_fragil         LLRVVASIEKNSNHPIARAIVAYAEEIAGYGLMTVLVGNAIGCIILSDVL
42526892_Treponema_dentic           FLKLTALAEFNSHHPIAKAVQDCAYEKAGKGISVLLAGKNIGCLILTDTL
28211582_Clostridium_tetani         LIRLASLGESFSNHPIAKSIIKYYEEISGHGIRALLVGNHCGYIIISDRI
19703603_Fusobacterium_nuclea       FISLVTMAESGSNHPISKSIQKYYKEISGKGIEAILVGNKTGYIVISDEI
46445921_Candidatus_Protoc          LLSCAAGIEKLSEHPLAQSIVQAAESFIGKGAKAHCIGKLEGIIVLEDEI
83815585_Salinibacter_ruber         LLRAAVAAERLSDHPLAEAVVQYGKGITGRGIRAVHVGNALGVVGLADAP
94972049_Deinococcus_geothe         VVLLAAAVETGSAHPLAKAILARAKAISGKAVTALAVGSPLGLLAIRDEP
77462336_Rhodobacter_sphaer         VLALAAAVERGSAHPLAQAILARARSLAGRGAEALWVASPLGLLALRDEP
84488951_Methanosphaera_stadtm      LIEIASSLEKKSTHPIATAIVNHAKNIPGKGIVGYYAANEIGIITVMDKI
89340253_Mycobacterium_flaves       ALRWAAALEARSEHPLAQAILTAATAVPGHGLHGLRLGKPVAAIAVRDEL
71368538_Nocardioides_sp.           VLDIAAALESRSEHPLARAILADVEAVTGAGLTGARLGRPIGAVAVRDDL
76258037_Chloroflexus_aurant        LLTIVARAEQPSEHPIARAILQAAVAVTGMGVRATLVGSPLGLVAVMDRE
75910063_Anabaena_variab            VLKVAASLESSSEHPIAQAIVQAAQAIPGQGIVGVIVGNIMGIIAIADMI
16331905_Synechocystis_sp.          LLQIAAALESLSEHPIGEAIADFTQAQAGQGIIGAIVGKALGLIAVADTV
56962051_Bacillus_clausi            LFAIVAALENRSQHPLASAIVAKATALTGKGITGYYVGSPLAIVAVADEV
52841244_Legionella_pneumo          ILNAAATLESRSQHPMAQAILQQAKSLTGSGVLGFYIGNPLGLIAIADPL
118065328_Roseiflexus_casten        LLELACAVERRSEHPLAHAIVDAAAALPGHGIIGVVIGSHAGMLSVADTV
87311031_Blastopirellula_marin      VLRIAASLEQHSNHPLATAILQAAAEIAGRGASGAWVGSLLGLIVLGDQI
110347018_Mesorhizobium_sp.         LLGLAAALEARSGHPIARAILAKAQAITGRGVSGMWLGSRWGLVAVADAV
118058132_Pseudomonas_mendoc        LALWAASLAARSDHPVSQALARALEALPGRGTRGLHLGNHLMLFAVADTL
114564618_Shewanella_frigid         SRSIAASLAARSDHPVSKAVAQAANALPGRGVQGYHLGNQHALFAVADTI
                                                 *  .  .        . .            . . .

42525989_Treponema_dentic           RSEAKEVVSELHKLGINIIMLTGDGPQTARSIAEKTGHAQALPDTKADFI
114563186_Shewanella_frigid         RVDSLATVNALKAANIKLSIASGDNSGHVEFLASKLGHSGLSPQDKLALV
19704525_Fusobacterium_nuclea       RENIKKAINRLRNYGVDIVLLTGDLRQQAETIASRMSESELLPEDKAKNI
16082160_Thermoplasma_acidop        RPEAEETIAEMKEKGMKISVLTGDRSRDSASAVESIGLTGLSPEDKAAII
70606661_Sulfolobus_acidoc          RPEAKMVIEYLKKHGYDVEIATGDSSKFADEVAKSLGHKGLSAEEKAELV
84489487_Methanosphaera_stadtm      RETAPYVVNTLNDLDTDVELLTGDNHKAANYFASKVGYSELLPENKVEII
78189332_Chlorobium_chloro          RSDALPLINGLRTKGLKLHLFTGDNKGVARYIAERCGQAEQSPLDKAARI
71417434_Trypanosoma_cruzi          KEEAAGVVRFLHRQGFRVLMVTGDNEGVAAHVASAVGYAEALPTTKASIV
15606387_Aquifex_aeolic             KEESYEAVRSLKELGKKVVMITGDSEEVAKYVAKELEFARVLPHEKAQKI
6320475_Saccharomyces_cerevi        KHDSYATVQYLQRNGYETYMITGDNNSAAKRVAREVGYSDVSPTGKCDLV
42573159_Arabidopsis_thalia         REDAAQVVENLTRQGIDVYMLSGDKRNAANYVASVVGIAGVKPAEKKNFI
75907770_Anabaena_variab            RPDAQTTVDKLRQMGLRVMLLSGDRPEAANAIAQQLGMAGIPPAKKATFI
16331210_Synechocystis_sp.          RPEAKATIAQLKQWGIAPLLLTGDHPAIAQAIAMEVGQAQMTPQAKVAKI
15643086_Thermotoga_mariti          REDSPEAVRRLKEMGIEPVMITGDNEKTARAVARRLGHAGVKPSEKLDLV
46445712_Candidatus_Protoc          RKESMEVIRLLKLKGIYPILLTGDRKYVAETIAKKLGRFELLPDDKMQII
45549362_Drosophila_melano          KPEAHLAVYTLKRMGIDVVLLTGDNKNTAASIAREVGYAEVLPSHKVAKI
55743071_Homo_sapien                KQEAALAVHTLQSMGVDVVLITGDNRKTARAIATQVGFAEVLPSHKVAKV
115444827_Oryza_sativa              KPEAGRAISYLSSMGISSIMVTGDNWATAKSIAKEVGFAEIDPVGKAEKI
56962006_Bacillus_clausi            KPDSKEGINQLKRMGLDVIMVTGDNPQTAASVAEQVGKANVLPDGKRTLI
116330313_Leptospira_borgpe         KESTPTAVEKLKSLGMKIYIITGDNERTARAIANVCGLAEILPQEKAKEI
```

```
118060384_Pseudomonas_mendoc        KPTSQQAVSKLQSEDVKVIMATGDGLTTARAVAKQLGHGEVKPQDKEKLV
110635001_Mesorhizobium_sp.         KESTAGAIRALHESGLRIIMATGDNERTAKAVALKLGRADMLPEGKNKLV
87306843_Blastopirellula_marin      KDSTPEAIRQLHDLGLKVIMLTGDNQRTAASVAKQLGTAEVRPEQKQQRV
52841258_Legionella_pneumo          KSSTPETILELQQSGIEIVMLTGDSKRTAEAVAGTLGVAEIMPEDKSRIV
53713321_Bacteroides_fragil         KPTSAEAVKELKRQGIDICMLTGDGQRTALAVSGKLGVADALPDDKEEFV
78188927_Chlorobium_chloro          KPEAASAVATLKHKGIRVLMLSGDHQAAANAVAAQAGIADVLPHDKANKV
67921918_Crocosphaera_watson        KPSSIEAVKKLKKMGLEVIMLTGDNQQTAQAIADEVGFAEVRPDEKANKI
21228430_Methanosarcina_mazei       KENSKEAVETLKNMGIEVVMITGDNSITAGAIASEVGLAEVLPEDKANEI
28210589_Clostridium_tetani         KENSKKAIEELHKMNIEVVMITGDNKRTAEAIGKKVGLAEVLPEDKALWV
89340248_Mycobacterium_flaves       KPTSAQAISQMRDLGLTPVLLTGDNEAVARQIAAEVGIAEVMPKDKVDVI
83374897_Rhodobacter_sphaer         KPTARAAVEALGRLGLPAAMVTGDAPGVARAIGRELGHAGVLPGGKVEEV
94972021_Deinococcus_geothe         KKGSQEAVNALHHMGLKVAIITGDNARTASAIARQLGLAEVLPSGKSEAV
76258102_Chloroflexus_aurant        KPTAAEAVAKLHQAGIEVALLTGDNQRTAAAIAAAVGYAEVKPDEKAAIV
71366115_Nocardioides_sp.           KESSAAAVAALERLGLEVWMVTGDNATTARAVAEQVGMADVRPADKARRV
CM4147_Cyanidioschyzon_merolae      REEAPACIAQLRTQGYSVQLLTGDSKETALAIAEAVGRYALRPEDKMLFV
92875650_Medicago_trunca            RSGALEAMEELKLLGVRSVMLTGDSSQVAMYVQSQLKHAELLPHEKAKLI
15224717_Arabidopsis_thalia         RSGVSQAMAELKSLGIKTAMLTGDNQAAAMHAQEQLGHGDLLPEDKSRII
115469636_Oryza_sativa              RSGVCEVISTLRDKAKRIMMLTGDHESSALRVAKAVCHCCLKPEDKLNKV
63253545_Arabidopsis_cp             RPGVSGVIAELKSWARRVMMLTGDHDSSAWRVANAVGYCNLKPEDKLNHV
89897922_Chlamydophila_felis        RPDAGKIIKELKDEGYSISMLTGDHQVSAENTAKLLGFSNLSPDDKLEKV
15605460_Chlamydia_tracho           RHDAADIVSYLKKNGYPVCMLTGDHRISAENTARLLGFYDLTPDNKLSKI
53712588_Bacteroides_fragil         KDDASDTVKALKELNINIQILSGDKQSIVTIFANKLGYGDLLPEGKVEHI
42526892_Treponema_dentic           KNEAREAISDLNKLGLRVEILTGDNEKSAKKIAEDLGTSMLLPHEKVSRF
28211582_Clostridium_tetani         KEDSKKTIKELKNKGIKTIMLTGDNKSAGEKIGKELLYTDLLPENKIEEL
19703603_Fusobacterium_nuclea       KKDAEKAIKGLKDIGIKSIMLTGDVEKVAKKVGEDLGYSNLLPQDKVSKF
46445921_Candidatus_Protoc          RSDSAQFIADLKQLQVYPIMLTGDHLNVAHSIAQQVGKANLLPQDKSKAI
83815585_Salinibacter_ruber         RPEAAGVVEQLRRAGIRMVMLSGDNQRVVDAAAERLGRGDLLPSDKVDTV
94972049_Deinococcus_geothe         RADAKEAVARLKGLGVRSLMLTGDNARTGNAIARDLGEAELLPEDKLQRI
77462336_Rhodobacter_sphaer         RGDAAEAVRQLGRMGVSATILTGDNARTAEAIAARLGRAQMRPDDKLAAI
84488951_Methanosphaera_stadtm      RDESEEVIRNLNNAGVKTVMLTGDNKIAAHAVSDKIGYSDLLPEDKLNIV
89340253_Mycobacterium_flaves       RPEAADTVSLLKRLGITVAMLTGDNTRTANAVAAEAGHSELLPEDKAALL
71368538_Nocardioides_sp.           RPEAAEVVARLRAGGYQVAMLTGDNERTAAALAAQAGHAELRPEDKSAII
76258037_Chloroflexus_aurant        RPDAARQIAALRAVGIRIVMLTGDNPQVAEAMAQRLGYAGLLPADKLQRV
75910063_Anabaena_variab            RPEAAATIARLRKLGIQIVMITGDNQRTAESVAQAVGFAELLPEDKLDVI
16331905_Synechocystis_sp.          RPTAAQAIARLKRLGIRIVMLTGDNSRTAHSIAQQVGYAELLPEDKVDVI
56962051_Bacillus_clausi            RPSSKEVIRKLHEAGIKTVMLTGDNKQTAHAIGQAVGQAELMPEDKLRFI
52841244_Legionella_pneumo          RPTARQMVSNLKSMGIRVVMLTGDNLITASSIGEQAGFSDLSPEDKTRKI
118065328_Roseiflexus_casten        RPESREAVALLREAGIHIIMLTGDRRETAYAVAADVGRAEVLPAHKAETV
87311031_Blastopirellula_marin      RAGAADVIAQLHKLGVDVAVISGDNNAAVEQVAAEVGRGEQLPQDKIEAV
110347018_Mesorhizobium_sp.         RPEAKDIVTALHRAGIHVVMLTGDNRATAEAIAKQTGQAELLPGDKVAAV
118058132_Pseudomonas_mendoc        RQSSREAVQQLHALGVRTCMLSGDNAHTVAAIAEQVGRGDLLPADKLAWV
114564618_Shewanella_frigid         KESSRSAIAELHALGINTMMLTGDNPHTAQAIAAQAGQGNLLPDDKLREV
                                    :      :  :        : :**                  .  *   .

42525989_Treponema_dentic           KELIVMIGDGINDSPALSEADVGIAMGQASSIAGETADILLPDDGLRALP
114563186_Shewanella_frigid         NQLVAMFGDGINDAPVLAGANLSVAMGSGSAIAKNSADLILLGDHLSRFN
19704525_Fusobacterium_nuclea       LKFVIMIGDGVNDAPALSYANVGVALGSRTDVAMEAADITITQDNPLLVP
16082160_Thermoplasma_acidop        RKYVMFVGDGINDVMAIDQADVGVAMGSGSDITRSQGDFVLLRNDLRAIL
70606661_Sulfolobus_acidoc          RE-TVFIGDGVNDAIALKESHVGIAISSGTDIAKYAGDIIISN--LSQLP
84489487_Methanosphaera_stadtm      EQLVCMVGDGINDAPALKTADVSVAMGGGSDIVIDAADIALLGDDIENLT
78189332_Chlorobium_chloro          RALVMMVGDGINDAPALTEATVGVTLGNASAIALDSAGVAILHDDLRLIT
71417434_Trypanosoma_cruzi          RQLVIFVGDGINDSPALAQADVGIALGAGTQIAIEAADAVLISNSLVDIL
15606387_Aquifex_aeolic             KELVAMVGDGVNDAPALIQADVGIAIGSGTDIAIESADVILVKSDPRDVV
6320475_Saccharomyces_cerevi        KKIVAVVGDGINDAPALALSDLGIAISTGTEIAIEAADIVILCGNLRGLA
42573159_Arabidopsis_thalia         NELVAMVGDGINDAAALASSNVGVAMGGGAGAASEVSPVVLMGNRLTQLL
75907770_Anabaena_variab            QELVAMVGDGINDAPALSQADVGIALHSGTDVAMETAQIVLMRDRISDVV
16331210_Synechocystis_sp.          KAMIAMVGDGINDAPALAQADVGISLSGATAVAMETADVVLMRSHLSDVL
15643086_Thermotoga_mariti          RSYVAMVGDGMNDAAALKGADVGIAIGSGTDLAIDSADIIITKGGISKVV
46445712_Candidatus_Protoc          REAVGMIGDGINDAPALAEADVSFALAFGSDIAIEVADITLIRNDLMSLV
45549362_Drosophila_melano          QRIVAMVGDGVNDSPALAQADVGITIAAGTDVAAEASDIVLMRNDLLDVV
55743071_Homo_sapien                QELVAMVGDGVNDSPALAQADMGVAIGTGTDVAIEAADVVLIRNDLLDVV
115444827_Oryza_sativa              KDLVAMVGDGINDSPALAAADVGLAIGAGTDVAIEAADIVLMRSSLEDVI
56962006_Bacillus_clausi            SAMVAMVGDGINDAPALVTADVGIAIGSGADVAVESADIALISGSLKKVG
116330313_Leptospira_borgpe         KKLVAMVGDGINDAPALVIADLGISMGTGTDVAMESSDVVIVNGDLVSIV
118060384_Pseudomonas_mendoc        ADLVAMAGDGINDAPALARSDVGIAMGTGTDVAMNSAQVTLVKGDLMGIL
```

```
110635001_Mesorhizobium_sp.         DELVAMAGDGVNDAPALAAADVGIAMGTGADVAVESAGITLVKGDLNGIV
87306843_Blastopirellula_marin      TQLVAMAGDGVNDAPALAAADVGVAMGAGSDVAIESAGVTLVKGDLRGIV
52841258_Legionella_pneumo          SELVAMAGDGVNDAPALAKADIGIAMGTGTDVAIESAGVTLLHGDLRGIA
53713321_Bacteroides_fragil         RELVAMVGDGINDSQALALADVSIAMGKGTDIAMDVAMVTLMTSDLLLLP
78188927_Chlorobium_chloro          MELVAMVGDGINDAPALAQADVGIAMATGTDVAMETAGITLLKGDINKIP
67921918_Crocosphaera_watson        KEIVAMVGDGINDAPALAQADVGMAIGTGTDVAMSASDITLISGDLQGIV
21228430_Methanosarcina_mazei       KKLVGMVGDGINDAPALIQSDVGIAMGAGTDVAMESAKIVLIKNDPIDVV
28210589_Clostridium_tetani         KKLVAMVGDGINDAPALAQSDVGIAIGSGTDVAIESADIVLMRSDLMDVP
89340248_Mycobacterium_flaves       ARLVAMVGDGVNDAPALAQADLGLAMGTGTDVAIEASDITLVRGDLRSAV
83374897_Rhodobacter_sphaer         TAMAAFVGDGINDAPALAAAEVGIAIGTGTDVAIEAADVVLMSGDPVGVA
94972021_Deinococcus_geothe         KELVAFVGDGINDAPALAQADVGLAIGTGTDVAVETADVILMSGDLRGVP
76258102_Chloroflexus_aurant        ARLVAMVGDGINDAPALAQADVGIAMGSGTDVAMETADITLMRSDPRGVV
71366115_Nocardioides_sp.           AELVAMVGDGVNDAPALAQADLGIAIGTGTDVAIAASDITLVGGDLHGIA
CM4147_Cyanidioschyzon_merolae      KQQLVMVGDGMNDAPALALATTGISIGFHSATAVSAADVVLVDGYIRRIA
92875650_Medicago_trunca            ENFIAMIGDGINDAPALATADIGISMGIGSALANETSNAILMSNDIRKVP
15224717_Arabidopsis_thalia         QEFTAMVGDGVNDAPALATADIGISMGIGSALATQTGNIILMSNDIRRIP
115469636_Oryza_sativa              KAVLIMVGDGINDAPALAAATVGIVLAQASATAVAVADVLLLQDNICGVP
63253545_Arabidopsis_cp             KNILIMVGEGINDAPALAAATVGIVLAQASATAIAVADILLLRDNITGVP
89897922_Chlamydophila_felis        RELIVMVGDGINDAPALAQATVGVAMGEGSATAVEAADIVLLHDAISLLP
15605460_Chlamydia_tracho           QELIMMIGDGINDAPALAQATVGIAMGEGSATAIEAADVVLLNQGLSSLP
53712588_Bacteroides_fragil         ERLIAFVGDGMNDAPVLALSDVGIAMGGGSDAAIETADVVIQTDQPSKVA
42526892_Treponema_dentic           EEIVIFVGDGINDAPVLARSDAGIAMGGGSDAAIEAADVVLMNDNPRLVA
28211582_Clostridium_tetani         EKLLVFVGDGVNDAPVLARADIGIAMGGGSDAAIEASDIVIMQDNPFKII
19703603_Fusobacterium_nuclea       EEIVVFVGDGINDAPVLARADVGIAMGAGSDAAIEAADVVIMTDEPSKIV
46445921_Candidatus_Protoc          EELVAMFGDGVNDAPALALSSVGISMSSGSDTALEAASIVILNDNLSLIP
83815585_Salinibacter_ruber         RALVAMVGDGVNDAPAMANATVGVAMGAGSDAALETADVALMADDLSQLP
94972049_Deinococcus_geothe         AALVAMVGDGINDAPALAQSDVGIAMGGGTDVALETADAALLRHSVTGVA
77462336_Rhodobacter_sphaer         REMVMMIGDGINDAPALKAASVGVAMGSGTDVALETADAAILRDRVTDVP
84488951_Methanosphaera_stadtm      DTIVAMVGDGINDAPALARSNVGIAMGAGSDVAIETADVALMQDDLTKLP
89340253_Mycobacterium_flaves       ETLIAMVGDGINDAPALATANIGIAMGAGTDVAIETADVALMGEDLRHLP
71368538_Nocardioides_sp.           ARLTAMVGDGVNDAPALATADVGIAMGAGSDVAIETADVALMGEDLRHLP
76258037_Chloroflexus_aurant        EQLVAMVGDGVNDAPALAAATVGIAMGAGTDAALETADLVLMSDDLSAIV
75910063_Anabaena_variab            RSLVAMVGDGINDAPALAQASVGIAMGVGSDVALETADIVLMADKLEKID
16331905_Synechocystis_sp.          RQLVAMVGDGINDAPALAQASVGIAMGAGSDVALETADIVLMADRLERLE
56962051_Bacillus_clausi            KQWVAMVGDGVNDAPALAAATVGIAMGGGTDTALETADIALMGDDLKKLP
52841244_Legionella_pneumo          EELVMMVGDGVNDAPALAAAHVGVAMGAGTDVALETADVALMGDNLLKLP
118065328_Roseiflexus_casten        ENLVAMVGDGINDTPALASATVGIAIGAGANQAMETADVTLMSNDLRRLP
87311031_Blastopirellula_marin      RQLIAMIGDGVNDGPALAAADVGIAMGAGADAAIETADVALMSDEINKLP
110347018_Mesorhizobium_sp.         EDLVAMVGDGVNDAPAMGRANLGIAMGAGSDAAIETADVALMSDDLSKLP
118058132_Pseudomonas_mendoc        ETRVGMVGDGINDAPALAKAQIGFAMGAGTDTAIETADVALMDDDLRKIP
114564618_Shewanella_frigid         ELLVGMVGDGINDAPALARADIGFAMGAGTDTAIETADVALMDDNLGKIP
                                         . *:*:**  .:  :  .. :   :  .   .   :

42525989_Treponema_dentic           ILRRIATGLIKRINVNNKAIIGINSALIAGELAGSIPPATAALVHNGSTV
114563186_Shewanella_frigid         DAIDVAKQTQRIIHQNLLWAFAYNIVILPLAVTGHVAPYIAAIGMSVSSL
19704525_Fusobacterium_nuclea       GVIGLSKNTVKTIKENFAMVIGLNTFALVLGATGILAPIYASVLHNSTTI
16082160_Thermoplasma_acidop        SIFDISSKTISKVKQNIIWAISYNSALIPVAAGVLFLPMLAAFAMGMSSS
70606661_Sulfolobus_acidoc          YLLSQSTRIVRKVKENVLWALVYNIILMPLAAGLLISPEIAALAMSMNSV
84489487_Methanosphaera_stadtm      YLKKLSNATLFTIHLSITISMIINAVAIVCSVLGLLDPISGALVHNIGSC
78189332_Chlorobium_chloro          TLLNDASRCFSIIRQNLVWAFSYNLIALPLAVSGTLHPIVSALLMATSSL
71417434_Trypanosoma_cruzi          NLRALSIATVKRVYGNFFWAFGYNLCMLPLASGMLLSPILAAAAMVFSSI
15606387_Aquifex_aeolic             KVIKLSQVTVSKMLQNLFWAVGYNVITLPLATGLGLKPAVGAIFMSASTV
6320475_Saccharomyces_cerevi        NAIDISLKTFKRIKLNLFWALCYNIFMIPIAMGVLLPPMLAGLAMAFSSV
42573159_Arabidopsis_thalia         DAMELSRQTMKTVKQNLWWAFGYNIVRIPIAAGVLLTPSMAGALMGVSSL
75907770_Anabaena_variab            ESIHLSRATFNKIRQNLFWAFAYNTIGIPLAAGVLLSPSGAAALMAFSSV
16331210_Synechocystis_sp.          KALTLSRSTVAKIKQNLLWALGYNLLAIPLAAGAFLTPAIAAAMMASSSI
15643086_Thermotoga_mariti          DAIEISRKTFKIIKQNLFWAFFYNVIAIPMAMMGLLHPVIAELAMAFSSI
46445712_Candidatus_Protoc          DAIDLSQQTMRKMKYNLFLAFIYNVLAIPLAAFGLLNPMIAAGTMAMSSV
45549362_Drosophila_melano          ACLDLSRCTVRRIRYNFFFASMYNLLGIPLASGLFLLPWMASVAMAASSV
55743071_Homo_sapien                ASIHLSKRTVRRIRINLVLALIYNLVGIPIAAGVFLQPWMGSAAMAASSV
115444827_Oryza_sativa              TAIDLSRKTLSRIRLNYVWALGYNVLGMPVAAGVLLPPWLAGACMAASSV
56962006_Bacillus_clausi            SALTVSHLTMANIKQNLFWALIYNSIGIPIAAAGLLAPWLAGTAMAFSSV
116330313_Leptospira_borgpe         NAFSISKKTVYNIRQNLFWALFYNTLGIPIAAAGLLTPWIAGGAMALSSV
118060384_Pseudomonas_mendoc        RARTLSVATVKNMRQNLAFAFLYNAMGIPLAAGLLLSPLIAALAMSVSSA
110635001_Mesorhizobium_sp.         RARTLAQATIRNIKQNLFFAFVYNALGVPIAAGVLLSPMFAAAAMSLSSV
```

```
87306843_Blastopirellula_marin      RSVQLSRRTMQNIRQNLFFAFAYNVIGIPIAAGVLLNPMIAAAAMSFSSV
52841258_Legionella_pneumo          KARRLSESTMSNIRQNLFFAFIYNVLGVPLAAGVLLSPMIAAAAMALSSV
53713321_Bacteroides_fragil         RAFELSKQTVKLIHQNLFWAFIYNLIGIPIAAGILLNPMLASAAMAFSSV
78188927_Chlorobium_chloro          QALNLAHATMRVIRQNLFWAFFYNIICIPLAAGAFLNPAFAGLAMAGSSV
67921918_Crocosphaera_watson        TAIELSRATMKNIRQNLFFAFIYNTLGIPIAAGILLNPMIAGAAMAFSSV
21228430_Methanosarcina_mazei       AALRLSRLTIRKIKQNLLWAFGYNTIGIPIAAGILITPELAAAFMALSSV
28210589_Clostridium_tetani         TAILLSKKTIKNIKENLFWAFAYNTIGIPIAMGLLLNPMIAAAAMSFSSV
89340248_Mycobacterium_flaves       DAIRLSRRTLSTIKTNLFWAFAYNVAAIPVAALGMLNPMLAGAAMAFSSV
83374897_Rhodobacter_sphaer         TAVALSRRTMRNIRQNLGWAFGYNVALIPVAAGVLLSPMLAAGAMAFSSV
94972021_Deinococcus_geothe         NAFALSRATLRNIRLNLFWAFAYNIVLIPVAAGVLLSPVLAAAAMGFSSV
76258102_Chloroflexus_aurant        QAIALSRATVRTIRWNLFWAFAYNVLLIPVAAGVFLSPVLAAAAMAFSSV
71366115_Nocardioides_sp.           SAIDISRRTVTTIKQGLVWAFAYNVLLVPVAAGALLDPVLAGAAMAMSSV
CM4147_Cyanidioschyzon_merolae      WYLQKSRQTQRVMTQNVAIALILIVSTAIGAVSGGLPLWAAVLLHEGGTL
92875650_Medicago_trunca            EAIRLARKTTRKLVENVIISVGFKCAILALAIAGYPLVWLAVLTDVGTCL
15224717_Arabidopsis_thalia         QAVKLARRARRKVVENVCLSIILKAGILALAFAGHPLIWAAVLVDVGTCL
115469636_Oryza_sativa              FCIAKARQTTSLVKQSVALALSCIVFAALPSVLGFLPLWLTVLLHEGGTL
63253545_Arabidopsis_cp             FCVAKSRQTTSLVKQNVALALTSIFLAALPSVLGFVPLWLTVLLHEGGTL
89897922_Chlamydophila_felis        WIIKKAKKTRRIVTQNLGLALAIILLVSWPASLGIIPLWLAVILHEGSTI
15605460_Chlamydia_tracho           WLIDKAKKTRRIVSQNLALALAIILFISGPASMGVIPLWLAVILHEGSTV
53712588_Bacteroides_fragil         TAIKVGRCTRRIIWQNVLLAFGVKLLVLILGASGIATLWEAVFADVGVTL
42526892_Treponema_dentic           AAVKHARFTRKIVWQNIALAFGIKIGFLTLGALGLANLWAAVFADVGVAL
28211582_Clostridium_tetani         KGIEIAKRTKKIVWENIIFSLGIKFVVLSLGALGFANMWAAVFADVGVTL
19703603_Fusobacterium_nuclea       TAIKSSKKTMKIAMQNIILAFGIKIIALILSALGIADMWMAVFADTGVTI
46445921_Candidatus_Protoc          YLIKLGRKTLSTIKINITFALSVKILFIILALLSISNLALAIFADVGVTL
83815585_Salinibacter_ruber         FAVGLSRQTRRVITQNLWIALGMVAVLVPATIAGLIGPAV--VLHEGSTL
94972049_Deinococcus_geothe         ELVELSRAVMTNIRQNVAFALGLKAIFLVTTLLGITGLWPAILSDTGATV
77462336_Rhodobacter_sphaer         ATIRLARAAMRNIRQNIAVALGLKGVFLVTSVLGITGLWIAILADTGATV
84488951_Methanosphaera_stadtm      YLFSLSTKTMSIIKQNIITAISVKFLFVILAILGLITLMMAVIGDMGLTL
89340253_Mycobacterium_flaves       QVLAHSRRARRIMVQNIAFSLAIIAVLIPLAAFGVLGLATVVLIHETAEV
71368538_Nocardioides_sp.           HTLTHARRARSIMLQNVGLSLVLIAILIPLAALGVLGLAEVVLVHELAEI
76258037_Chloroflexus_aurant        YALRLSRQTQRVVWQNIVFALAVVVVLVLTTLTVGVPLPLGVVGHEGSTI
75910063_Anabaena_variab            VAMKLGRRSHSIIQQNIVVALGFIGLLLIGNFLGNINLPIGVIGHEGSTV
16331905_Synechocystis_sp.          HAIRLGRRAQGVVKQNIVFALGFVMILLIANFAGNITLPFGVLGHEGSTV
56962051_Bacillus_clausi            FTVKLSRKTLNIIKANIAFAIAIKLAALLLVVPGWLTLWIAILSDMGATL
52841244_Legionella_pneumo          YLIAFSRRTWNIILQNLGLSIIVISSLVVGAITGYFTLPIAVLAHEISEL
118065328_Roseiflexus_casten        FVFALARAARRTIMLNVAFSIAVKVVFLAIVAAGWSTMWMAVFADMGTSL
87311031_Blastopirellula_marin      WLVGLARRTLRMIQFNIAFALCVKVIFVLLAIFGMTNLWLAIIADTGVSL
110347018_Mesorhizobium_sp.         WLVRHSRATLAVIRQNVAFSIAVKLLFTALTVIGLASLWGAIAADVGASL
118058132_Pseudomonas_mendoc        AFVRLSRQTHAILVQNIVLALGIKAIFLALTLAGEATMWMAVFADMGVSL
114564618_Shewanella_frigid         TFVRLSRATAQVLMQNIVLALGIKAVFLVLTFTGQATMWMAVFADMGASL
                                          .         .

42525989_Treponema_dentic           AIGMSAMRSY
114563186_Shewanella_frigid         IVVSNSLRLL
19704525_Fusobacterium_nuclea       LVVLNSLKLL
16082160_Thermoplasma_acidop        TVVLNSIRLR
70606661_Sulfolobus_acidoc          SVVLWSLVQ-
84489487_Methanosphaera_stadtm      AVVMLAASLY
78189332_Chlorobium_chloro          VVVSNSLRLR
71417434_Trypanosoma_cruzi          SVLLSSLSIR
15606387_Aquifex_aeolic             IVALNSMLMK
6320475_Saccharomyces_cerevi        SVVLSSLMLK
42573159_Arabidopsis_thalia         GVMTNSLLLR
75907770_Anabaena_variab            SVVTNSLLLR
16331210_Synechocystis_sp.          VVVLNALALR
15643086_Thermotoga_mariti          TVTLNSMRVR
46445712_Candidatus_Protoc          SVIANALLLR
45549362_Drosophila_melano          SVVCSSLLLK
55743071_Homo_sapien                SVVLSSLQLK
115444827_Oryza_sativa              SVVCSSLLLQ
56962006_Bacillus_clausi            SVVLNALRLQ
116330313_Leptospira_borgpe         SVVLNALRLQ
118060384_Pseudomonas_mendoc        SVVFNALRLK
110635001_Mesorhizobium_sp.         SVIANALRLR
87306843_Blastopirellula_marin      SVIANSLRLR
```

52841258_Legionella_pneumo	SVIINALRLK
53713321_Bacteroides_fragil	SVVLNSLSLA
78188927_Chlorobium_chloro	SVVSNSLRLK
67921918_Crocosphaera_watson	SVVSNALRLR
21228430_Methanosarcina_mazei	SVTTNSLLMK
28210589_Clostridium_tetani	SVLLNALRLK
89340248_Mycobacterium_flaves	FVVGNSLRLR
83374897_Rhodobacter_sphaer	FVLTNALRLR
94972021_Deinococcus_geothe	FVLSNALRLR
76258102_Chloroflexus_aurant	FVVTNSLRLR
71366115_Nocardioides_sp.	SVVTNALRLR
CM4147_Cyanidioschyzon_merolae	LVGLSGLRLL
92875650_Medicago_trunca	LVILNSMLIL
15224717_Arabidopsis_thalia	LVIFNSMLLL
115469636_Oryza_sativa	LVCLNSIRAL
63253545_Arabidopsis_cp	LVCLNSVRGL
89897922_Chlamydophila_felis	IVGLNALRLL
15605460_Chlamydia_tracho	IVGLNALRLL
53712588_Bacteroides_fragil	IAIMNAVRIQ
42526892_Treponema_dentic	LAVFNSLRAK
28211582_Clostridium_tetani	IAVLNSMRAL
19703603_Fusobacterium_nuclea	LAVLNSFRAL
46445921_Candidatus_Protoc	LVILNSLKLM
83815585_Salinibacter_ruber	LVVGNALRLL
94972049_Deinococcus_geothe	LVTANALRLL
77462336_Rhodobacter_sphaer	LVTLNALRLL
84488951_Methanosphaera_stadtm	LVILNSFRIG
89340253_Mycobacterium_flaves	FVILNAIRAA
71368538_Nocardioides_sp.	FVIGNGVRAG
76258037_Chloroflexus_aurant	IVVLNGLRLL
75910063_Anabaena_variab	LVTLSGLRLL
16331905_Synechocystis_sp.	IVTLSGLRLL
56962051_Bacillus_clausi	LVALNSIRLI
52841244_Legionella_pneumo	IVIASGLRML
118065328_Roseiflexus_casten	LVTLNGLRLL
87311031_Blastopirellula_marin	LVIANSLRLL
110347018_Mesorhizobium_sp.	LVVLNGLRLL
118058132_Pseudomonas_mendoc	LVVFNGLRLL
114564618_Shewanella_frigid	LVVGNGLRLL
	.

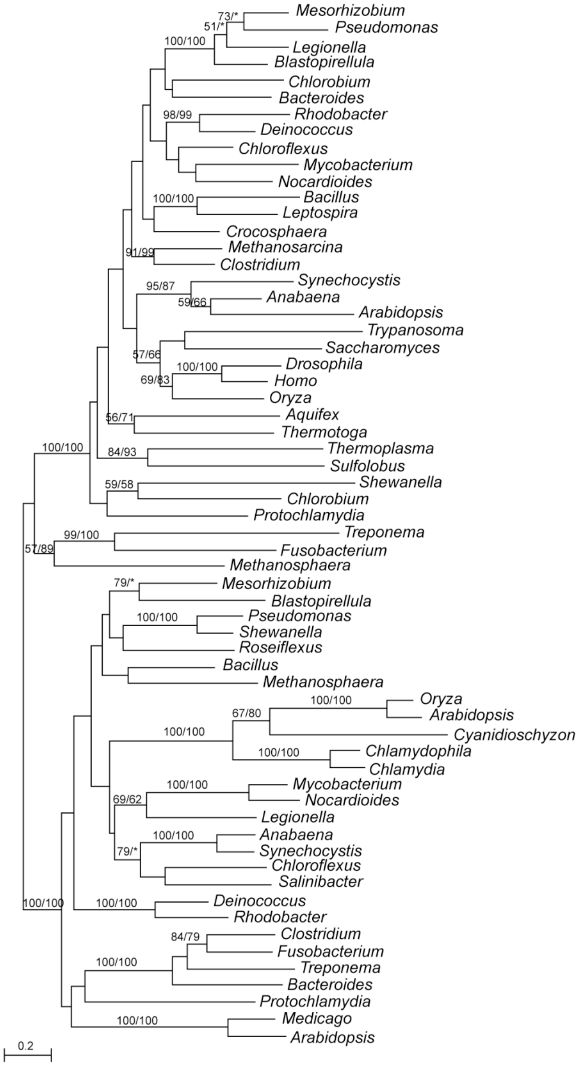

Figure 21. Phylogeny of Metal P-type ATPase. There are multiple copies of this gene. Topology of one paralogous copy (lower part of the tree) shows that red algal, green plants and chlamydial sequences form a strongly supported monophyletic group that is distinct from cyanobacterial homologs. The *Arabidopsis* sequence (GI 63253545) is experimentally annotated as a heavy metal P-type ATPase, localized in the chloroplast envelope and essential for growth under adverse light conditions.
